# Investigation of olive leaf extract as a potential environmentally-friendly corrosion inhibitor for carbon steel

**DOI:** 10.1038/s41598-023-43701-x

**Published:** 2023-10-10

**Authors:** Sherifa Elhady, Hatice Inan, Mahmoud Shaaban, Irene S. Fahim

**Affiliations:** 1 Smart Engineering Systems Centre, Nile University, Cairo, Egypt; 2 Mechanical Engineering Program, School of Engineering and Applied Sciences, Nile University, Cairo, Egypt

**Keywords:** Biochemistry, Biogeochemistry, Health care, Energy science and technology

## Abstract

Corrosion constitutes a significant issue in industries that handle metals. Corrosion inhibitors with a low impact on the environment provide a significant economic benefit in various engineering applications. In this work, the effectiveness of olive leaves extract is evaluated as a cost-effective and environmentally-friendly corrosion inhibitor. The corrosion of carbon steel in different concentrations of hydrochloric acid (0.1, 1.0, and 2.0 M) when protected by an aqueous solution of olive leaf extract of concentrations ranging from 10 to 60 ppm is investigated. A green extraction process based upon water extraction is used to ensure minimum impact on the environment. Results show that the corrosion inhibition efficiency increased as the concentration of the olive leaf extract increased. An analysis of variance showed a significant effect of acidic molarity, temperature, and inhibitor concentration on the corrosion rate. A significant statistical model indicates that the inhibitor exhibits higher efficiencies at higher acidic molarity. Results of SEM and EDX also demonstrated that a protective film of the inhibitor on the specimen surface plays a role in corrosion inhibition, suggesting that the inhibitor molecules are adsorbed at the interface between the carbon steel and the acid solution. The study provides an insight on the corrosion mechanism and highlights the potential of olive oil extract as an eco-friendly alternative to traditional corrosion inhibitors.

## Introduction

Corrosion is a characteristic phenomenon that reveals itself in metals, where they tend to revert to their original state, metal oxide due to environmental factors^1,2^. This process is a significant challenge in applications involving steel structures^3^, leading to equipment breakdown, reduced production, and safety concerns^4^. For instance, corrosion in pipelines used for oil and gas transportation is a major issue and has been observed to arise within pipelines, with an estimated annual cost of $1.372 billion^5^. In some estimates, the cumulative annual cost of corrosion is valued at $1.372 billion, consisting of the overall expenses associated with the surface pipeline and facility costs ($589 million), down-hole tubing costs ($463 million), and capital expenses ($320 million). In addition, the cumulative yearly expenditure on corrosion amounts to a staggering $70 billion; with a potential saving of 14% achievable through the direct application of currently available anti-corrosion methods^6^.

To mitigate corrosion, chemical inhibitors are commonly used, but their negative effects on human health and the environment have led to a growing interest in exploring natural substances as eco-friendly corrosion inhibitors^7^. The utilization of natural resources, known as green inhibitors, has gained momentum due to the imperative to minimize environmental impacts. Green corrosion inhibitors are used in many industries, including oil and gas, automotive, and construction to protect metal equipment and infrastructure from rust and corrosion caused by exposure to harsh environments such as saltwater, hydrogen sulfide gases, and acidic conditions, these inhibitors help extend the life of pipelines, storage tanks, and other equipment, minimizing maintenance and replacement costs^8^.

Plant extracts have been extensively investigated as environmentally friendly corrosion inhibitors since the 1960s^9,10^. Among the array of natural products explored for their potential as corrosion inhibitors for various metals are peels, alum sludge, and olive leaves^11–14^.

Olive leaf extract has garnered significant attention in Turkey and Egypt^15^ because olive waste products are found in abundance in both countries as olive-growing and exporting countries. It is known that olive leaves contain antioxidants and tannins, which strongly contribute to its use as an inhibitor^12^.

Extensive research has been conducted to address the environmental concerns associated with synthetic corrosion inhibitors by exploring natural resources as innovative and environmentally-friendly alternatives. Among countries with a rich agricultural tradition, such as Egypt and Turkey, the cultivation of olive trees has been prominent for centuries. Olive trees thrive in elevated temperate and arid regions, resulting in significant agricultural waste in the form of olive leaves. The potential utilization of olive leaves as a corrosion inhibitor offers a promising opportunity to transform this waste into a valuable resource while effectively addressing environmental concerns.

Olive leaves are rich in various phenolic compounds, including oleuropein, hydroxytyrosol, and tyrosol, which have been extensively studied for their notable antioxidant, antimicrobial, and anti-inflammatory properties^16^.

Recently, olive leaf extract has been proposed as a corrosion inhibition agent. Refait et al.^17^ studied the inhibiting action of olive leaf extract on corrosion of copper in a NaCl solution, observing an inhibition efficiency of up to 90%. They suggested that oleuropein was the major compound of the leaf extract and thus more likely the main inhibiting species. However, the authors did not provide sufficient details on the mechanism of inhibition and used a single acid molarity. Elabbasy^18^ studied the inhibiting action of olive leaf extract on corrosion of C-steel in Sulfamic acid solution, observing an inhibition efficiency of up to 90%. Their observations using Scanning Electron Microscopy (SEM) suggested that the inhibition mechanism involves physisorption. These studies imply that olive leaf extract could potentially serve as a sustainable corrosion inhibitor. Nevertheless, further research is required to fully comprehend the mechanism of action of olive leaf extract and optimize its use as a corrosion inhibitor.

The process of inhibiting corrosion by olive extract entails the interplay between the molecules of the inhibitor and the surface of the metal^19^. The inhibitor molecules undergo adsorption onto the metal surface, leading to the creation of a protective layer that serves to impede the reaction of the metal with the corrosive environment^20^. The efficacy of the inhibitor is predicated on various factors, including the concentration of the inhibitor, temperature, the nature of the metal and PH. The effectiveness of OLE as a corrosion inhibitor is significantly influenced by the pH of the solution in which it is employed^21^. At specific pH values, the OLE may exhibit greater potential in generating a protective film on the metal surface. many studies revealed that when decreasing the pH from 8 to 3.9, different changes were observed in the corrosion potential after adding the inhibitor and that the efficiency of the inhibitor decreases with increasing acidity, and also the weight loss decreases with increasing the pH from 3.15 to 8.0^22,23^ It was noticed in the practical results that as the acidity of the solution increased and the pH decreased, the effectiveness of the extract decreased. Therefore, comprehending the correlation between pH and its impact on the efficacy of OLE as a corrosion inhibitor is of utmost importance in optimizing its usage.

While it is evident that olive leaf extract is an environmentally-friendly and cheap extraction solution and can potentially offer a sustainable solution for corrosion management, the discussion above reveals gaps in the data available in literature on the mechanism of the corrosion inhibition. In addition, there is no detailed information regarding the water heating extraction process. In this article, the use of olive leaf extract as a corrosion inhibition agent for carbon steel in acidic media is experimentally investigated. The efficacy of an extract obtained from waste olive leaf is evaluated as a potent inhibitor for X70 carbon steel alloy submerged in different concentrations of hydrochloric acid and at different temperatures. Measurements of metal loss, along with Scanning Electron Microscopy and Energy-Dispersive X-ray spectroscopy are used to investigate the mechanism of corrosion inhibition.

## Materials and methods

### Preparation of samples

Samples of X70 Carbon Steel (CS)with dimensions of 2 × 1 × 0.02 cm (± 1%), were prepared. The samples experienced a thorough cleaning process and were subsequently polished with emery paper until their surface exhibited a glossy and smooth appearance. Following this, the samples were strictly rinsed with distilled water before being subjected to treatment with acetone.

### Olive leaf extraction

**Figure 1:**
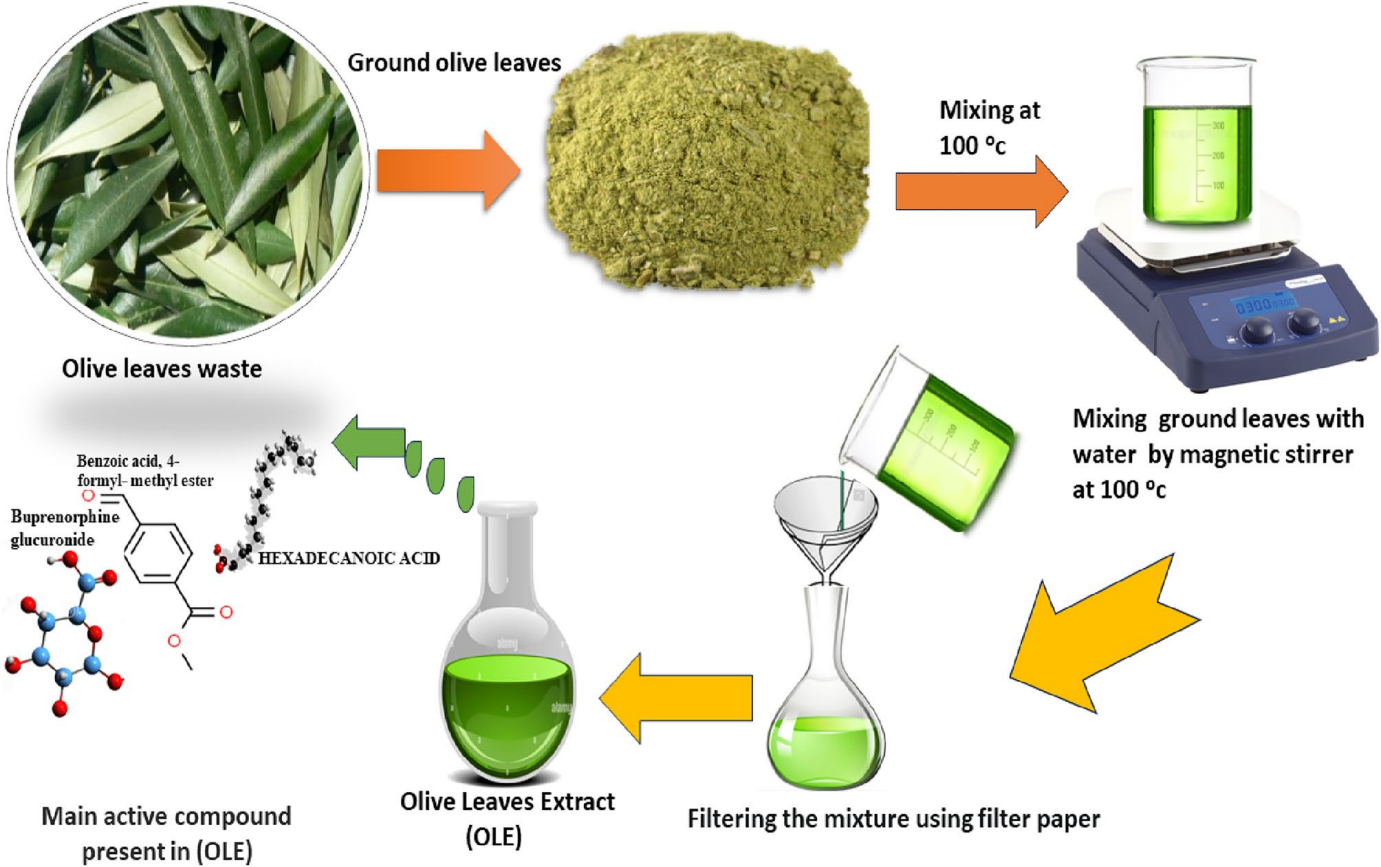
The water extract process of olive leaves waste, which is a rich source of bioactive compounds, making it a valuable resource for the production of green corrosion inhibitors for a variety of metals and alloys.

Olive leaves collected as wastes from the factory presented the olive oil in Egypt and Turkey. Olive leaf extract (OLE) was produced through the incorporation of 10 g of pulverized olive leaves with 100 ml of distilled water. Two sources for olive leaves were used, originating from Egypt and Turkey. Extraction was carried on at 100 °C during 60 min with a 500 rpm mixing speed^24^. Subsequently, the extraction solution was filtered to remove the grounded olive leaves as shown in Fig. 1. It used for at different HCL solution, with varying concentrations and soaking durations at different temperature for each metal sample.

### Methods

#### GC–MS analysis

The chemical composition of the samples was performed using Trace GC1310-ISQ mass spectrometer (Thermo Scientific, Austin, TX, USA) with a direct capillary column TG–5MS (30 m × 0.25 mm × 0.25 µm film thickness). The column oven temperature was initially held at 50 °C and then increased by 5 °C/min to 230 °C hold for 2 min. Increased to the final temperature 290 °C by 30 °C/min and hold for 2 min. The injector and MS transfer line temperatures were kept at 250, 260 °C respectively; Helium was used as a carrier gas at a constant flow rate of 1 ml/min. The solvent delay was 3 min and diluted samples of 1 µl were injected automatically using Autosampler AS1300 coupled with GC in the split mode. EI mass spectra were collected at 70 eV ionization voltages over the range of m/z 40–1000 in full scan mode. The ion source temperature was set at 200 °C. The components were identified by comparison of their retention times and mass spectra with those of WILEY 09 and NIST 11 mass spectral database.

#### Weight loss test

Samples of carbon steel were employed to measure weight loss. The samples were first smoothed with emery paper, then weighed, and subsequently exposed to varying concentrations of hydrochloric acid (0.1, 1.0, and 2.0 M) in the absence and presence of a range of concentrations of olive leaf extract for eight hours at ambient temperature. The carbon steel samples were subsequently extracted, washed with water, cleaned with acetone, dried, and then strictly weighed on a lab scale with an accuracy of 0.01 g. The inhibitory effectiveness percentage (IE%) was determined according to Eq. (1).

1 $$IE\left(\%\right)=\left(1-\frac{{W}_{2}}{{W}_{1}}\right)\times 100$$

where W_1_ and W_2_ express weight loss of the samples before and after submerging the treated metal samples in the corrosive medium.

And the corrosion rate *CR* was determined from Eq. (2):

2 $$CR= \frac{{\mathrm{m}}_{\mathrm{f}}-{\mathrm{m}}_{\mathrm{i}}}{At}$$

where ${m}_{i}$ and ${m}_{f}$ are the initial and final masses, $A$ is the total surface area, and $t$ is the total immersion time. The inhibitory effectiveness percentage (IE%) was determined according to Eq. (3):

3 $$\eta =\frac{CR-C{R}_{ref} }{CR\_ref} \times 100\%$$

where $C{R}_{ref}$ represents the reference corrosion rate of a carbon steel sample at 60 °C in a 2.0 M acidic solution with no OLE treatment.

#### Surface characterization

The utilization of the scanning electron microscopy (SEM) technique allowed for the demonstration of OLE inhibitor adsorption by surface characterization of carbon steel with and without the corrosion inhibiting treatment. Surface morphology investigations were conducted on CS immersed in 0.5 M HCl solution for 3 h before and after the addition of inhibitor, using an analytical scanning electron microscope (Quanta FEG 250) at the EDRC, DRC, Cairo.

#### Response surface method

The response surface methodology refers to a collection of statistical and mathematical techniques employed in the modelling and analysis of problems where the response of interest is contingent on diverse variables^25^. The statistical technique known as analysis of variance (ANOVA) can be used. This technique is based on variance ratios to significant differences exist among the means of several groups of observation. ANOVA analysis involves the consideration of inputs, which belong to the independent variable or the factor that is being tested. In this research, the independent variables consist of the inhibitor type, inhibitor concentration, concentration of corrosive media and the temperature at which the inhibitor is subjected to testing. The response, which is the dependent variable, is the corrosion rate that is being measured.

Two-way ANOVA has been used to determine the effect of temperature and inhibitor concentration on corrosion rate at different corrosive solution, (0.1, 1.0 and 2 M) HCL. The analysis determines whether variations in temperature affects and inhibitor concentration result in significant differences in inhibitor effectiveness.

The null hypothesis is rejected if the computed F values (F) are too large^26^.

### Ethical approval

The collection and handling of plants leaves were in accordance with all the relevant guidelines.

## Results and discussion

Several parameters, such as temperature, concentration of corrosion inhibitors, composition of the corrosive medium, surface features, and environmental conditions, have an effect on the rate of corrosion. By controlling and changing these input factors, it is possible to change the rate of corrosion and possibly slow it down. In this section, the effect of selected parameters on the effectiveness of oil leaf extract as a corrosion inhibition agent is analysed.

### GC–MS analysis

The results obtained from gas chromatography-mass spectrometry (GC–MS), as presented in Table 1, exhibit the presence of various chemical compounds containing functional groups comprising heteroatoms such as oxygen, and nitrogen. These heteroatoms can form a protective layer on the metal surface by physical adsorption, thereby shielding it from corrosive media. The inhibitory solution of OLE, an aqueous solution that can dissolve in the corrosive medium of HCl very easily. Since water is used as an extraction solvent to extract the plant's active components, the water in this inhibitory solution has no negative effects on metal corrosion^27^.

**Table 1:** Main compounds obtained from GC–MS analysis of an OLE extract.

Name	Formula	2D structure	Compound percentage
Prostaglandin F2à-biotinamide	C_35_H_60_N_4_O_6_S		6.54%
Benzoic acid, 4-formyl-methyl ester	C_9_H_8_O_3_		43.78%
n-Hexadecanoic acid	C_16_H_32_O_2_		70.16%
Hexadecanoic acid	C_19_H_38_O_4_		32.38%
1-Ethyl-6-methyl-5,6,6a,7-tetrahydro-4H-dibenzo[de,g]quinolin-2-ol	C_17_H_17_NO_2_		44.30%
Buprenorphine glucuronide	C_35_H_49_NO_10_		18.73%

### Weight loss

Weight loss and corrosion rates of carbon steel samples treated with OLE solutions obtained from samples originating in Egypt and Turkey for 8 h, under the effect of considering different temperatures and HCl molarities. The inhibition efficiencies values were calculated and compared for the OLE inhibitor, at various concentrations for 8 h of immersion at different temperature, and the obtained results are summarized in Table 2.

**Table 2:** Weight loss and corrosion rate changes according to different experimental parameters.

Origin of olive leaf	Molarity (mol/l)	Temperature (°C)	OLE concentration (ppm)	Immersion time (h)	Wight loss (mg)	Corrosion rate $\frac{dm}{dt}$ (g m^−2^/day)	IE (%)
Egypt	2	60	30	8	0.0068	39.23	22.73
Egypt	2	30	60	8	0.0048	27.69	39.25
Egypt	0.1	60	0	8	0.0067	38.65	–
Egypt	2	60	0	8	0.0088	50.77	–
Turkey	0.1	60	60	8	0.0047	27.12	29.83
Turkey	2	30	0	8	0.0079	45.58	–
Turkey	2	30	0	8	0.0077	44.42	–
Turkey	1	30	60	8	0.0058	33.46	26.60
Turkey	2	45	60	8	0.0064	36.92	19.00
Egypt	0.1	30	30	8	0.0046	26.54	6.12
Egypt	2	60	60	8	0.0059	34.04	32.96
Turkey	0.1	60	60	8	0.0048	27.69	28.3
Turkey	0.1	45	0	8	0.0052	30.00	–
Egypt	0.1	30	0	8	0.0049	28.27	–
Egypt	1	45	30	8	0.0068	39.23	4.22
Turkey	1	45	30	8	0.0069	39.81	2.81
Egypt	0.1	30	60	8	0.0041	23.65	16.3
Turkey	1	60	0	8	0.0071	40.96	–

The data in Table 2 indicates that the presence of the inhibitor leads to lower weight loss compared to nontreated samples, indicating that the inhibitor provides protection to the metal surface against the damaging effects of acidic corrosion and the inhabitation efficiency of this inhibitor increase by increasing the concentration of OLE which mean more protection level for the metal in the presence of OLE inhibitor. Data presented in Table 2 also indicate that with increasing the temperature and molarity of corrosive medium the efficiency of the OLE as inhibitor is decreased which means that this inhibitor is not suitable for application in industries that deal with metals at high temperatures. The observed reduction in corrosion rate with increasing inhibitor concentration can be attributed to the formation of an insulating adsorption layer at the metal-solution interface, enhancing the efficiency of the inhibitor^28^. This effect is particularly noticeable at a concentration of 60 ppm, where the inhibitor demonstrates superior effectiveness compared to a lower concentration.

### Surface characterization

**Figure 2:**
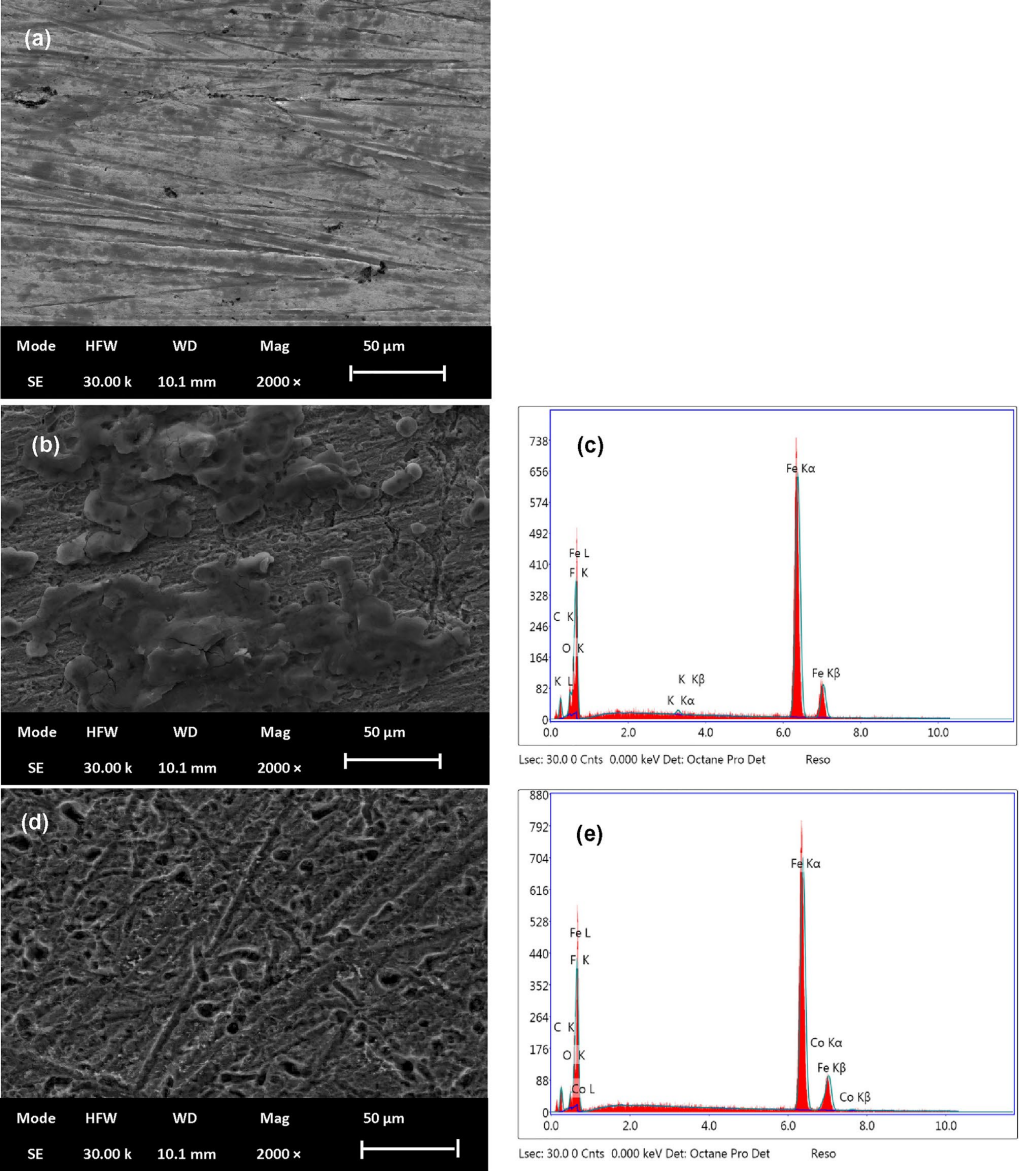
SEM and EDX images for samples of carbon steel (**a**) sample freshly polished, (**b**, **c**) sample immersed in 2 M HCl, and (**d**, **e**) specimen exposed to 2 M HCl and 60 ppm of inhibitor.

Scanning Electron Microscopy (SEM) images provide important insights into the surface characteristics and microstructure of materials, allowing to characterize and evaluate their properties at the micro- or nanoscale. In this section, Fig. 2 presents (SEM) images of the carbon Steel (CS) surfaces after exposure to a 2 M Hydrochloric Acid (HCl) solution containing 60 ppm of corrosion inhibitor (OLE) for 8 h. Specifically, Fig. 2a shows the SEM image of the freshly polished CS surface. Figure 2b displays the magnified SEM image of the carbon steel sample immersed in 2 M HCl, revealing the destruction of the steel surface.

The severe corrosion is evident through the presence of large and deep vacuoles on the steel surface. However, the SEM image in Fig. 2d demonstrates that the carbon steel surface treated with 60 ppm OLE exhibits a relatively smoother morphology, similar to a newly sanded surface. These observations indicate that the OLE inhibitor molecules have adhered to the CS surface and forming a protective film and shielding it from corrosion. This film was formed by physical adsorption of oxygen and nitrogen atoms which exist in the OLE extract as detailed by GC–MS analysis. The weak bond of oxygen and nitrogen molecules facilitates their interaction with metal surface. As the metal surface is exposed to oxygen or nitrogen gas, the gas molecules get in contact with the metal atoms and physically adsorbed on the metal surface. The physical adsorption of these gases can have significant effects on the properties of the metal surface. For instance, oxygen adsorption can result in the development of a layer of metal oxide, which may protect against further oxidation. This protective oxide layer can prove beneficial in preventing rust or corrosion of the metal.

Additionally, in order to recognize the elemental composition of the metal surface samples before and after implementation of the studied inhibitor, Energy-Dispersive X-ray spectroscopy (EDX) was carried out and examined. It is evident that the surface of metal which immersed in uninhibited solution composed of a significant proportion of atoms consisting of iron, oxygen, and carbon as shown in Fig. 2c. Upon comparison of the percentage displayed in the blank spectrum with that of the specimen in the existence of OLE, a noteworthy reduction in O peaks is discernible as depicted in Fig. 2e, which prove that there is a decrease in the density of corrosion active area^29^. These observations indicate a reduction in the corrosion of metal after the addition OLE inhibitor. This surface characterization is consistent with the results obtained by gravimetric measurements and statistical calculations.

### Response surface methodology (RSM)

(RSM) is a statistical technique utilized for the purpose of enhancing and optimizing processes through the examination of the correlation between input variables and response variables. The identification of optimal input variable settings is crucial for achieving the desired response and providing effective and productive process optimization.

An analysis of variance (ANOVA) was performed to investigate the influence of OLE concentration on corrosion inhibition. The study utilized a Taguchi Orthogonal Array (OA) design, which involved the integration of three levels of temperature, three levels of OLE concentration, three levels of corrosive media molarity, and two distinct sources of olive leaves. Table 3 contains the empirical results regarding the rate of corrosion.

**Table 3:** Empirical results regarding the rate of corrosion depend on the input variables.

ID	OLE source	Temperature	OLE concentration	Molarity	Corrosion rate	Inhibition efficiency
#	#	$T$ (^o^C)	${C}_{extract}$ (ppm)	$M$ (mol l^−1^)	$\frac{dm}{dt}$ (g m^−2^ day^−1^)	η (%)
1	2	60	30	2.0	39.2	22.7
2	2	30	60	2.0	27.7	45.5
3	2	60	0	0.1	38.7	23.9
4	2	60	0	2.0	50.8	0.0
5	1	60	60	0.1	27.1	46.6
6	1	30	0	2.0	45.6	10.2
7	1	30	0	2.0	44.4	12.5
8	1	30	60	1.0	33.5	34.1
9	1	45	60	2.0	36.9	27.3
10	2	30	30	0.1	26.5	47.7
11	2	60	60	2.0	34.0	33.0
12	1	60	60	0.1	27.7	45.5
13	1	45	0	0.1	30.0	40.9
14	2	30	0	0.1	28.3	44.3
15	2	45	30	1.0	39.2	22.7
16	1	45	30	1.0	39.8	21.6
17	2	30	60	0.1	23.7	53.4
18	1	60	0	1.0	41.0	19.3

A screening test was carried out and the origin of OLE did not have any significant effect on corrosion inhibition. Subsequently, the effect of inhibitor concentration, temperature and time as independent variables along with their interactions up to the third degree on the inhibition efficiency as a response variable was screened via a full factorial model.

The results of the screening model illustrated a strongly significant effect of OLE concentration and the molarity of the solution. The temperature effect on the inhibition efficiency was marginally significant. The interaction between OLE concentration and the Molarity was the only second or third order effect to have an effect on the inhibition efficiency. The detailed parameters of the screening model are shown in Table 4.

**Table 4:** ANOVA parameters for a full factorial screening model.

Variable	Degrees of freedom	Sum of squares	F ratio	Null hypothesis probability (p)
Model	7	3399.6	9.89	**Significant 0.09%**
A: OLE concentration (ppm)	1	1172.4	23.87	** > 0.01%**
B: temperature (°C)	1	257.3	5.24	4.51%
C: molarity (mol l^−1^)	1	1484.7	30.23	**0.03%**
AB	1	59.8	1.22	*29.5%*
BC	1	12.7	0.26	*62.2%*
AC	1	195.8	3.99	7.38%
ABC	1	30.18	0.61	*45.1%*
Lack of fit	10	491.1		
Correlation, total	17	3890.7		
R^2^	0.8737			
Adjusted R^2^	0.7854			
RMS error	7.01			

Significant values are in bold, italics and underlined.

To analyze the effect of the significant parameters on the corrosion inhibition efficiency, an Analysis of Variance was performed considering only the first order parameters along with the interaction between Molarity and OLE concentration. The parameters of this due to the reduction of the noise error by disregarding the contribution of insignificant variables, the F ratios are improved compared to the full factorial model.

The second-order model predicts that the corrosion inhibition efficiency is related to the independent effects by the form in Eq. (4), with high confidence, exhibiting a root mean square error of 3.4121, R2 value of 0.85, and a P value of less than 0.0001.

4 $$\eta \left[\%\right]=45.02-0.273 T-0.318 {C}_{extract}-11.26 M+0.138\left(M-1.0388\right)\left({C}_{extract}-30\right)$$

**Figure 3:**
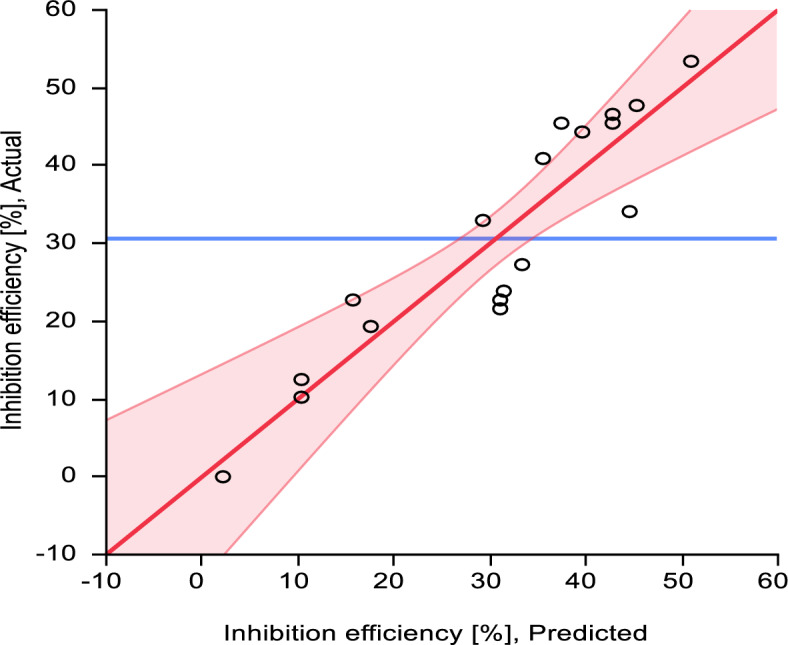
ANOVA model prediction of inhibition efficiency versus experimental data. Analysis of Variance with first order parameters and the interaction between Molarity and OLE concentration. RMSE = 6.7208, R^2^ = 0.85, P value ≤ 0.0001. Data mean = 30.6%, standard deviation = 15.1%.

The model predictions agree well with the measurements as shown in Fig. 3 and Table 5. The inhibition effect of the olive leaves extract concentration is significant at different corrosive media molarity. Notably, there was no significant difference between the OLE of different origin used in the experiment.

**Table 5:** Significant-parameter ANOVA model results for factors affecting the response variable with selected interactions.

Variable	Degrees of freedom	Sum of squares	F ratio	Null hypothesis probability (p)
Model	4	3303.5	18.28	
Temperature (°C)	1	234.0	5.18	4.04%
OLE concentration (ppm)	1	1280.0	28.33	0.01%
Molarity (mol l^−1^)	1	1602.6	35.48	< 0.01%
OLE conc. interaction with molarity	1	185.9	4.117	6.34%
Lack of fit	13	587.2		
Correlation, total	17	3890.7		
R^2^	0.849			
Adjusted R^2^	0.802			
RMS error	6.72			

**Figure 4:**
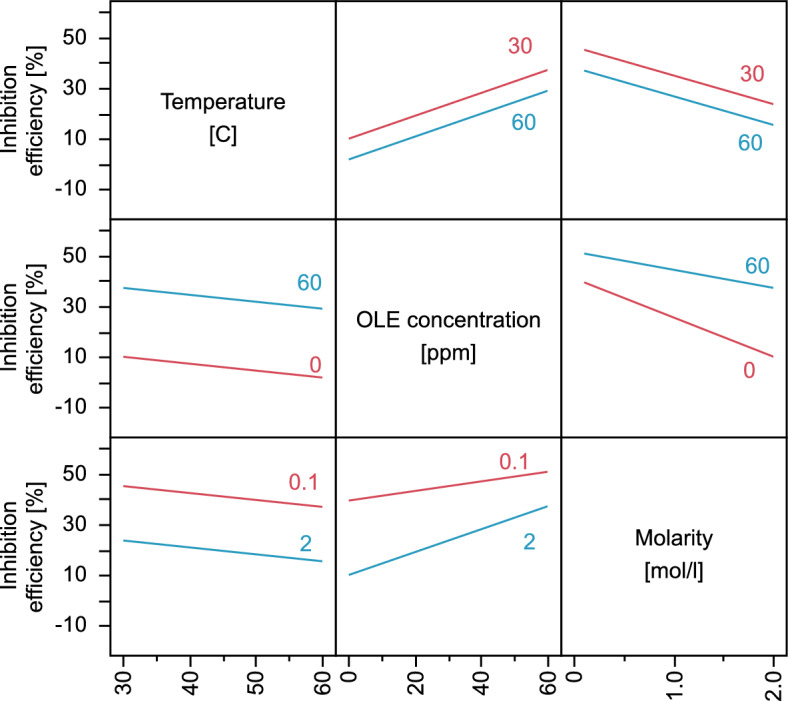
Interactions plots for the effects of temperature, solution molarity, and OLE concentration on the Inhibition efficiency based on the prediction expression of the partial second-order model. *Upper* effect of acidic medium molarity on inhibition efficiency at different OLE concentrations. *Lower* effect of OLE concentration on inhibition efficiency at different acidic medium molarities. Red and blue lines represent the minimum and maximum values within the data range.

Interaction plots of the three parameters are presented in Fig. 4. Although the temperature, as expected, has a significant effect on the inhibition efficiency, the parallel lines in interaction plots that include temperature assert the fact that the interaction between temperature and the two other parameters does not yield significant effects on the inhibition efficiency. This suggests that the temperature is not expected to hinder the use of the OLE extract for corrosion inhibition.

The increase in OLE concentration leads to a significant increase in inhibition efficiency at all temperatures and all solution molarity values. On the other hand, the interaction between molarity and OLE concentration results in a higher inhibition effect at higher solution molarities, resulting in enhanced protection when required.

**Figure 5:**
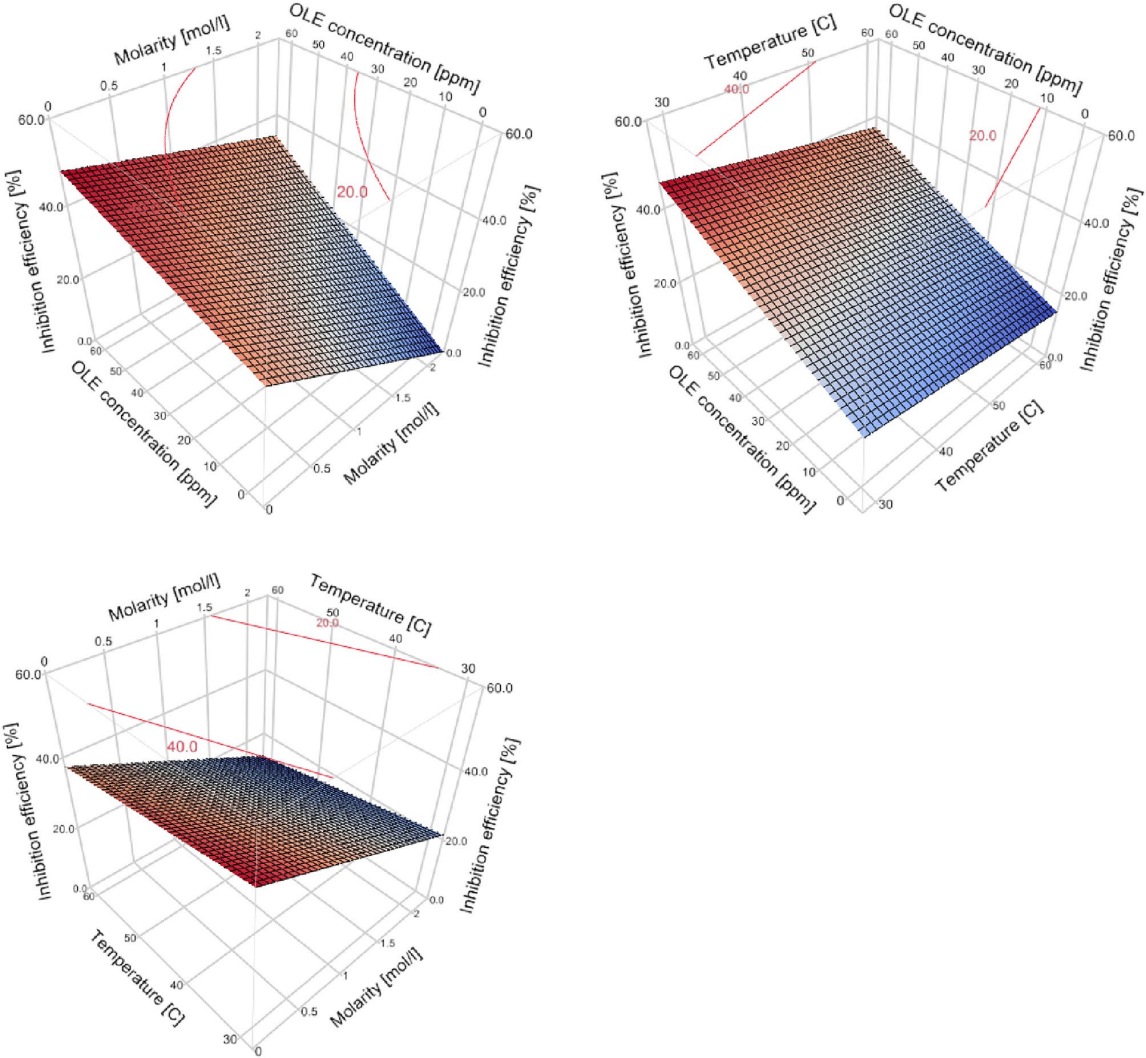
Response surface plots for the effects of temperature, OLE concentration, and acid molarity on the inhibition efficiency.

Figure 5 demonstrates the response surfaces of inhibition efficiency to the interacting effects of the three considered parameters. The behaviour in the response surfaces in the figure assert the predictions of the model. The contour plots indicate inhibition efficiency values. The interaction of OLE concentration and acid molarity is observed, where the increased molarity would positively enhance the efficiency of the OLE. Increased temperature result in lower inhibition efficiency without an interaction with the OLE concentration, such that the reduction is the same at different OLE concentrations.

From the previous discussion, results indicate that the corrosion inhibition activity of OLE may be due to the presence of various organic compounds in its composition such as phenolic compounds, tannins, flavonoids and oleuropein. These organic compounds contain polar function groups with hetero atom such as O in addition to conjugated double bonds or aromatic rings, which are the major adsorption centers.

The inhibitory process involves the absorption of compounds by the metallic exterior, resulting in the formation of a protective layer that shields the alloy from corrosion^30^. The corrosion medium generates hydrogen ions on the outer layer of metal, which combine with other ions to form hydrogen gas. However, inhibitory compounds can generate neutral molecules that attach to the metal surface, forming a protective layer instead of hydrogen ions, thereby inhibiting corrosion^31^. Consequently, it may be suggested that the protective films may be formed on the metal surface through the adsorption process to inhibit the corrosion of CS in HCl medium.

## Conclusion

In this study, the use of an environmentally friendly extract from olive leaf as a corrosion inhibition agent is considered to address economic and environmental challenges while also providing additional revenue opportunities through the utilization of an underappreciated material. A process to produce an aqueous extract from oil leave waste is proposed. The effect of treating carbon steel surfaces with the olive oil extract is analyzed through metal-loss measurements, SEM, chromatography-mass spectrometry, and EDX to quantify corrosion inhibition efficiency and give an insight on its mechanism. Results showed that olive leaf extract exhibits corrosion inhibition properties on carbon steel surfaces, with an efficiency of 53% at an inhibitor concentration of 60 ppm compared with the reference case. Statistical analysis confirms that corrosion rate is affected by temperature, inhibitor concentration, and their synergistic impact. SEM analysis further reveal that the olive leaf extract acts as an inhibitor through adsorption, effectively blocking the active sites on the carbon-steel surface. This inhibitory process is facilitated by the interaction between the organic component in the olive leaf extract and the metal surface. Olive leaf extract offers promising potential as a green corrosion inhibitor in various applications, such as oil and gas, construction, and transportation. By promoting the use of olive leaf extract as a green corrosion inhibitor, we can reduce the dependence on toxic chemicals and contribute to a more sustainable future, while reducing the environmental footprint of heavy industries. Given that it is a natural and biodegradable product, it could also contribute to cost-efficiency as it represents a cost-effective alternative to traditional corrosion inhibitors (Supplementary Figure 1).

### Supplementary Information

Supplementary Figures.

Supplementary Information 2.

## Data Availability

All data generated or analysed during this study are included in this published article.
