# Supplementary material for: Investigation of olive leaf extract as a potential environmentally-friendly corrosion inhibitor for carbon steel
Source: Sci Rep. 2023 Oct 10;13:17151. doi: 10.1038/s41598-023-43701-x (PMC10564776; doi:10.1038/s41598-023-43701-x)
Supplement: Supplementary file 1 — Supplementary Figures. [file 41598_2023_43701_MOESM1_ESM.docx]

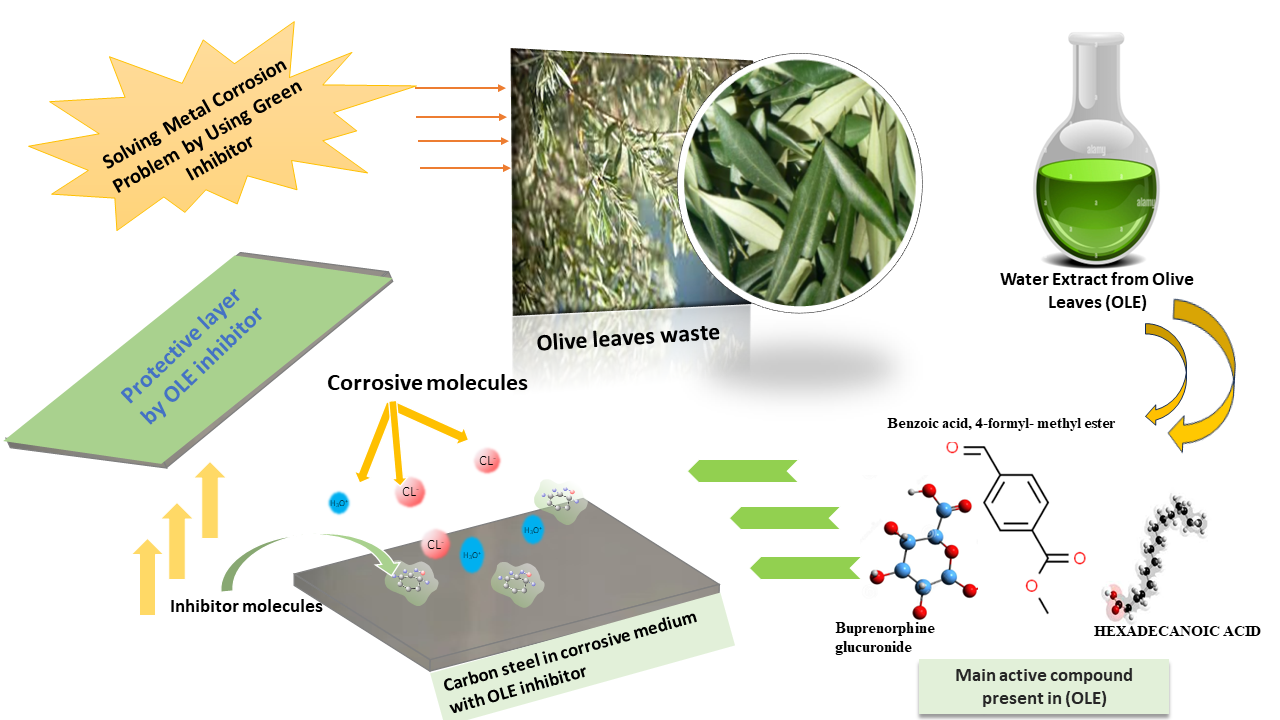


Supplementary Figure1. Experimental setup showcasing the use of water extracted from olive leaves waste as a novel green corrosion inhibitor. The study explores the potential of this eco-friendly alternative in effectively inhibiting corrosion and protecting metal surfaces.
