# Supplementary material for: Investigation of olive leaf extract as a potential environmentally-friendly corrosion inhibitor for carbon steel
Source: Sci Rep. 2023 Oct 10;13:17151. doi: 10.1038/s41598-023-43701-x (PMC10564776; doi:10.1038/s41598-023-43701-x)
Supplement: Supplementary file 2 — Supplementary Information 2. [file 41598_2023_43701_MOESM2_ESM.pdf]

Library Search Report

Data File:	shrefa100	Original Data Path:	D:\work\DR.SYED\20-8-2023
Current Data Path:	D:\work\DR.SYED\20-8-2023	Sample Type:	Unknown
Sample ID:	shrefa 100	Sample Name:	
Run Time(min):	87.45		

Instrument Method:	D:\work\DR.SYED\Dr sayed 45.meth
Sample Name:	
Original Processing Method:	
Current Processing Method:	N/A

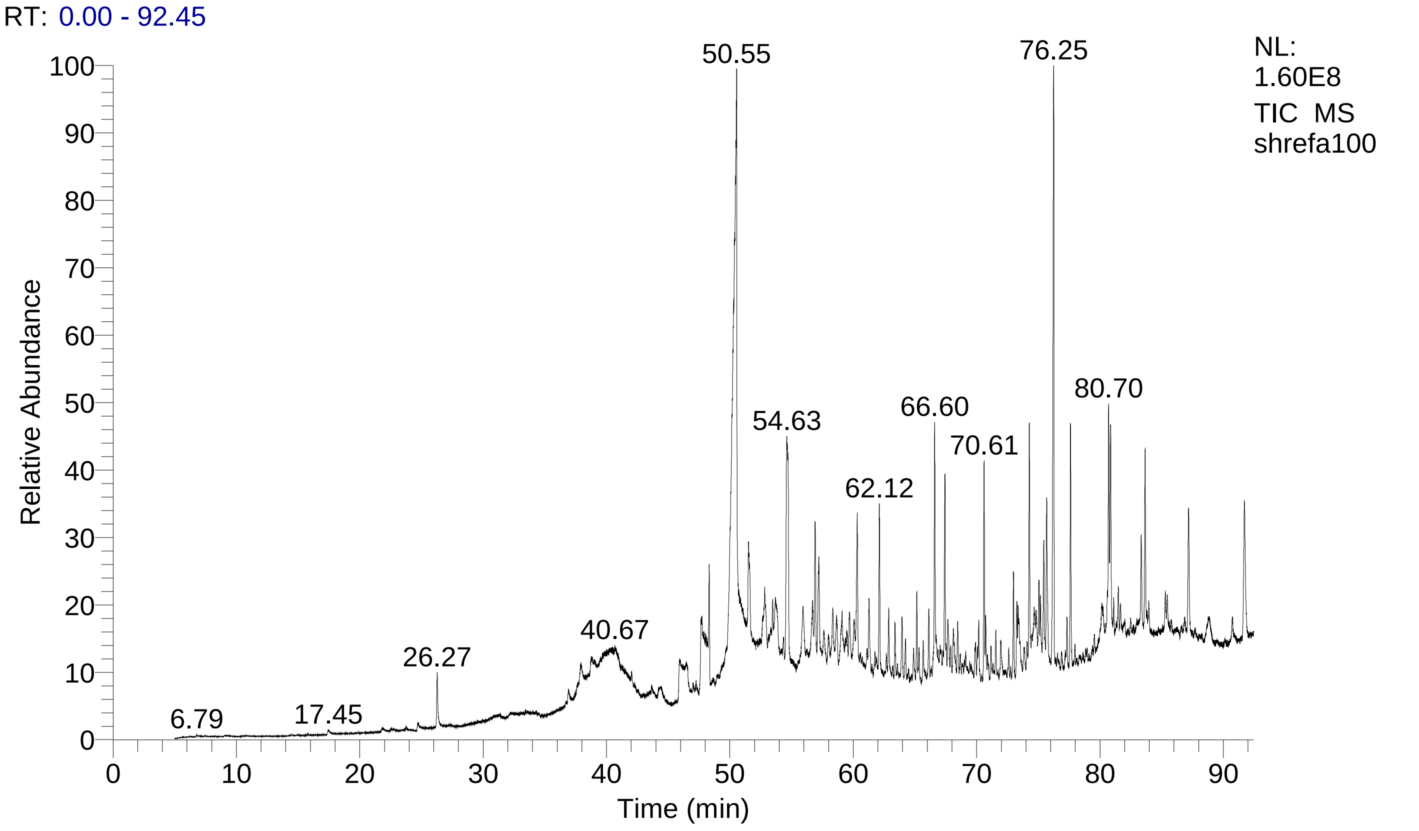

There is no signature data to report.

Library Search Report

shrefa100 #6255 RT: 26.27 AV: 1 NL: 3.51E6
T: + c EI Full ms [50.00-1000.00]

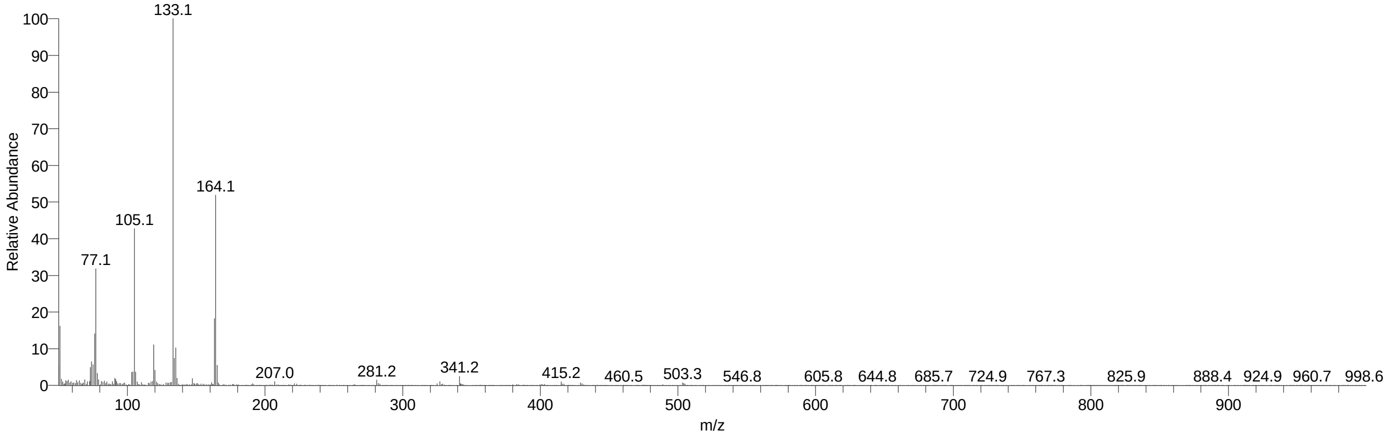

RT	Scan #	Probability	Compound Name	SI	RSI	Cas #	Area	Area %	Library
26.27	6255.000000	43.78	Benzoic acid, 4-formyl-, methyl ester	790	894	1571-08-0	63038745.90	0.77	mainlib
26.27	6255.000000	43.78	BENZOIC ACID, 2-FORMYL-, METHYL ESTER	790	883	4122-56-9	63038745.90	0.77	WileyRegistry8e
26.27	6255.000000	43.78	Benzoic acid, 2-formyl-, methyl ester	790	883	4122-56-9	63038745.90	0.77	mainlib
26.27	6255.000000	43.78	BENZOIC ACID, 4-FORMYL-, METHYL ESTER	757	894	1571-08-0	63038745.90	0.77	WileyRegistry8e
26.27	6255.000000	43.78	Benzoic acid, 4-formyl-, methyl ester	747	892	1571-08-0	63038745.90	0.77	replib

Hit Spectrum | Compound Structure

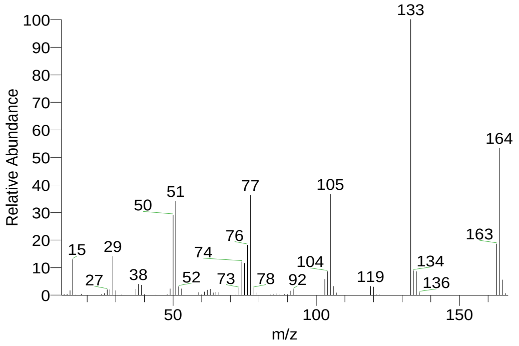

Benzoic acid, 4-formyl-, methyl ester
Formula C9H8O3, MW 164, CAS# 1571-08-0, Entry# 128889
p-Carbomethoxybenzaldehyde

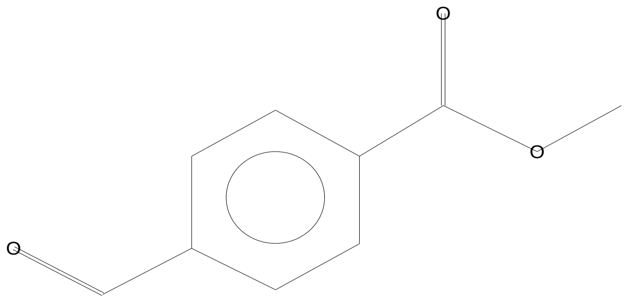

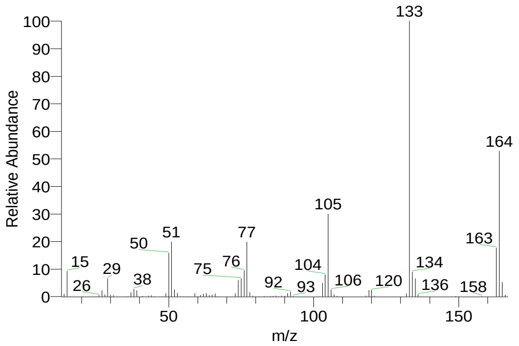

BENZOIC ACID, 2-FORMYL-, METHYL ESTER
Formula C9H8O3, MW 164, CAS# 4122-56-9, Entry# 46652
METHYL 2-FORMYLBENZOATE

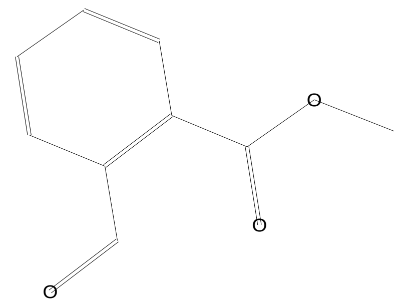

There is no signature data to report.

Library Search Report

Hit Spectrum	Compound Structure

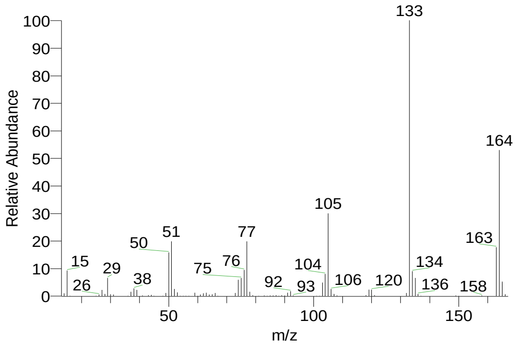

Benzoic acid, 2-formyl-, methyl ester
Formula C9H8O3, MW 164, CAS# 4122-56-9, Entry# 128893
Phthalaldehydic acid, methyl ester

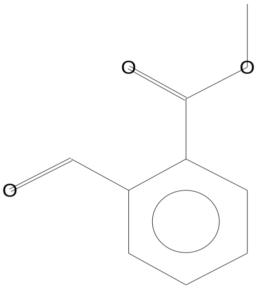

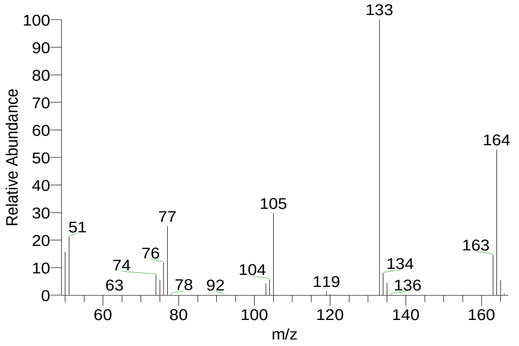

BENZOIC ACID, 4-FORMYL-, METHYL ESTER
Formula C9H8O3, MW 164, CAS# 1571-08-0, Entry# 46655
METHYL 4-FORMYLBENZOATE

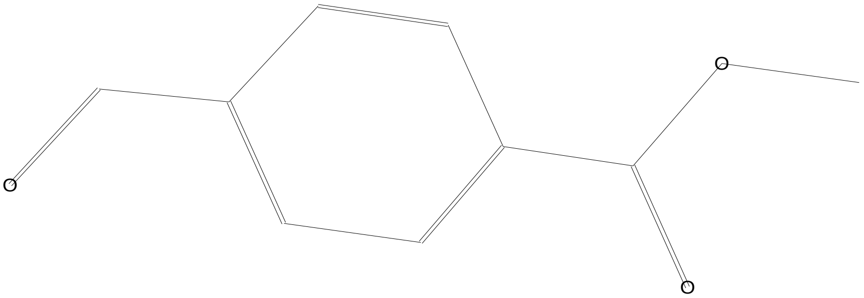

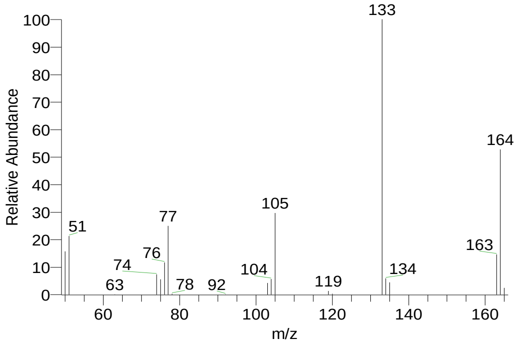

Benzoic acid, 4-formyl-, methyl ester
Formula C9H8O3, MW 164, CAS# 1571-08-0, Entry# 24544
p-Carbomethoxybenzaldehyde

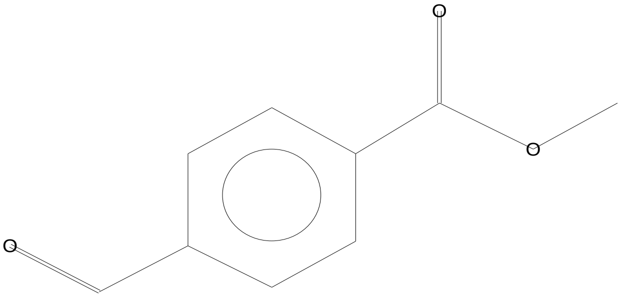

There is no signature data to report.

Library Search Report

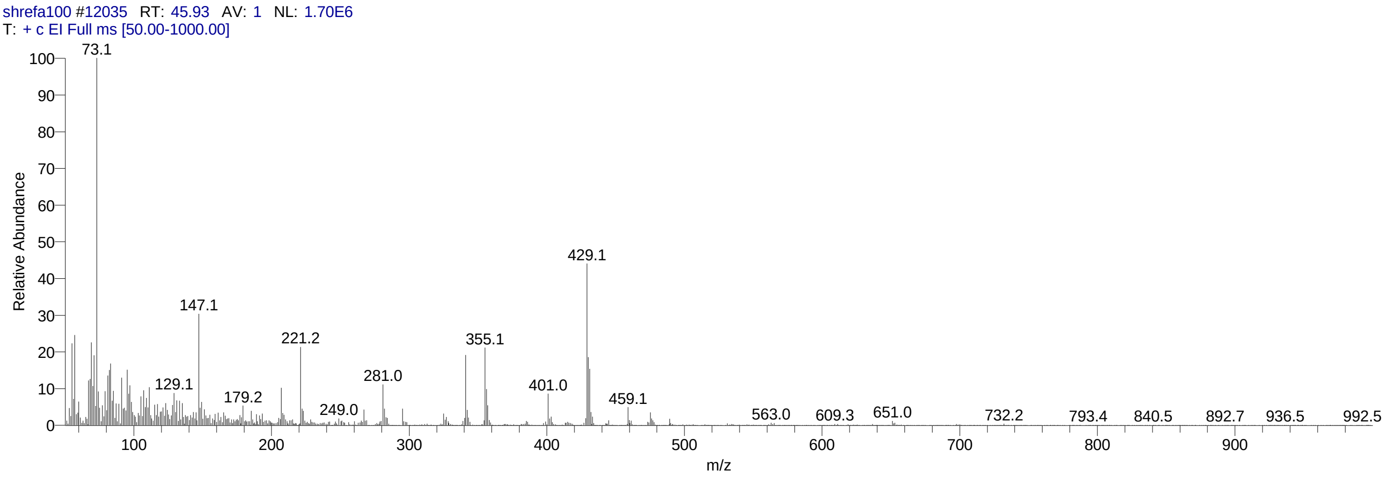

RT	Scan #	Probability	Compound Name	SI	RSI	Cas #	Area	Area %	Library
45.93	12035.000000	45.97	Cyclononasiloxane, octadecamethyl-	694	881	556-71-8	80935119.93	0.99	replib
45.93	12035.000000	45.97	Cyclononasiloxane, octadecamethyl-	673	834	556-71-8	80935119.93	0.99	mainlib
45.93	12035.000000	45.97	CYCLONONASILOXANE, OCTADECAMETHYL-	673	834	556-71-8	80935119.93	0.99	WileyRegistry8e
45.93	12035.000000	45.97	CYCLONONASILOXANE, OCTADECAMETHYL-	673	834	556-71-8	80935119.93	0.99	WileyRegistry8e
45.93	12035.000000	14.02	SILICONE OIL	669	726	NA	80935119.93	0.99	WileyRegistry8e

Hit Spectrum

Compound Structure

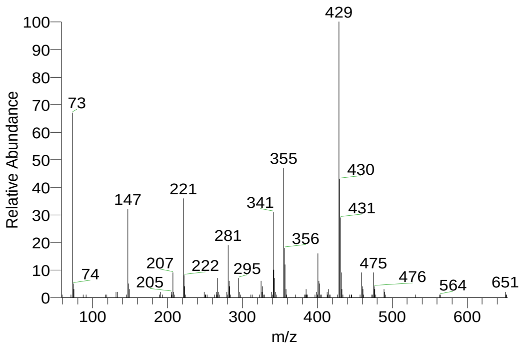

Cyclononasiloxane, octadecamethyl-
Formula C18H54O9Si9, MW 666, CAS# 556-71-8, Entry# 39116
Octadeamethyl-cyclononasiloxane

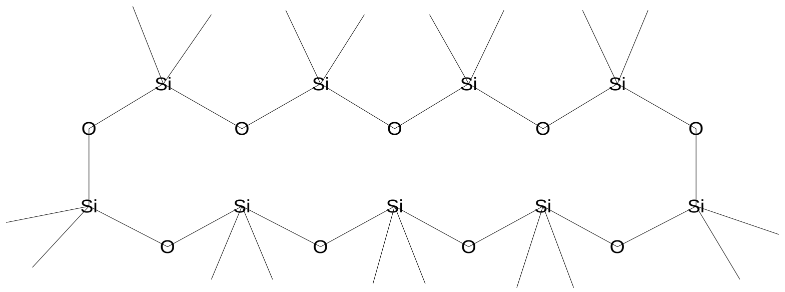

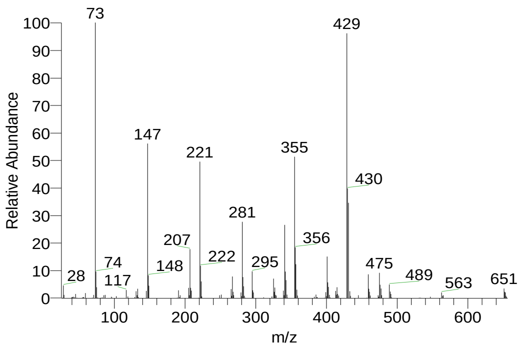

Cyclononasiloxane, octadecamethyl-
Formula C18H54O9Si9, MW 666, CAS# 556-71-8, Entry# 48481
Octadeamethyl-cyclononasiloxane

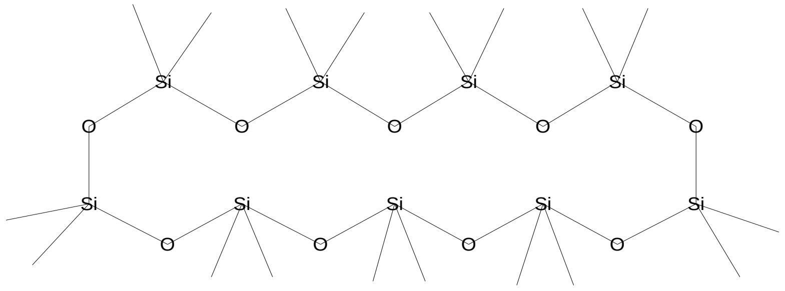

There is no signature data to report.

Library Search Report

Hit Spectrum | Compound Structure

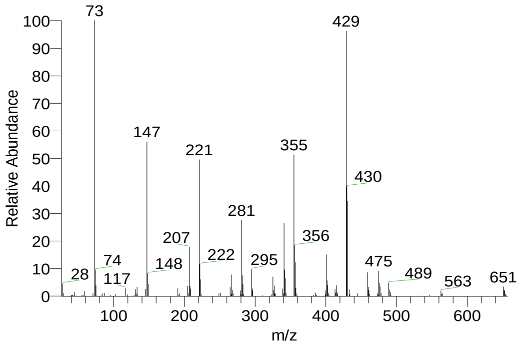

CYCLONONASILOXANE, OCTADECAMETHYL-
Formula C18H54O9Si9, MW 666, CAS# 556-71-8, Entry# 300334
2,2,4,4,6,6,8,8,10,10,12,12,14,14,16,16,18,18-OCTADECAMETHYLCYCLONONASILOXANE #

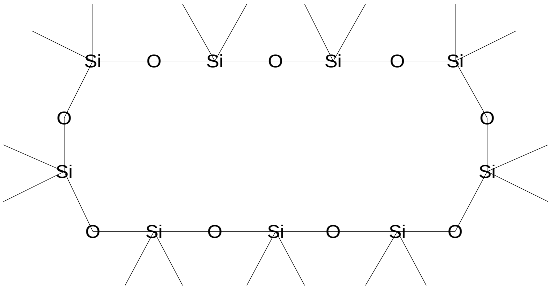

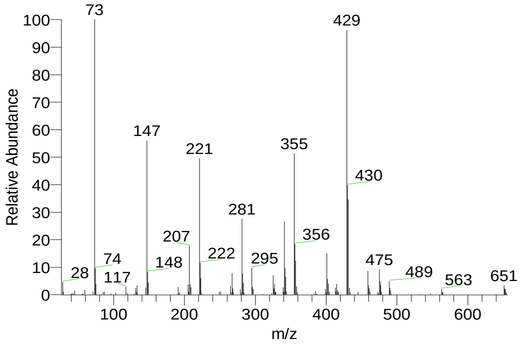

CYCLONONASILOXANE, OCTADECAMETHYL-
Formula C18H54O9Si9, MW 666, CAS# 556-71-8, Entry# 385416
2,2,4,4,6,6,8,8,10,10,12,12,14,14,16,16,18,18-OCTADECAMETHYLCYCLONONASILOXANE #

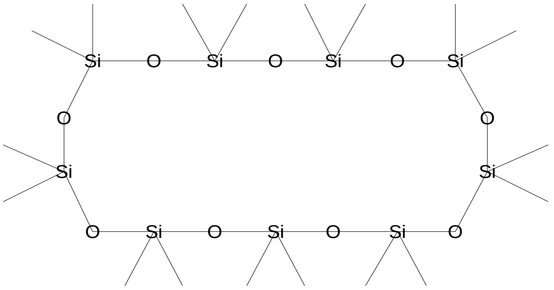

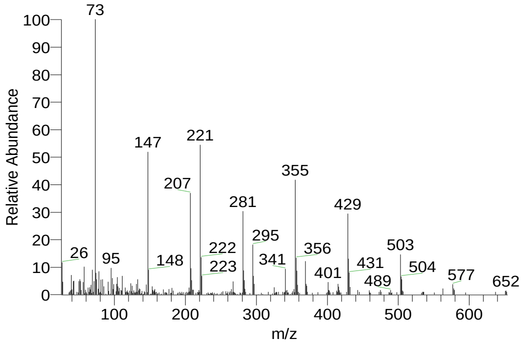

SILICONE OIL
Formula , MW 0, CAS# NA, Entry# 305490
SILIKONFETT SE30 (GREVELS)

There is no signature data to report.

Library Search Report

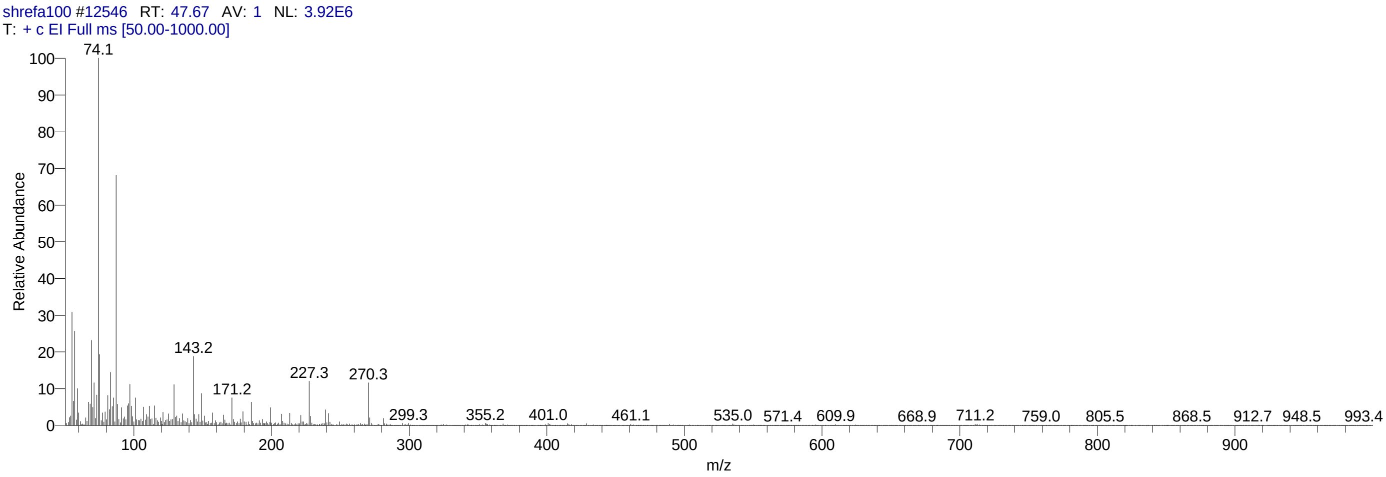

RT	Scan #	Probability	Compound Name	SI	RSI	Cas #	Area	Area %	Library
47.67	12546.000000	57.50	PENTADECANOIC ACID, 14-METHYL-, METHYL ESTER	797	822	5129-60-2	137232598.02	1.68	WileyRegistry8e
47.67	12546.000000	12.68	Hexadecanoic acid, methyl ester	754	889	112-39-0	137232598.02	1.68	replib
47.67	12546.000000	12.68	HEXADECANOIC ACID, METHYL ESTER	747	880	112-39-0	137232598.02	1.68	WileyRegistry8e
47.67	12546.000000	9.46	PENTADECANOIC ACID, 13-METHYL-, METHYL ESTER	746	799	5487-50-3	137232598.02	1.68	WileyRegistry8e
47.67	12546.000000	57.50	PENTADECANOIC ACID, 14-METHYL-, METHYL ESTER	745	812	5129-60-2	137232598.02	1.68	WileyRegistry8e

Hit Spectrum	Compound Structure

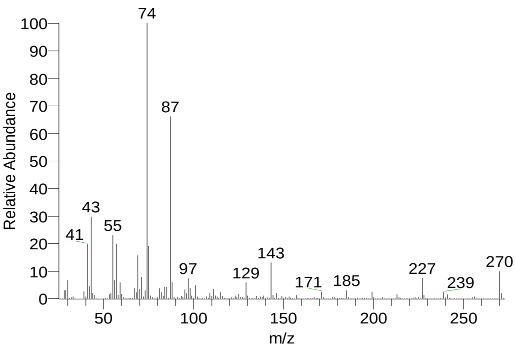

PENTADECANOIC ACID, 14-METHYL-, METHYL ESTER
Formula C17H34O2, MW 270, CAS# 5129-60-2, Entry# 161312
METHYL 14-METHYLPENTADECANOATE

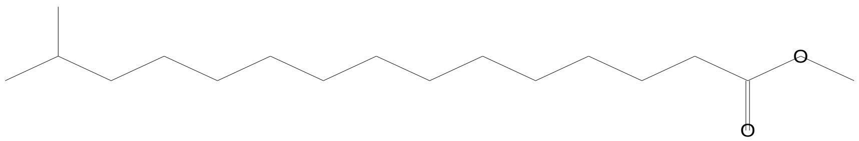

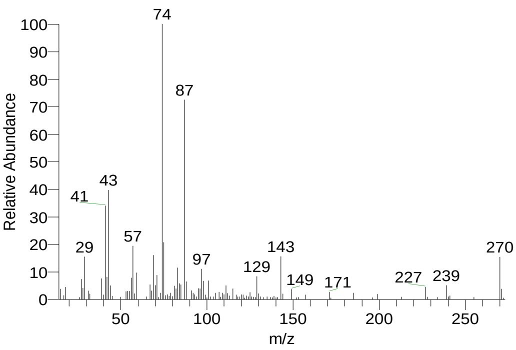

Hexadecanoic acid, methyl ester
Formula C17H34O2, MW 270, CAS# 112-39-0, Entry# 12041
Palmitic acid, methyl ester

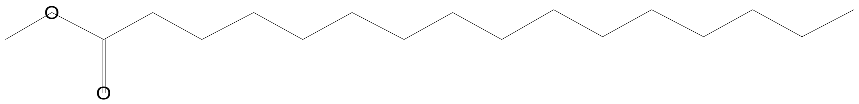

There is no signature data to report.

Library Search Report

Hit Spectrum | Compound Structure

HEXADECANOIC ACID, METHYL ESTER
Formula C17H34O2, MW 270, CAS# 112-39-0, Entry# 161284
METHYL HEXADECANOATE

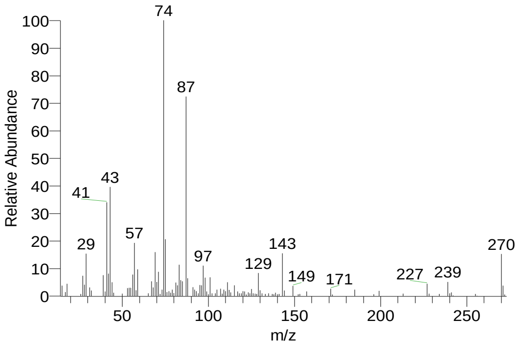

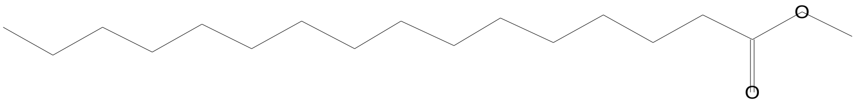

PENTADECANOIC ACID, 13-METHYL-, METHYL ESTER
Formula C17H34O2, MW 270, CAS# 5487-50-3, Entry# 161304
METHYL 13-METHYLPENTADECANOATE

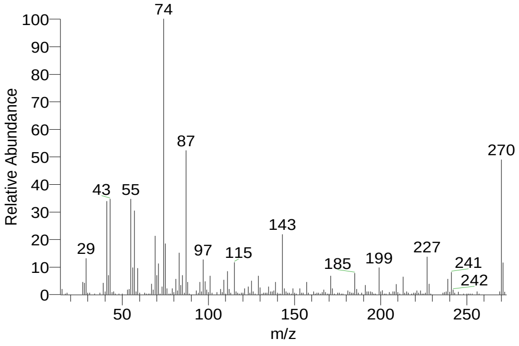

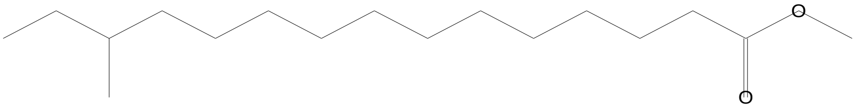

PENTADECANOIC ACID, 14-METHYL-, METHYL ESTER
Formula C17H34O2, MW 270, CAS# 5129-60-2, Entry# 161313
METHYL 14-METHYLPENTADECANOATE

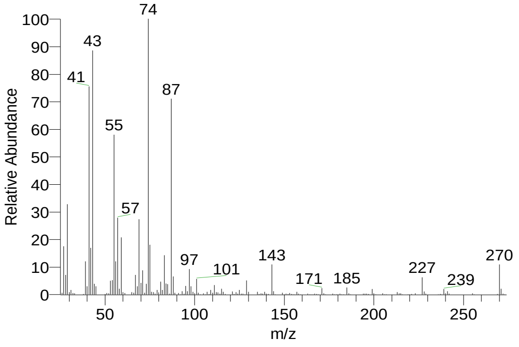

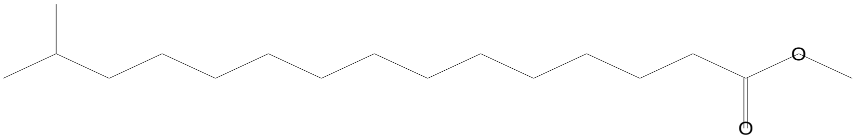

There is no signature data to report.

Library Search Report

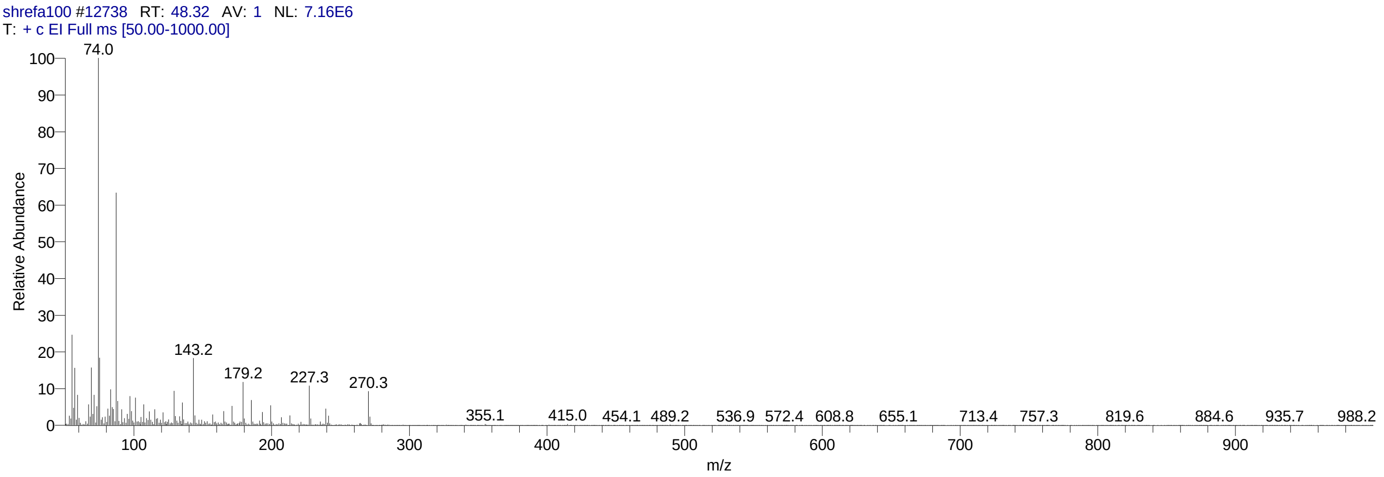

RT	Scan #	Probability	Compound Name	SI	RSI	Cas #	Area	Area %	Library
48.32	12738.000000	64.45	PENTADECANOIC ACID, 14-METHYL-, METHYL ESTER	814	817	5129-60-2	92866791.55	1.14	WileyRegistry8e
48.32	12738.000000	19.31	HEXADECANOIC ACID, METHYL ESTER	788	865	112-39-0	92866791.55	1.14	WileyRegistry8e
48.32	12738.000000	19.31	Hexadecanoic acid, methyl ester	788	863	112-39-0	92866791.55	1.14	mainlib
48.32	12738.000000	19.31	Hexadecanoic acid, methyl ester	783	894	112-39-0	92866791.55	1.14	replib
48.32	12738.000000	19.31	HEXADECANOIC ACID, METHYL ESTER	782	923	112-39-0	92866791.55	1.14	WileyRegistry8e

Hit Spectrum | Compound Structure

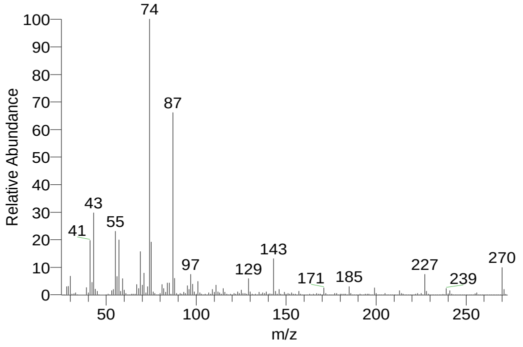

PENTADECANOIC ACID, 14-METHYL-, METHYL ESTER
Formula C17H34O2, MW 270, CAS# 5129-60-2, Entry# 161312
METHYL 14-METHYLPENTADECANOATE

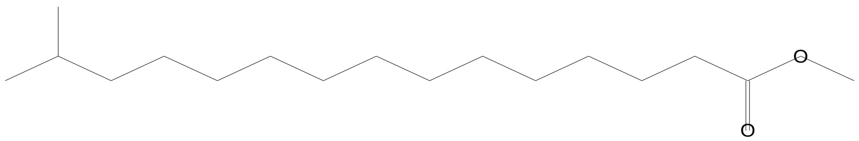

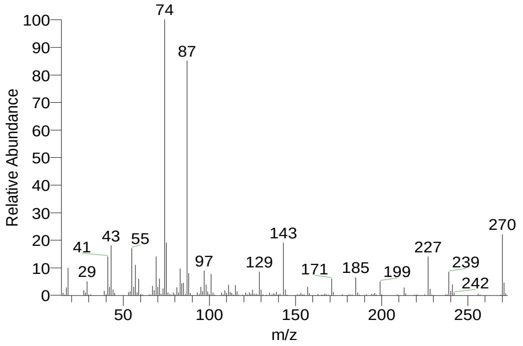

HEXADECANOIC ACID, METHYL ESTER
Formula C17H34O2, MW 270, CAS# 112-39-0, Entry# 161278
METHYL HEXADECANOATE

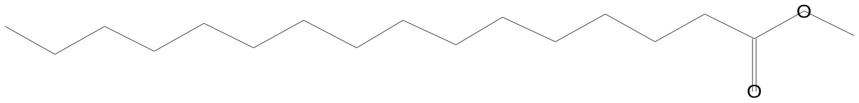

There is no signature data to report.

Library Search Report

Hit Spectrum | Compound Structure

Hexadecanoic acid, methyl ester
Formula C17H34O2, MW 270, CAS# 112-39-0, Entry# 48957
Palmitic acid, methyl ester

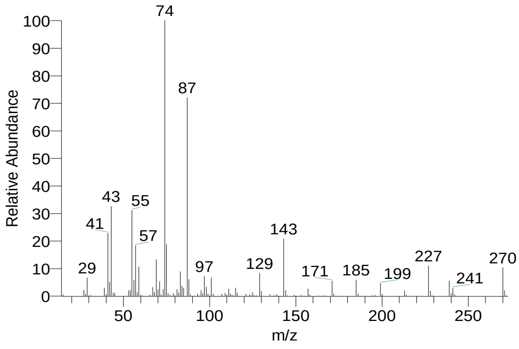

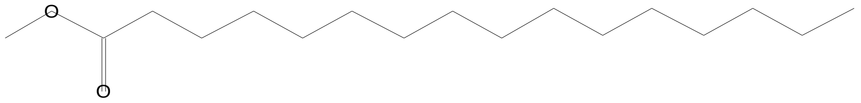

Hexadecanoic acid, methyl ester
Formula C17H34O2, MW 270, CAS# 112-39-0, Entry# 12041
Palmitic acid, methyl ester

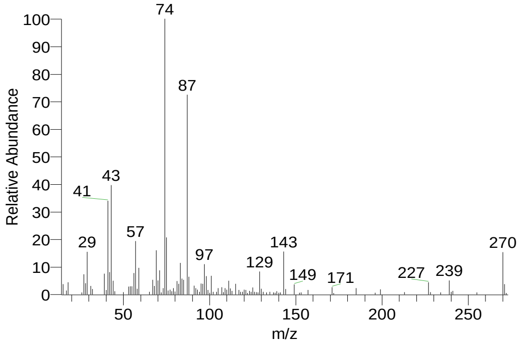

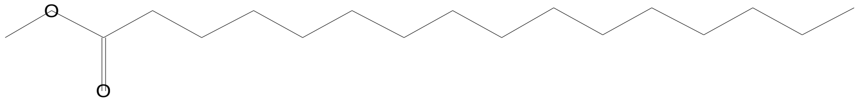

HEXADECANOIC ACID, METHYL ESTER
Formula C17H34O2, MW 270, CAS# 112-39-0, Entry# 161275
METHYL HEXADECANOATE

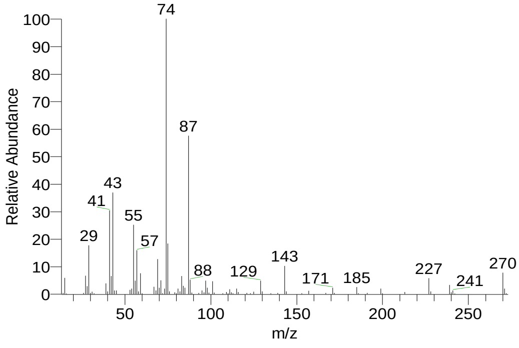

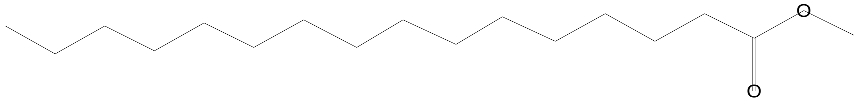

There is no signature data to report.

Library Search Report

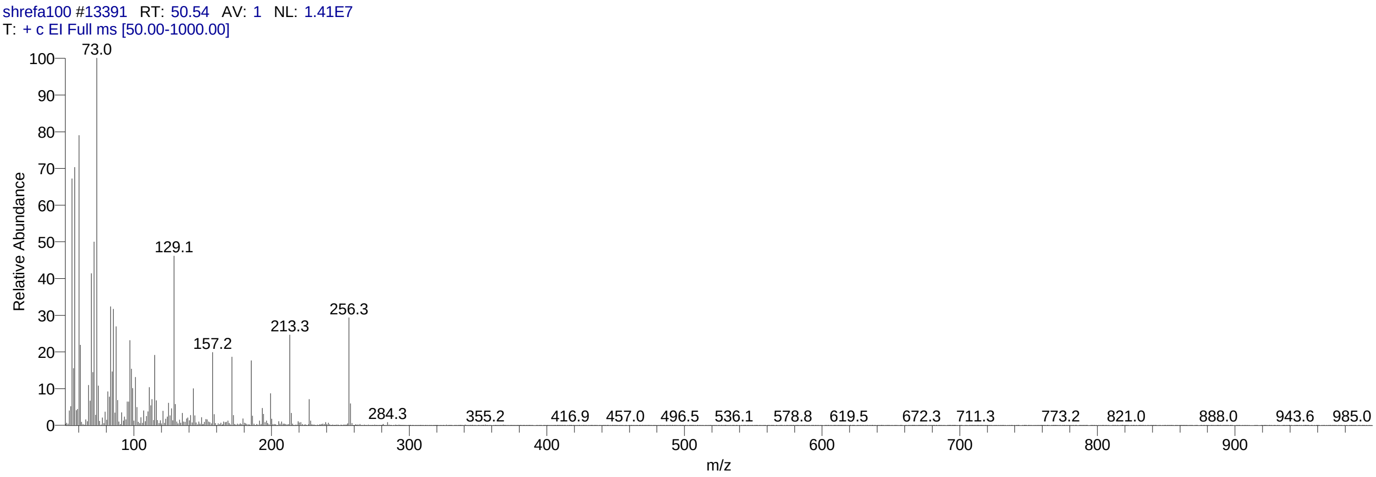

RT	Scan #	Probability	Compound Name	SI	RSI	Cas #	Area	Area %	Library
50.54	13391.000000	70.16	n-Hexadecanoic acid	901	935	57-10-3	676930395.70	8.29	replib
50.54	13391.000000	70.16	HEXADECANOIC ACID	893	918	57-10-3	676930395.70	8.29	WileyRegistry8e
50.54	13391.000000	70.16	n-Hexadecanoic acid	890	919	57-10-3	676930395.70	8.29	replib
50.54	13391.000000	70.16	HEXADECANOIC ACID	889	914	57-10-3	676930395.70	8.29	WileyRegistry8e
50.54	13391.000000	70.16	n-Hexadecanoic acid	874	910	57-10-3	676930395.70	8.29	replib

Hit Spectrum	Compound Structure

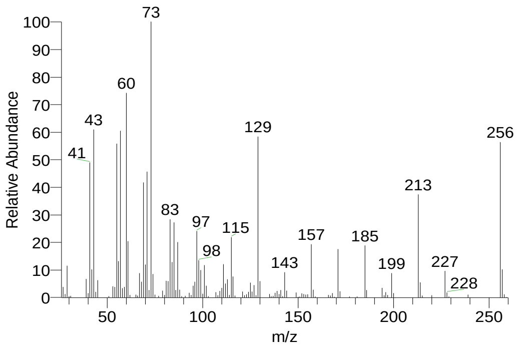

n-Hexadecanoic acid
Formula C16H32O2, MW 256, CAS# 57-10-3, Entry# 11136
Hexadecanoic acid

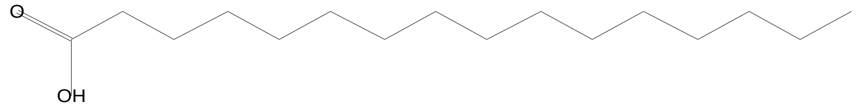

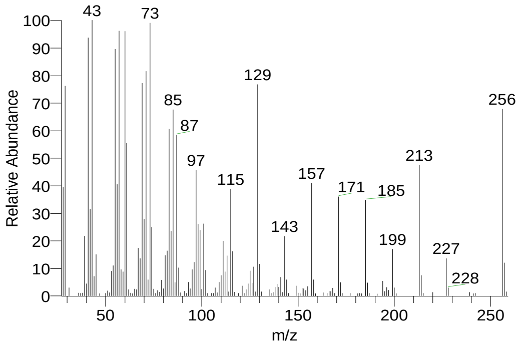

HEXADECANOIC ACID
Formula C16H32O2, MW 256, CAS# 57-10-3, Entry# 397116
HEXADECANOATE

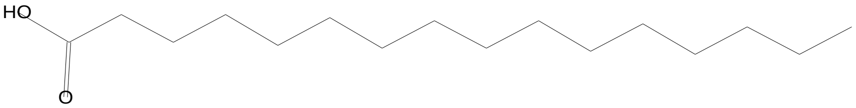

There is no signature data to report.

Library Search Report

Hit Spectrum | Compound Structure

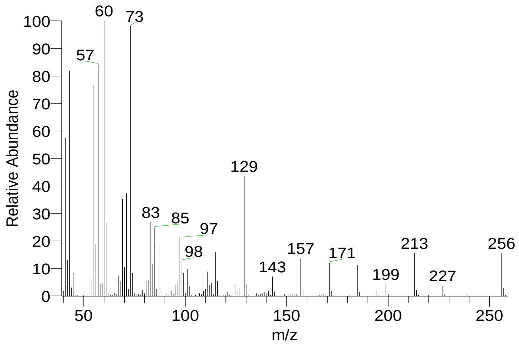

n-Hexadecanoic acid
Formula C16H32O2, MW 256, CAS# 57-10-3, Entry# 8750
Hexadecanoic acid

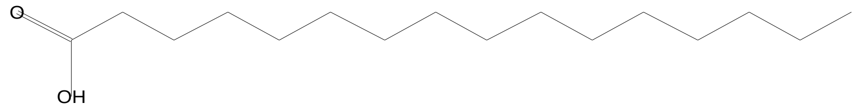

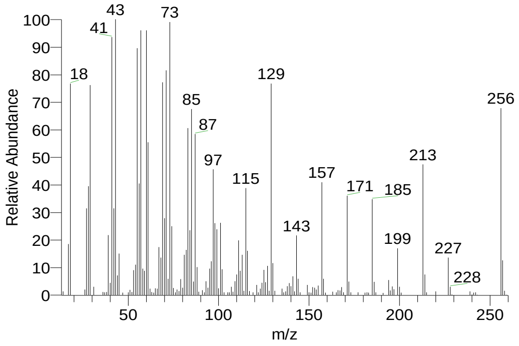

HEXADECANOIC ACID
Formula C16H32O2, MW 256, CAS# 57-10-3, Entry# 146744
HEXADECANOATE

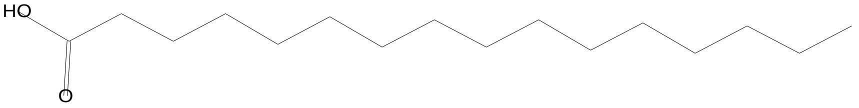

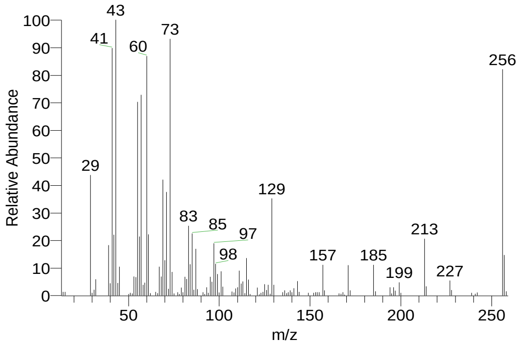

n-Hexadecanoic acid
Formula C16H32O2, MW 256, CAS# 57-10-3, Entry# 3180
Hexadecanoic acid

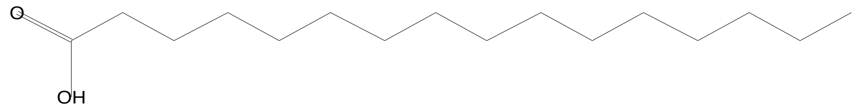

There is no signature data to report.

Library Search Report

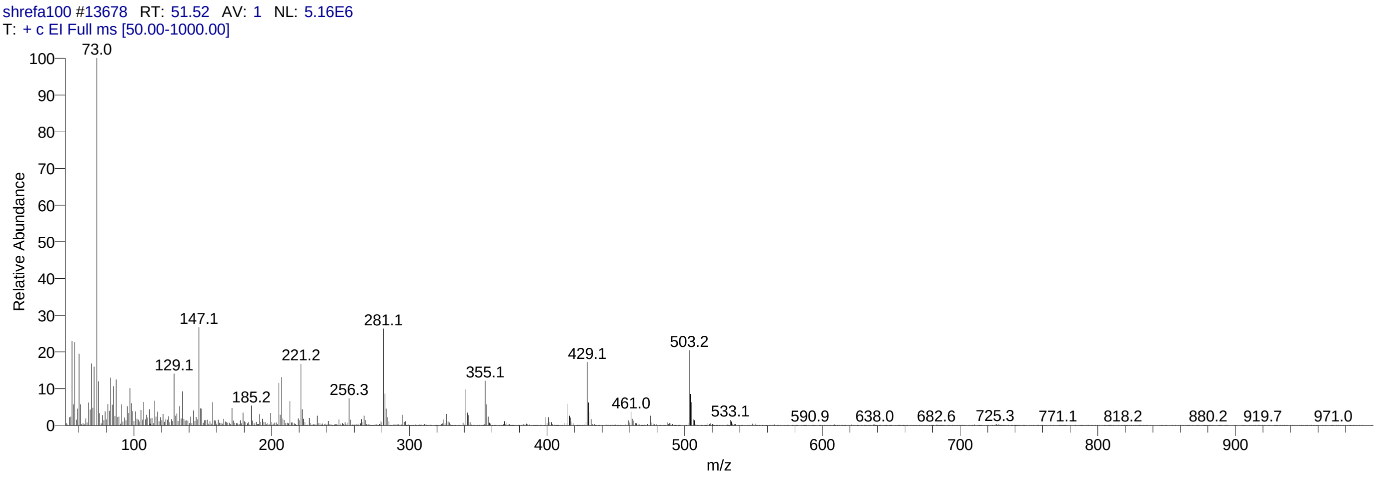

RT	Scan #	Probability	Compound Name	SI	RSI	Cas #	Area	Area %	Library
51.52	13678.000000	39.34	Cyclodecasiloxane, eicosamethyl-	712	775	18772-36-6	168231345.95	2.06	mainlib
51.52	13678.000000	39.34	2,2,4,4,6,6,8,8,10,10,12,12,14,14,16,16,18,18,20,20-ICOSAMETHYLCYCLODECASILOXANE #	712	775	18772-36-6	168231345.95	2.06	WileyRegistry8e
51.52	13678.000000	21.47	1H-PURIN-6-AMINE, [(2-FLUOROPHENYL)METHYL]-	695	761	74421-44-6	168231345.95	2.06	WileyRegistry8e
51.52	13678.000000	14.73	SILICONE OIL	684	743	NA	168231345.95	2.06	WileyRegistry8e
51.52	13678.000000	14.73	SILIKONFETT SE30 (GREVELS)	684	743	NA	168231345.95	2.06	WileyRegistry8e

Hit Spectrum | Compound Structure

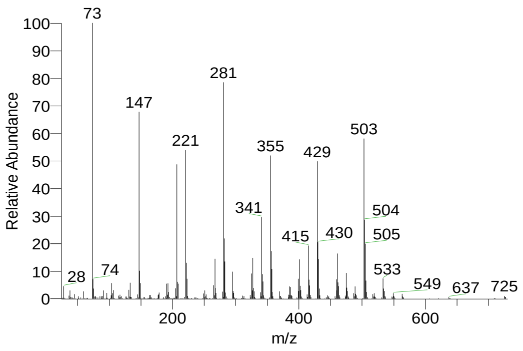

Cyclodecasiloxane, eicosamethyl-
Formula C20H60O10Si10, MW 740, CAS# 18772-36-6, Entry# 47864
2,2,4,4,6,6,8,8,10,10,12,12,14,14,16,16,18,18,20,20-Icosamethylcyclodecasiloxane #

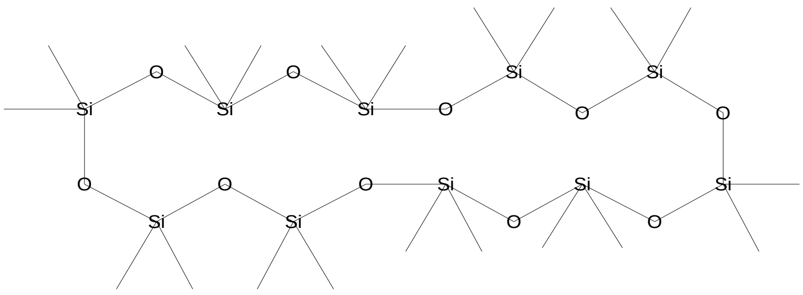

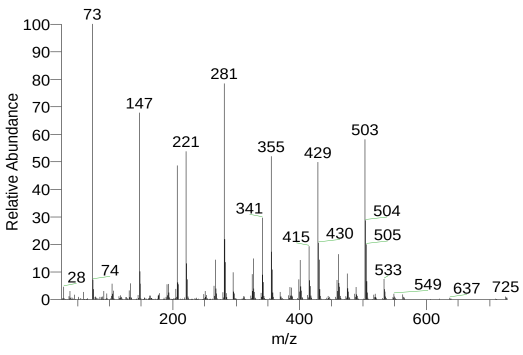

2,2,4,4,6,6,8,8,10,10,12,12,14,14,16,16,18,18,20,20-ICOSAMETHYLCYCLODECASILOXANE #
Formula C20H60O10Si10, MW 740, CAS# 18772-36-6, Entry# 380233
2,2,4,4,6,6,8,8,10,10,12,12,14,14,16,16,18,18,20,20-ICOSAMETHYLCYCLODECASILOXANE

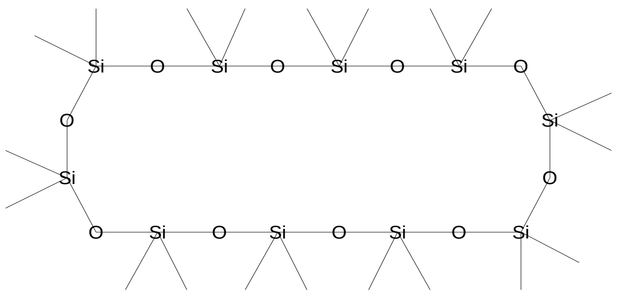

There is no signature data to report.

Library Search Report

Hit Spectrum | Compound Structure

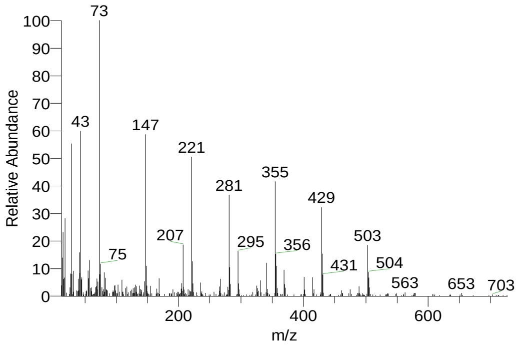

1H-PURIN-6-AMINE, [(2-FLUOROPHENYL)METHYL]-
Formula C12H10FN5, MW 243, CAS# 74421-44-6, Entry# 132518

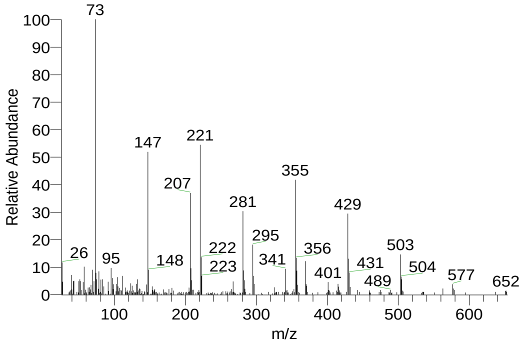

SILICONE OIL
Formula , MW 0, CAS# NA, Entry# 305490
SILIKONFETT SE30 (GREVELS)

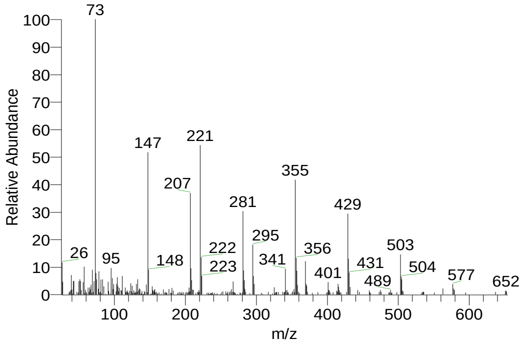

SILIKONFETT SE30 (GREVELS)
Formula , MW 0, CAS# NA, Entry# 392776

There is no signature data to report.

Library Search Report

shrefa100 #14060 RT: 52.82 AV: 1 NL: 2.39E6
T: + c EI Full ms [50.00-1000.00]

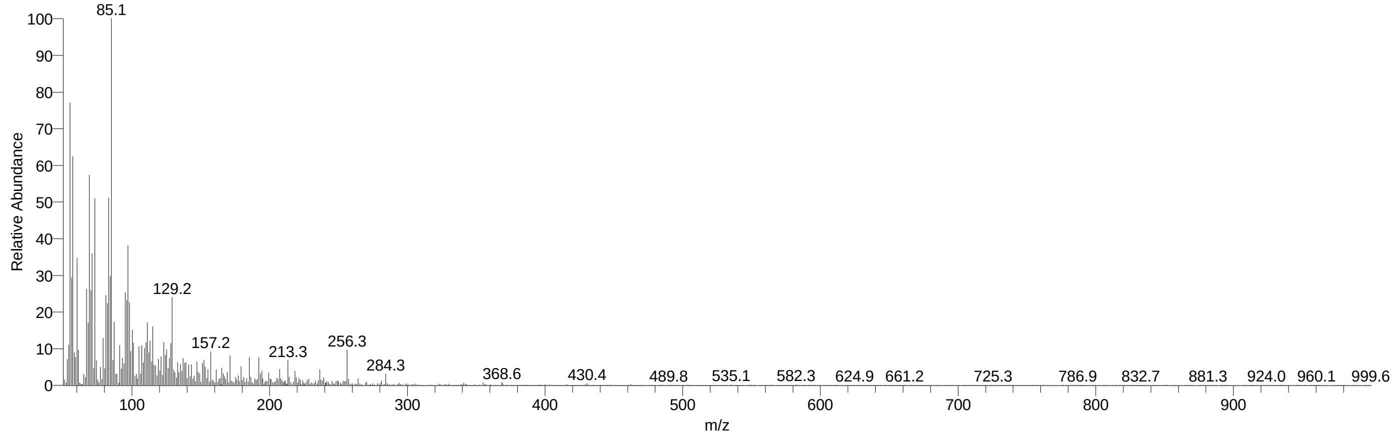

RT	Scan #	Probability	Compound Name	SI	RSI	Cas #	Area	Area %	Library
52.82	14060.000000	32.38	HEXADECANOIC ACID, 2,3-DIHYDROXYPROPYL ESTER	775	783	542-44-9	125894872.82	1.54	WileyRegistry8e
52.82	14060.000000	14.75	9-OCTADECENOIC ACID (Z)-	755	813	112-80-1	125894872.82	1.54	WileyRegistry8e
52.82	14060.000000	14.75	Oleic Acid	748	839	112-80-1	125894872.82	1.54	replib
52.82	14060.000000	11.29	9-OCTADECENOIC ACID	748	839	NA	125894872.82	1.54	WileyRegistry8e
52.82	14060.000000	10.42	Estra-1,3,5(10)-trien-17á-ol	746	810	2529-64-8	125894872.82	1.54	mainlib

Hit Spectrum	Compound Structure

HEXADECANOIC ACID, 2,3-DIHYDROXYPROPYL ESTER
Formula C19H38O4, MW 330, CAS# 542-44-9, Entry# 214589
2,3-DIHYDROXYPROPYL PALMITATE #

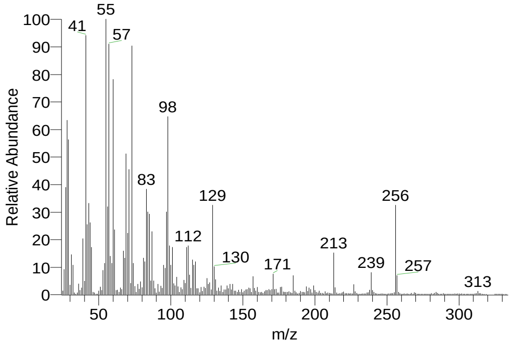

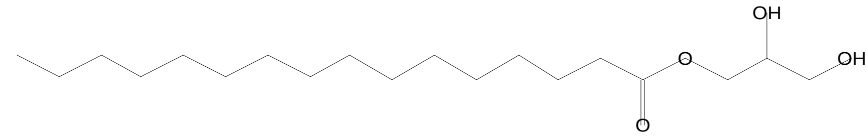

9-OCTADECENOIC ACID (Z)-
Formula C18H34O2, MW 282, CAS# 112-80-1, Entry# 172902
OCTADEC-9-ENOIC ACID

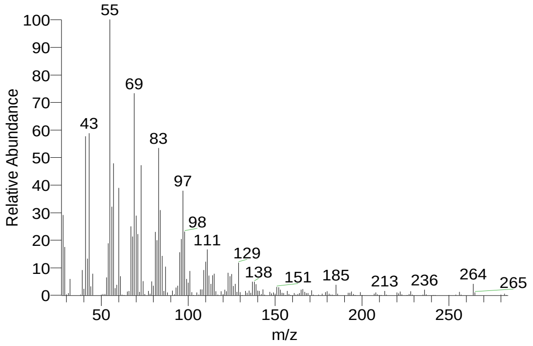

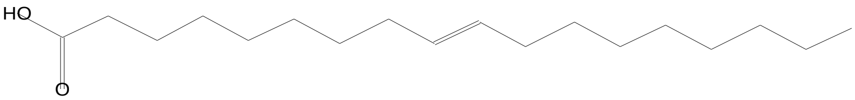

There is no signature data to report.

Library Search Report

Hit Spectrum | Compound Structure

SI 748, RSI 839, replib, Entry# 5489, CAS# 112-80-1, Oleic Acid

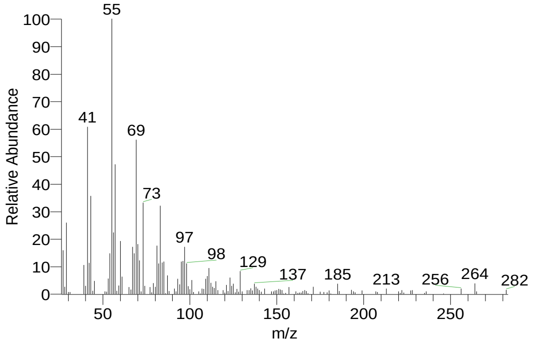

Oleic Acid
Formula C18H34O2, MW 282, CAS# 112-80-1, Entry# 5489
9-Octadecenoic acid (Z)-

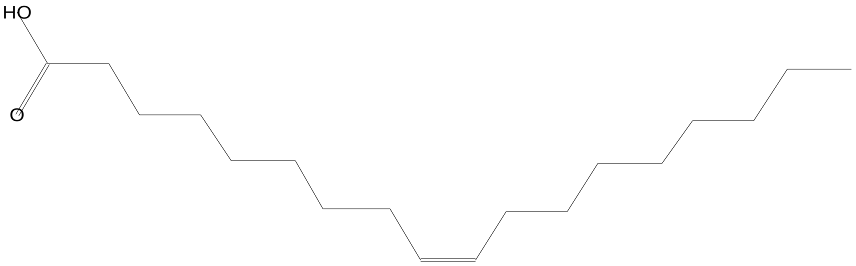

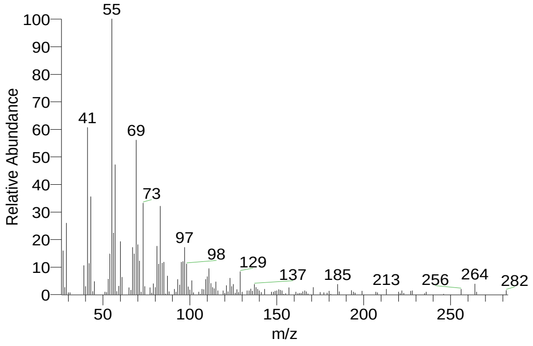

9-OCTADECENOIC ACID
Formula C18H34O2, MW 282, CAS# NA, Entry# 384641
9-OCTADECENSAEURE, (Z)- (OELSAEURE)

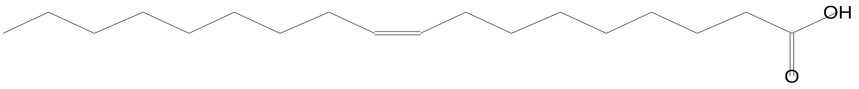

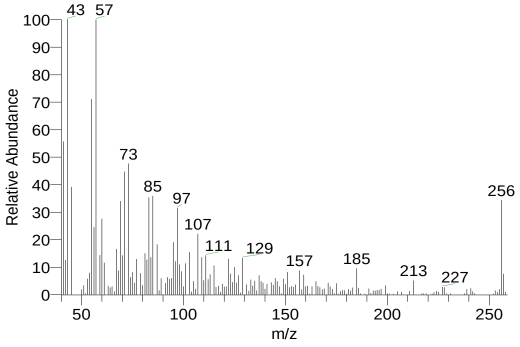

Estra-1,3,5(10)-trien-17á-ol
Formula C18H24O, MW 256, CAS# 2529-64-8, Entry# 8289
Estra-1,3,5(10)-trien-17-ol, (17á)-

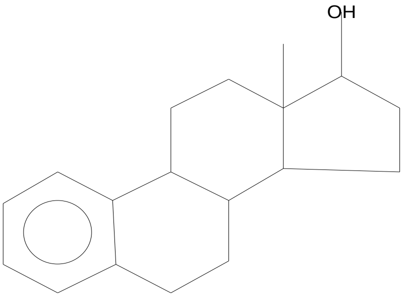

There is no signature data to report.

Library Search Report

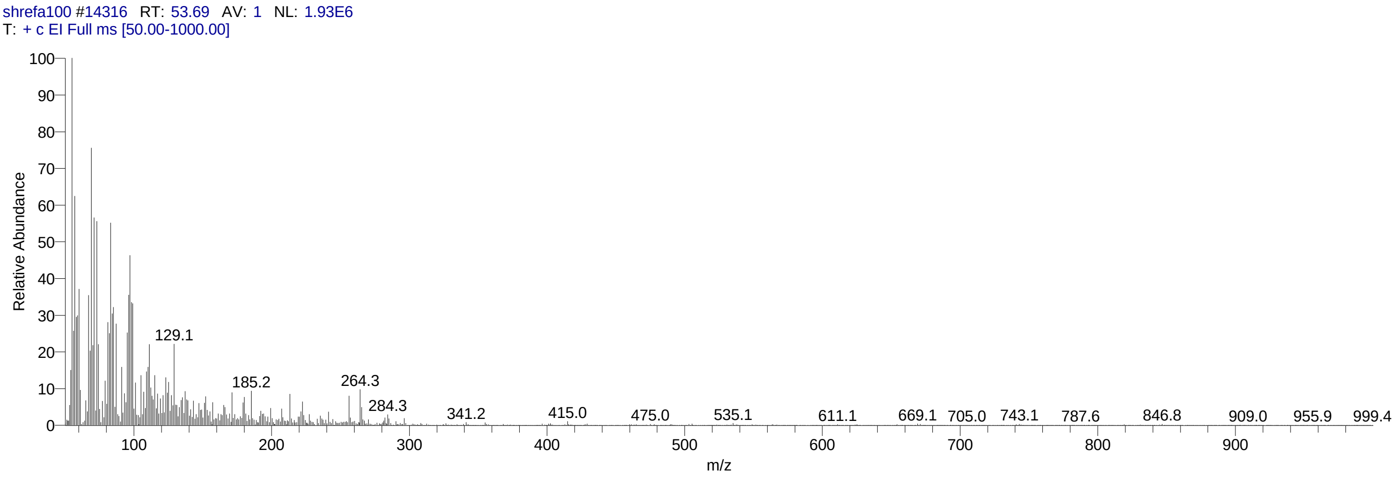

RT	Scan #	Probability	Compound Name	SI	RSI	Cas #	Area	Area %	Library
53.69	14316.000000	11.32	Oleic Acid	795	821	112-80-1	46943271.48	0.57	replib
53.69	14316.000000	7.31	2-HYDROXY-3-[(9E)-9-OCTADECENOYLOXY]PROPYL (9E)-9-OCTADECENOATE #	782	797	2465-32-9	46943271.48	0.57	WileyRegistry8e
53.69	14316.000000	5.89	HEXADECANOIC ACID, 2,3-DIHYDROXYPROPYL ESTER	777	791	542-44-9	46943271.48	0.57	WileyRegistry8e
53.69	14316.000000	4.63	10-Octadecenoic acid, methyl ester	771	794	13481-95-3	46943271.48	0.57	mainlib
53.69	14316.000000	4.63	10-OCTADECENOIC ACID, METHYL ESTER	771	794	13481-95-3	46943271.48	0.57	WileyRegistry8e

Hit Spectrum

Compound Structure

SI 795, RSI 821, replib, Entry# 5762, CAS# 112-80-1, Oleic Acid

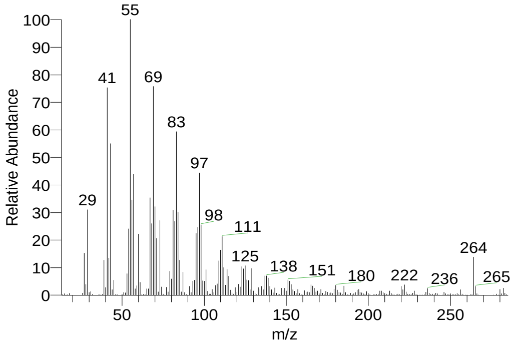

Oleic Acid
Formula C18H34O2, MW 282, CAS# 112-80-1, Entry# 5762
9-Octadecenoic acid (Z)-

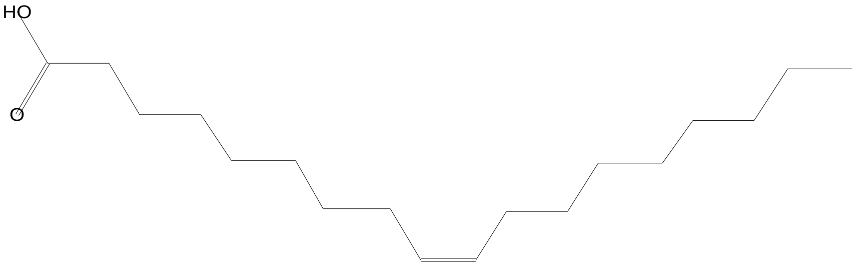

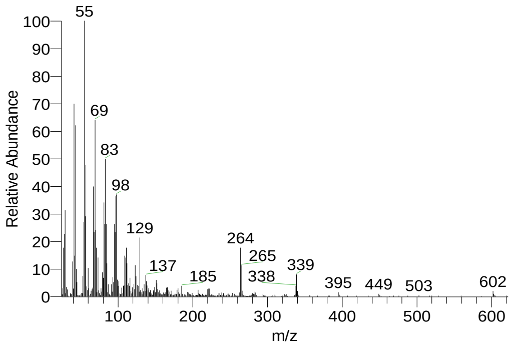

2-HYDROXY-3-[(9E)-9-OCTADECENOYLOXY]PROPYL (9E)-9-OCTADECENOATE #
Formula C39H72O5, MW 620, CAS# 2465-32-9, Entry# 298152
(Z,Z)-1,3-DIOCTADECENOYL GLYCEROL

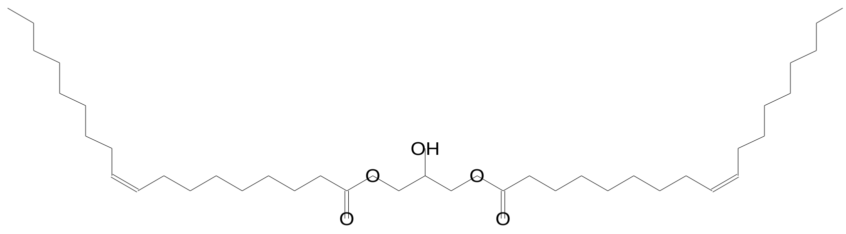

There is no signature data to report.

Library Search Report

Hit Spectrum

Compound Structure

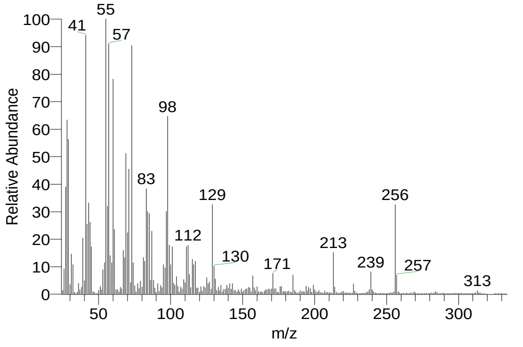

HEXADECANOIC ACID, 2,3-DIHYDROXYPROPYL ESTER
Formula C19H38O4, MW 330, CAS# 542-44-9, Entry# 214589
2,3-DIHYDROXYPROPYL PALMITATE #

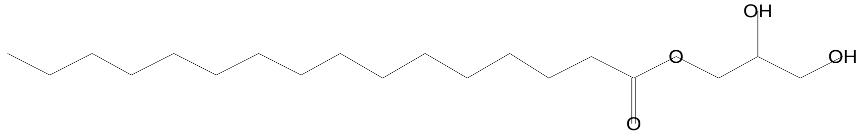

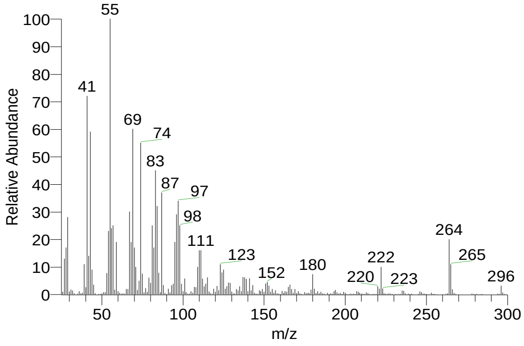

10-Octadecenoic acid, methyl ester
Formula C19H36O2, MW 296, CAS# 13481-95-3, Entry# 20928
Methyl 10-octadecenoate

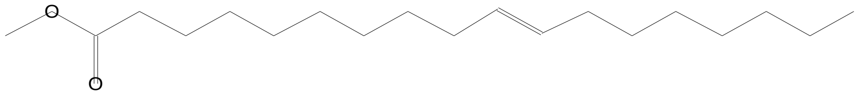

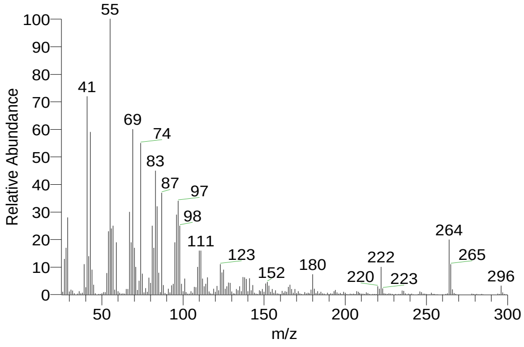

10-OCTADECENOIC ACID, METHYL ESTER
Formula C19H36O2, MW 296, CAS# 13481-95-3, Entry# 186173
METHYL OCTADEC-10-ENOATE

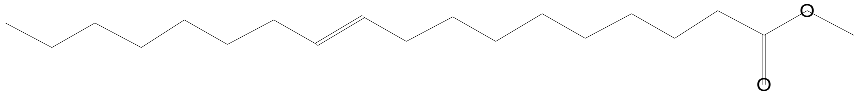

There is no signature data to report.

Library Search Report

shrefa100 #14342 RT: 53.78 AV: 1 NL: 1.75E6
T: + c EI Full ms [50.00-1000.00]

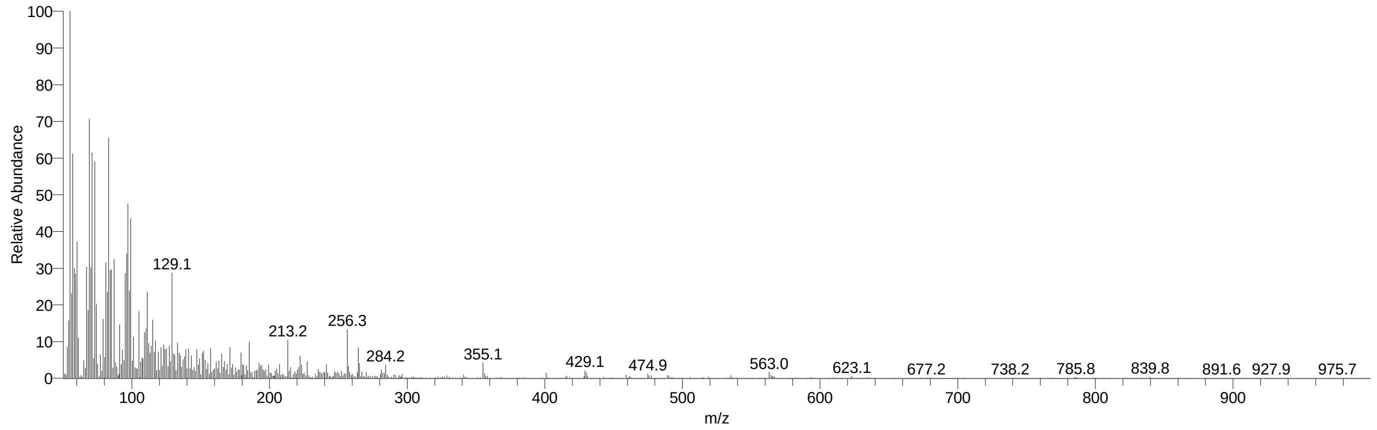

RT	Scan #	Probability	Compound Name	SI	RSI	Cas #	Area	Area %	Library
53.78	14342.000000	24.74	HEXADECANOIC ACID, 2,3-DIHYDROXYPROPYL ESTER	752	784	542-44-9	60444602.07	0.74	WileyRegistry8e
53.78	14342.000000	12.01	2-HYDROXY-3-[(9E)-9-OCTADECENOYLOXY]PROPYL (9E)-9-OCTADECENOATE #	733	763	2465-32-9	60444602.07	0.74	WileyRegistry8e
53.78	14342.000000	7.52	DI-2-BENZOTHIAZOLE DISULFANE	719	748	NA	60444602.07	0.74	WileyRegistry8e
53.78	14342.000000	5.61	Oleic Acid	711	837	112-80-1	60444602.07	0.74	replib
53.78	14342.000000	5.61	9-OCTADECENOIC ACID	711	837	NA	60444602.07	0.74	WileyRegistry8e

Hit Spectrum

Compound Structure

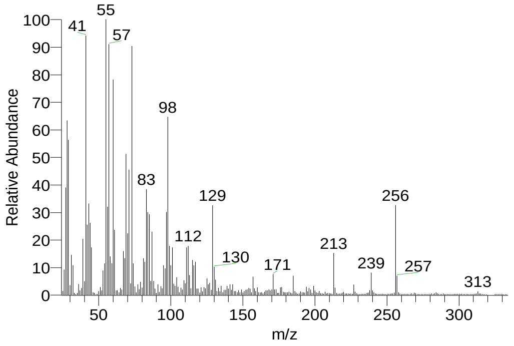

HEXADECANOIC ACID, 2,3-DIHYDROXYPROPYL ESTER
Formula C19H38O4, MW 330, CAS# 542-44-9, Entry# 214589
2,3-DIHYDROXYPROPYL PALMITATE #

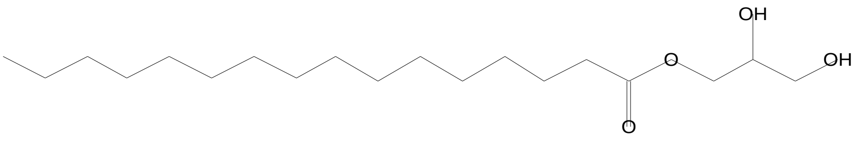

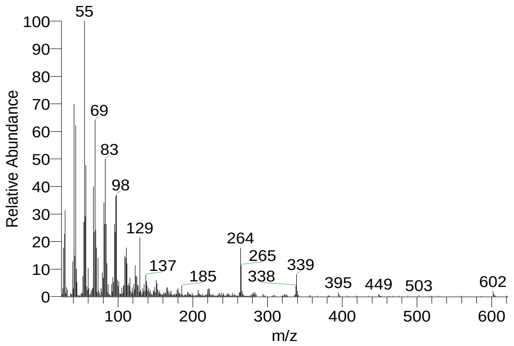

2-HYDROXY-3-[(9E)-9-OCTADECENOYLOXY]PROPYL (9E)-9-OCTADECENOATE #
Formula C39H72O5, MW 620, CAS# 2465-32-9, Entry# 298152
(Z,Z)-1,3-DIOCTADECENOYL GLYCEROL

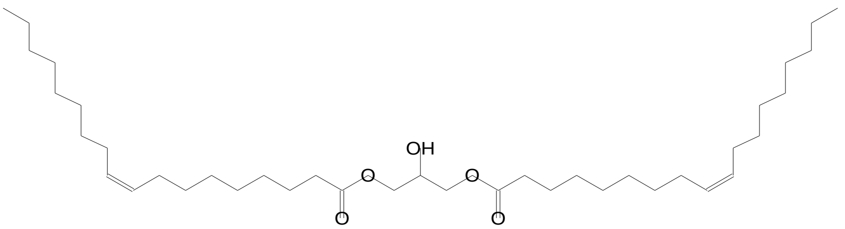

There is no signature data to report.

Library Search Report

Hit Spectrum | Compound Structure

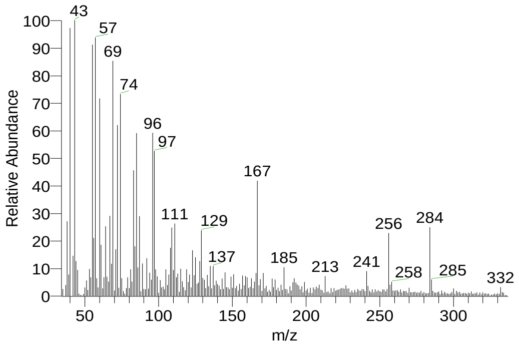

DI-2-BENZOTHIAZOLE DISULFANE
Formula C14H8N2S4, MW 332, CAS# NA, Entry# 215624

SI 711, RSI 837, replib, Entry# 5489, CAS# 112-80-1, Oleic Acid

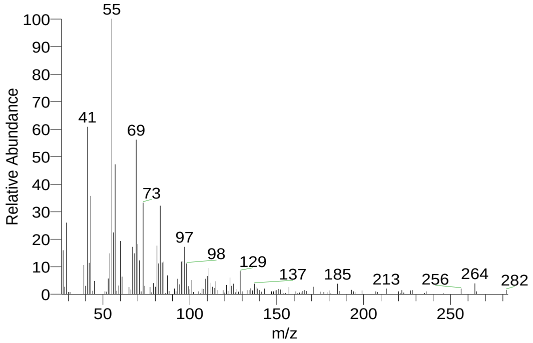

Oleic Acid
Formula C18H34O2, MW 282, CAS# 112-80-1, Entry# 5489
9-Octadecenoic acid (Z)-

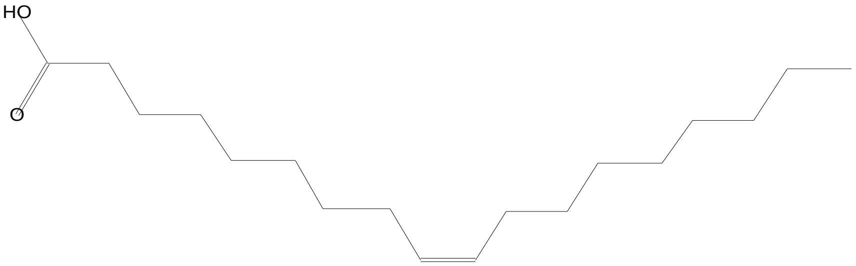

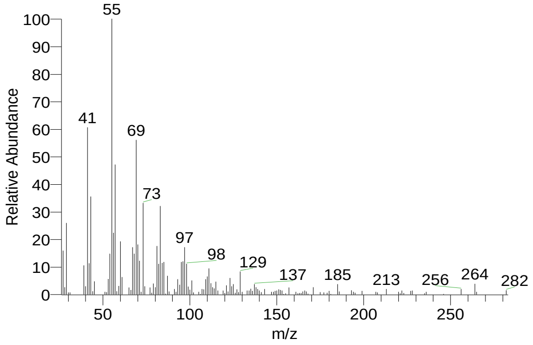

9-OCTADECENOIC ACID
Formula C18H34O2, MW 282, CAS# NA, Entry# 384641
9-OCTADECENSAEURE, (Z)- (OELSAEURE)

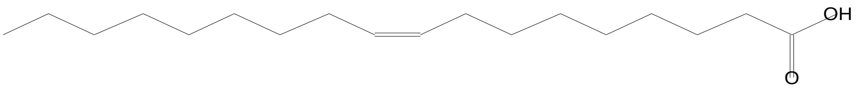

There is no signature data to report.

Library Search Report

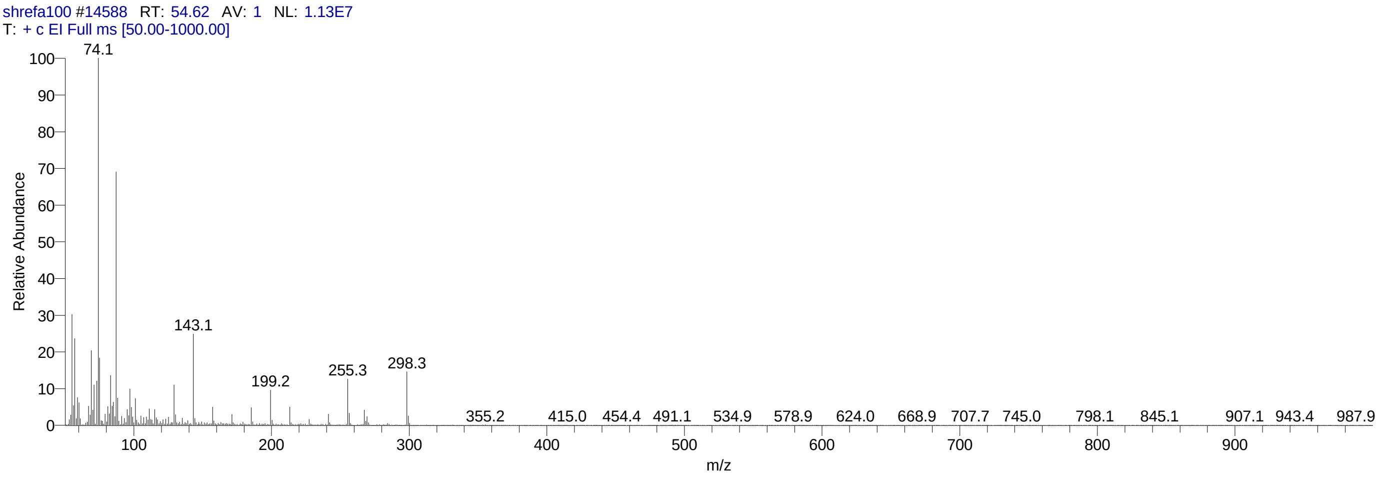

RT	Scan #	Probability	Compound Name	SI	RSI	Cas #	Area	Area %	Library
54.62	14588.000000	58.31	OCTADECANOIC ACID, METHYL ESTER	872	898	112-61-8	533333494.49	6.53	WileyRegistry8e
54.62	14588.000000	58.31	OCTADECANOIC ACID, METHYL ESTER	862	876	112-61-8	533333494.49	6.53	WileyRegistry8e
54.62	14588.000000	58.31	Methyl stearate	853	882	112-61-8	533333494.49	6.53	mainlib
54.62	14588.000000	19.53	HEPTADECANOIC ACID, 16-METHYL-, METHYL ESTER	848	877	5129-61-3	533333494.49	6.53	WileyRegistry8e
54.62	14588.000000	19.53	Heptadecanoic acid, 16-methyl-, methyl ester	846	874	5129-61-3	533333494.49	6.53	mainlib

Hit Spectrum	Compound Structure

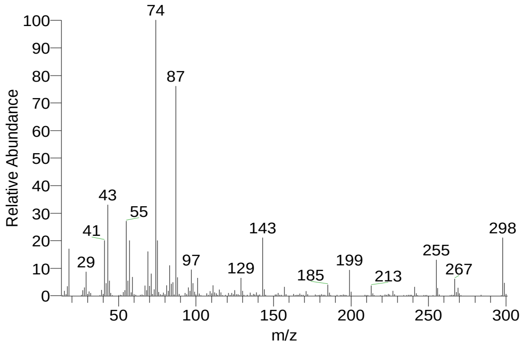

OCTADECANOIC ACID, METHYL ESTER
Formula C19H38O2, MW 298, CAS# 112-61-8, Entry# 187911
METHYL OCTADECANOATE

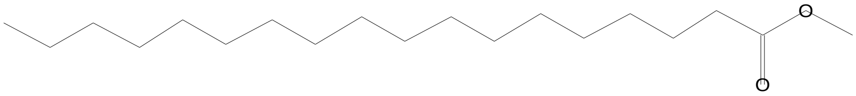

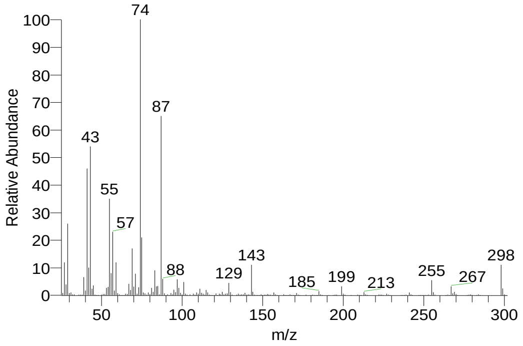

OCTADECANOIC ACID, METHYL ESTER
Formula C19H38O2, MW 298, CAS# 112-61-8, Entry# 187918
METHYL OCTADECANOATE

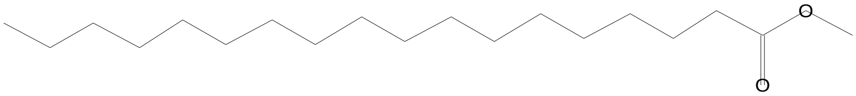

There is no signature data to report.

Library Search Report

Hit Spectrum | Compound Structure

SI 853, RSI 882, mainlib, Entry# 48980, CAS# 112-61-8, Methyl stearate

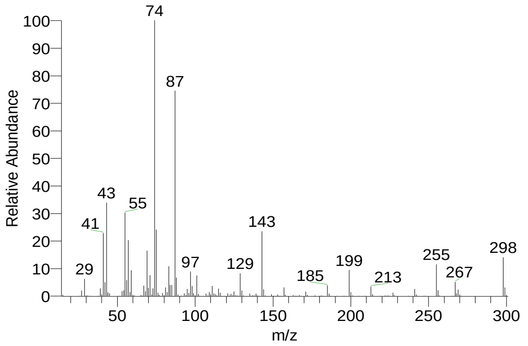

Methyl stearate
Formula C19H38O2, MW 298, CAS# 112-61-8, Entry# 48980
Octadecanoic acid, methyl ester

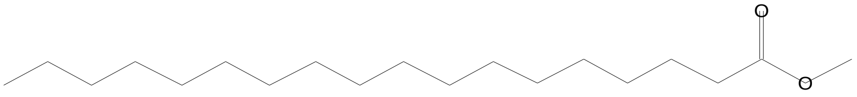

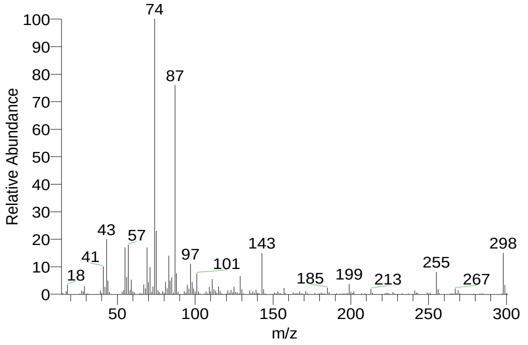

HEPTADECANOIC ACID, 16-METHYL-, METHYL ESTER
Formula C19H38O2, MW 298, CAS# 5129-61-3, Entry# 188009
METHYL 16-METHYLHEPTADECANOATE

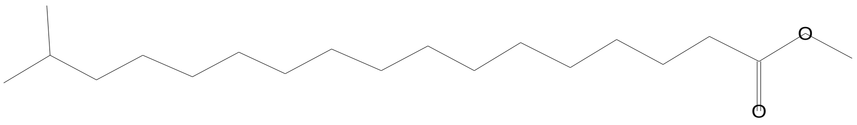

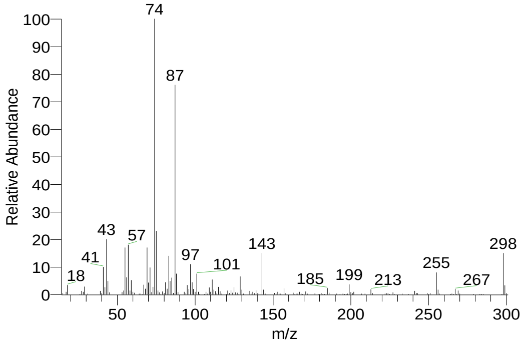

Heptadecanoic acid, 16-methyl-, methyl ester
Formula C19H38O2, MW 298, CAS# 5129-61-3, Entry# 49021
Methyl isostearate

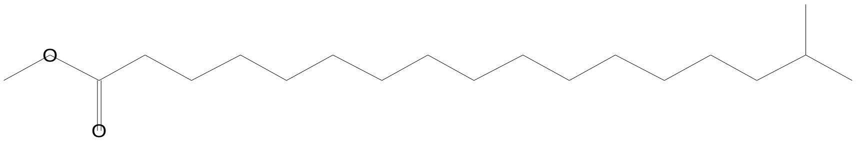

There is no signature data to report.

Library Search Report

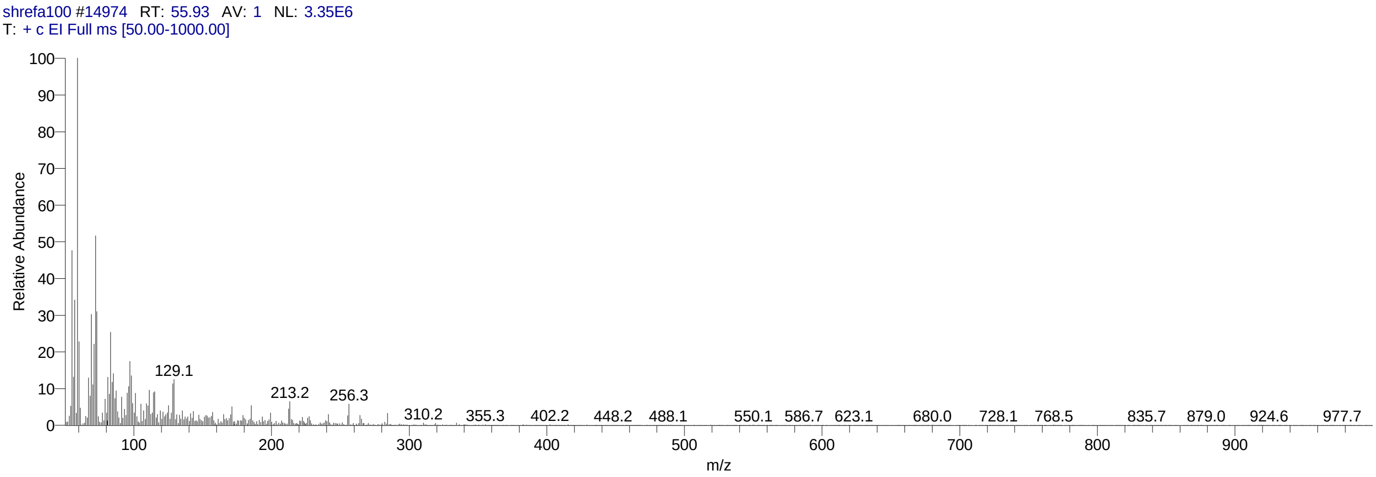

RT	Scan #	Probability	Compound Name	SI	RSI	Cas #	Area	Area %	Library
55.93	14974.000000	27.22	HEXADECANOIC ACID, 2,3-DIHYDROXYPROPYL ESTER	745	749	542-44-9	97930877.00	1.20	WileyRegistry8e
55.93	14974.000000	14.86	DI-2-BENZOTHIAZOLE DISULFANE	728	730	NA	97930877.00	1.20	WileyRegistry8e
55.93	14974.000000	9.60	18,19-Secoyohimban-19-oic acid, 16,17,20,21-tetradehydro-16-(hydroxymethyl)-, methyl ester, (15á,16E)-	715	742	5523-49-9	97930877.00	1.20	mainlib
55.93	14974.000000	9.60	18,19-SECOYOHIMBAN-19-OIC ACID, 16,17,20,21-TETRADEHYDRO-16-(HYDROXYMETHYL)-, METHYL ESTER, (15á,16E)-	715	742	5523-49-9	97930877.00	1.20	WileyRegistry8e
55.93	14974.000000	7.54	1-PROPYL-2-METHYL-7-METHOXY-5H,6H-PYRIDO[3,4-B]INDOLE	709	726	NA	97930877.00	1.20	WileyRegistry8e

Hit Spectrum

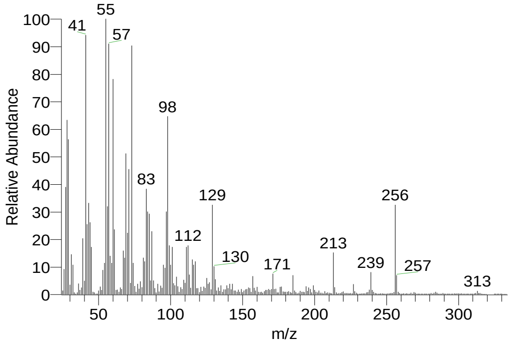

Compound Structure

HEXADECANOIC ACID, 2,3-DIHYDROXYPROPYL ESTER
Formula C19H38O4, MW 330, CAS# 542-44-9, Entry# 214589
2,3-DIHYDROXYPROPYL PALMITATE #

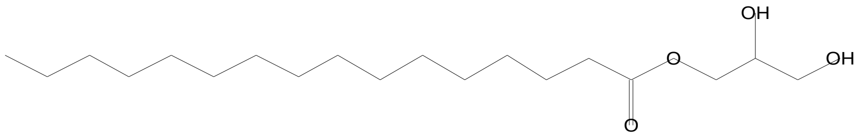

There is no signature data to report.

Library Search Report

Hit Spectrum	Compound Structure

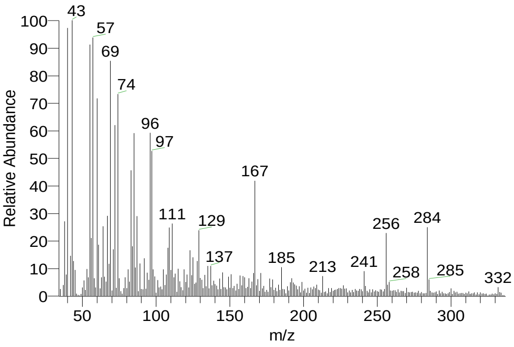

DI-2-BENZOTHIAZOLE DISULFANE
Formula C14H8N2S4, MW 332, CAS# NA, Entry# 215624

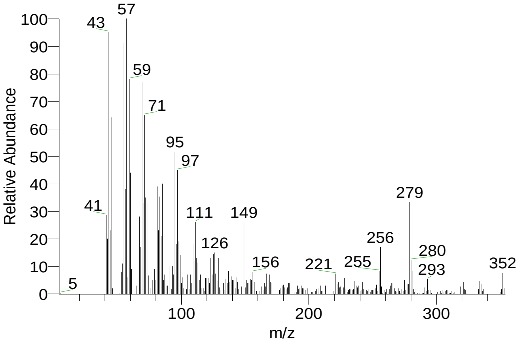

Formula C21H24N2O3, MW 352, CAS# 5523-49-9, Entry# 25985

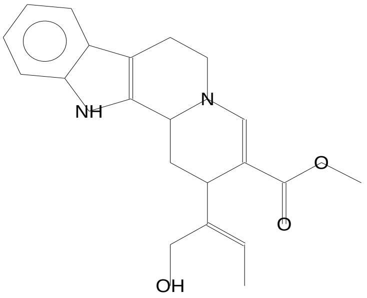

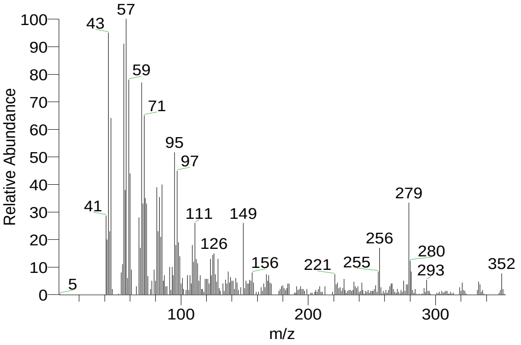

Formula C21H24N2O3, MW 352, CAS# 5523-49-9, Entry# 229989

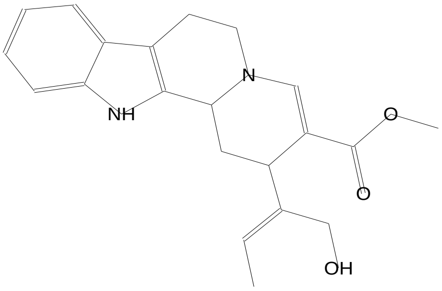

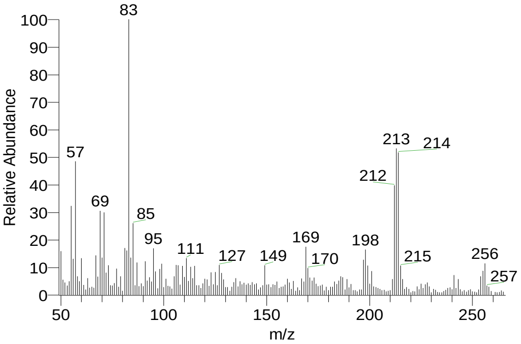

1-PROPYL-2-METHYL-7-METHOXY-5H,6H-PYRIDO[3,4-B]INDOLE
Formula C16H20N2O, MW 256, CAS# NA, Entry# 146736

There is no signature data to report.

Library Search Report

shrefa100 #15200 RT: 56.70 AV: 1 NL: 1.73E6
T: + c EI Full ms [50.00-1000.00]

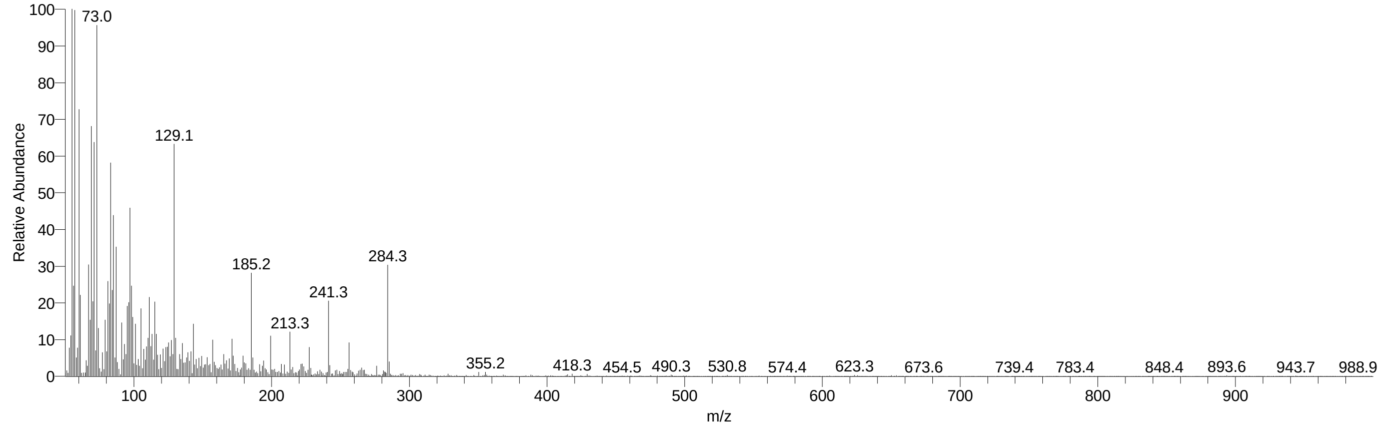

RT	Scan #	Probability	Compound Name	SI	RSI	Cas #	Area	Area %	Library
56.70	15200.000000	18.86	OCTADECANOIC ACID	784	838	57-11-4	88701632.56	1.09	WileyRegistry8e
56.70	15200.000000	17.39	HEXADECANOIC ACID, 2,3-DIHYDROXYPROPYL ESTER	782	791	542-44-9	88701632.56	1.09	WileyRegistry8e
56.70	15200.000000	12.63	OCTADECANOIC ACID, 2,3-DIHYDROXYPROPYL ESTER	773	784	123-94-4	88701632.56	1.09	WileyRegistry8e
56.70	15200.000000	18.86	Octadecanoic acid	755	803	57-11-4	88701632.56	1.09	mainlib
56.70	15200.000000	4.99	DI-2-BENZOTHIAZOLE DISULFANE	751	760	NA	88701632.56	1.09	WileyRegistry8e

Hit Spectrum | Compound Structure

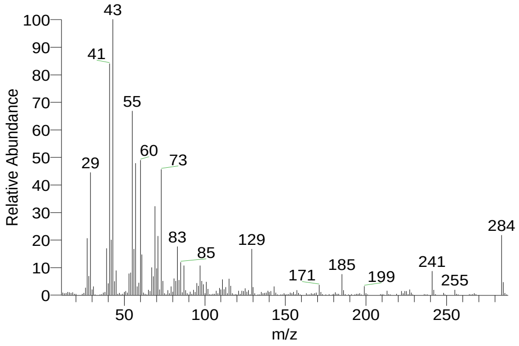

OCTADECANOIC ACID
Formula C18H36O2, MW 284, CAS# 57-11-4, Entry# 174897
STEARATE

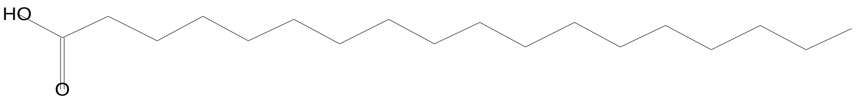

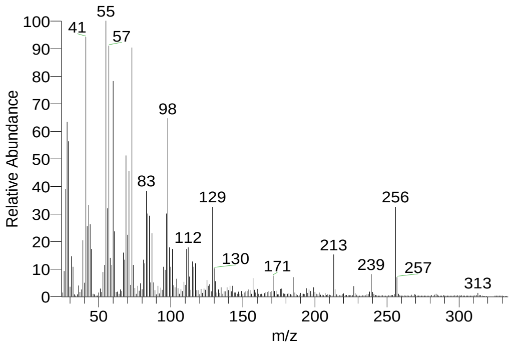

HEXADECANOIC ACID, 2,3-DIHYDROXYPROPYL ESTER
Formula C19H38O4, MW 330, CAS# 542-44-9, Entry# 214589
2,3-DIHYDROXYPROPYL PALMITATE #

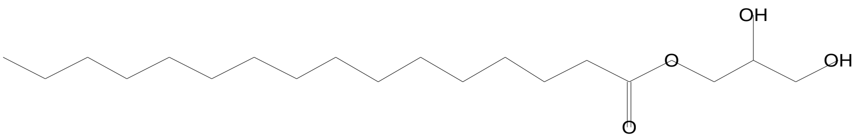

There is no signature data to report.

Library Search Report

Hit Spectrum	Compound Structure

OCTADECANOIC ACID, 2,3-DIHYDROXYPROPYL ESTER
Formula C21H42O4, MW 358, CAS# 123-94-4, Entry# 233812
2,3-DIHYDROXYPROPYL STEARATE #

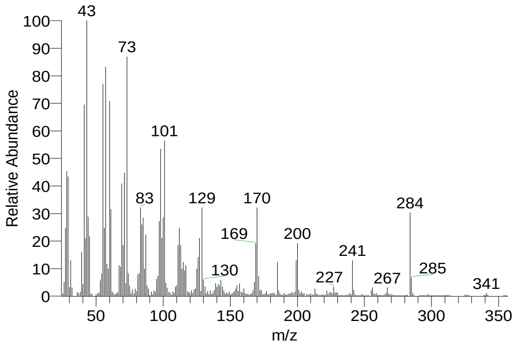

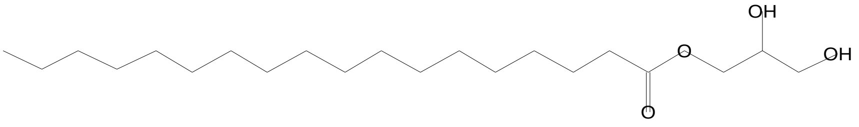

SI 755, RSI 803, mainlib, Entry# 9948, CAS# 57-11-4, Octadecanoic acid

Octadecanoic acid
Formula C18H36O2, MW 284, CAS# 57-11-4, Entry# 9948
Stearic acid

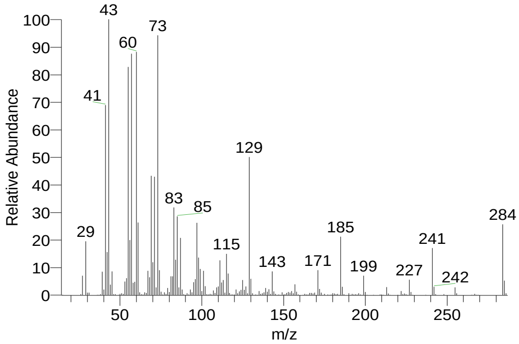

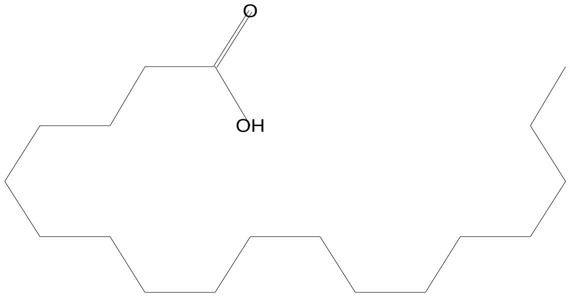

DI-2-BENZOTHIAZOLE DISULFANE
Formula C14H8N2S4, MW 332, CAS# NA, Entry# 215624

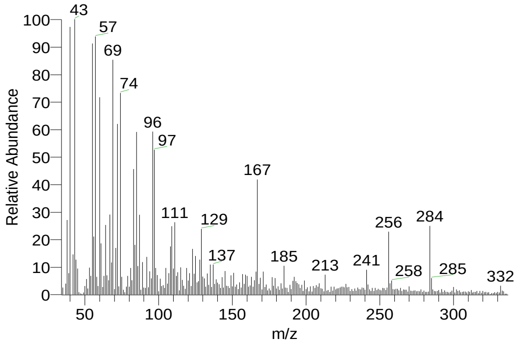

There is no signature data to report.

Library Search Report

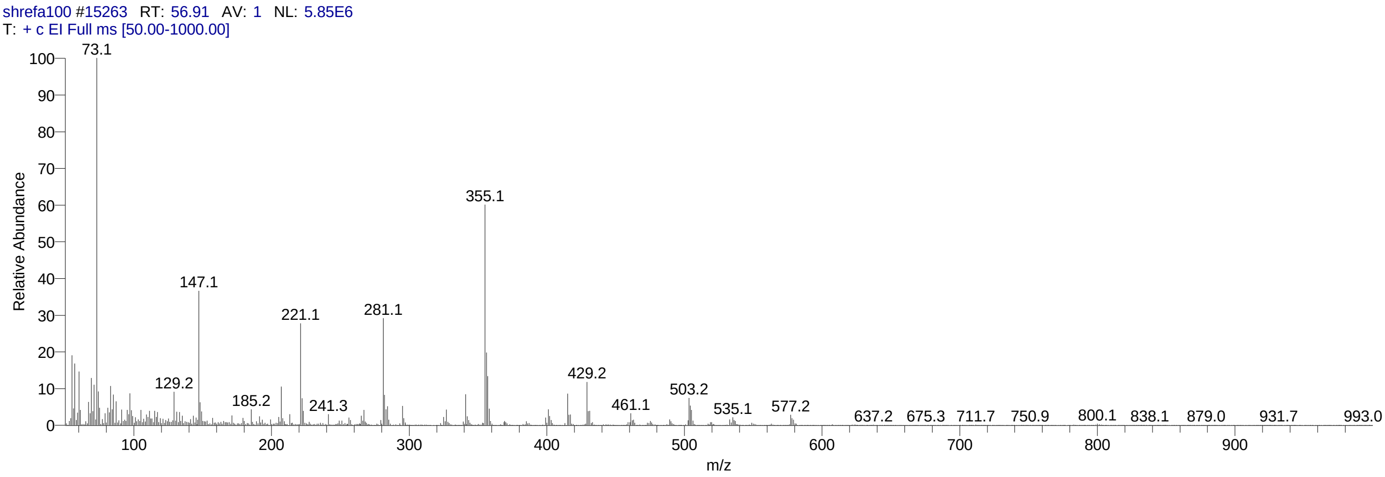

RT	Scan #	Probability	Compound Name	SI	RSI	Cas #	Area	Area %	Library
56.91	15263.000000	30.12	1H-PURIN-6-AMINE, [(2-FLUOROPHENYL)METHYL]-	745	805	74421-44-6	160989005.56	1.97	WileyRegistry8e
56.91	15263.000000	23.07	SILICONE OIL	738	791	NA	160989005.56	1.97	WileyRegistry8e
56.91	15263.000000	23.07	SILIKONFETT SE30 (GREVELS)	738	791	NA	160989005.56	1.97	WileyRegistry8e
56.91	15263.000000	16.29	Cyclodecasiloxane, eicosamethyl-	728	781	18772-36-6	160989005.56	1.97	mainlib
56.91	15263.000000	16.29	2,2,4,4,6,6,8,8,10,10,12,12,14,14,16,16,18,18,20,20-ICOSAMETHYLCYCLODECASILOXANE #	728	781	18772-36-6	160989005.56	1.97	WileyRegistry8e

Hit Spectrum	Compound Structure

1H-PURIN-6-AMINE, [(2-FLUOROPHENYL)METHYL]-
Formula C12H10FN5, MW 243, CAS# 74421-44-6, Entry# 132518

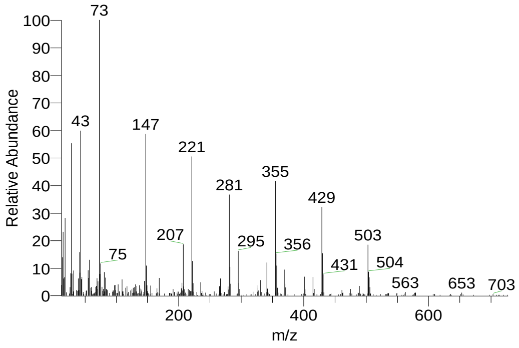

SILICONE OIL
Formula , MW 0, CAS# NA, Entry# 305490
SILIKONFETT SE30 (GREVELS)

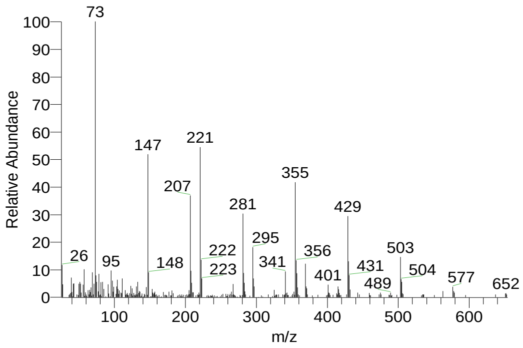

There is no signature data to report.

Library Search Report

Hit Spectrum | Compound Structure

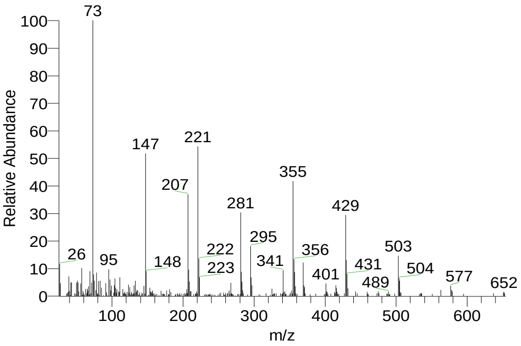

SILIKONFETT SE30 (GREVELS)
Formula , MW 0, CAS# NA, Entry# 392776

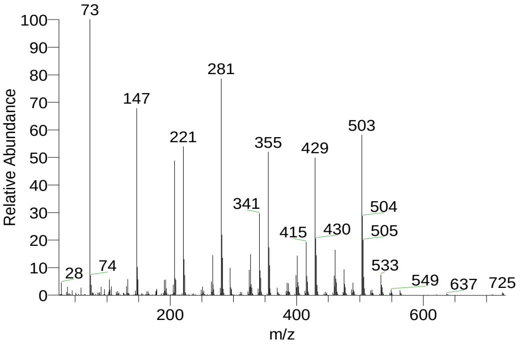

Cyclodecasiloxane, eicosamethyl-
Formula C20H60O10Si10, MW 740, CAS# 18772-36-6, Entry# 47864
2,2,4,4,6,6,8,8,10,10,12,12,14,14,16,16,18,18,20,20-Icosamethylcyclodecasiloxane #

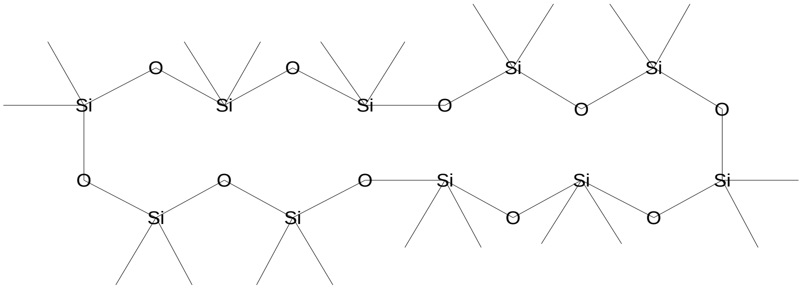

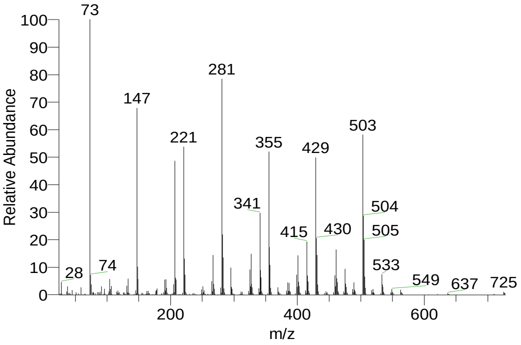

2,2,4,4,6,6,8,8,10,10,12,12,14,14,16,16,18,18,20,20-ICOSAMETHYLCYCLODECASILOXANE #
Formula C20H60O10Si10, MW 740, CAS# 18772-36-6, Entry# 380233
2,2,4,4,6,6,8,8,10,10,12,12,14,14,16,16,18,18,20,20-ICOSAMETHYLCYCLODECASILOXANE

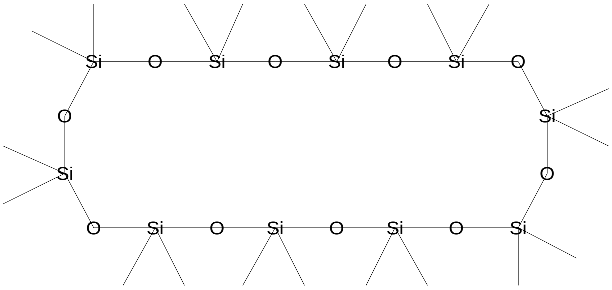

There is no signature data to report.

Library Search Report

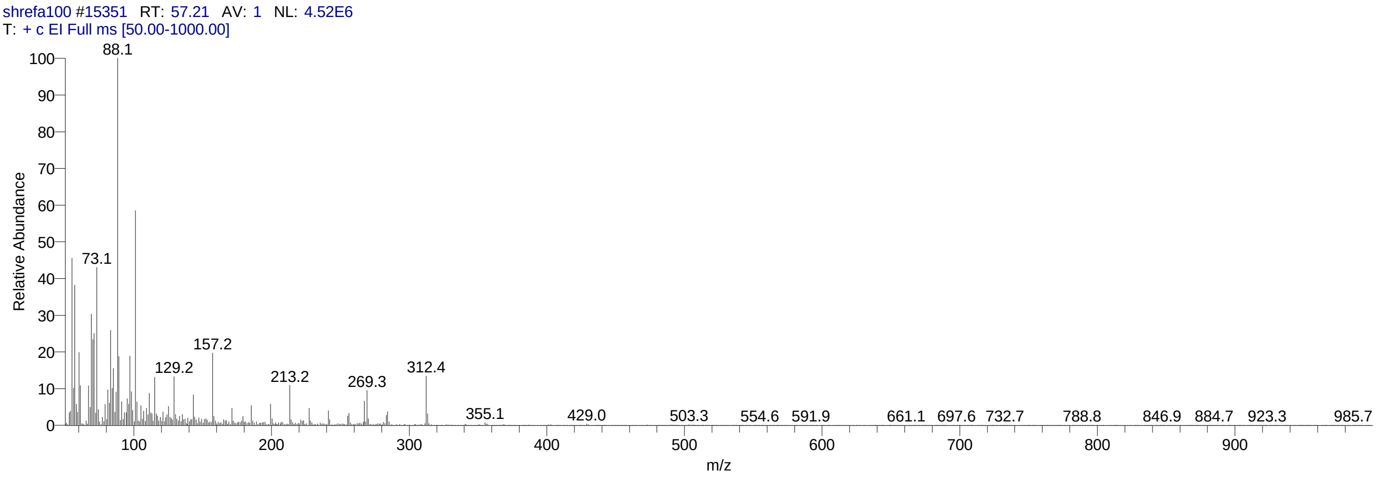

RT	Scan #	Probability	Compound Name	SI	RSI	Cas #	Area	Area %	Library
57.21	15351.000000	69.62	OCTADECANOIC ACID, ETHYL ESTER	829	847	111-61-5	142602558.22	1.75	WileyRegistry8e
57.21	15351.000000	69.62	Octadecanoic acid, ethyl ester	794	823	111-61-5	142602558.22	1.75	replib
57.21	15351.000000	69.62	OCTADECANOIC ACID, ETHYL ESTER	794	822	111-61-5	142602558.22	1.75	WileyRegistry8e
57.21	15351.000000	69.62	OCTADECANOIC ACID, ETHYL ESTER	777	872	111-61-5	142602558.22	1.75	WileyRegistry8e
57.21	15351.000000	69.62	OCTADECANOIC ACID, ETHYL ESTER	772	807	111-61-5	142602558.22	1.75	WileyRegistry8e

Hit Spectrum | Compound Structure

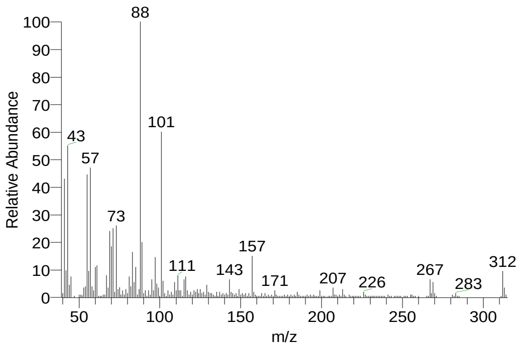

OCTADECANOIC ACID, ETHYL ESTER
Formula C20H40O2, MW 312, CAS# 111-61-5, Entry# 200367
ETHYL OCTADECANOATE

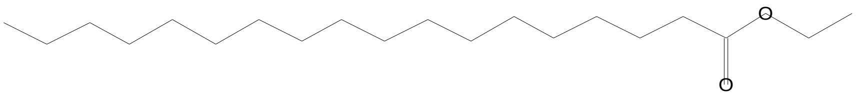

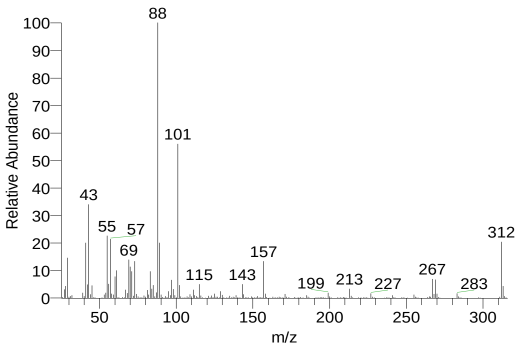

Octadecanoic acid, ethyl ester
Formula C20H40O2, MW 312, CAS# 111-61-5, Entry# 14822
Stearic acid, ethyl ester

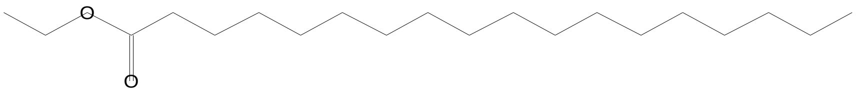

There is no signature data to report.

Library Search Report

Hit Spectrum | Compound Structure

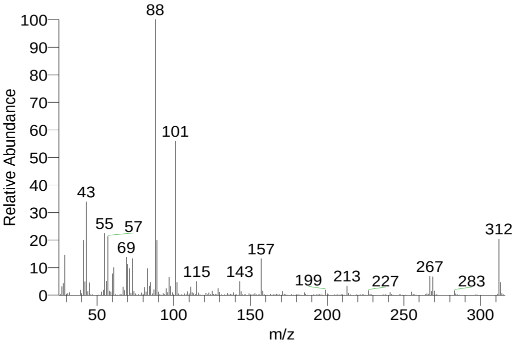

OCTADECANOIC ACID, ETHYL ESTER
Formula C20H40O2, MW 312, CAS# 111-61-5, Entry# 200363
ETHYL OCTADECANOATE

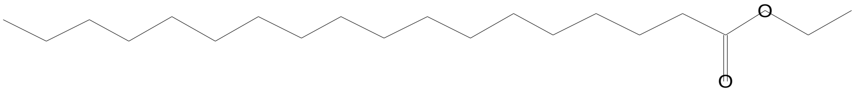

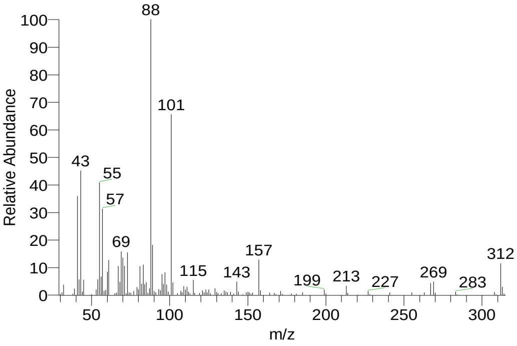

OCTADECANOIC ACID, ETHYL ESTER
Formula C20H40O2, MW 312, CAS# 111-61-5, Entry# 200364
ETHYL OCTADECANOATE

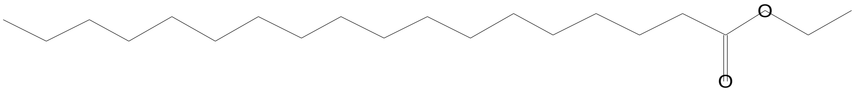

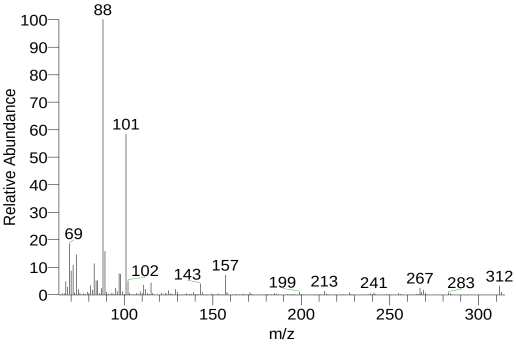

OCTADECANOIC ACID, ETHYL ESTER
Formula C20H40O2, MW 312, CAS# 111-61-5, Entry# 200368
ETHYL OCTADECANOATE

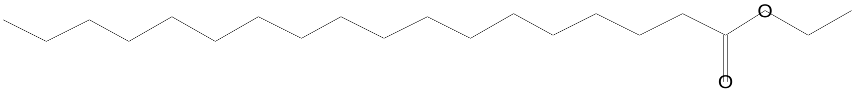

There is no signature data to report.

Library Search Report

shrefa100 #15684 RT: 58.34 AV: 1 NL: 1.30E6
T: + c EI Full ms [50.00-1000.00]

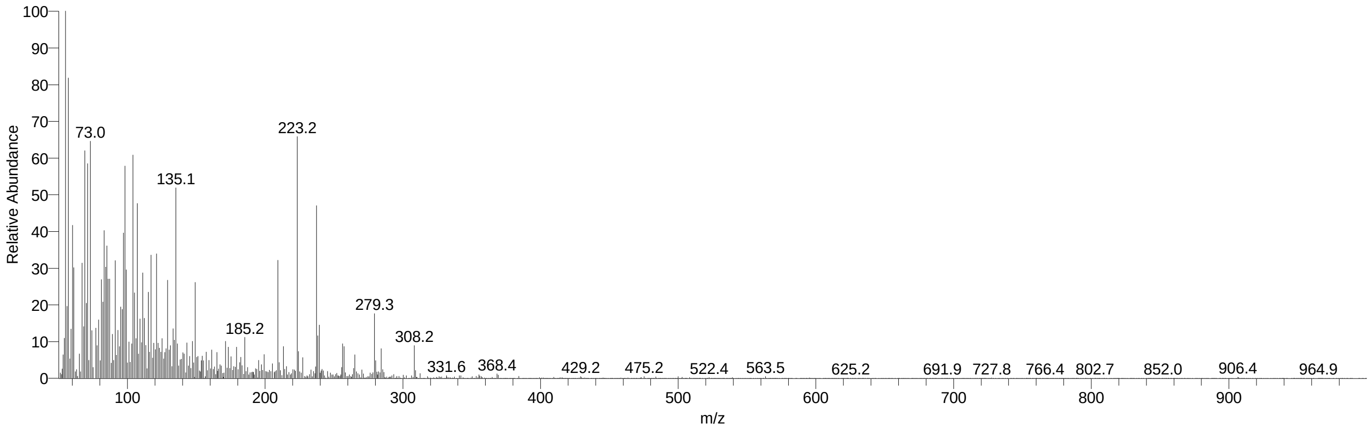

RT	Scan #	Probability	Compound Name	SI	RSI	Cas #	Area	Area %	Library
58.34	15684.000000	31.54	4H-1-BENZOPYRAN-4-ONE, 2-(3,4-DIMETHOXYPHENYL)-3,5-DIHYDROXY-7-METHOXY-	674	680	6068-80-0	62271766.90	0.76	WileyRegistry8e
58.34	15684.000000	21.01	N-methylasimilobine N-oxide	662	676	NA	62271766.90	0.76	mainlib
58.34	15684.000000	5.72	PENTADECANOIC ACID	631	682	1002-84-2	62271766.90	0.76	WileyRegistry8e
58.34	15684.000000	5.28	2-Bromotetradecanoic acid	629	705	10520-81-7	62271766.90	0.76	mainlib
58.34	15684.000000	5.07	4H-1-BENZOPYRAN-4-ONE, 2-(3,4-DIMETHOXYPHENYL)-5,7-DIHYDROXY-	628	635	4712-12-3	62271766.90	0.76	WileyRegistry8e

Hit Spectrum	Compound Structure

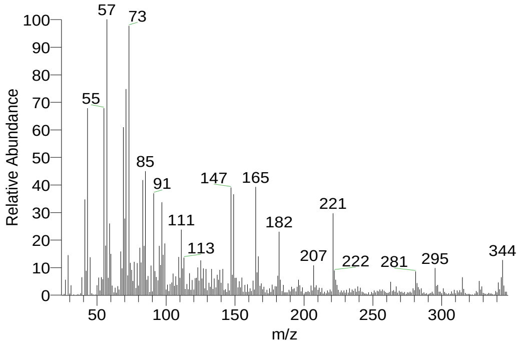

4H-1-BENZOPYRAN-4-ONE, 2-(3,4-DIMETHOXYPHENYL)-3,5-DIHYDROXY-7-METHOXY-
Formula C18H16O7, MW 344, CAS# 6068-80-0, Entry# 224392
3',4',7-TRIMETHYLQUERCETIN

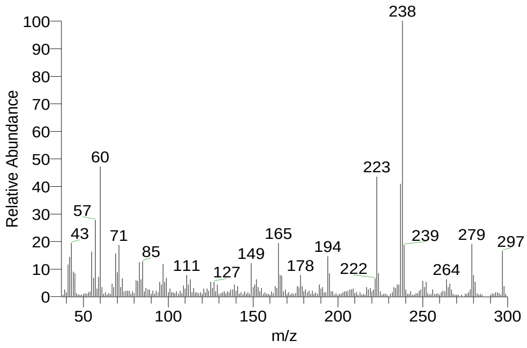

N-methylasimilobine N-oxide
Formula C18H19NO3, MW 297, CAS# NA, Entry# 224778
$:28NMZZOEWNAGKCFR-UHFFFAOYSA-N

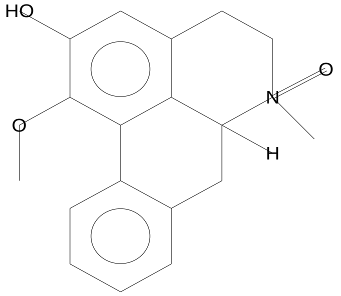

There is no signature data to report.

Library Search Report

Hit Spectrum	Compound Structure

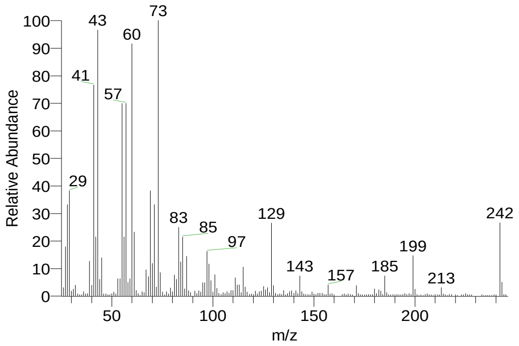

PENTADECANOIC ACID
Formula C15H30O2, MW 242, CAS# 1002-84-2, Entry# 131990
14FA

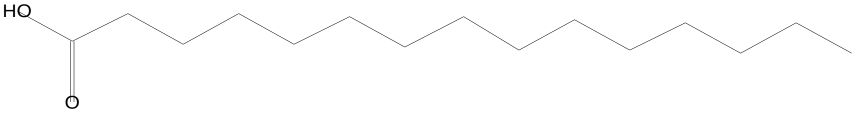

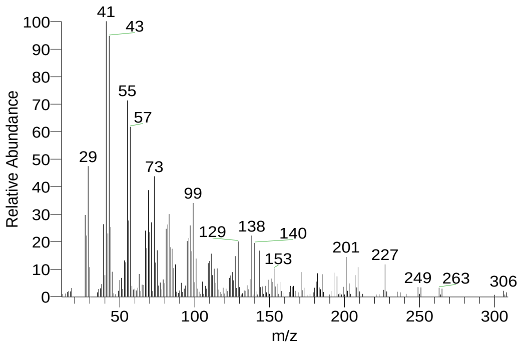

2-Bromotetradecanoic acid
Formula C14H27BrO2, MW 306, CAS# 10520-81-7, Entry# 2713
Tetradecanoic acid, 2-bromo-

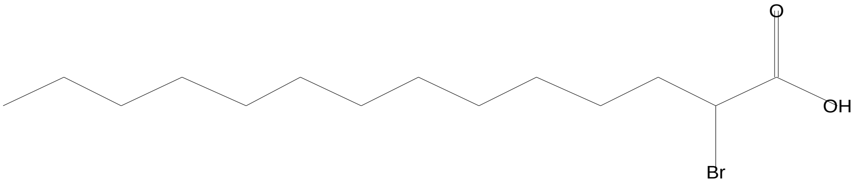

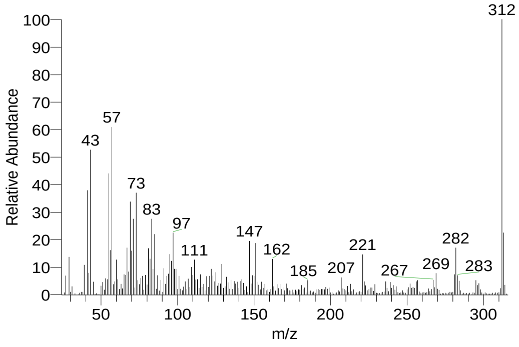

4H-1-BENZOPYRAN-4-ONE, 2-(3,4-DIMETHOXYPHENYL)-5,7-DIHYDROXY-
Formula C17H14O6, MW 314, CAS# 4712-12-3, Entry# 201548
08027209001 FLAVONE 5,7-OH,3',4'-OME

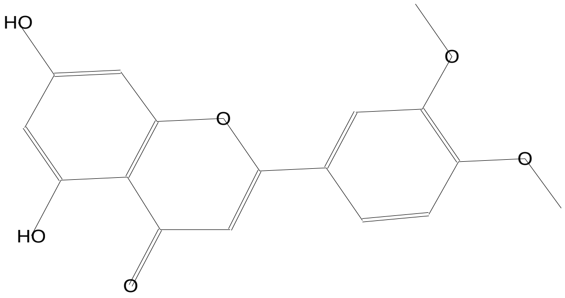

There is no signature data to report.

Library Search Report

shrefa100 #15778 RT: 58.66 AV: 1 NL: 1.33E6
T: + c EI Full ms [50.00-1000.00]

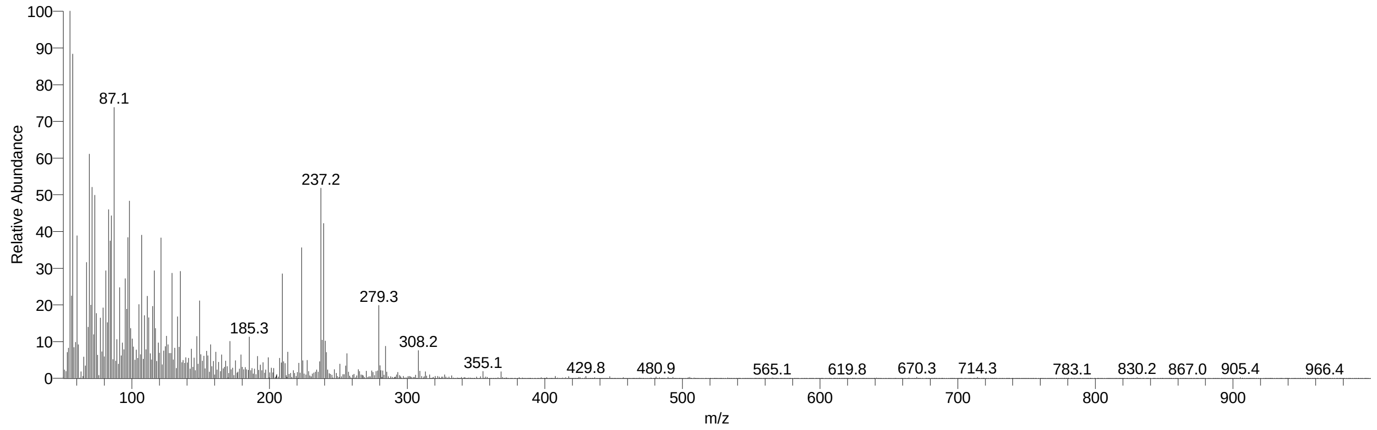

RT	Scan #	Probability	Compound Name	SI	RSI	Cas #	Area	Area %	Library
58.66	15778.000000	28.01	N-methylasimilobine N-oxide	662	680	NA	66416668.68	0.81	mainlib
58.66	15778.000000	26.92	Pyrano[4,3-b]benzopyran-1,9-dione, 5a-methoxy-9a-methyl-3-(1-propenyl)perhydro-	661	676	NA	66416668.68	0.81	mainlib
58.66	15778.000000	26.92	5A-METHOXY-9A-METHYL-3-[1-PROPENYL]DECAHYDRO-1H,9H-PYRANO[4,3-B]CHROMENE-1,9-DIONE	661	676	NA	66416668.68	0.81	WileyRegistry8e
58.66	15778.000000	5.27	4-Hexadecynoic acid, 2-butoxy-, butyl ester	607	680	40924-20-7	66416668.68	0.81	mainlib
58.66	15778.000000	5.27	4-HEXADECYNOIC ACID, 2-BUTOXY-, BUTYL ESTER	607	680	40924-20-7	66416668.68	0.81	WileyRegistry8e

Hit Spectrum | Compound Structure

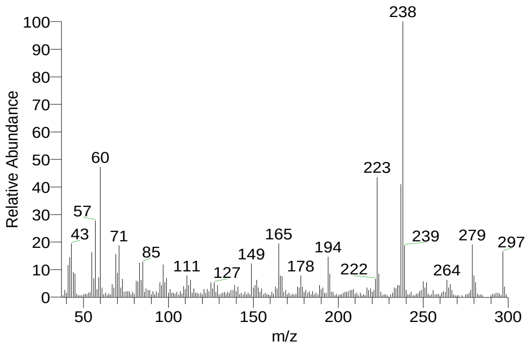

N-methylasimilobine N-oxide
Formula C18H19NO3, MW 297, CAS# NA, Entry# 224778
$:28NMZZOEWNAGKCFR-UHFFFAOYSA-N

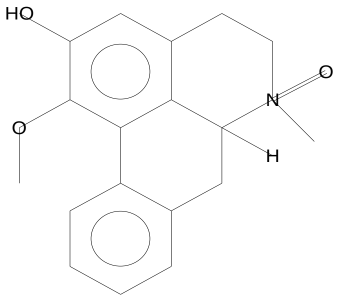

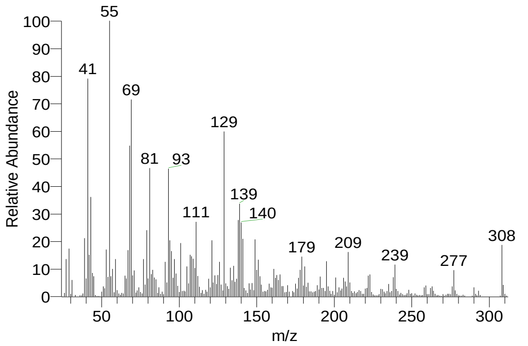

Pyrano[4,3-b]benzopyran-1,9-dione, 5a-methoxy-9a-methyl-3-(1-propenyl)perhydro-
Formula C17H24O5, MW 308, CAS# NA, Entry# 20834
5a-Methoxy-9a-methyl-3-[(1E)-1-propenyl]decahydro-1H,9H-pyrano[4,3-b]chromene-1,9-dione #

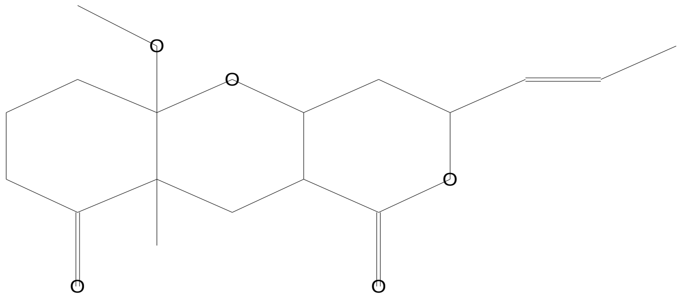

There is no signature data to report.

Library Search Report

Hit Spectrum | Compound Structure

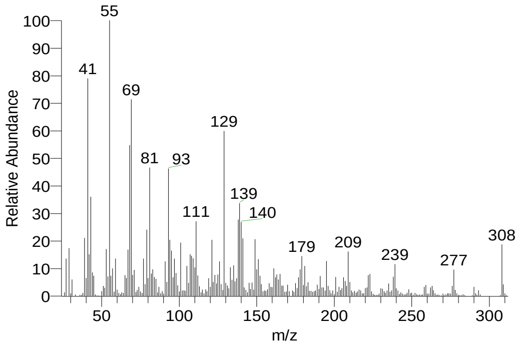

Formula C17H24O5, MW 308, CAS# NA, Entry# 364705

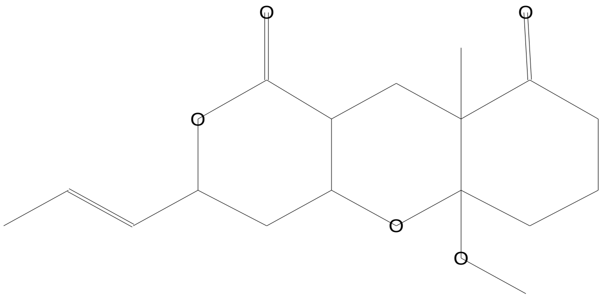

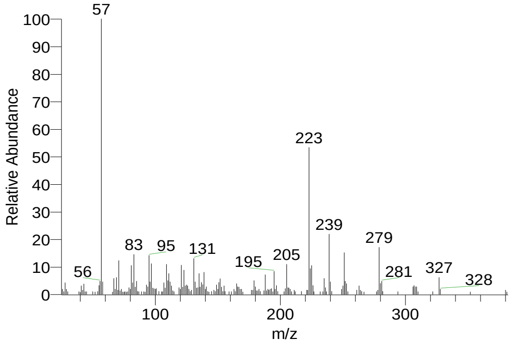

4-Hexadecynoic acid, 2-butoxy-, butyl ester
Formula C24H44O3, MW 380, CAS# 40924-20-7, Entry# 29563
Butyl 2-butoxy-4-hexadecynoate #

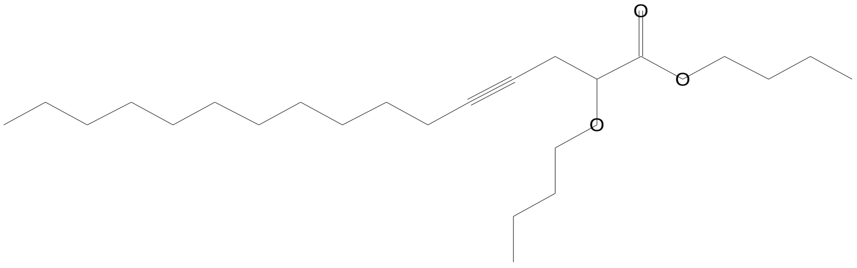

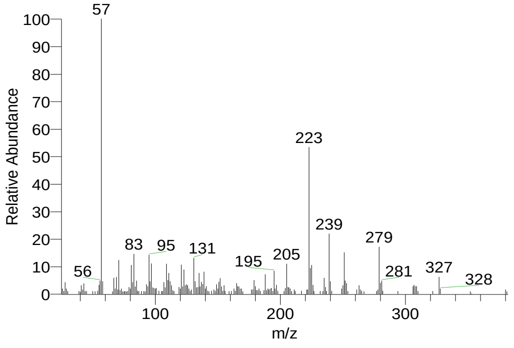

4-HEXADECYNOIC ACID, 2-BUTOXY-, BUTYL ESTER
Formula C24H44O3, MW 380, CAS# 40924-20-7, Entry# 246526
BUTYL 2-BUTOXY-4-HEXADECYNOATE #

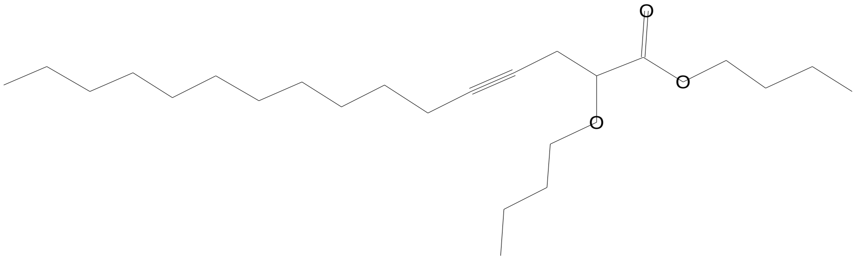

There is no signature data to report.

Library Search Report

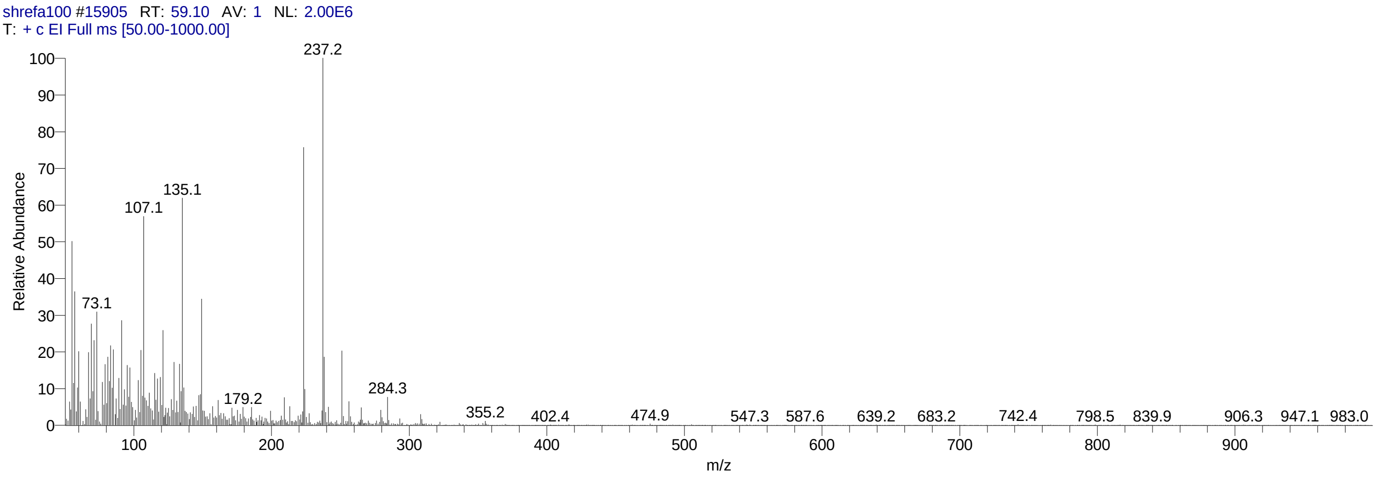

RT	Scan #	Probability	Compound Name	SI	RSI	Cas #	Area	Area %	Library
59.10	15905.000000	44.30	N-methylasimilobine N-oxide	677	686	NA	60949826.27	0.75	mainlib
59.10	15905.000000	9.35	NONYLPHENYL (DI-NONYL)PHENYL AMINE, MIX OF ISOMERS	631	749	NA	60949826.27	0.75	WileyRegistry8e
59.10	15905.000000	3.97	Ethanol, 2-[2-[4-(1,1,3,3-tetramethylbutyl)phenoxy]ethoxy]-	610	710	2315-61-9	60949826.27	0.75	replib
59.10	15905.000000	3.36	4aà,4bá-Gibbane-1à,10á-dicarboxylic acid, 4a-formyl-7-hydroxy-1-methyl-8-methylene-, dimethyl ester	606	612	6980-45-6	60949826.27	0.75	mainlib
59.10	15905.000000	2.64	[2-(5-Hydroxypent-2-enyl)-3-oxocyclopentyl]thioacetic acid, S-t-butyl ester	600	684	68931-53-3	60949826.27	0.75	mainlib

Hit Spectrum

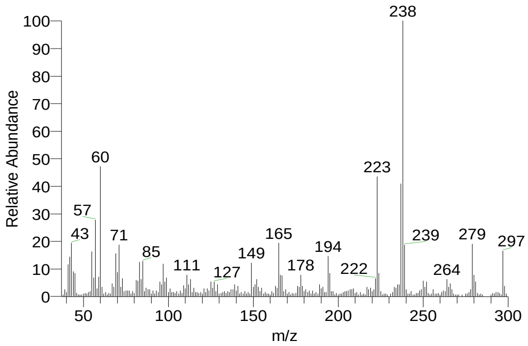

Compound Structure

N-methylasimilobine N-oxide
Formula C18H19NO3, MW 297, CAS# NA, Entry# 224778
$:28NMZZOEWNAGKCFR-UHFFFAOYSA-N

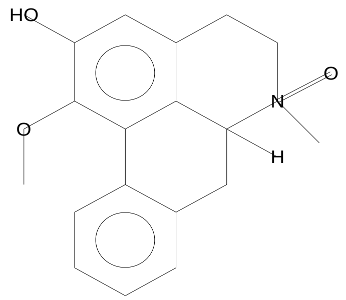

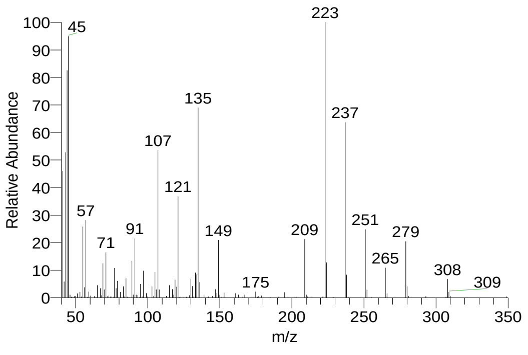

NONYLPHENYL (DI-NONYL)PHENYL AMINE, MIX OF ISOMERS
Formula C19H32O3, MW 308, CAS# NA, Entry# 196733

There is no signature data to report.

Library Search Report

Hit Spectrum	Compound Structure

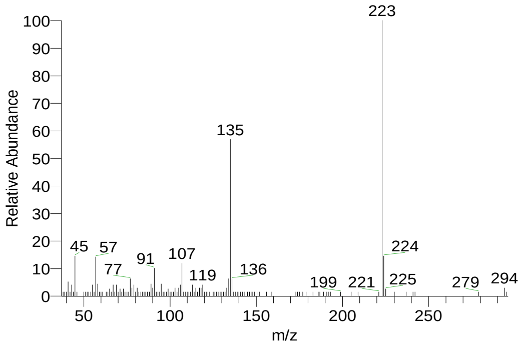

Ethanol, 2-[2-[4-(1,1,3,3-tetramethylbutyl)phenoxy]ethoxy]-
Formula C18H30O3, MW 294, CAS# 2315-61-9, Entry# 34920
2-(2-[4-(1,1,3,3-Tetramethylbutyl)phenoxy]ethoxy)ethanol #

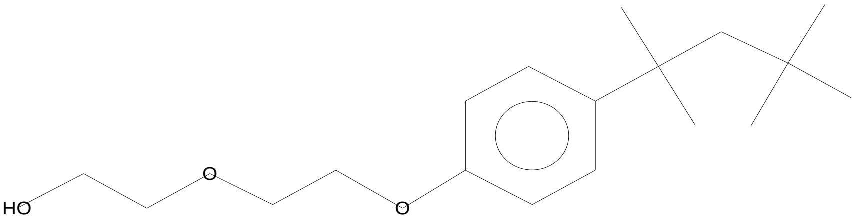

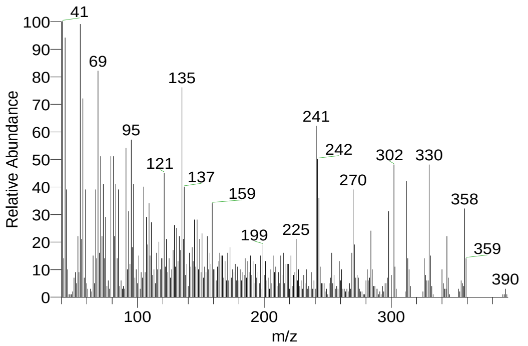

Formula C22H30O6, MW 390, CAS# 6980-45-6, Entry# 2981
Dimethyl 4a-formyl-7-hydroxy-1-methyl-8-methylenegibbane-1,10-dicarboxylate #

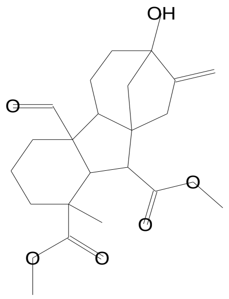

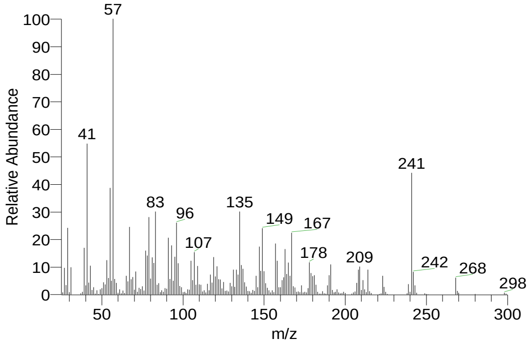

[2-(5-Hydroxypent-2-enyl)-3-oxocyclopentyl]thioacetic acid, S-t-butyl ester
Formula C16H26O3S, MW 298, CAS# 68931-53-3, Entry# 25729
S-(tert-Butyl) (2-[(2Z)-5-hydroxy-2-pentenyl]-3-oxocyclopentyl)ethanethioate #

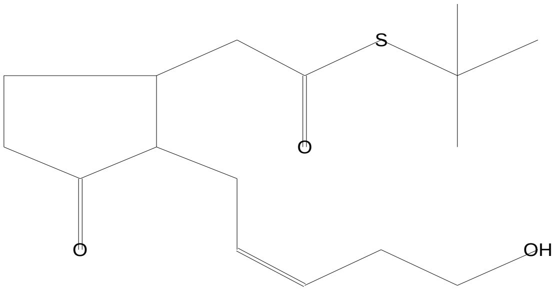

There is no signature data to report.

Library Search Report

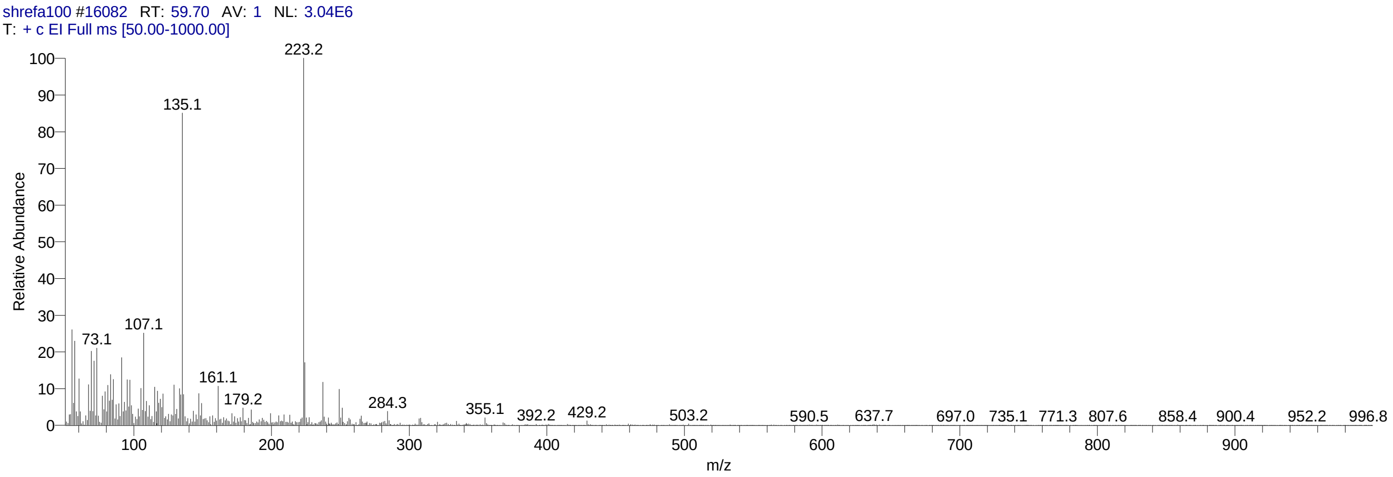

RT	Scan #	Probability	Compound Name	SI	RSI	Cas #	Area	Area %	Library
59.70	16082.000000	29.88	Ethanol, 2-[2-[4-(1,1,3,3-tetramethylbutyl)phenoxy]ethoxy]-	674	807	2315-61-9	66252478.10	0.81	replib
59.70	16082.000000	29.88	Ethanol, 2-[2-[4-(1,1,3,3-tetramethylbutyl)phenoxy]ethoxy]-	653	780	2315-61-9	66252478.10	0.81	mainlib
59.70	16082.000000	29.88	ETHANOL, 2-[2-[4-(1,1,3,3-TETRAMETHYLBUTYL)PHENOXY]ETHOXY]-	650	776	2315-61-9	66252478.10	0.81	WileyRegistry8e
59.70	16082.000000	7.98	9-Octadecenoic acid, (2-phenyl-1,3-dioxolan-4-yl)methyl ester, cis-	642	658	56599-45-2	66252478.10	0.81	mainlib
59.70	16082.000000	7.98	9-OCTADECENOIC ACID, (2-PHENYL-1,3-DIOXOLAN-4-YL)METHYL ESTER, CIS-	642	658	56599-45-2	66252478.10	0.81	WileyRegistry8e

Hit Spectrum | Compound Structure

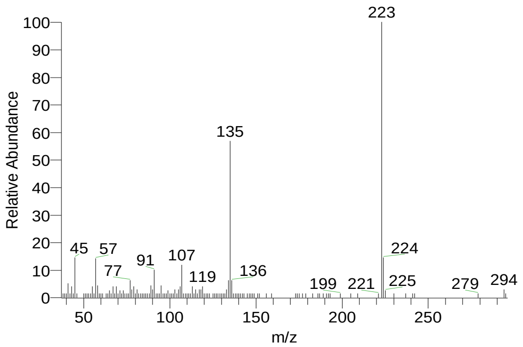

Ethanol, 2-[2-[4-(1,1,3,3-tetramethylbutyl)phenoxy]ethoxy]-
Formula C18H30O3, MW 294, CAS# 2315-61-9, Entry# 34920
2-(2-[4-(1,1,3,3-Tetramethylbutyl)phenoxy]ethoxy)ethanol #

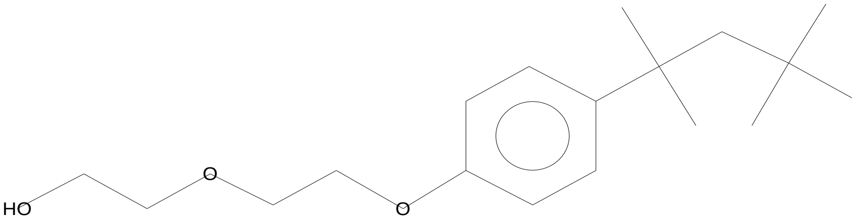

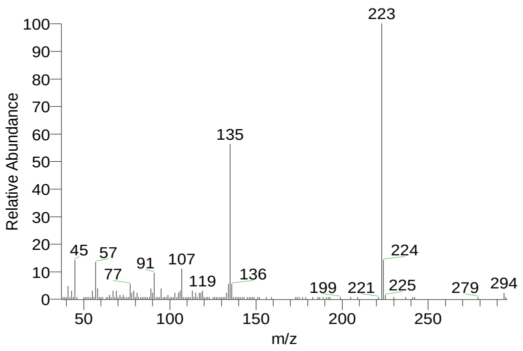

Ethanol, 2-[2-[4-(1,1,3,3-tetramethylbutyl)phenoxy]ethoxy]-
Formula C18H30O3, MW 294, CAS# 2315-61-9, Entry# 217265
2-(2-[4-(1,1,3,3-Tetramethylbutyl)phenoxy]ethoxy)ethanol #

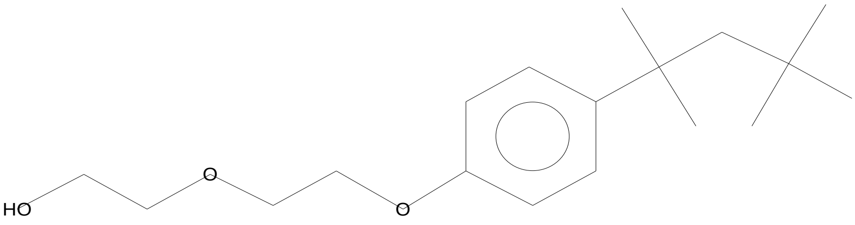

There is no signature data to report.

Library Search Report

Hit Spectrum	Compound Structure

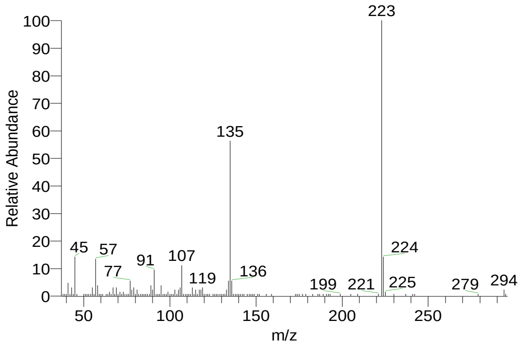

ETHANOL, 2-[2-[4-(1,1,3,3-TETRAMETHYLBUTYL)PHENOXY]ETHOXY]-
Formula C18H30O3, MW 294, CAS# 2315-61-9, Entry# 184040
2-(2-[4-(1,1,3,3-TETRAMETHYLBUTYL)PHENOXY]ETHOXY)ETHANOL #

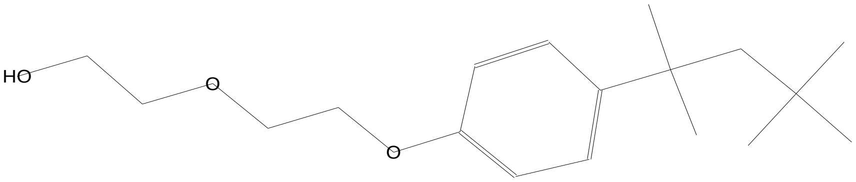

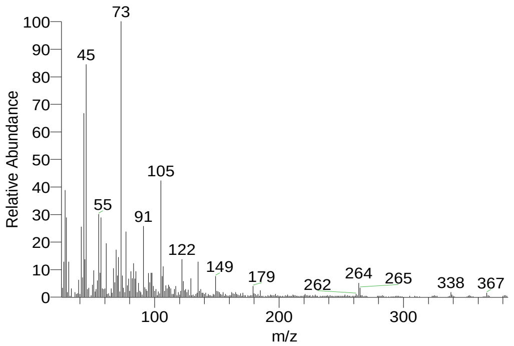

9-Octadecenoic acid, (2-phenyl-1,3-dioxolan-4-yl)methyl ester, cis-
Formula C28H44O4, MW 444, CAS# 56599-45-2, Entry# 44507
(2-Phenyl-1,3-dioxolan-4-yl)methyl 9-octadecenoate, cis-

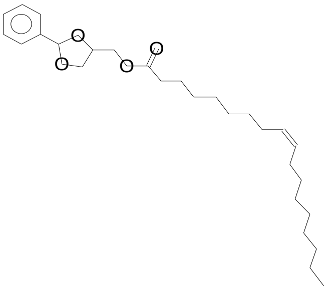

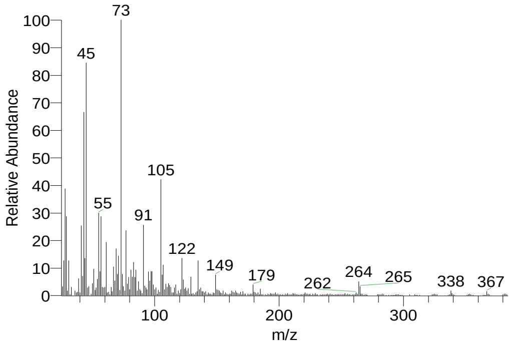

9-OCTADECENOIC ACID, (2-PHENYL-1,3-DIOXOLAN-4-YL)METHYL ESTER, CIS-
Formula C28H44O4, MW 444, CAS# 56599-45-2, Entry# 272719
(2-PHENYL-1,3-DIOXOLAN-4-YL)METHYL (9E)-9-OCTADECENOATE #

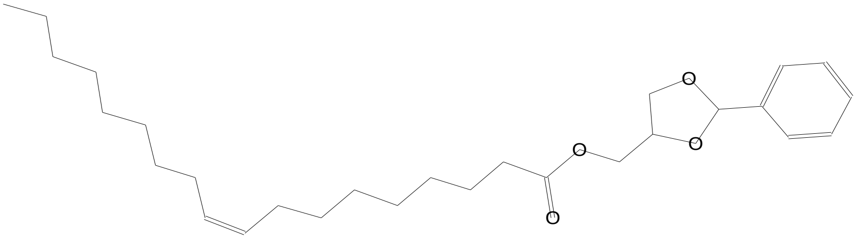

There is no signature data to report.

Library Search Report

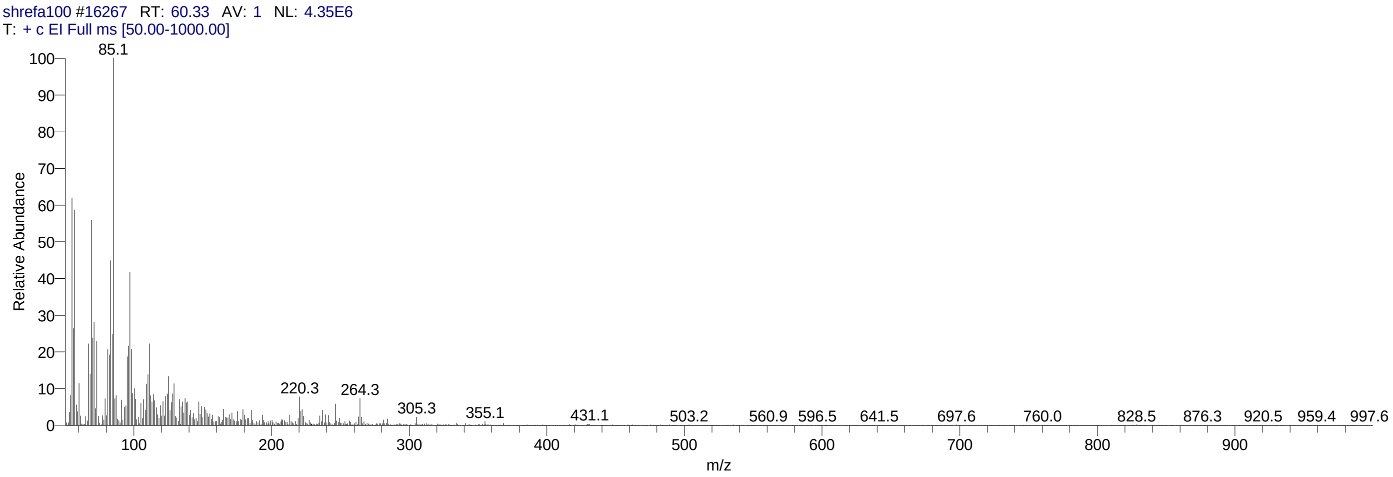

RT	Scan #	Probability	Compound Name	SI	RSI	Cas #	Area	Area %	Library
60.33	16267.000000	9.92	Octadecanoic acid, 4-hydroxy-, methyl ester	805	833	2420-38-4	167466973.88	2.05	mainlib
60.33	16267.000000	9.92	OCTADECANOIC ACID, 4-HYDROXY-, METHYL ESTER	803	830	2420-38-4	167466973.88	2.05	WileyRegistry8e
60.33	16267.000000	6.01	2(3H)-Furanone, dihydro-5-tetradecyl-	790	829	502-26-1	167466973.88	2.05	mainlib
60.33	16267.000000	6.01	2(3H)-FURANONE, DIHYDRO-5-TETRADECYL-	790	829	502-26-1	167466973.88	2.05	WileyRegistry8e
60.33	16267.000000	5.54	Oleic Acid	788	808	112-80-1	167466973.88	2.05	replib

Hit Spectrum — Compound Structure

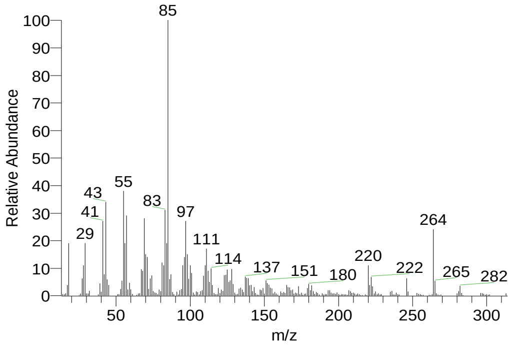

Octadecanoic acid, 4-hydroxy-, methyl ester
Formula C19H38O3, MW 314, CAS# 2420-38-4, Entry# 59666
Methyl 4-hydroxyoctadecanoate #

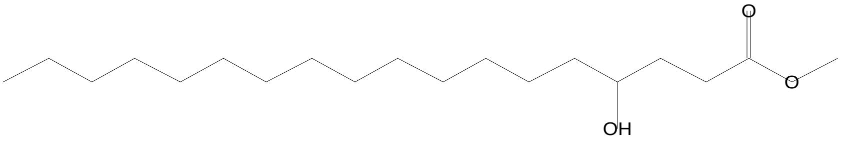

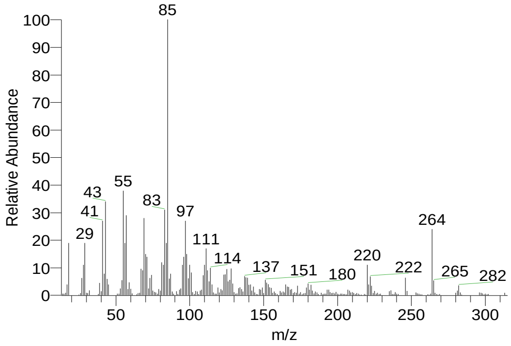

OCTADECANOIC ACID, 4-HYDROXY-, METHYL ESTER
Formula C19H38O3, MW 314, CAS# 2420-38-4, Entry# 201909
METHYL 4-HYDROXYOCTADECANOATE

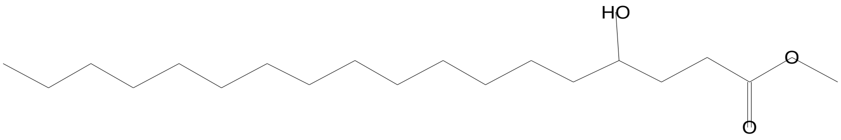

There is no signature data to report.

Library Search Report

Hit Spectrum | Compound Structure

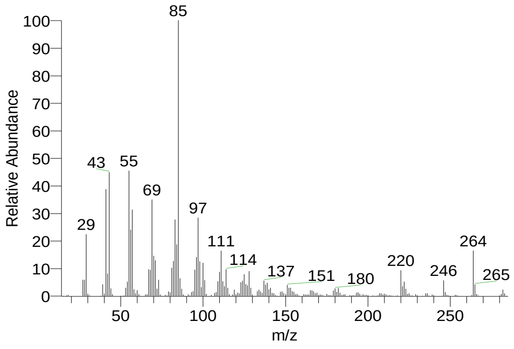

2(3H)-Furanone, dihydro-5-tetradecyl-
Formula C18H34O2, MW 282, CAS# 502-26-1, Entry# 59669
ç-Stearolactone

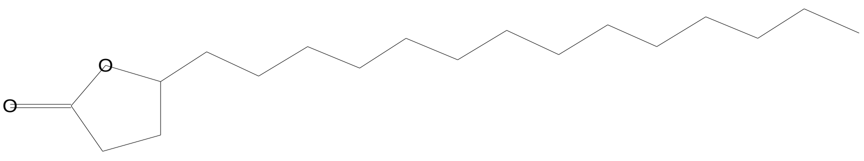

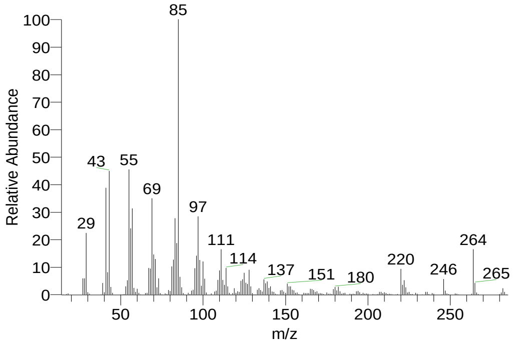

2(3H)-FURANONE, DIHYDRO-5-TETRADECYL-
Formula C18H34O2, MW 282, CAS# 502-26-1, Entry# 172918
5-TETRADECYLDIHYDRO-2(3H)-FURANONE #

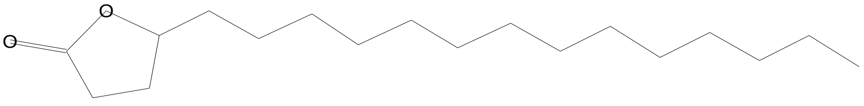

SI 788, RSI 808, replib, Entry# 5762, CAS# 112-80-1, Oleic Acid

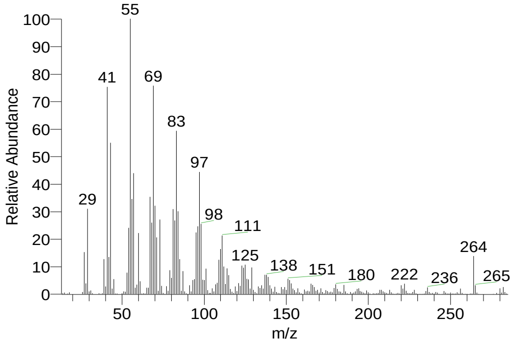

Oleic Acid
Formula C18H34O2, MW 282, CAS# 112-80-1, Entry# 5762
9-Octadecenoic acid (Z)-

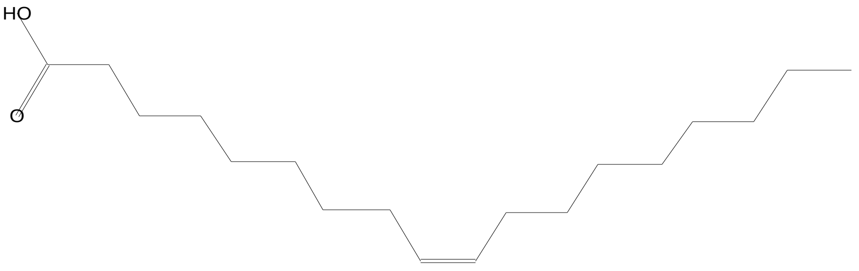

There is no signature data to report.

Library Search Report

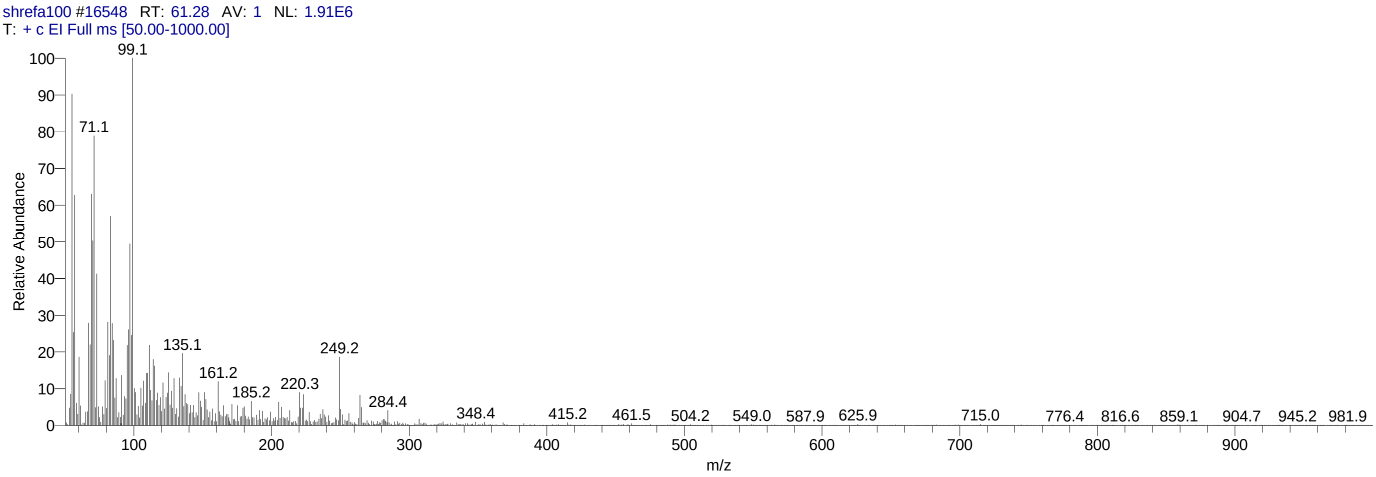

RT	Scan #	Probability	Compound Name	SI	RSI	Cas #	Area	Area %	Library
61.28	16548.000000	8.48	9-Octadecenoic acid (Z)-, tetradecyl ester	738	746	22393-85-7	97464535.08	1.19	mainlib
61.28	16548.000000	7.50	Octadecanal, 2-bromo-	735	744	56599-95-2	97464535.08	1.19	mainlib
61.28	16548.000000	7.50	OCTADECANAL, 2-BROMO-	732	741	56599-95-2	97464535.08	1.19	WileyRegistry8e
61.28	16548.000000	6.33	HEPTADECENE-(8)-CARBONIC ACID-(1)	731	773	NA	97464535.08	1.19	WileyRegistry8e
61.28	16548.000000	8.48	9-OCTADECENOIC ACID (Z)-, TETRADECYL ESTER	724	740	22393-85-7	97464535.08	1.19	WileyRegistry8e

Hit Spectrum	Compound Structure

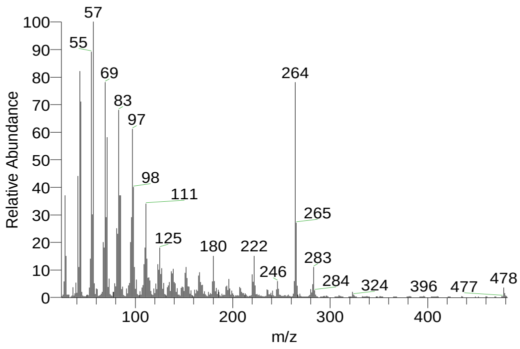

9-Octadecenoic acid (Z)-, tetradecyl ester
Formula C32H62O2, MW 478, CAS# 22393-85-7, Entry# 26632
Oleic acid, tetradecyl ester

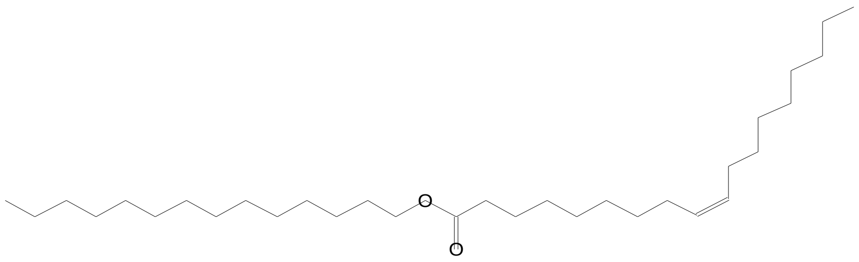

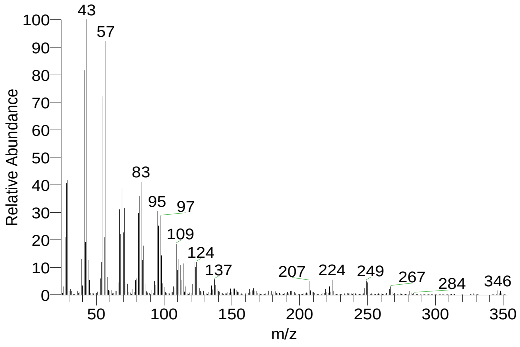

Octadecanal, 2-bromo-
Formula C18H35BrO, MW 346, CAS# 56599-95-2, Entry# 8205
2-Bromooctadecanal #

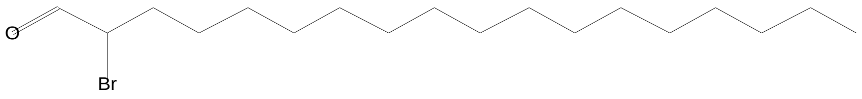

There is no signature data to report.

Library Search Report

Hit Spectrum | Compound Structure

OCTADECANAL, 2-BROMO-
Formula C18H35BrO, MW 346, CAS# 56599-95-2, Entry# 225777
2-BROMOOCTADECANAL

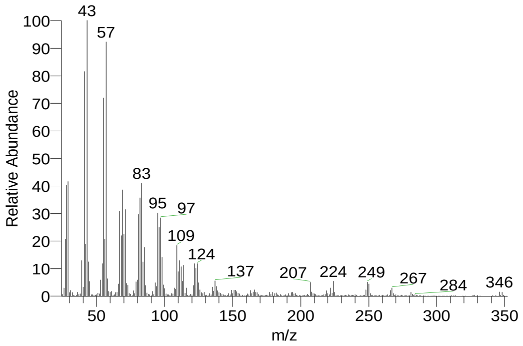

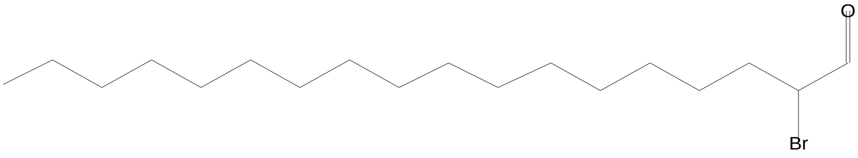

HEPTADECENE-(8)-CARBONIC ACID-(1)
Formula C18H34O2, MW 282, CAS# NA, Entry# 172900

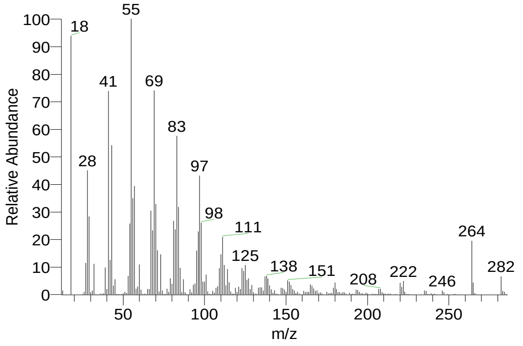

9-OCTADECENOIC ACID (Z)-, TETRADECYL ESTER
Formula C32H62O2, MW 478, CAS# 22393-85-7, Entry# 281496
TETRADECYL (9Z)-9-OCTADECENOATE #

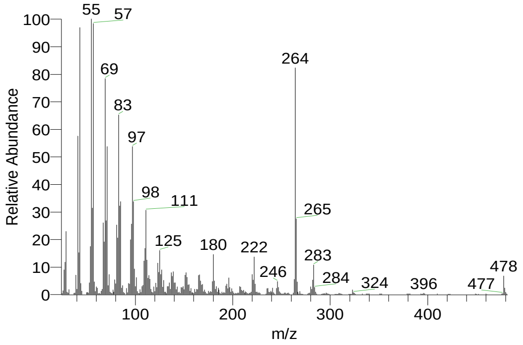

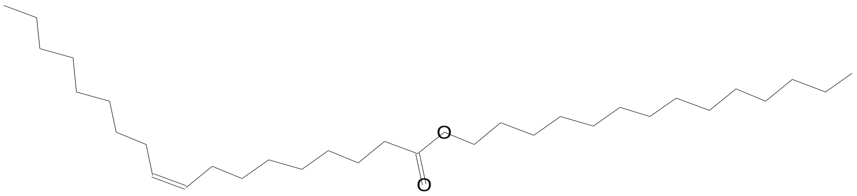

There is no signature data to report.

Library Search Report

shrefa100 #16793 RT: 62.12 AV: 1 NL: 6.42E6
T: + c EI Full ms [50.00-1000.00]

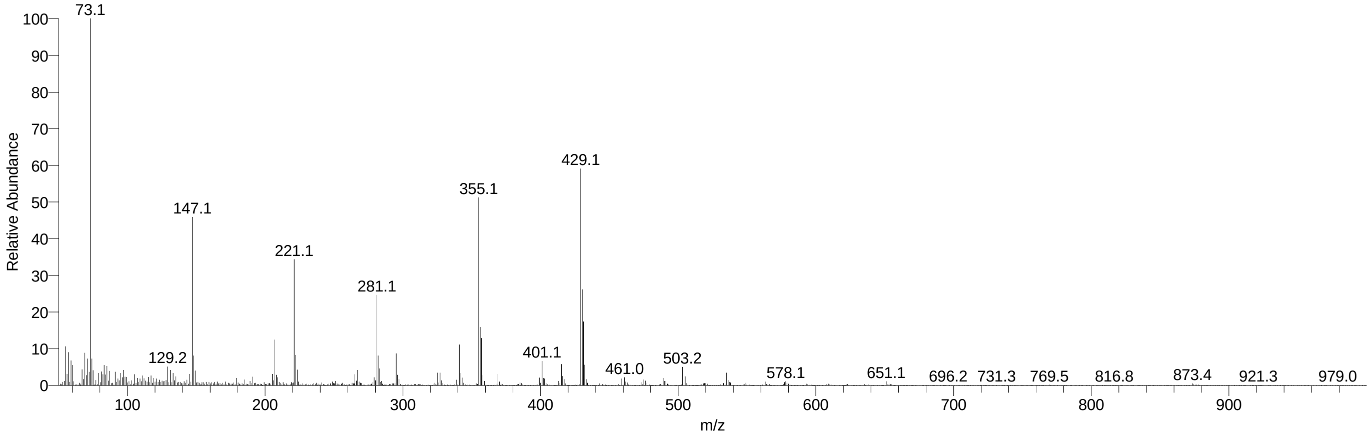

RT	Scan #	Probability	Compound Name	SI	RSI	Cas #	Area	Area %	Library
62.12	16793.000000	29.22	Cyclononasiloxane, octadecamethyl-	774	887	556-71-8	175568569.20	2.15	replib
62.12	16793.000000	21.79	SILIKONFETT SE30 (GREVELS)	766	814	NA	175568569.20	2.15	WileyRegistry8e
62.12	16793.000000	21.79	SILICONE OIL	766	813	NA	175568569.20	2.15	WileyRegistry8e
62.12	16793.000000	19.26	1H-PURIN-6-AMINE, [(2-FLUOROPHENYL)METHYL]-	763	816	74421-44-6	175568569.20	2.15	WileyRegistry8e
62.12	16793.000000	29.22	Cyclononasiloxane, octadecamethyl-	759	872	556-71-8	175568569.20	2.15	mainlib

Hit Spectrum	Compound Structure

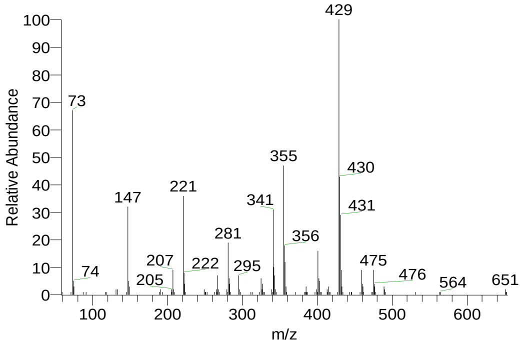

Cyclononasiloxane, octadecamethyl-
Formula C18H54O9Si9, MW 666, CAS# 556-71-8, Entry# 39116
Octadeamethyl-cyclononasiloxane

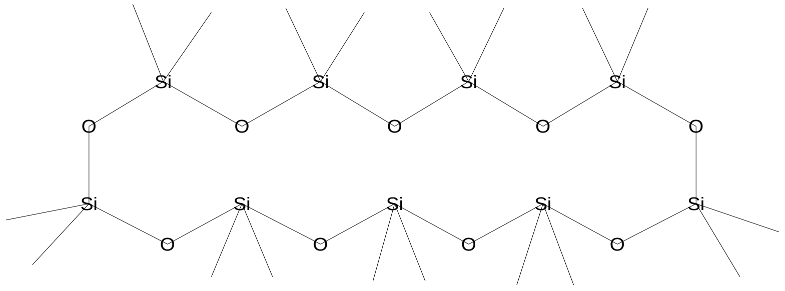

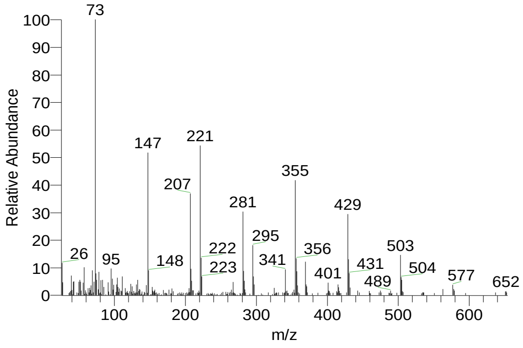

SILIKONFETT SE30 (GREVELS)
Formula , MW 0, CAS# NA, Entry# 392776

There is no signature data to report.

Library Search Report

Hit Spectrum

Compound Structure

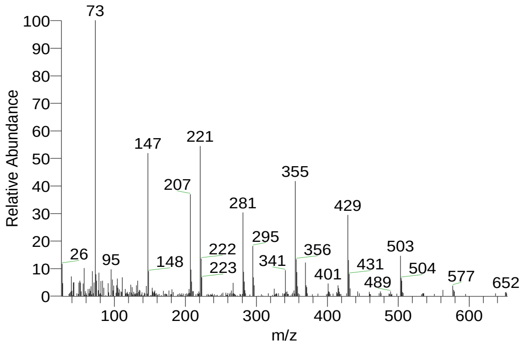

SILICONE OIL
Formula , MW 0, CAS# NA, Entry# 305490
SILIKONFETT SE30 (GREVELS)

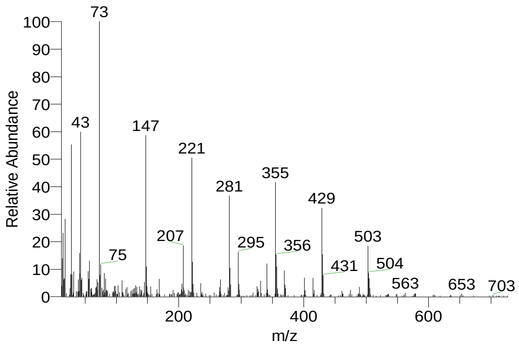

1H-PURIN-6-AMINE, [(2-FLUOROPHENYL)METHYL]-
Formula C12H10FN5, MW 243, CAS# 74421-44-6, Entry# 132518

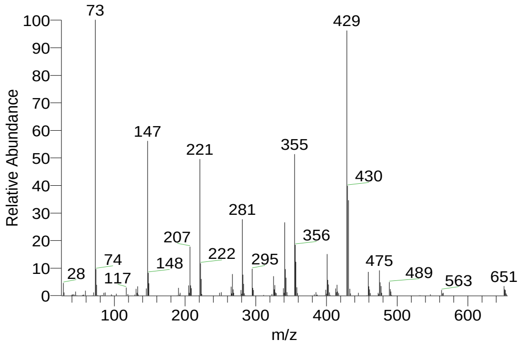

Cyclononasiloxane, octadecamethyl-
Formula C18H54O9Si9, MW 666, CAS# 556-71-8, Entry# 48481
Octadeamethyl-cyclononasiloxane

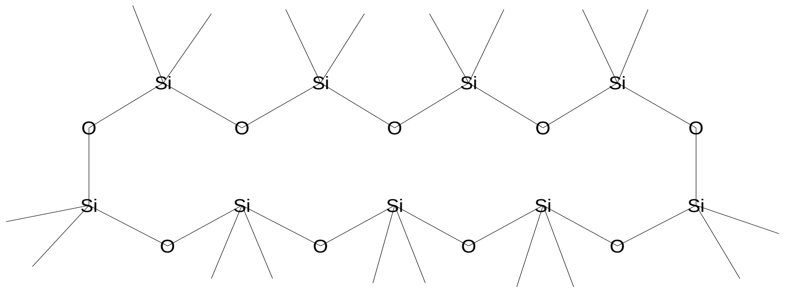

There is no signature data to report.

Library Search Report

shrefa100 #17017 RT: 62.88 AV: 1 NL: 5.47E6
T: + c EI Full ms [50.00-1000.00]

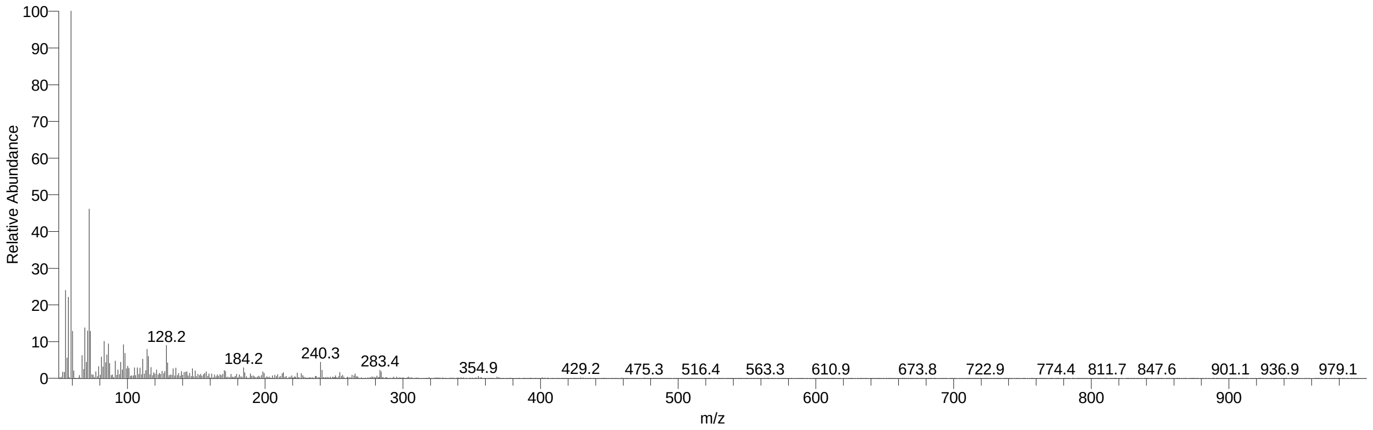

RT	Scan #	Probability	Compound Name	SI	RSI	Cas #	Area	Area %	Library
62.88	17017.000000	15.09	ISOCHIAPIN B	703	709	NA	66329220.94	0.81	WileyRegistry8e
62.88	17017.000000	15.09	ISOCHIAPIN B %2<	703	709	NA	66329220.94	0.81	WileyRegistry8e
62.88	17017.000000	10.65	18,19-Secoyohimban-19-oic acid, 16,17,20,21-tetradehydro-16-(hydroxymethyl)-, methyl ester, (15á,16E)-	693	719	5523-49-9	66329220.94	0.81	mainlib
62.88	17017.000000	10.65	18,19-SECOYOHIMBAN-19-OIC ACID, 16,17,20,21-TETRADEHYDRO-16-(HYDROXYMETHYL)-, METHYL ESTER, (15á,16E)-	692	718	5523-49-9	66329220.94	0.81	WileyRegistry8e
62.88	17017.000000	9.00	4H-1-BENZOPYRAN-4-ONE, 2-(3,4-DIMETHOXYPHENYL)-3,5-DIHYDROXY-7-METHOXY-	689	694	6068-80-0	66329220.94	0.81	WileyRegistry8e

Hit Spectrum | Compound Structure

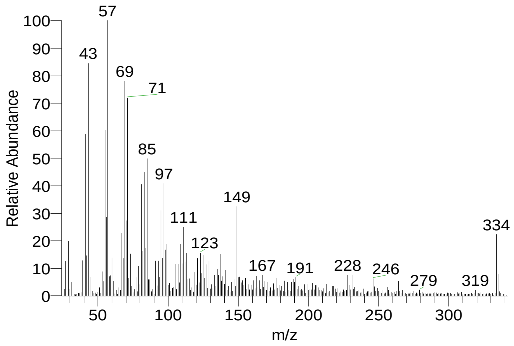

ISOCHIAPIN B
Formula C19H22O6, MW 346, CAS# NA, Entry# 225807

There is no signature data to report.

Library Search Report

Hit Spectrum | Compound Structure

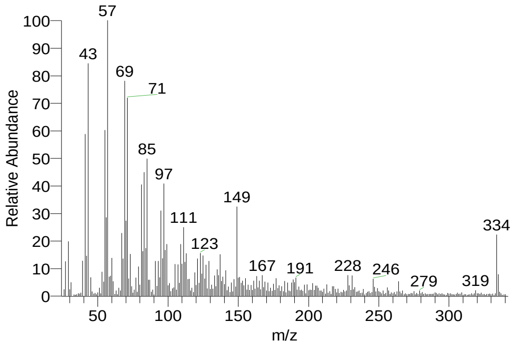

ISOCHIAPIN B %2<
Formula C19H26O6, MW 350, CAS# NA, Entry# 228500

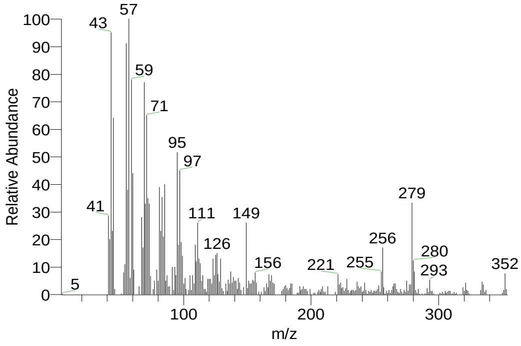

Formula C21H24N2O3, MW 352, CAS# 5523-49-9, Entry# 25985

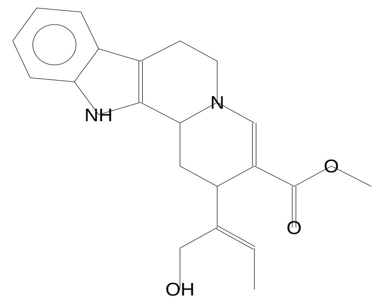

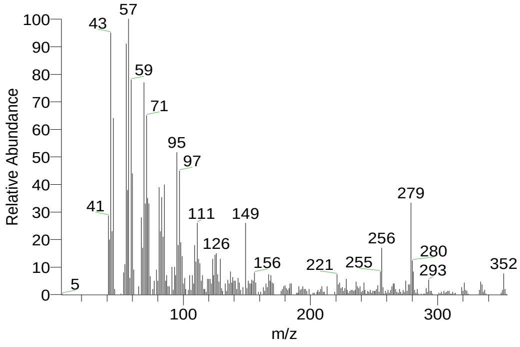

Formula C21H24N2O3, MW 352, CAS# 5523-49-9, Entry# 229989

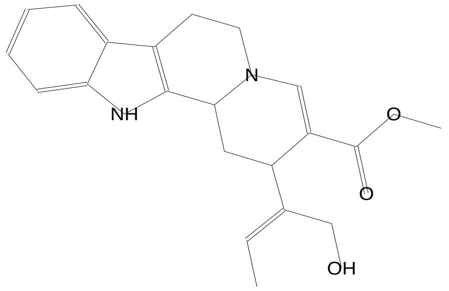

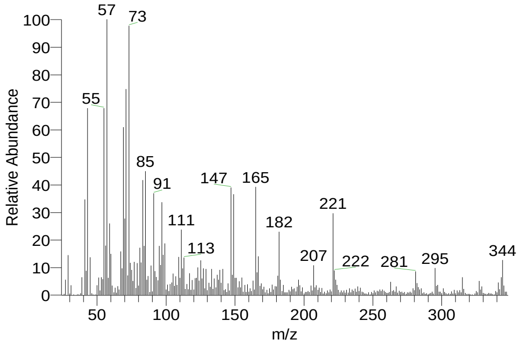

4H-1-BENZOPYRAN-4-ONE, 2-(3,4-DIMETHOXYPHENYL)-3,5-DIHYDROXY-7-METHOXY-
Formula C18H16O7, MW 344, CAS# 6068-80-0, Entry# 224392
3',4',7-TRIMETHYLQUERCETIN

There is no signature data to report.

Library Search Report

shrefa100 #17166 RT: 63.38 AV: 1 NL: 1.50E6
T: + c EI Full ms [50.00-1000.00]

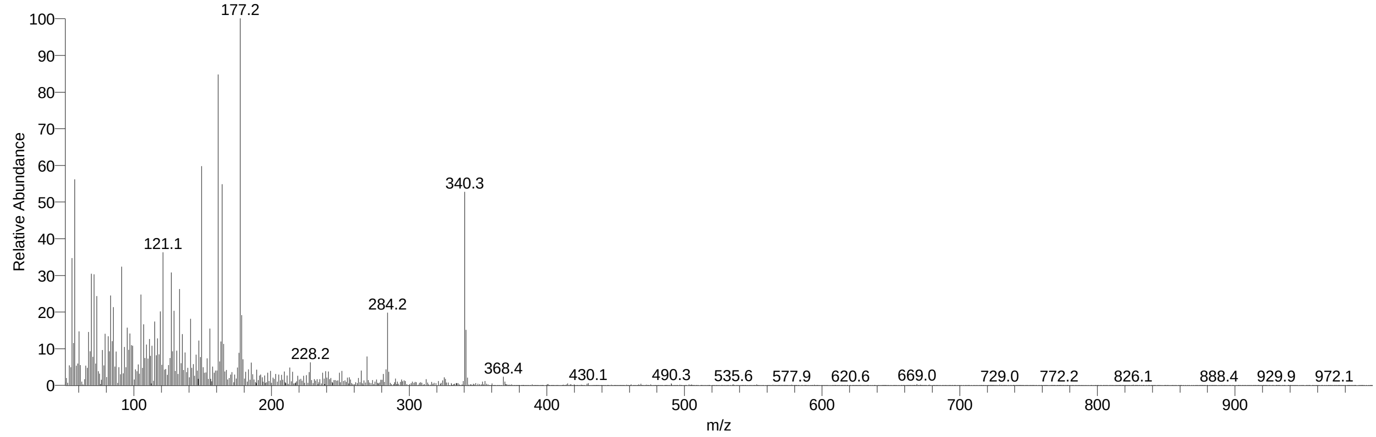

RT	Scan #	Probability	Compound Name	SI	RSI	Cas #	Area	Area %	Library
63.38	17166.000000	80.11	Phenol, 2,2'-methylenebis[6-(1,1-dimethylethyl)-4-methyl-	753	797	119-47-1	55642168.56	0.68	mainlib
63.38	17166.000000	80.11	Phenol, 2,2'-methylenebis[6-(1,1-dimethylethyl)-4-methyl-	750	780	119-47-1	55642168.56	0.68	replib
63.38	17166.000000	80.11	Phenol, 2,2'-methylenebis[6-(1,1-dimethylethyl)-4-methyl-	733	842	119-47-1	55642168.56	0.68	replib
63.38	17166.000000	10.91	2,2'-Methylenebis(6-tert-butyl-4 methylphenol), acetate	680	697	41620-33-1	55642168.56	0.68	mainlib
63.38	17166.000000	80.11	Phenol, 2,2'-methylenebis[6-(1,1-dimethylethyl)-4-methyl-	654	915	119-47-1	55642168.56	0.68	replib

Hit Spectrum | Compound Structure

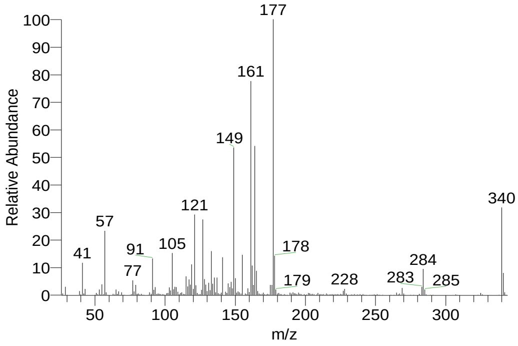

Phenol, 2,2'-methylenebis[6-(1,1-dimethylethyl)-4-methyl-
Formula C23H32O2, MW 340, CAS# 119-47-1, Entry# 181630
p-Cresol, 2,2'-methylenebis[6-tert-butyl-

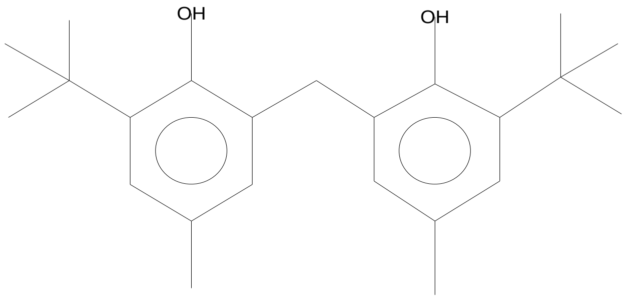

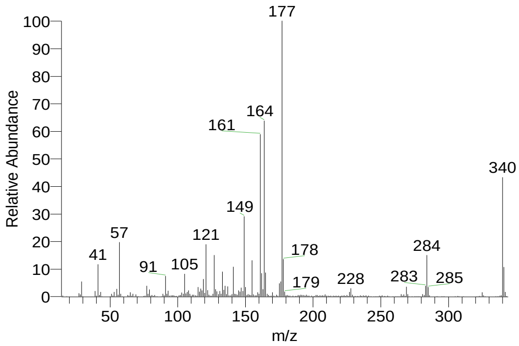

Phenol, 2,2'-methylenebis[6-(1,1-dimethylethyl)-4-methyl-
Formula C23H32O2, MW 340, CAS# 119-47-1, Entry# 30916
p-Cresol, 2,2'-methylenebis[6-tert-butyl-

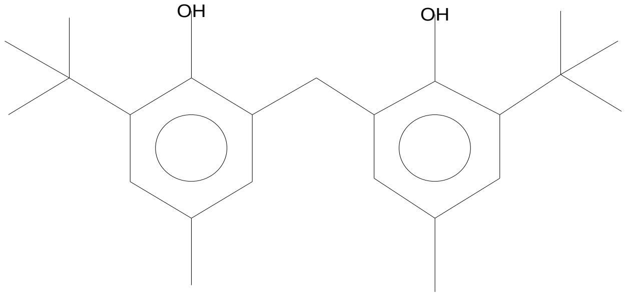

There is no signature data to report.

Library Search Report

Hit Spectrum	Compound Structure

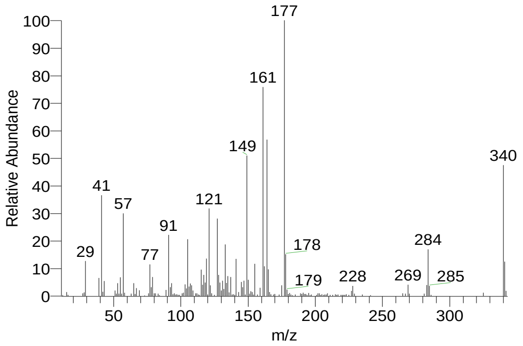

Phenol, 2,2'-methylenebis[6-(1,1-dimethylethyl)-4-methyl-
Formula C23H32O2, MW 340, CAS# 119-47-1, Entry# 30906
p-Cresol, 2,2'-methylenebis[6-tert-butyl-

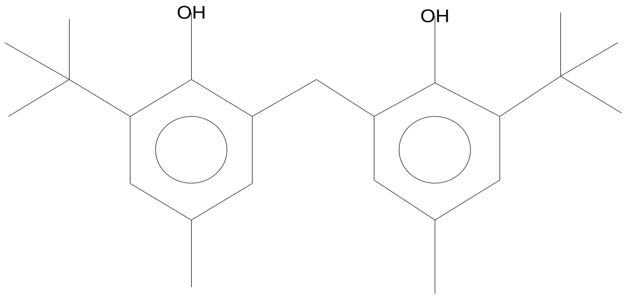

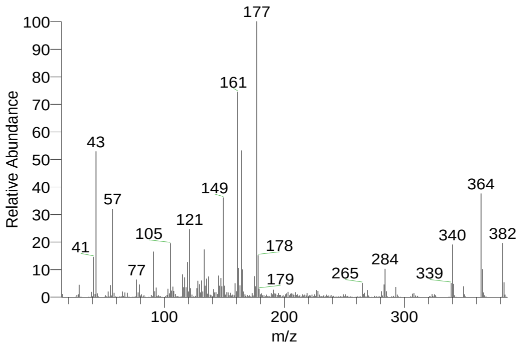

2,2'-Methylenebis(6-tert-butyl-4 methylphenol), acetate
Formula C25H34O3, MW 382, CAS# 41620-33-1, Entry# 181629
$:28NJCYMDOCYOTTMU-UHFFFAOYSA-N

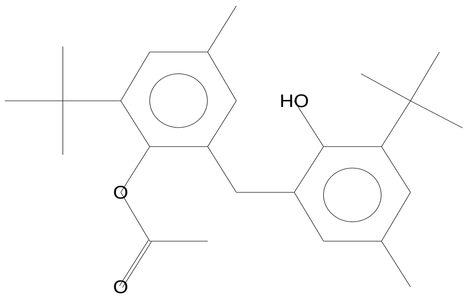

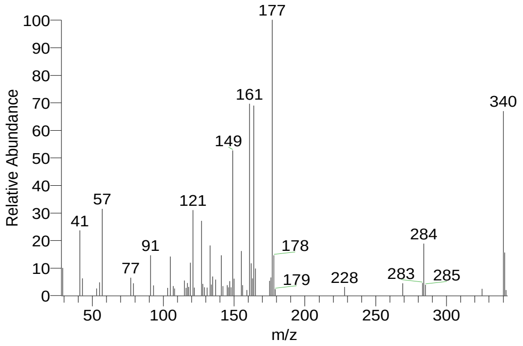

Phenol, 2,2'-methylenebis[6-(1,1-dimethylethyl)-4-methyl-
Formula C23H32O2, MW 340, CAS# 119-47-1, Entry# 30907
p-Cresol, 2,2'-methylenebis[6-tert-butyl-

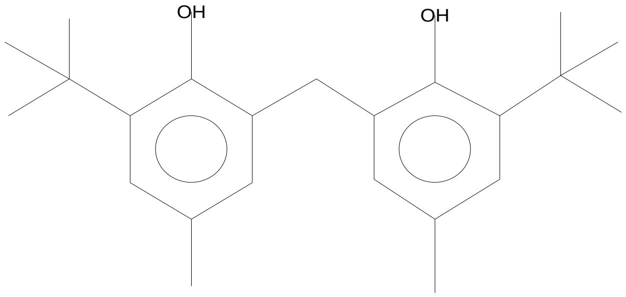

There is no signature data to report.

Library Search Report

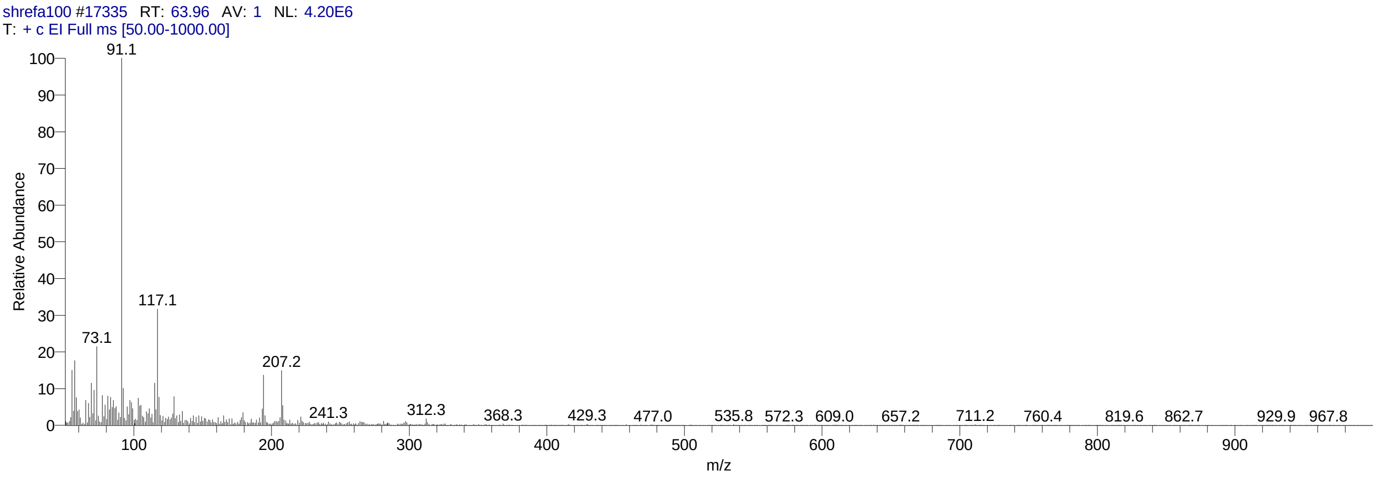

RT	Scan #	Probability	Compound Name	SI	RSI	Cas #	Area	Area %	Library
63.96	17335.000000	18.11	TRISTRIMETHYLSILYL ETHER DERIVATIVE OF 1,25-DIHYDROXYVITAMIN D2	705	732	NA	71289780.40	0.87	WileyRegistry8e
63.96	17335.000000	14.59	4H-1-BENZOPYRAN-4-ONE, 2-(3,4-DIMETHOXYPHENYL)-3,5-DIHYDROXY-7-METHOXY-	700	706	6068-80-0	71289780.40	0.87	WileyRegistry8e
63.96	17335.000000	13.46	4-Hexyl-1-(7-methoxycarbonylheptyl)bicyclo[4.4.0]deca-2,5,7-triene	698	738	NA	71289780.40	0.87	mainlib
63.96	17335.000000	11.37	9-OCTADECENOIC ACID, (2-PHENYL-1,3-DIOXOLAN-4-YL)METHYL ESTER, CIS-	694	712	56599-45-2	71289780.40	0.87	WileyRegistry8e
63.96	17335.000000	11.37	9-Octadecenoic acid, (2-phenyl-1,3-dioxolan-4-yl)methyl ester, cis-	693	712	56599-45-2	71289780.40	0.87	mainlib

Hit Spectrum	Compound Structure

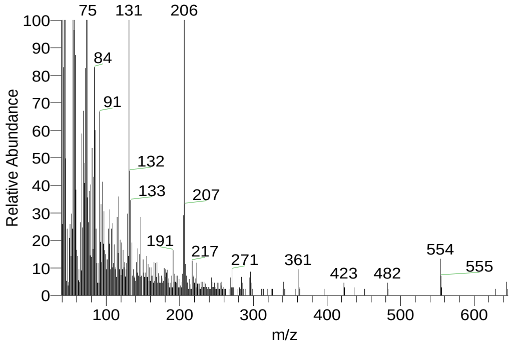

TRISTRIMETHYLSILYL ETHER DERIVATIVE OF 1,25-DIHYDROXYVITAMIN D2
Formula C37H68O3Si3, MW 644, CAS# NA, Entry# 299431

There is no signature data to report.

Library Search Report

Hit Spectrum	Compound Structure

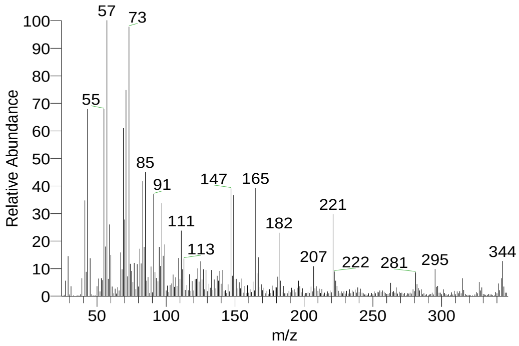

4H-1-BENZOPYRAN-4-ONE, 2-(3,4-DIMETHOXYPHENYL)-3,5-DIHYDROXY-7-METHOXY-
Formula C18H16O7, MW 344, CAS# 6068-80-0, Entry# 224392
3',4',7-TRIMETHYLQUERCETIN

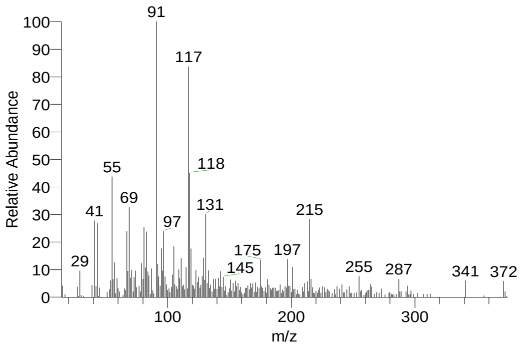

4-Hexyl-1-(7-methoxycarbonylheptyl)bicyclo[4.4.0]deca-2,5,7-triene
Formula C25H40O2, MW 372, CAS# NA, Entry# 66959
Methyl 8-(7-hexyl-3,7-dihydro-4a(4H)-naphthalenyl)octanoate #

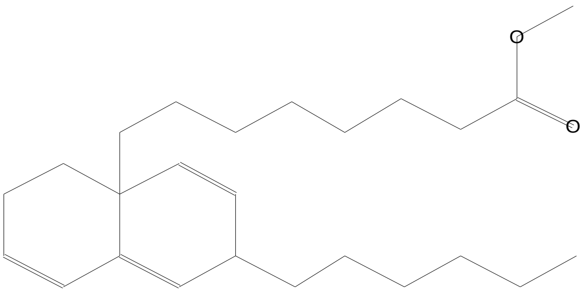

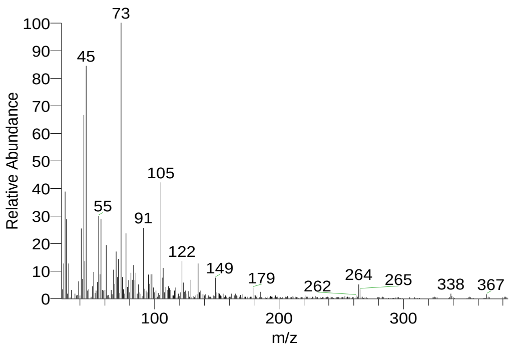

9-OCTADECENOIC ACID, (2-PHENYL-1,3-DIOXOLAN-4-YL)METHYL ESTER, CIS-
Formula C28H44O4, MW 444, CAS# 56599-45-2, Entry# 272719
(2-PHENYL-1,3-DIOXOLAN-4-YL)METHYL (9E)-9-OCTADECENOATE #

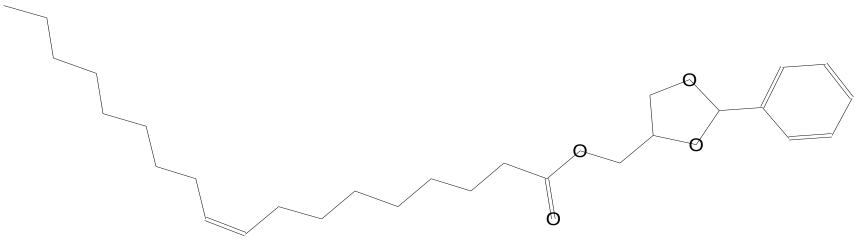

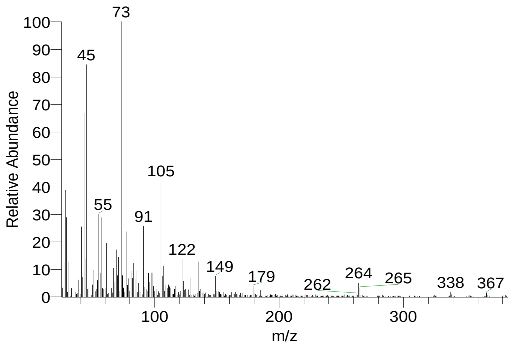

9-Octadecenoic acid, (2-phenyl-1,3-dioxolan-4-yl)methyl ester, cis-
Formula C28H44O4, MW 444, CAS# 56599-45-2, Entry# 44507
(2-Phenyl-1,3-dioxolan-4-yl)methyl 9-octadecenoate, cis-

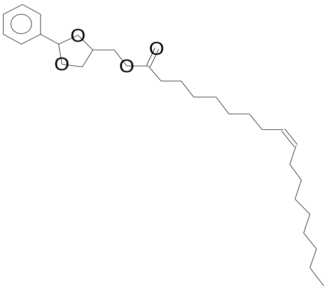

There is no signature data to report.

Library Search Report

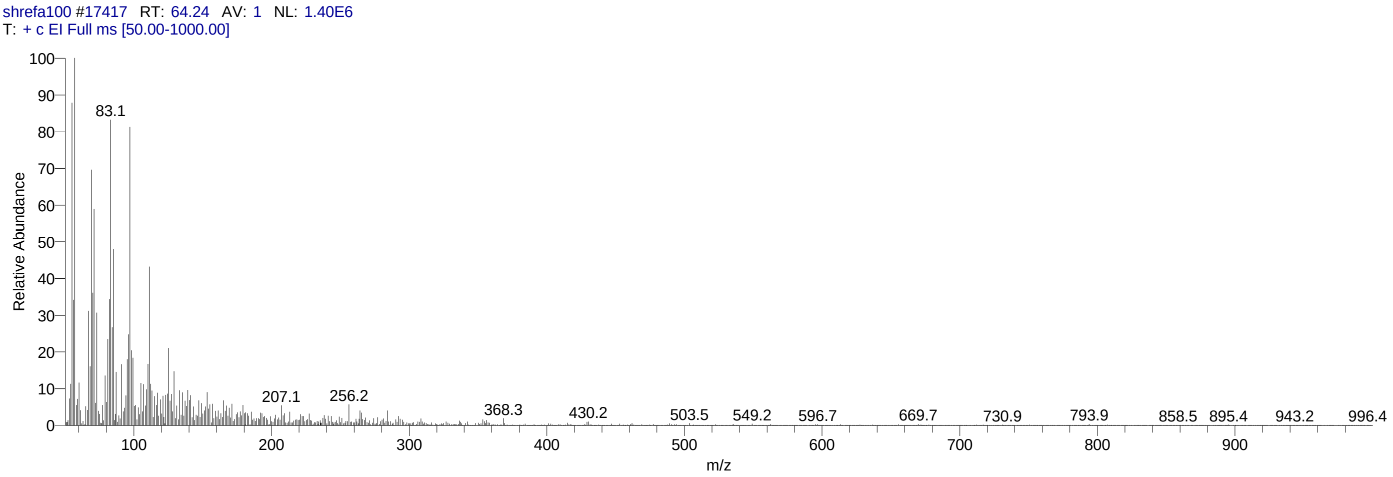

RT	Scan #	Probability	Compound Name	SI	RSI	Cas #	Area	Area %	Library
64.24	17417.000000	7.45	HAHNFETT	743	765	NA	41402489.15	0.51	WileyRegistry8e
64.24	17417.000000	7.45	HAHNFETT	743	765	NA	41402489.15	0.51	WileyRegistry8e
64.24	17417.000000	6.30	17-Pentatriacontene	739	756	6971-40-0	41402489.15	0.51	mainlib
64.24	17417.000000	5.81	cis-13-Eicosenoic acid	737	796	17735-94-3	41402489.15	0.51	mainlib
64.24	17417.000000	4.91	Oleic Acid	733	788	112-80-1	41402489.15	0.51	replib

Hit Spectrum | Compound Structure

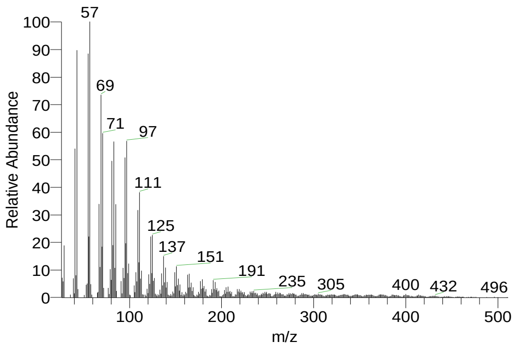

HAHNFETT
Formula , MW 0, CAS# NA, Entry# 305496

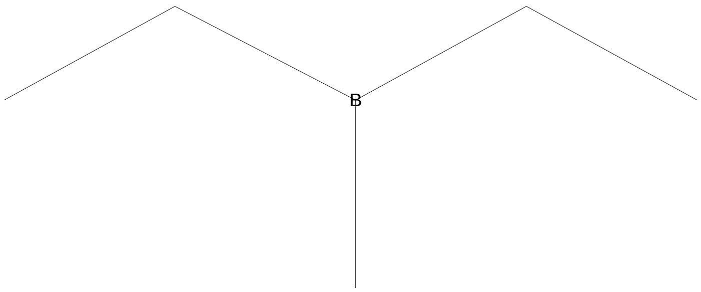

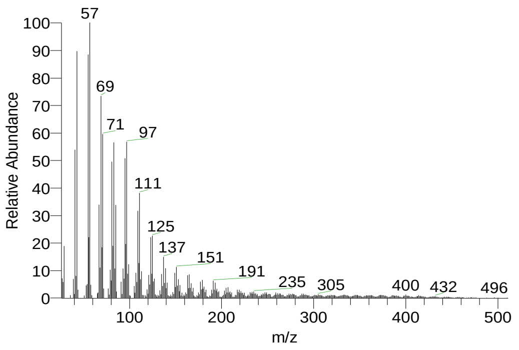

HAHNFETT
Formula , MW 0, CAS# NA, Entry# 391160

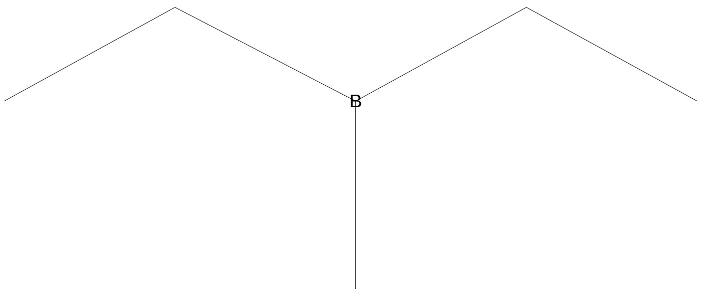

There is no signature data to report.

Library Search Report

Hit Spectrum | Compound Structure

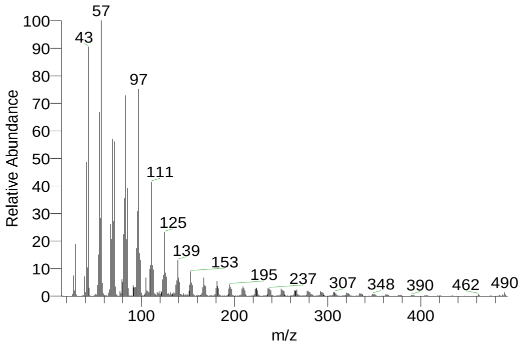

17-Pentatriacontene
Formula C35H70, MW 490, CAS# 6971-40-0, Entry# 26388
(17E)-17-Pentatriacontene #

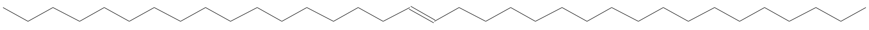

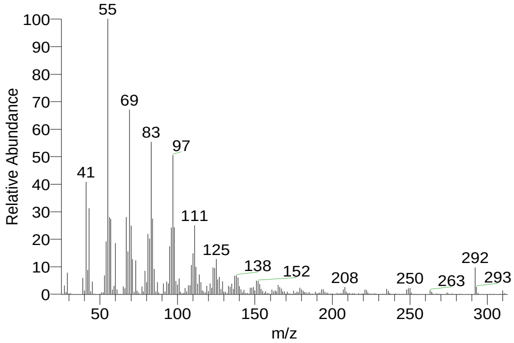

cis-13-Eicosenoic acid
Formula C20H38O2, MW 310, CAS# 17735-94-3, Entry# 21893
$:28URXZXNYJPAJJOQ-FPLPWBNLSA-N

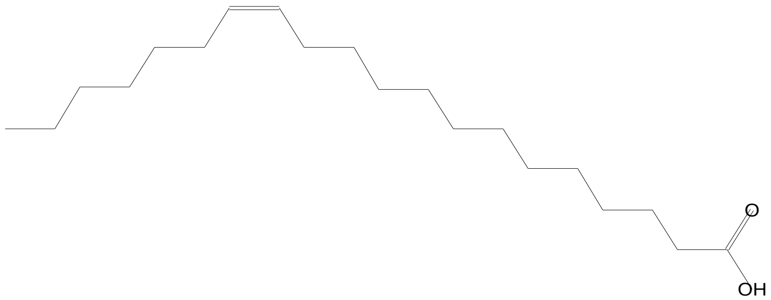

SI 733, RSI 788, replib, Entry# 5762, CAS# 112-80-1, Oleic Acid

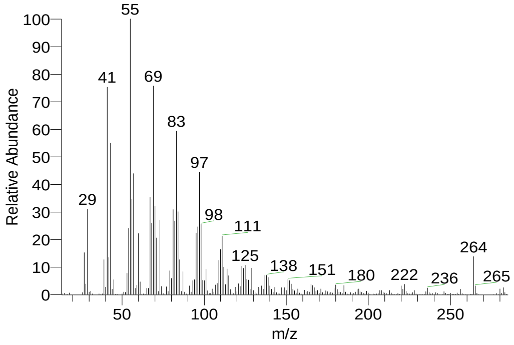

Oleic Acid
Formula C18H34O2, MW 282, CAS# 112-80-1, Entry# 5762
9-Octadecenoic acid (Z)-

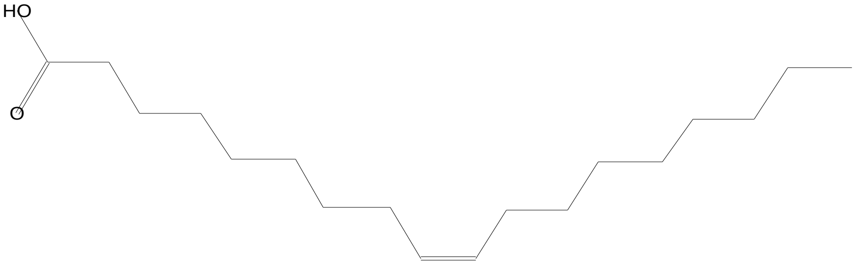

There is no signature data to report.

Library Search Report

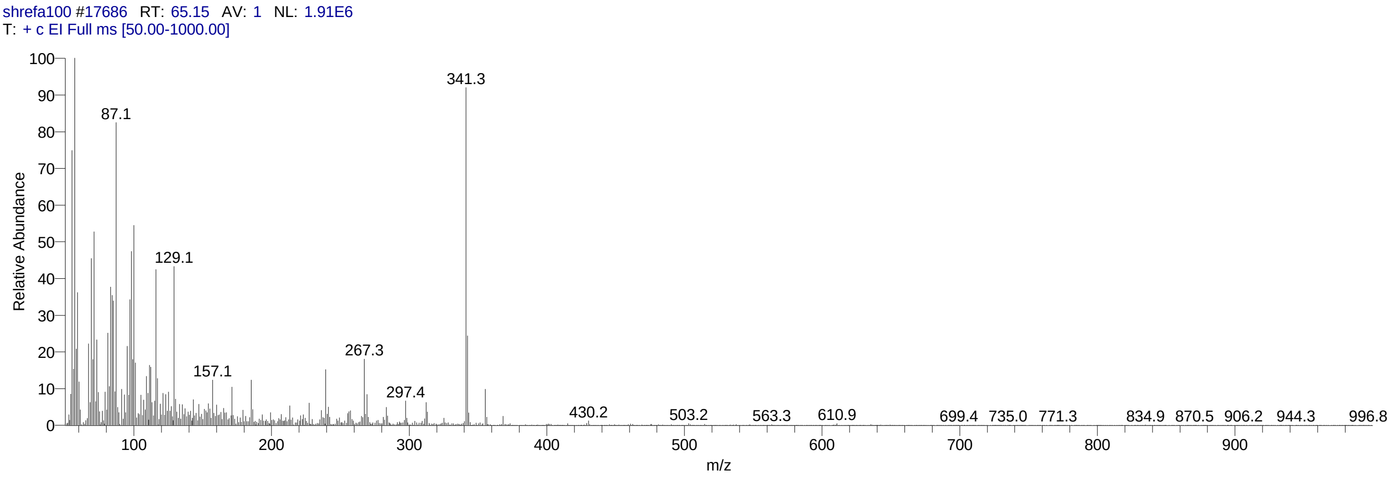

RT	Scan #	Probability	Compound Name	SI	RSI	Cas #	Area	Area %	Library
65.15	17686.000000	53.40	Hexadecanoic acid, (2-phenyl-1,3-dioxolan-4-yl)methyl ester, cis-	729	747	42495-31-8	85472107.95	1.05	mainlib
65.15	17686.000000	53.40	HEXADECANOIC ACID, (2-PHENYL-1,3-DIOXOLAN-4-YL)METHYL ESTER, CIS-	729	747	42495-31-8	85472107.95	1.05	WileyRegistry8e
65.15	17686.000000	12.59	Octadecanoic acid, 2-hydroxy-1,3-propanediyl di-ester	690	712	504-40-5	85472107.95	1.05	replib
65.15	17686.000000	12.59	OCTADECANOIC ACID, 2-HYDROXY-1,3-PROPANEDIYL ESTER	690	712	504-40-5	85472107.95	1.05	WileyRegistry8e
65.15	17686.000000	8.63	Distearin	679	688	1188-58-5	85472107.95	1.05	mainlib

Hit Spectrum | Compound Structure

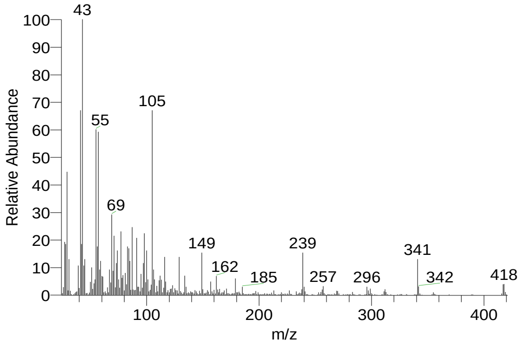

Hexadecanoic acid, (2-phenyl-1,3-dioxolan-4-yl)methyl ester, cis-
Formula $C_{26}H_{42}O_4$, MW 418, CAS# 42495-31-8, Entry# 6839
(2-Phenyl-1,3-dioxolan-4-yl)methyl palmitate, cis

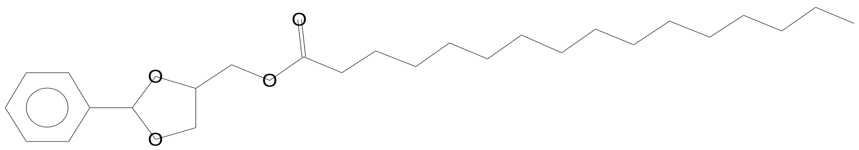

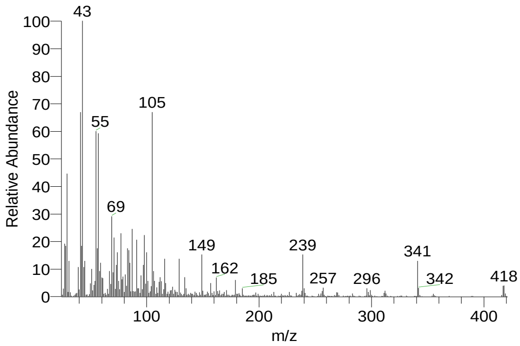

HEXADECANOIC ACID, (2-PHENYL-1,3-DIOXOLAN-4-YL)METHYL ESTER, CIS-
Formula $C_{26}H_{42}O_4$, MW 418, CAS# 42495-31-8, Entry# 263979
(2-PHENYL-1,3-DIOXOLAN-4-YL)METHYL PALMITATE #

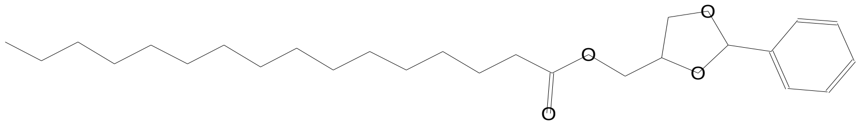

There is no signature data to report.

Library Search Report

Hit Spectrum | Compound Structure

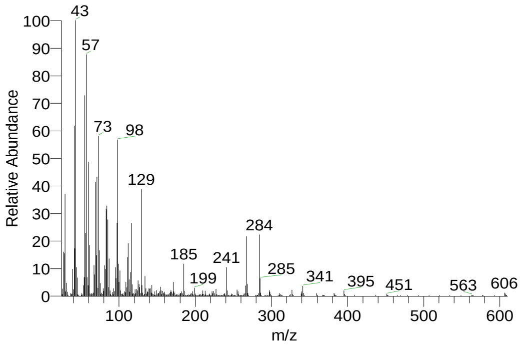

Octadecanoic acid, 2-hydroxy-1,3-propanediyl di-ester
Formula C39H76O5, MW 624, CAS# 504-40-5, Entry# 2607
Stearin, 1,3-di-

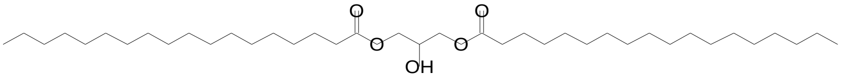

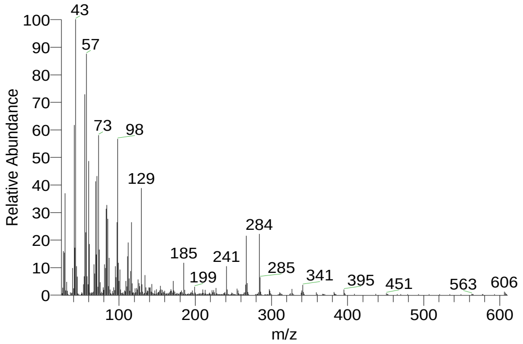

OCTADECANOIC ACID, 2-HYDROXY-1,3-PROPANEDIYL ESTER
Formula C39H76O5, MW 624, CAS# 504-40-5, Entry# 298386
2-HYDROXY-3-(STEAROYLOXY)PROPYL STEARATE #

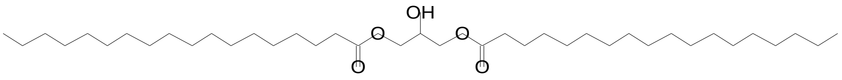

SI 679, RSI 688, mainlib, Entry# 237117, CAS# 1188-58-5, Distearin

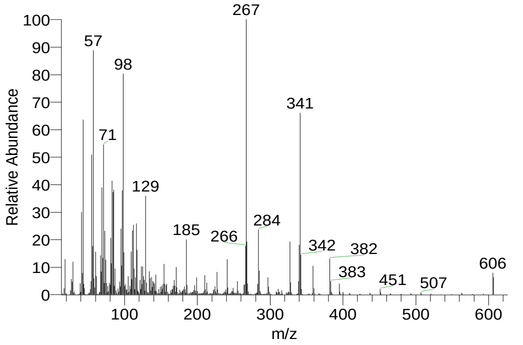

Distearin
Formula C39H76O5, MW 624, CAS# 1188-58-5, Entry# 237117
Octadecanoic acid, 1-(hydroxymethyl)-1,2-ethanediyl ester

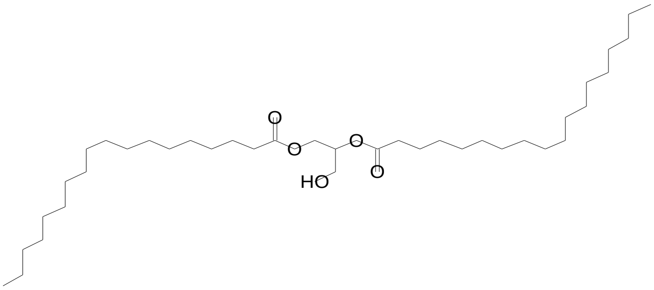

There is no signature data to report.

Library Search Report

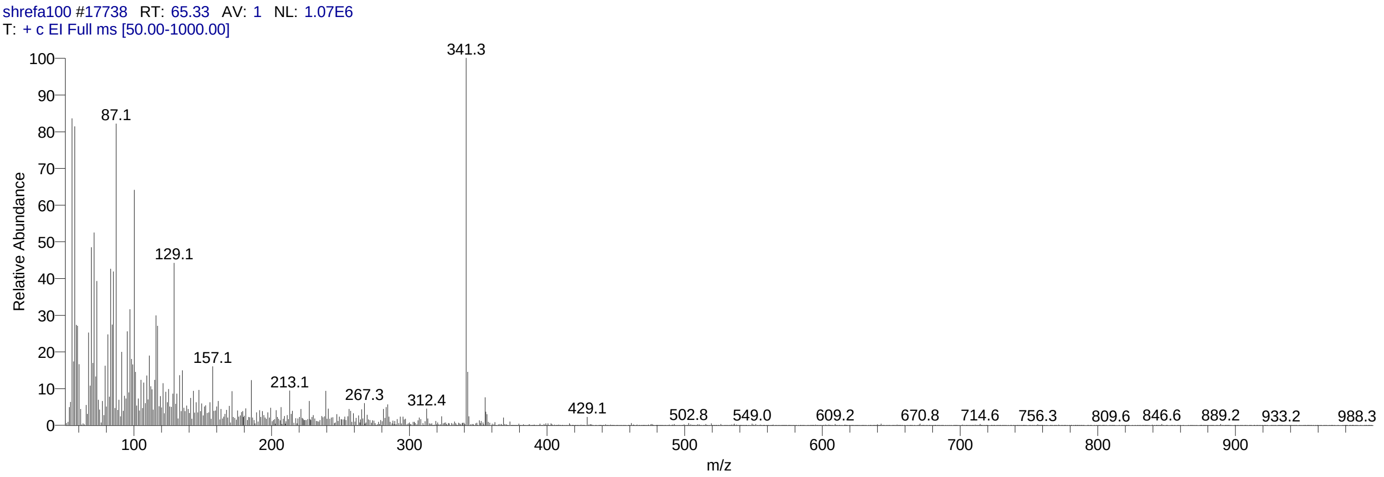

RT	Scan #	Probability	Compound Name	SI	RSI	Cas #	Area	Area %	Library
65.33	17738.000000	62.46	Spironolactone	739	910	52-01-7	35767005.54	0.44	nist_msms
65.33	17738.000000	62.46	Spironolactone	732	904	52-01-7	35767005.54	0.44	nist_msms
65.33	17738.000000	16.35	Hexadecanoic acid, (2-phenyl-1,3-dioxolan-4-yl)methyl ester, cis-	706	735	42495-31-8	35767005.54	0.44	mainlib
65.33	17738.000000	16.35	HEXADECANOIC ACID, (2-PHENYL-1,3-DIOXOLAN-4-YL)METHYL ESTER, CIS-	706	735	42495-31-8	35767005.54	0.44	WileyRegistry8e
65.33	17738.000000	4.36	3,20-DIOXO-11-à-HYDROXYCONANINE-1,4-DIENE	674	730	NA	35767005.54	0.44	WileyRegistry8e

Hit Spectrum | Compound Structure

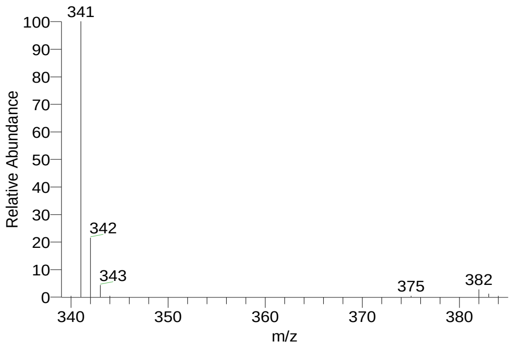

Spironolactone
Formula C24H32O4S, MW 416, CAS# 52-01-7, Entry# 74003
4-Pregnen-21-oic acid-17à-ol-3-one-7à-thiol ç-lactone 7-acetate

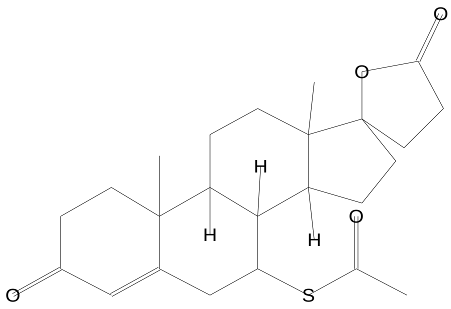

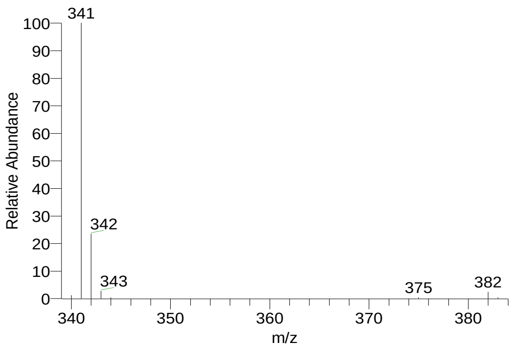

Spironolactone
Formula C24H32O4S, MW 416, CAS# 52-01-7, Entry# 74004
4-Pregnen-21-oic acid-17à-ol-3-one-7à-thiol ç-lactone 7-acetate

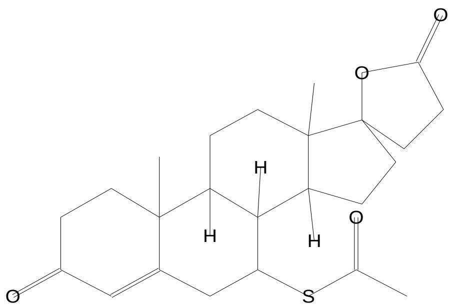

There is no signature data to report.

Library Search Report

Hit Spectrum	Compound Structure

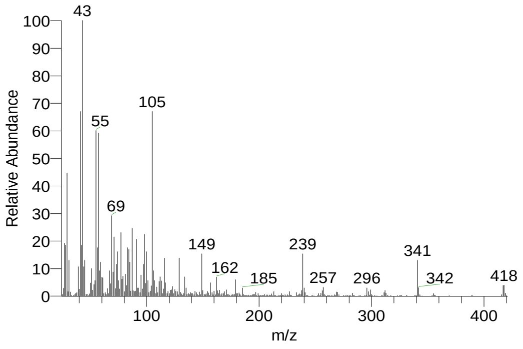

Hexadecanoic acid, (2-phenyl-1,3-dioxolan-4-yl)methyl ester, cis-
Formula C26H42O4, MW 418, CAS# 42495-31-8, Entry# 6839
(2-Phenyl-1,3-dioxolan-4-yl)methyl palmitate, cis

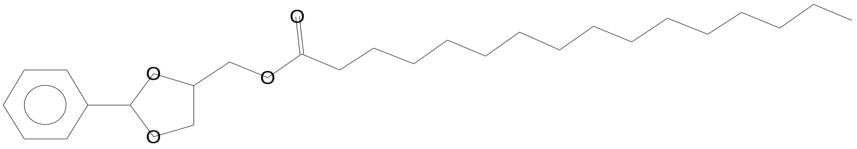

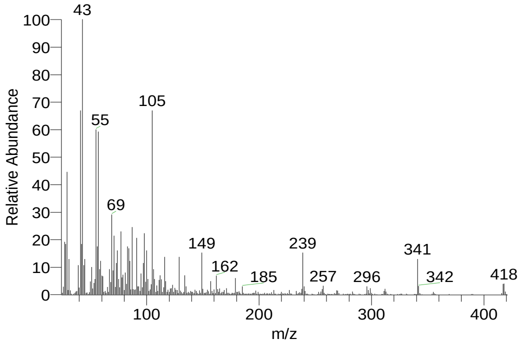

HEXADECANOIC ACID, (2-PHENYL-1,3-DIOXOLAN-4-YL)METHYL ESTER, CIS-
Formula C26H42O4, MW 418, CAS# 42495-31-8, Entry# 263979
(2-PHENYL-1,3-DIOXOLAN-4-YL)METHYL PALMITATE #

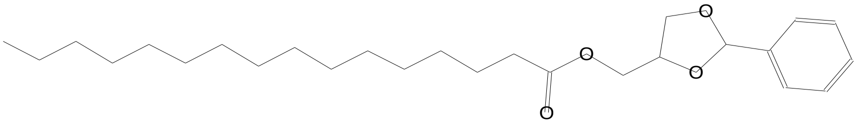

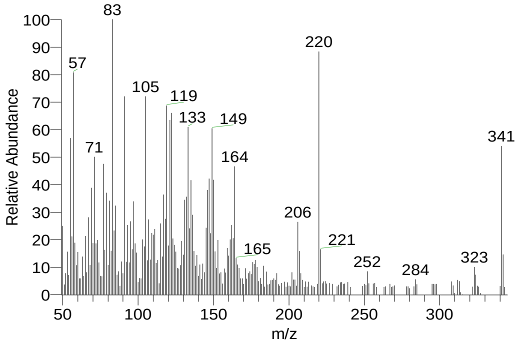

3,20-DIOXO-11-à-HYDROXYCONANINE-1,4-DIENE
Formula C21H27NO3, MW 341, CAS# NA, Entry# 222649

There is no signature data to report.

Library Search Report

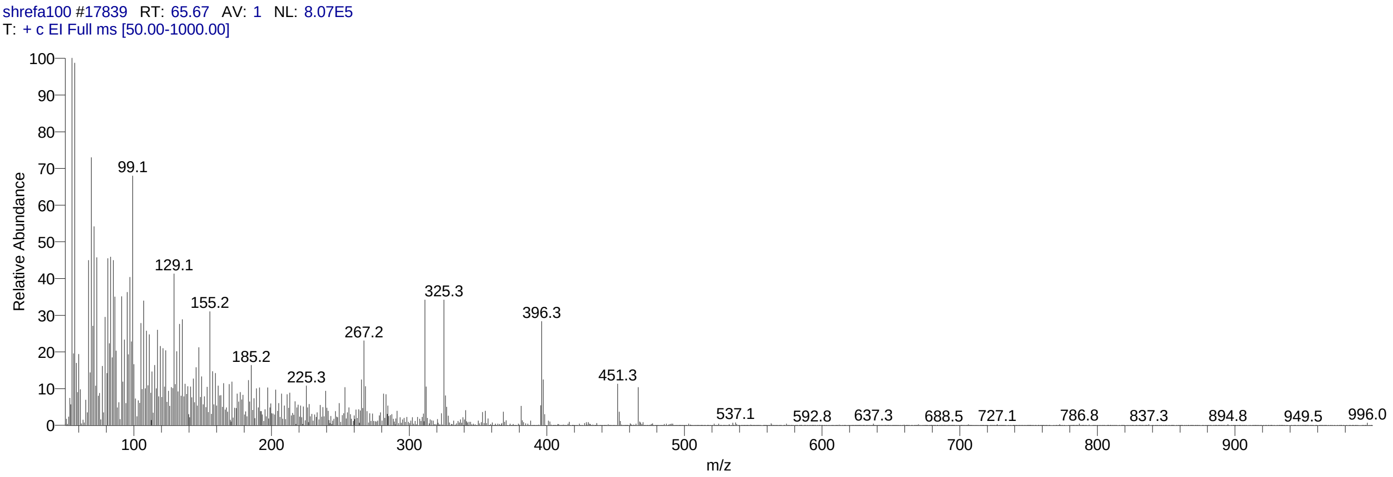

RT	Scan #	Probability	Compound Name	SI	RSI	Cas #	Area	Area %	Library
65.67	17839.000000	22.17	Docosanoic acid, 1,2,3-propanetriyl ester	688	697	18641-57-1	43499728.66	0.53	mainlib
65.67	17839.000000	22.17	DOCOSANOIC ACID, 1,2,3-PROPANETRIYL ESTER	688	697	18641-57-1	43499728.66	0.53	WileyRegistry8e
65.67	17839.000000	18.73	Buprenorphine glucuronide	684	727	101224-22-0	43499728.66	0.53	nist_msms
65.67	17839.000000	7.40	OCTADECANOIC ACID, 2-HYDROXYETHYL ESTER	662	766	111-60-4	43499728.66	0.53	WileyRegistry8e
65.67	17839.000000	7.11	<No Name>	661	731	NA	43499728.66	0.53	WileyRegistry8e

Hit Spectrum	Compound Structure

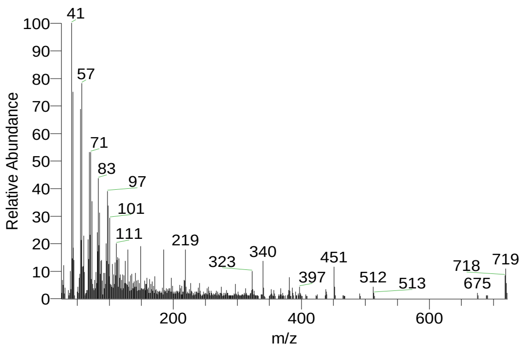

Docosanoic acid, 1,2,3-propanetriyl ester
Formula C69H134O6, MW 1058, CAS# 18641-57-1, Entry# 3322
Docosanoin, tri-

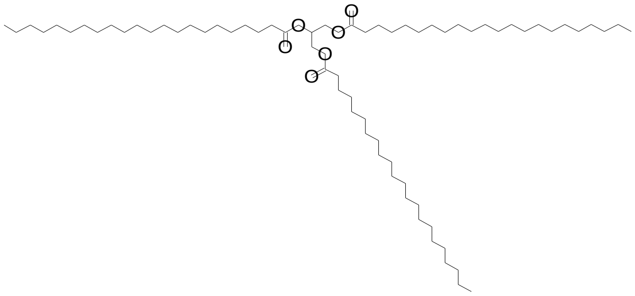

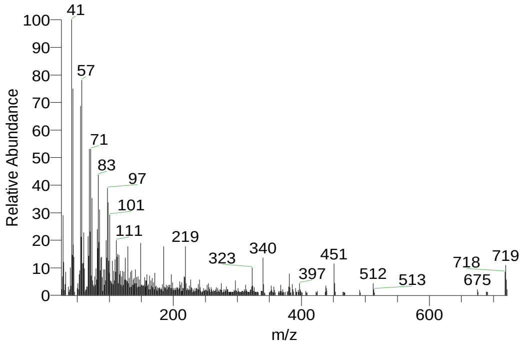

DOCOSANOIC ACID, 1,2,3-PROPANETRIYL ESTER
Formula C69H134O6, MW 1058, CAS# 18641-57-1, Entry# 305117
2,3-BIS(DOCOSANOYLOXY)PROPYL DOCOSANOATE #

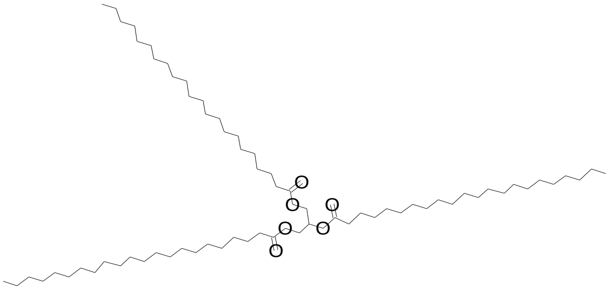

There is no signature data to report.

Library Search Report

Hit Spectrum | Compound Structure

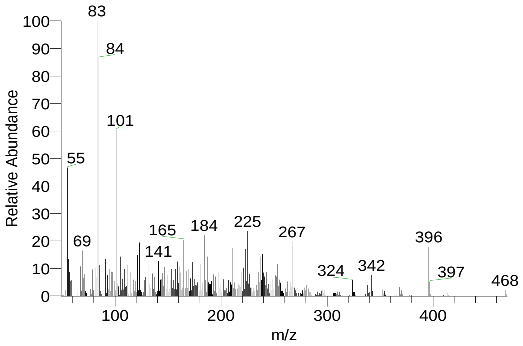

Buprenorphine glucuronide
Formula C35H49NO10, MW 643, CAS# 101224-22-0, Entry# 322531

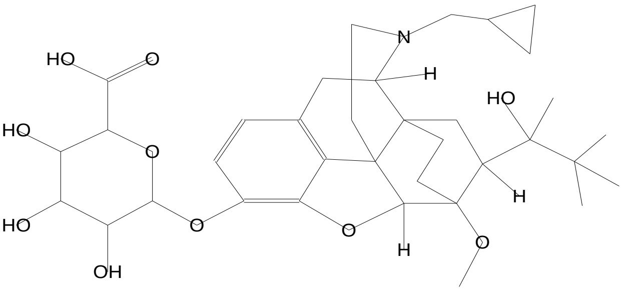

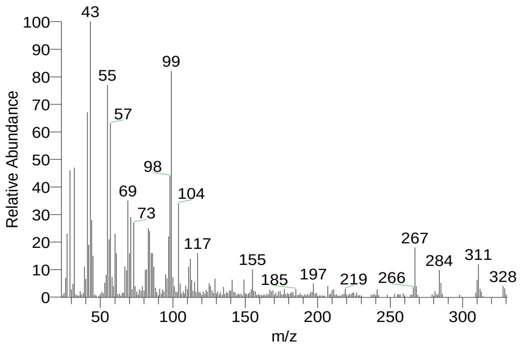

OCTADECANOIC ACID, 2-HYDROXYETHYL ESTER
Formula C20H40O3, MW 328, CAS# 111-60-4, Entry# 213198
2-HYDROXYETHYL OCTADECANOATE

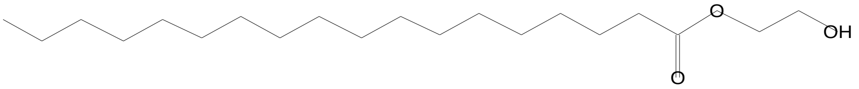

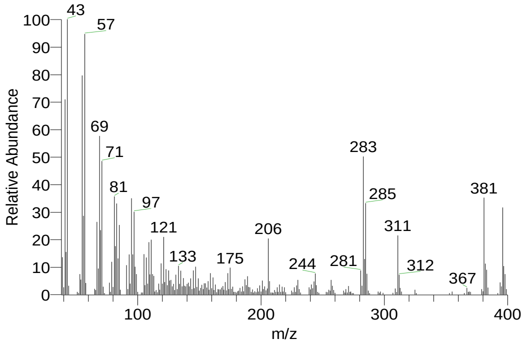

<No Name>
Formula C29H48, MW 396, CAS# NA, Entry# 361900

There is no signature data to report.

Library Search Report

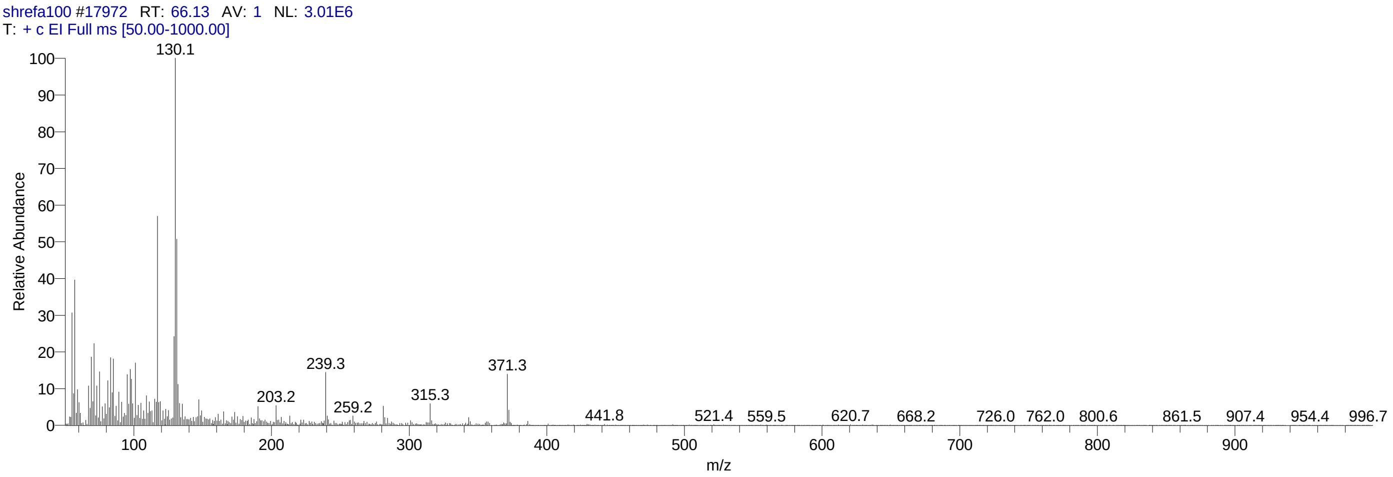

RT	Scan #	Probability	Compound Name	SI	RSI	Cas #	Area	Area %	Library
66.13	17972.000000	63.26	3-Hydroxypropyl palmitate, TMS derivative	645	746	56630-48-9	73284747.16	0.90	mainlib
66.13	17972.000000	63.26	HEXADECANOIC ACID, 3-[(TRIMETHYLSILYL)OXY]PROPYL ESTER	641	740	56630-48-9	73284747.16	0.90	WileyRegistry8e
66.13	17972.000000	18.61	1-Monopalmitin, 2TMS derivative	618	727	1188-74-5	73284747.16	0.90	replib
66.13	17972.000000	18.61	HEXADECANOIC ACID, 2,3-BIS[(TRIMETHYLSILYL)OXY]PROPYL ESTER	617	726	1188-74-5	73284747.16	0.90	WileyRegistry8e
66.13	17972.000000	5.17	10,12-Docosadiynedioic acid, 2TMS derivative	588	623	NA	73284747.16	0.90	mainlib

Hit Spectrum | Compound Structure

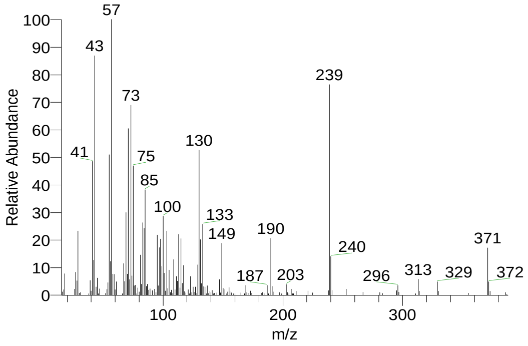

3-Hydroxypropyl palmitate, TMS derivative
Formula C22H46O3Si, MW 386, CAS# 56630-48-9, Entry# 26457
Hexadecanoic acid, 3-[(trimethylsilyl)oxy]propyl ester

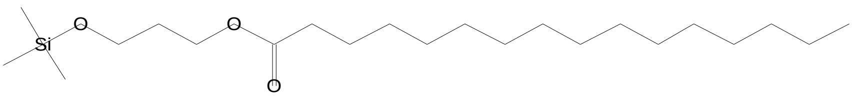

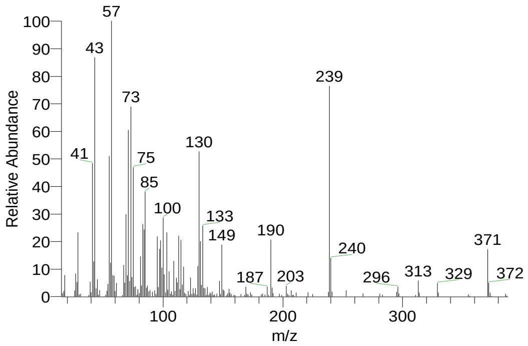

HEXADECANOIC ACID, 3-[(TRIMETHYLSILYL)OXY]PROPYL ESTER
Formula C22H46O3Si, MW 386, CAS# 56630-48-9, Entry# 249351
3-[(TRIMETHYLSILYL)OXY]PROPYL PALMITATE #

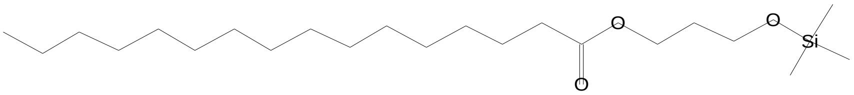

There is no signature data to report.

Library Search Report

Hit Spectrum	Compound Structure

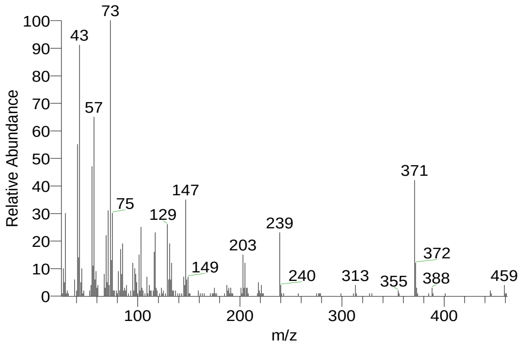

1-Monopalmitin, 2TMS derivative
Formula C25H54O4Si2, MW 474, CAS# 1188-74-5, Entry# 11005
Hexadecanoic acid, 2,3-bis[(trimethylsilyl)oxy]propyl ester

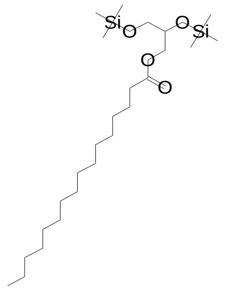

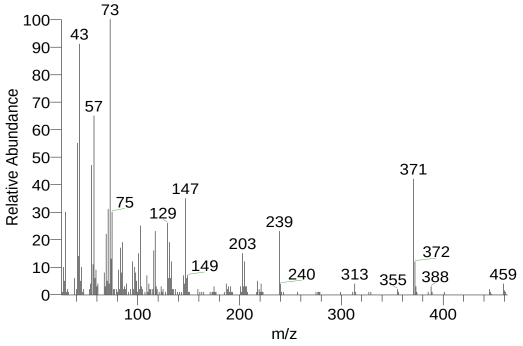

HEXADECANOIC ACID, 2,3-BIS[(TRIMETHYLSILYL)OXY]PROPYL ESTER
Formula C25H54O4Si2, MW 474, CAS# 1188-74-5, Entry# 280422
2,3-BIS[(TRIMETHYLSILYL)OXY]PROPYL PALMITATE #

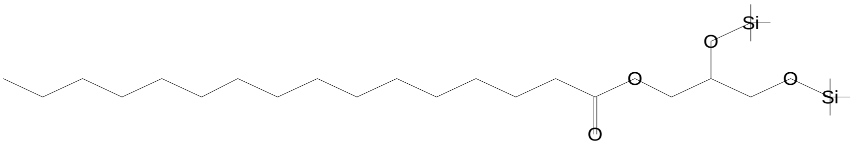

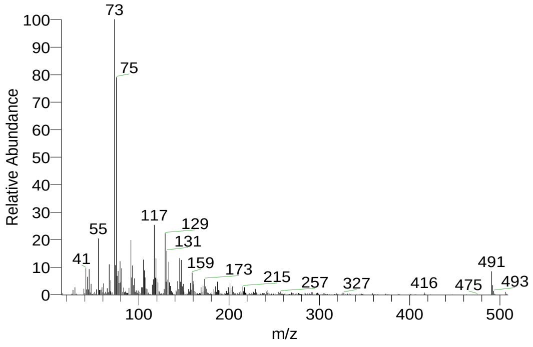

10,12-Docosadiynedioic acid, 2TMS derivative
Formula C28H50O4Si2, MW 506, CAS# NA, Entry# 45179
10,12-Docosadiynedioic acid ditms

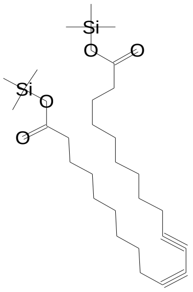

There is no signature data to report.

Library Search Report

shrefa100 #18111 RT: 66.60 AV: 1 NL: 7.60E6
T: + c EI Full ms [50.00-1000.00]

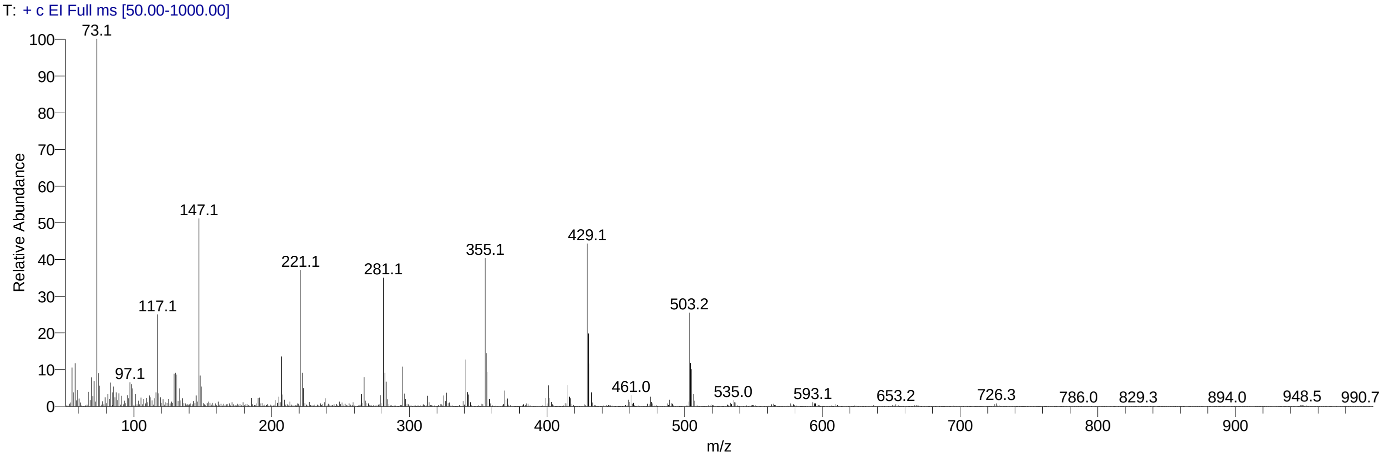

RT	Scan #	Probability	Compound Name	SI	RSI	Cas #	Area	Area %	Library
66.60	18111.000000	27.08	Cyclodecasiloxane, eicosamethyl-	775	816	18772-36-6	235647654.73	2.89	mainlib
66.60	18111.000000	27.08	2,2,4,4,6,6,8,8,10,10,12,12,14,14,16,16,18,18,20,20-ICOSAMETHYLCYCLODECASILOXANE #	775	816	18772-36-6	235647654.73	2.89	WileyRegistry8e
66.60	18111.000000	23.93	SILICONE OIL	772	827	NA	235647654.73	2.89	WileyRegistry8e
66.60	18111.000000	23.93	SILIKONFETT SE30 (GREVELS)	772	827	NA	235647654.73	2.89	WileyRegistry8e
66.60	18111.000000	20.21	1H-PURIN-6-AMINE, [(2-FLUOROPHENYL)METHYL]-	768	829	74421-44-6	235647654.73	2.89	WileyRegistry8e

Hit Spectrum | Compound Structure

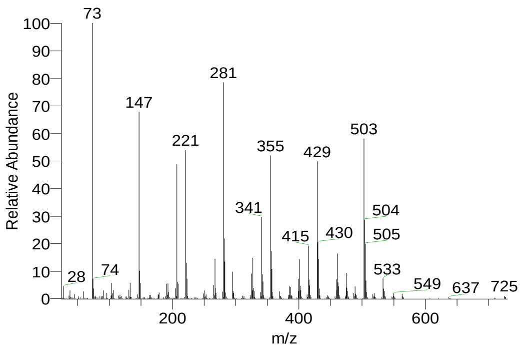

Cyclodecasiloxane, eicosamethyl-
Formula C20H60O10Si10, MW 740, CAS# 18772-36-6, Entry# 47864
2,2,4,4,6,6,8,8,10,10,12,12,14,14,16,16,18,18,20,20-Icosamethylcyclodecasiloxane #

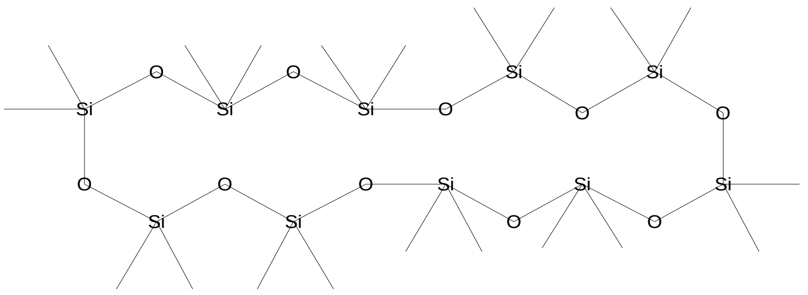

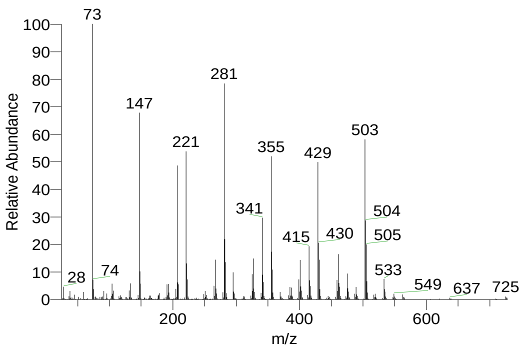

2,2,4,4,6,6,8,8,10,10,12,12,14,14,16,16,18,18,20,20-ICOSAMETHYLCYCLODECASILOXANE #
Formula C20H60O10Si10, MW 740, CAS# 18772-36-6, Entry# 380233
2,2,4,4,6,6,8,8,10,10,12,12,14,14,16,16,18,18,20,20-ICOSAMETHYLCYCLODECASILOXANE

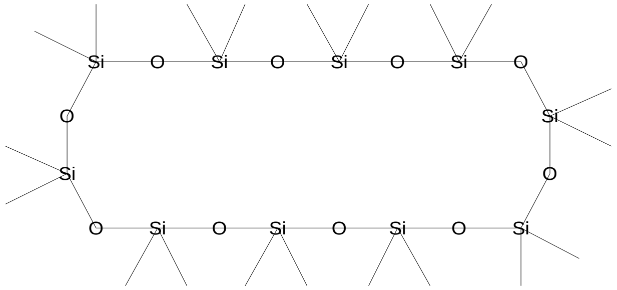

There is no signature data to report.

Library Search Report

Hit Spectrum | Compound Structure

SILICONE OIL
Formula , MW 0, CAS# NA, Entry# 305490
SILIKONFETT SE30 (GREVELS)

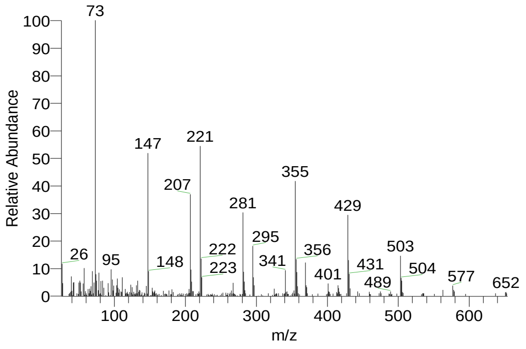

SILIKONFETT SE30 (GREVELS)
Formula , MW 0, CAS# NA, Entry# 392776

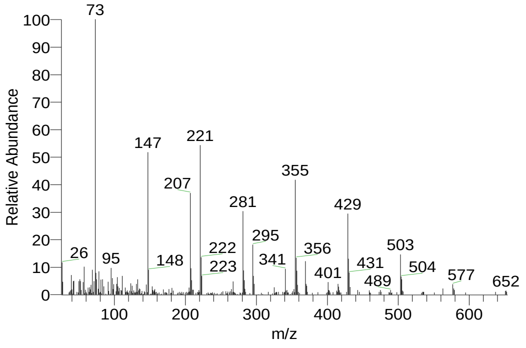

1H-PURIN-6-AMINE, [(2-FLUOROPHENYL)METHYL]-
Formula C12H10FN5, MW 243, CAS# 74421-44-6, Entry# 132518

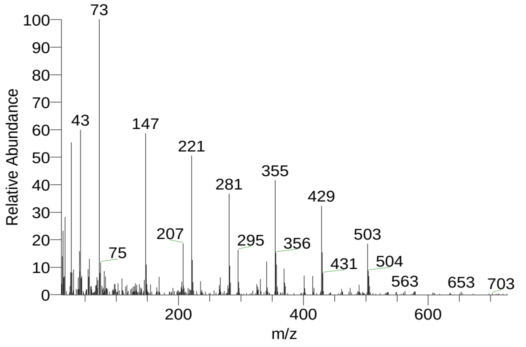

There is no signature data to report.

Library Search Report

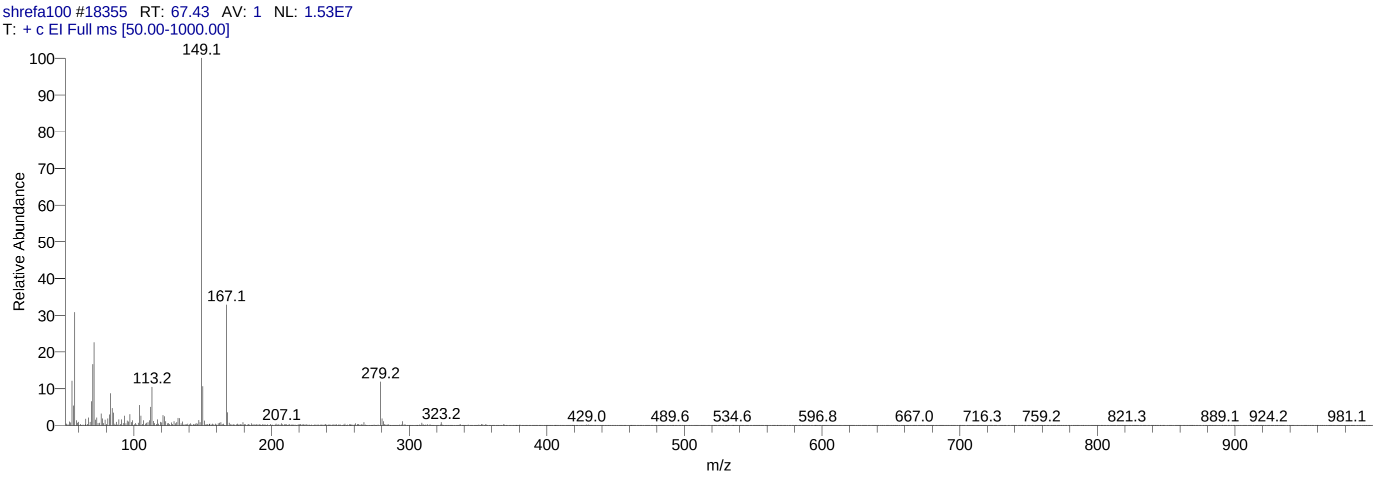

RT	Scan #	Probability	Compound Name	SI	RSI	Cas #	Area	Area %	Library
67.43	18355.000000	15.00	1,2-BENZENEDICARBOXYLIC ACID	812	849	117-81-7	186231228.22	2.28	WileyRegistry8e
67.43	18355.000000	14.42	BIS(2-ETHYLHEXYL) PHTHALATE	811	895	NA	186231228.22	2.28	WileyRegistry8e
67.43	18355.000000	9.60	1,2-BENZENEDICARBOXYLIC ACID, DIOCTYL ESTER	799	864	117-84-0	186231228.22	2.28	WileyRegistry8e
67.43	18355.000000	15.00	1,2-BENZENEDICARBOXYLIC ACID	790	874	117-81-7	186231228.22	2.28	WileyRegistry8e
67.43	18355.000000	6.97	1,2-BENZENEDICARBOXYLIC ACID, DIISOOCTYL ESTER	790	874	27554-26-3	186231228.22	2.28	WileyRegistry8e

Hit Spectrum

Compound Structure

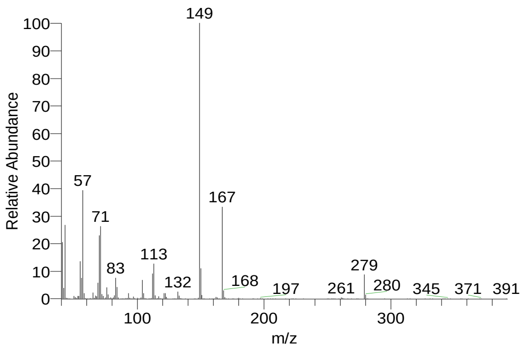

1,2-BENZENEDICARBOXYLIC ACID
Formula C24H38O4, MW 390, CAS# 117-81-7, Entry# 251627
1,2-BENZENEDICARBOXYLIC ACID, BIS(2-ETHYLHEXYL) ESTER

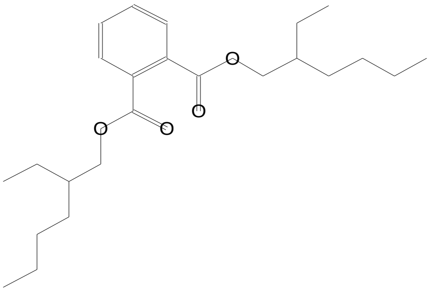

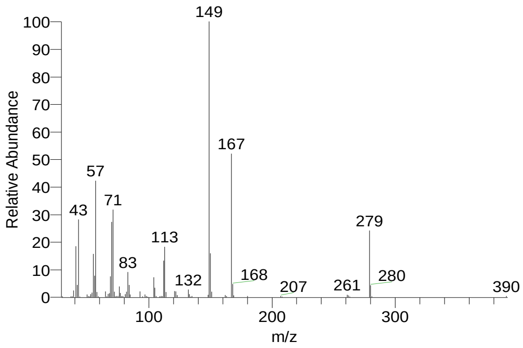

BIS(2-ETHYLHEXYL) PHTHALATE
Formula C24H38O4, MW 390, CAS# NA, Entry# 359289
BIS-(2-ETHYLHEXYL)PHTHALAT

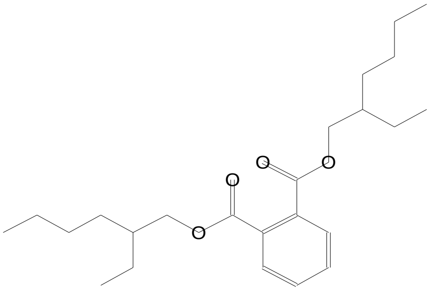

There is no signature data to report.

Library Search Report

Hit Spectrum	Compound Structure

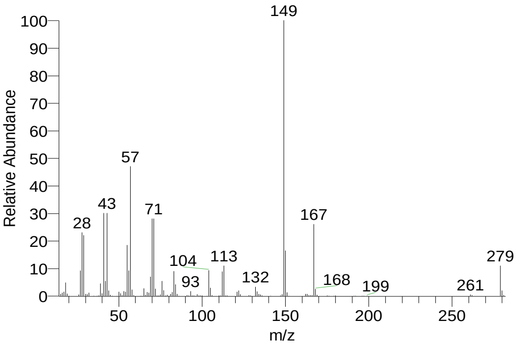

1,2-BENZENEDICARBOXYLIC ACID, DIOCTYL ESTER
Formula C24H38O4, MW 390, CAS# 117-84-0, Entry# 251617
1,2-BENZENEDICARBONIC ACID, DIOCTYL ESTER

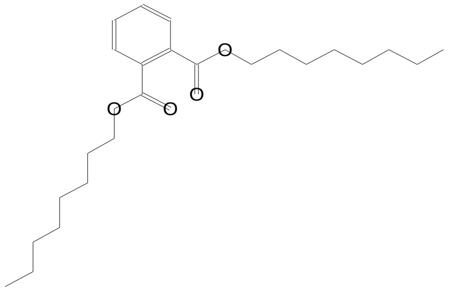

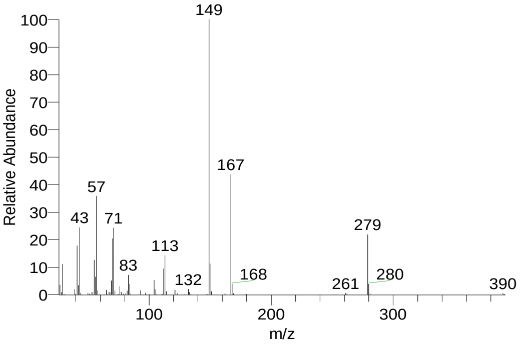

1,2-BENZENEDICARBOXYLIC ACID
Formula C24H38O4, MW 390, CAS# 117-81-7, Entry# 251625
1,2-BENZENEDICARBOXYLIC ACID, BIS(2-ETHYLHEXYL) ESTER

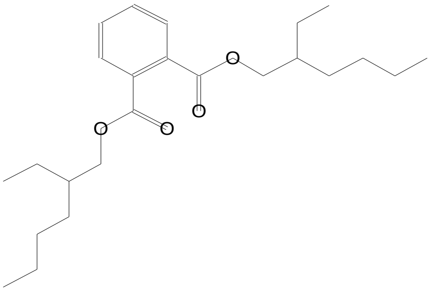

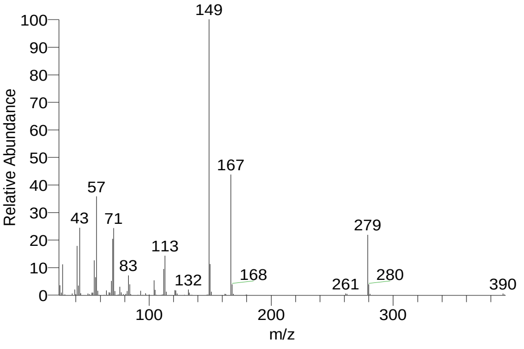

1,2-BENZENEDICARBOXYLIC ACID, DIISOOCTYL ESTER
Formula C24H38O4, MW 390, CAS# 27554-26-3, Entry# 251641
BIS(6-METHYLHEPTYL) PHTHALATE #

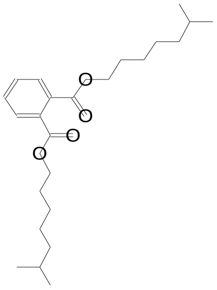

There is no signature data to report.

Library Search Report

shrefa100 #18427 RT: 67.67 AV: 1 NL: 1.83E6
T: + c EI Full ms [50.00-1000.00]

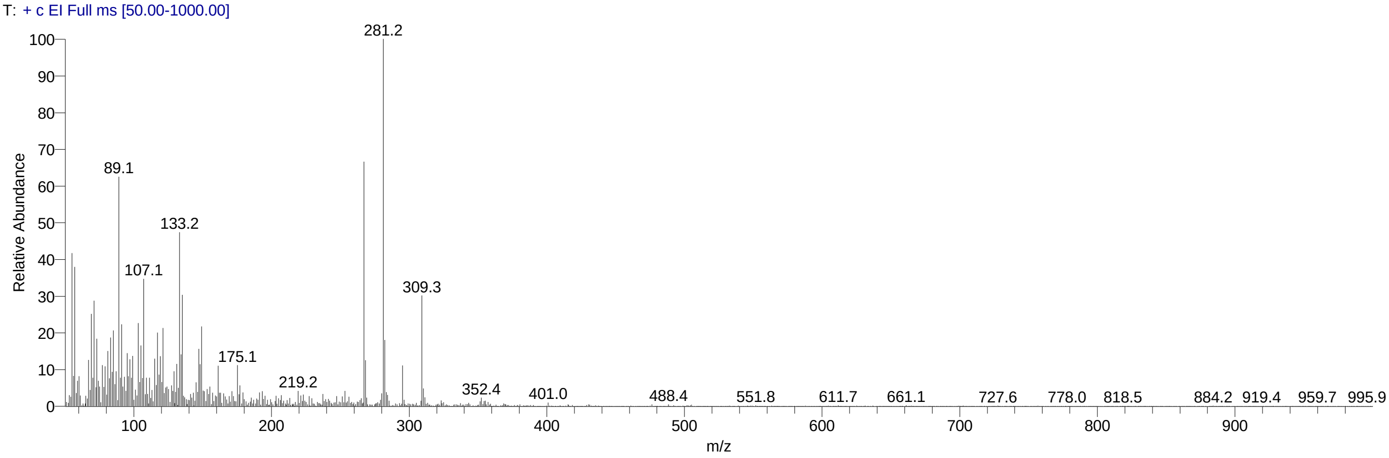

RT	Scan #	Probability	Compound Name	SI	RSI	Cas #	Area	Area %	Library
67.67	18427.000000	12.07	9-NONYLPHENYL-3,6,9-TRIOXANONANOL, MIX OF ISOMERS	621	738	NA	64508404.54	0.79	WileyRegistry8e
67.67	18427.000000	10.19	4,11-Dispiro(2'-cyclobutanone)tricyclo[6.2.2.0(2,7)]dodeca-5, 9-diene, 1,3,3,5,12,12-hexamethyl-	617	634	NA	64508404.54	0.79	mainlib
67.67	18427.000000	10.19	4,11-DISPIRO(2'-CYCLOBUTANONE)TRICYCLO[6.2.2.0(2,7)]DODECA-5,9-DIENE, 1,3,3,5,12,12-HEXAMETHYL-	617	634	NA	64508404.54	0.79	WileyRegistry8e
67.67	18427.000000	9.01	Strychane, 1-acetyl-20à-hydroxy-16-methylene-	614	650	2111-98-0	64508404.54	0.79	mainlib
67.67	18427.000000	9.01	STRYCHANE, 1-ACETYL-20à-HYDROXY-16-METHYLENE-	614	648	2111-98-0	64508404.54	0.79	WileyRegistry8e

Hit Spectrum | Compound Structure

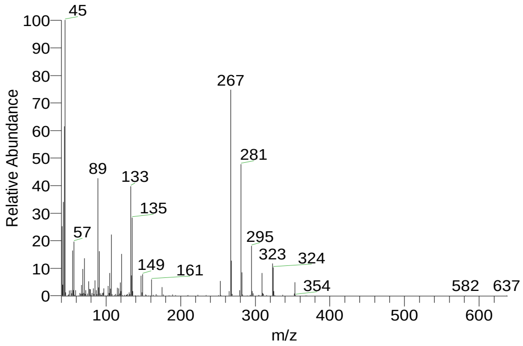

9-NONYLPHENYL-3,6,9-TRIOXANONANOL, MIX OF ISOMERS
Formula C21H36O4, MW 352, CAS# NA, Entry# 230107

There is no signature data to report.

Library Search Report

Hit Spectrum | Compound Structure

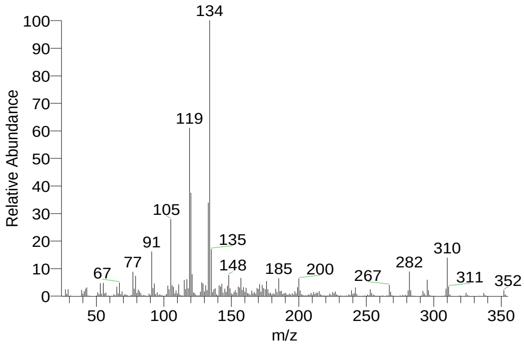

4,11-Dispiro(2'-cyclobutanone)tricyclo[6.2.2.0(2,7)]dodeca-5,9-diene, 1,3,3,5,12,12-hexamethyl-
Formula C24H32O2, MW 352, CAS# NA, Entry# 129761
$:28GKQMPZRTBMBGBR-UHFFFAOYSA-N

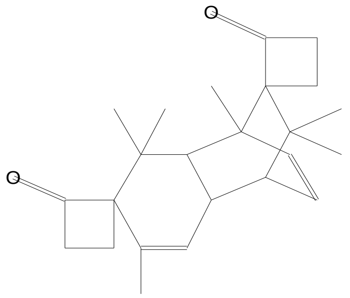

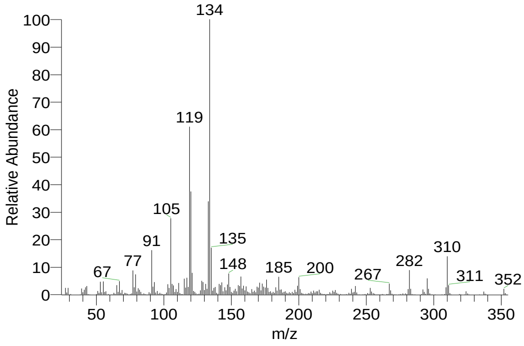

Formula C24H32O2, MW 352, CAS# NA, Entry# 370430

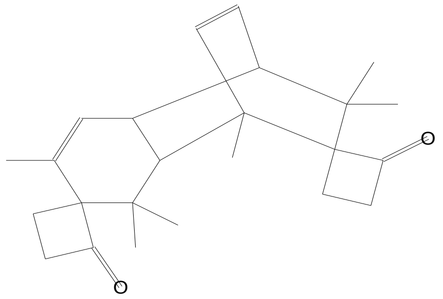

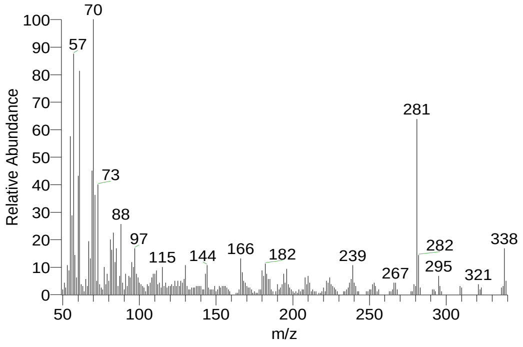

Strychane, 1-acetyl-20à-hydroxy-16-methylene-
Formula C21H26N2O2, MW 338, CAS# 2111-98-0, Entry# 40455
1-Acetylcur-16-en-20-ol #

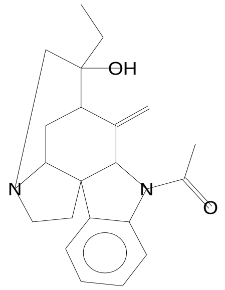

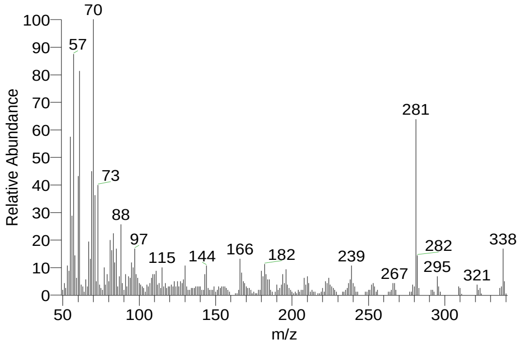

STRYCHANE, 1-ACETYL-20à-HYDROXY-16-METHYLENE-
Formula C21H26N2O2, MW 338, CAS# 2111-98-0, Entry# 220600
1-ACETYLCUR-16-EN-20-OL #

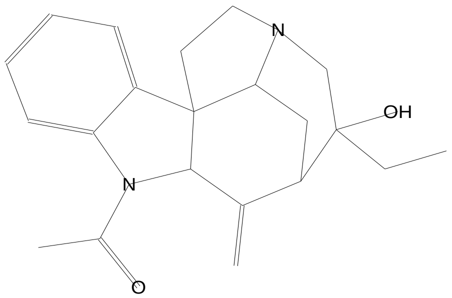

There is no signature data to report.

Library Search Report

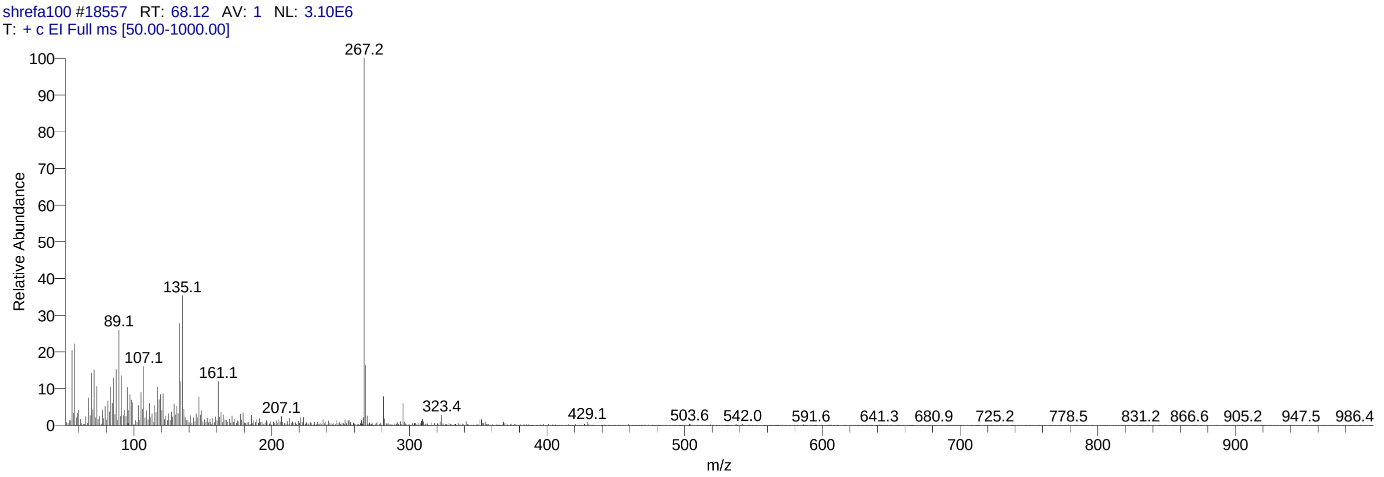

RT	Scan #	Probability	Compound Name	SI	RSI	Cas #	Area	Area %	Library
68.12	18557.000000	49.97	Ethanol, 2-[2-[2-[4-(1,1,3,3-tetramethylbutyl)phenoxy]ethoxy]ethoxy]-	693	794	2315-62-0	74718272.87	0.91	mainlib
68.12	18557.000000	49.97	ETHANOL, 2-[2-[2-[4-(1,1,3,3-TETRAMETHYLBUTYL)PHENOXY]ETHOXY]ETHOXY]-	688	789	2315-62-0	74718272.87	0.91	WileyRegistry8e
68.12	18557.000000	12.16	2-Hydroxy-2-octylsebacic acid	656	695	101885-38-5	74718272.87	0.91	mainlib
68.12	18557.000000	9.31	Octadecanoic acid, 1-[(tetradecyloxy)carbonyl]pentadecyl ester	649	661	69688-49-9	74718272.87	0.91	mainlib
68.12	18557.000000	9.31	OCTADECANOIC ACID, 1-[(TETRADECYLOXY)CARBONYL]PENTADECYL ESTER	649	661	69688-49-9	74718272.87	0.91	WileyRegistry8e

Hit Spectrum	Compound Structure

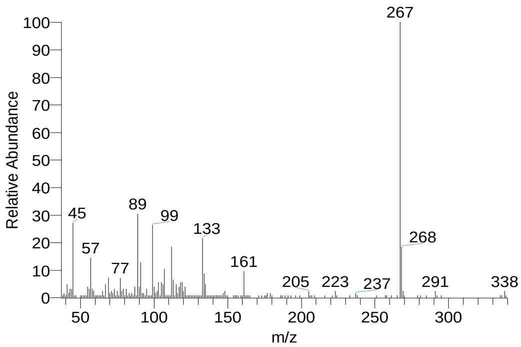

Ethanol, 2-[2-[2-[4-(1,1,3,3-tetramethylbutyl)phenoxy]ethoxy]ethoxy]-
Formula C20H34O4, MW 338, CAS# 2315-62-0, Entry# 237170
2-(2-(2-[4-(1,1,3,3-Tetramethylbutyl)phenoxy]ethoxy)ethoxy)ethanol #

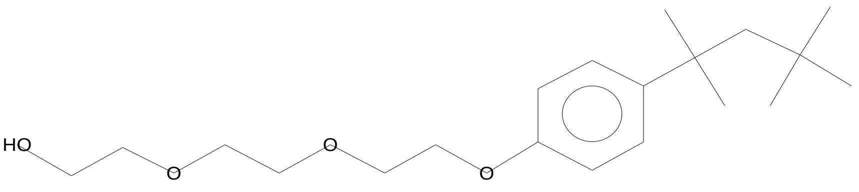

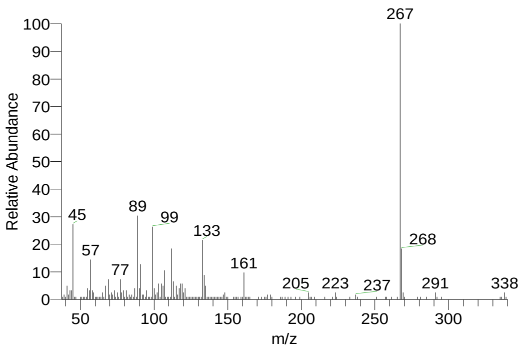

ETHANOL, 2-[2-[2-[4-(1,1,3,3-TETRAMETHYLBUTYL)PHENOXY]ETHOXY]ETHOXY]-
Formula C20H34O4, MW 338, CAS# 2315-62-0, Entry# 220509
2-(2-(2-[4-(1,1,3,3-TETRAMETHYLBUTYL)PHENOXY]ETHOXY)ETHOXY)ETHANOL #

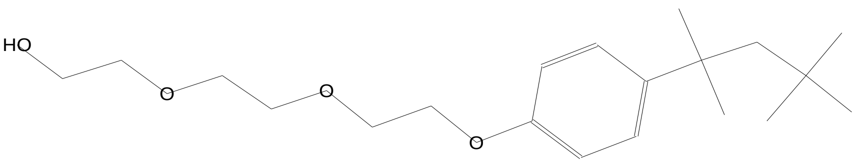

There is no signature data to report.

Library Search Report

Hit Spectrum | Compound Structure

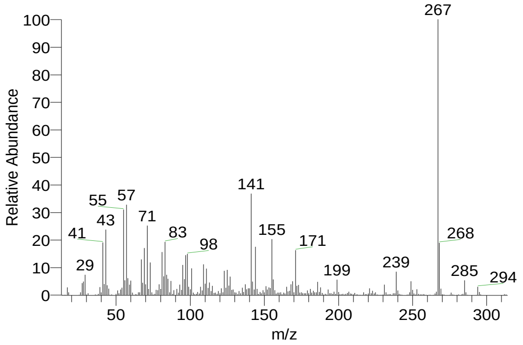

2-Hydroxy-2-octylsebacic acid
Formula C18H34O5, MW 330, CAS# 101885-38-5, Entry# 237218
2-Hydroxy-2-octyldecanedioic acid #

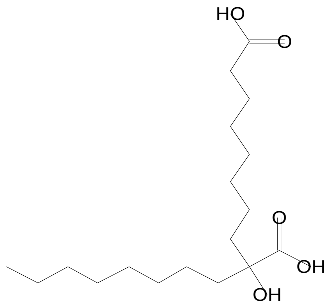

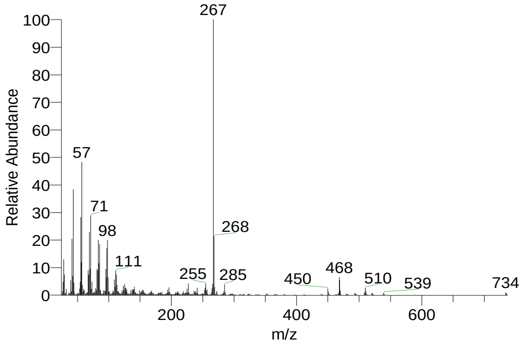

Octadecanoic acid, 1-[(tetradecyloxy)carbonyl]pentadecyl ester
Formula C48H94O4, MW 734, CAS# 69688-49-9, Entry# 237116
1-[(Tetradecyloxy)carbonyl]pentadecyl stearate #

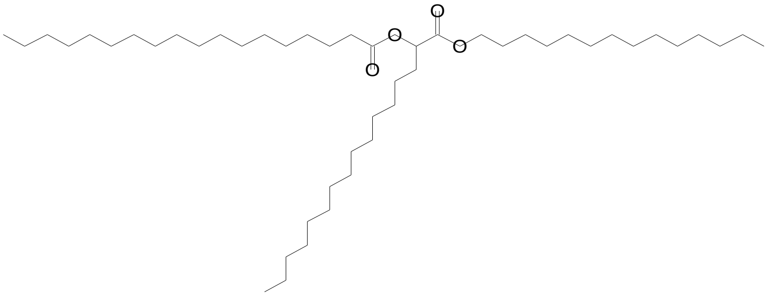

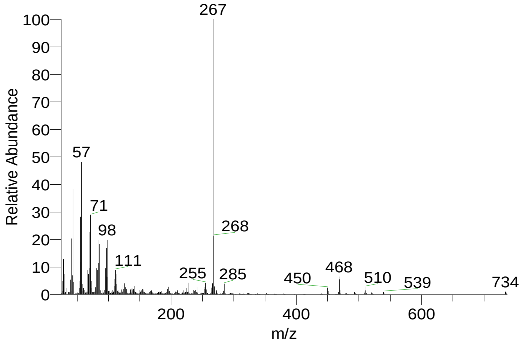

OCTADECANOIC ACID, 1-[(TETRADECYLOXY)CARBONYL]PENTADECYL ESTER
Formula C48H94O4, MW 734, CAS# 69688-49-9, Entry# 302602
1-[(TETRADECYLOXY)CARBONYL]PENTADECYL STEARATE #

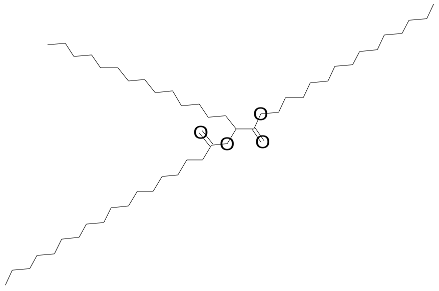

There is no signature data to report.

Library Search Report

shrefa100 #18660 RT: 68.47 AV: 1 NL: 1.72E6
T: + c EI Full ms [50.00-1000.00]

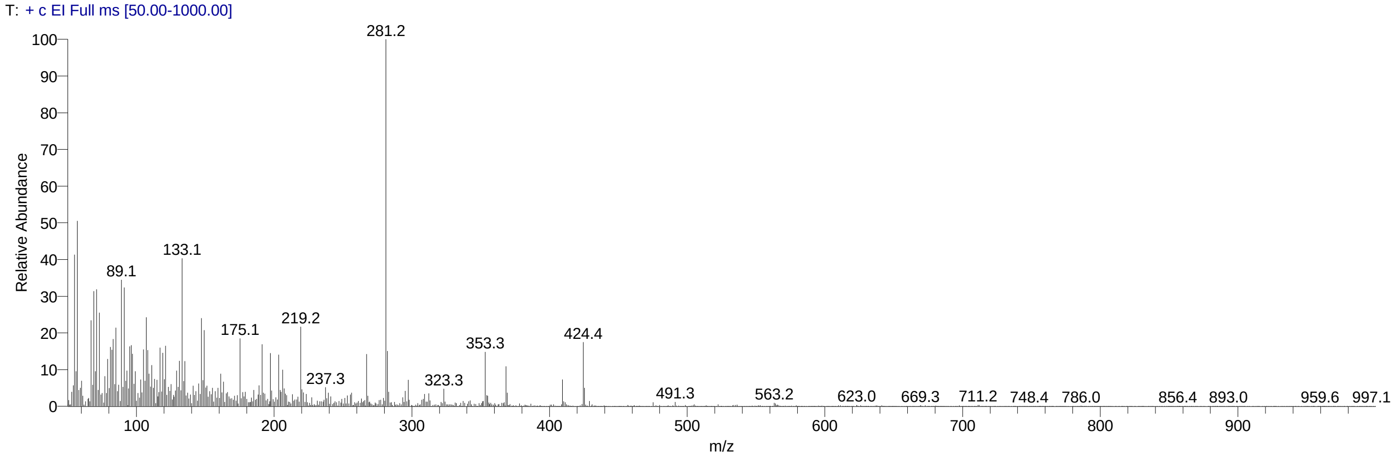

RT	Scan #	Probability	Compound Name	SI	RSI	Cas #	Area	Area %	Library
68.47	18660.000000	22.84	Astaxanthin	646	649	472-61-7	58067465.11	0.71	mainlib
68.47	18660.000000	22.84	á,á-CAROTENE-4,4'-DIONE, 3,3'-DIHYDROXY-, (3S,3'S)-	645	649	472-61-7	58067465.11	0.71	WileyRegistry8e
68.47	18660.000000	18.41	Corynan-17-ol, 18,19-didehydro-10-methoxy-, acetate (ester)	641	702	56053-13-5	58067465.11	0.71	mainlib
68.47	18660.000000	18.41	CORYNAN-17-OL, 18,19-DIDEHYDRO-10-METHOXY-, ACETATE (ESTER)	637	696	56053-13-5	58067465.11	0.71	WileyRegistry8e
68.47	18660.000000	8.94	9,19-Cyclo-27-norlanostan-25-one, 3-(acetyloxy)-24-methyl-, (3á,24R)-	622	635	83110-15-0	58067465.11	0.71	mainlib

Hit Spectrum | Compound Structure

SI 646, RSI 649, mainlib, Entry# 66455, CAS# 472-61-7, Astaxanthin

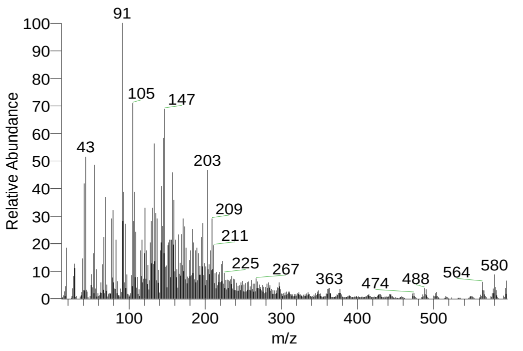

Astaxanthin
Formula C40H52O4, MW 596, CAS# 472-61-7, Entry# 66455
á,á-Carotene-4,4'-dione, 3,3'-dihydroxy-, (3S,3'S)-

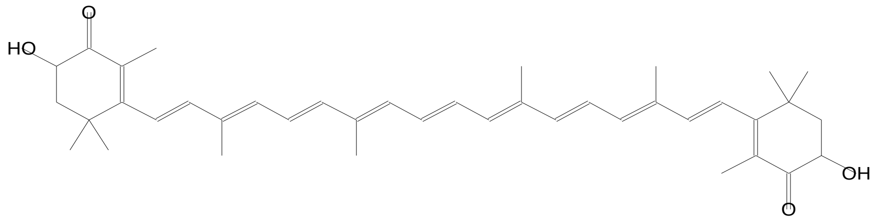

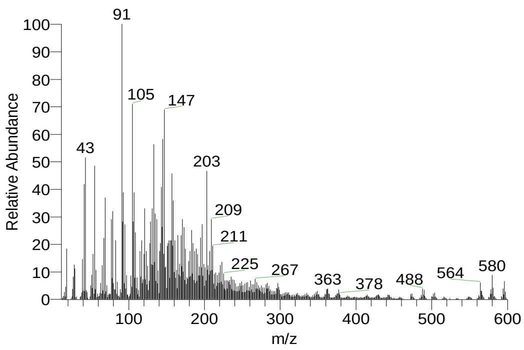

á,á-CAROTENE-4,4'-DIONE, 3,3'-DIHYDROXY-, (3S,3'S)-
Formula C40H52O4, MW 596, CAS# 472-61-7, Entry# 296446
3,3'-DIHYDROXY-BETA,BETA-CAROTENE-4,4'-DIONE #

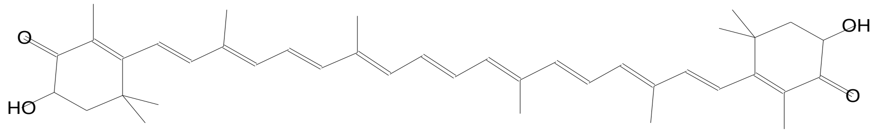

There is no signature data to report.

Library Search Report

Hit Spectrum | Compound Structure

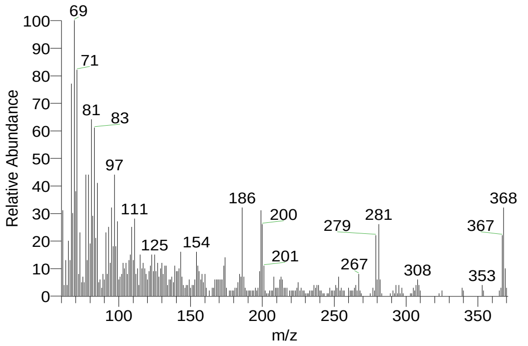

Corynan-17-ol, 18,19-didehydro-10-methoxy-, acetate (ester)
Formula C22H28N2O3, MW 368, CAS# 56053-13-5, Entry# 38043
10-Methoxycoryn-18-en-17-yl acetate #

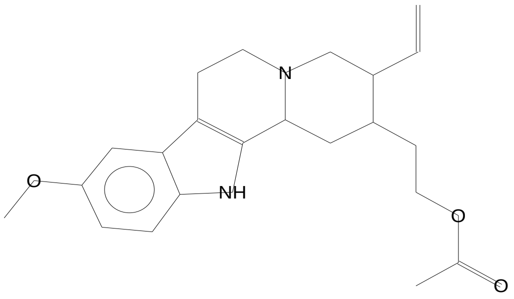

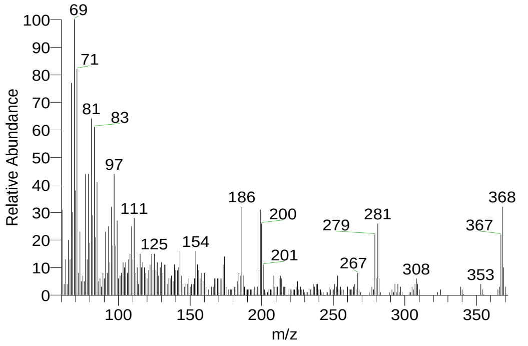

CORYNAN-17-OL, 18,19-DIDEHYDRO-10-METHOXY-, ACETATE (ESTER)
Formula C22H28N2O3, MW 368, CAS# 56053-13-5, Entry# 239814
10-METHOXYCORYN-18-EN-17-YL ACETATE #

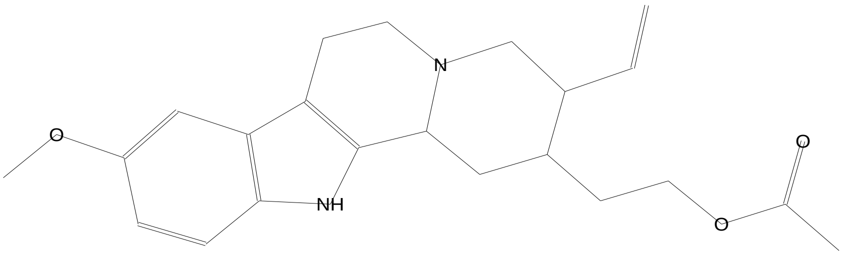

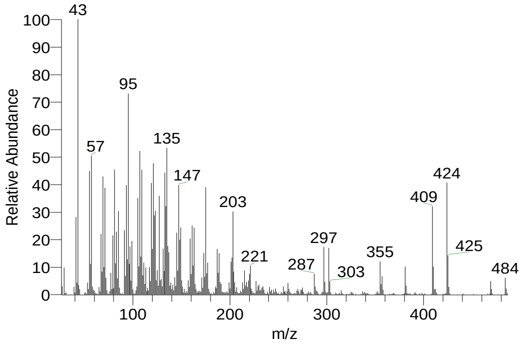

9,19-Cyclo-27-norlanostan-25-one, 3-(acetyloxy)-24-methyl-, (3á,24R)-
Formula C32H52O3, MW 484, CAS# 83110-15-0, Entry# 11201

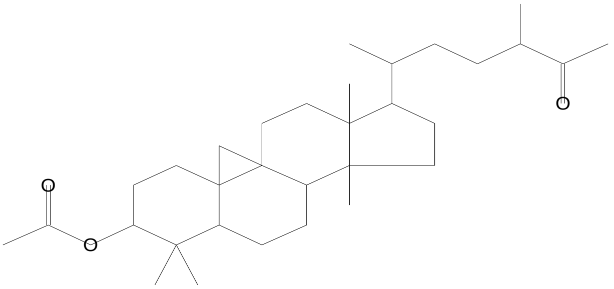

There is no signature data to report.

Library Search Report

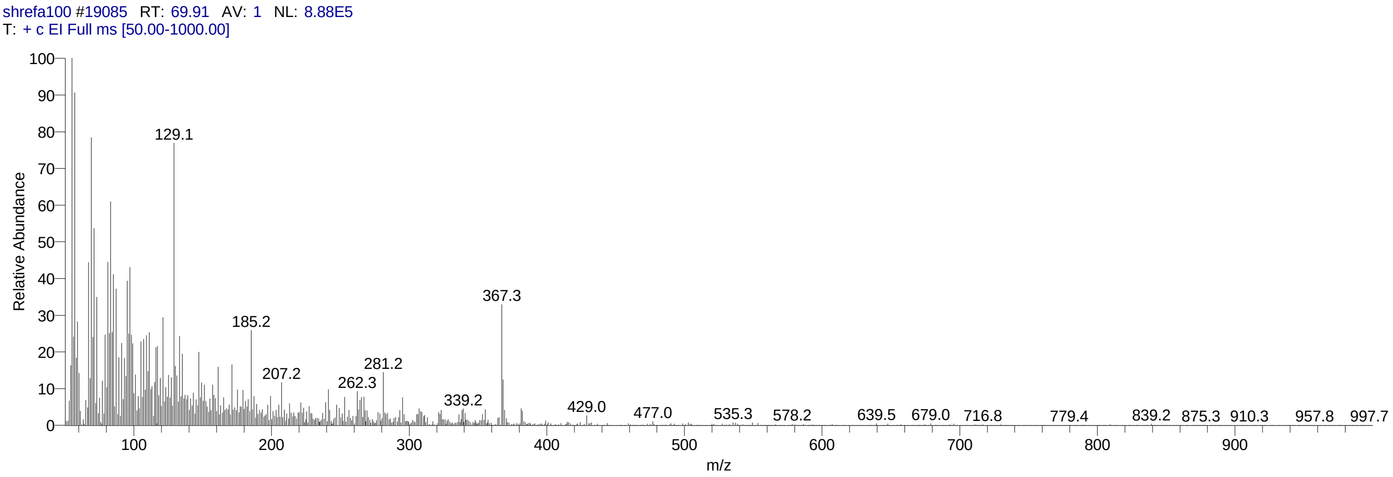

RT	Scan #	Probability	Compound Name	SI	RSI	Cas #	Area	Area %	Library
69.91	19085.000000	68.67	9-OCTADECENOIC ACID, (2-PHENYL-1,3-DIOXOLAN-4-YL)METHYL ESTER, CIS-	798	834	56599-45-2	45330349.83	0.56	WileyRegistry8e
69.91	19085.000000	6.05	Corynan-17-ol, 18,19-didehydro-10-methoxy-, acetate (ester)	711	778	56053-13-5	45330349.83	0.56	mainlib
69.91	19085.000000	6.05	CORYNAN-17-OL, 18,19-DIDEHYDRO-10-METHOXY-, ACETATE (ESTER)	709	770	56053-13-5	45330349.83	0.56	WileyRegistry8e
69.91	19085.000000	5.58	Docosanoic acid, 1,2,3-propanetriyl ester	709	726	18641-57-1	45330349.83	0.56	mainlib
69.91	19085.000000	5.58	DOCOSANOIC ACID, 1,2,3-PROPANETRIYL ESTER	709	726	18641-57-1	45330349.83	0.56	WileyRegistry8e

Hit Spectrum | Compound Structure

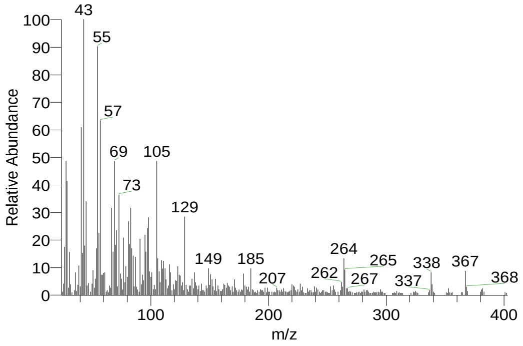

9-OCTADECENOIC ACID, (2-PHENYL-1,3-DIOXOLAN-4-YL)METHYL ESTER, CIS-
Formula C28H44O4, MW 444, CAS# 56599-45-2, Entry# 272720
(2-PHENYL-1,3-DIOXOLAN-4-YL)METHYL (9E)-9-OCTADECENOATE #

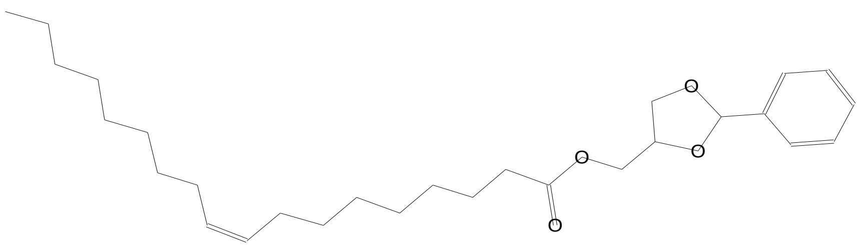

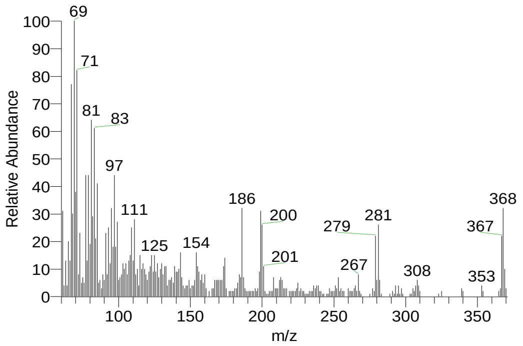

Corynan-17-ol, 18,19-didehydro-10-methoxy-, acetate (ester)
Formula C22H28N2O3, MW 368, CAS# 56053-13-5, Entry# 38043
10-Methoxycoryn-18-en-17-yl acetate #

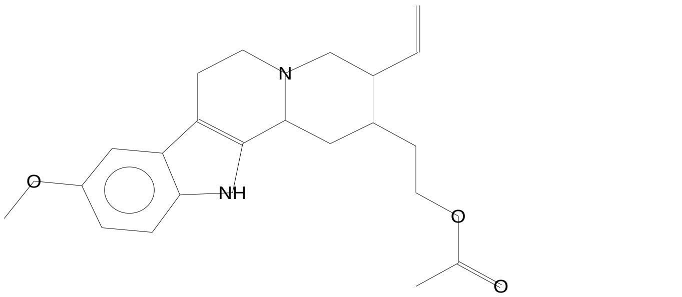

There is no signature data to report.

Library Search Report

Hit Spectrum	Compound Structure

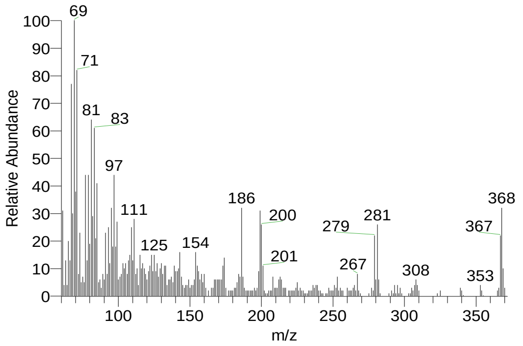

CORYNAN-17-OL, 18,19-DIDEHYDRO-10-METHOXY-, ACETATE (ESTER)
Formula C22H28N2O3, MW 368, CAS# 56053-13-5, Entry# 239814
10-METHOXYCORYN-18-EN-17-YL ACETATE #

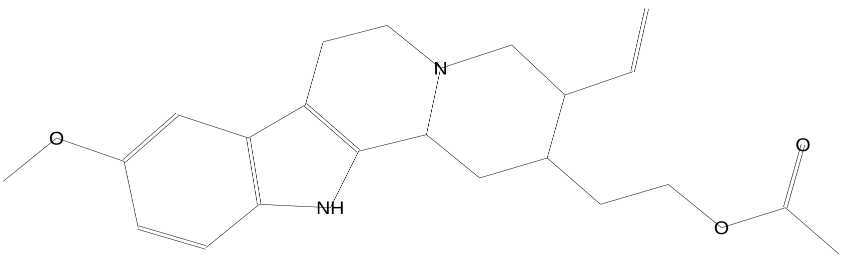

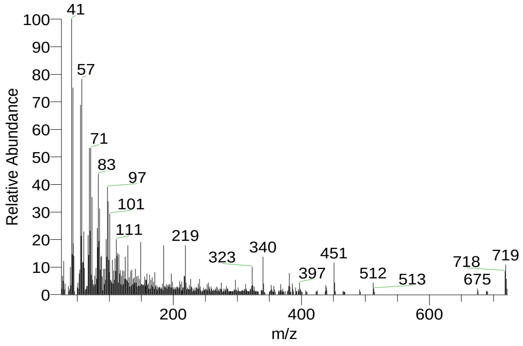

Docosanoic acid, 1,2,3-propanetriyl ester
Formula C69H134O6, MW 1058, CAS# 18641-57-1, Entry# 3322
Docosanoin, tri-

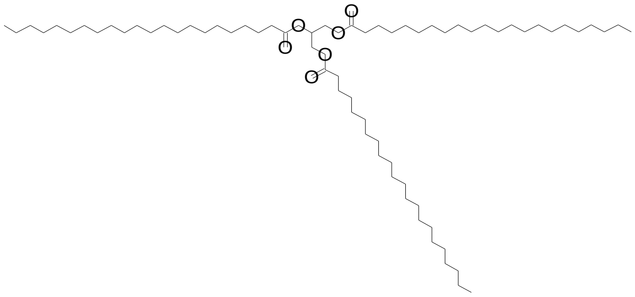

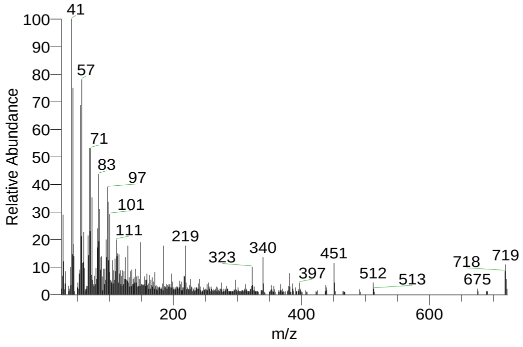

DOCOSANOIC ACID, 1,2,3-PROPANETRIYL ESTER
Formula C69H134O6, MW 1058, CAS# 18641-57-1, Entry# 305117
2,3-BIS(DOCOSANOYLOXY)PROPYL DOCOSANOATE #

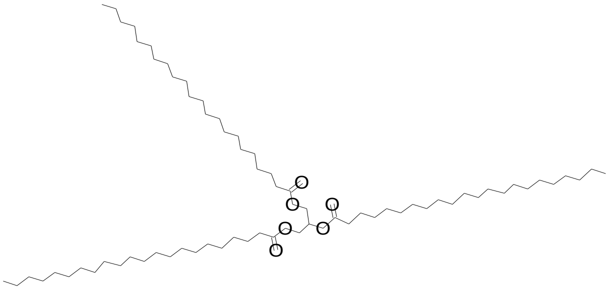

There is no signature data to report.

Library Search Report

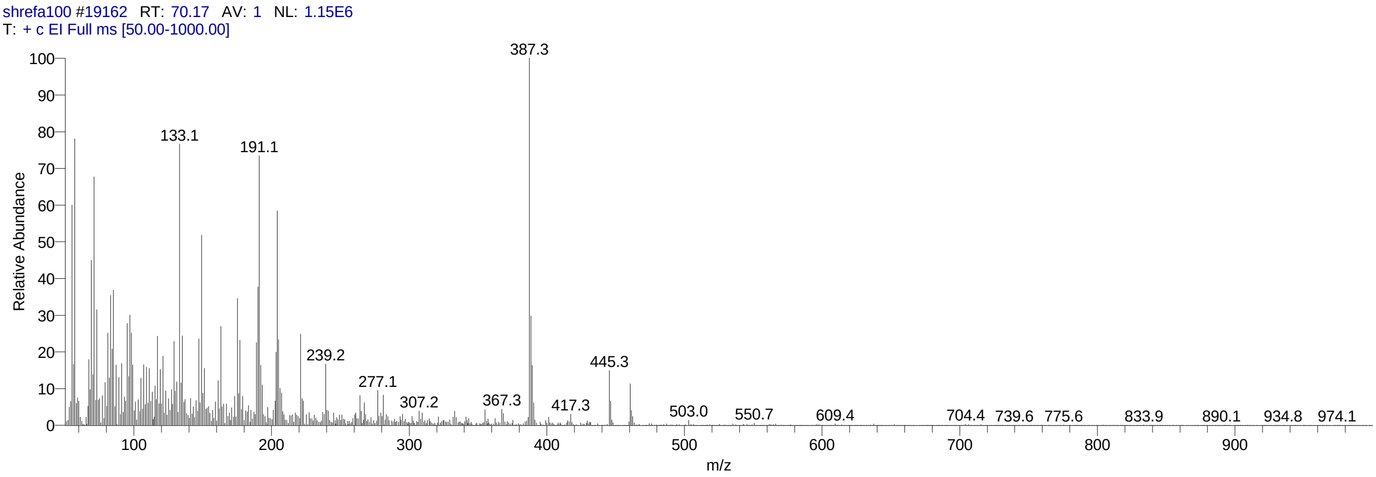

RT	Scan #	Probability	Compound Name	SI	RSI	Cas #	Area	Area %	Library
70.17	19162.000000	66.22	2-(5,12-DIHYDROXY-4A,6A-DIMETHYL-2-OXO-8-PROPYL-2,4A,4B,5,6,6A,9A,10,10A,10B,11,12-DODECAHYDRO-6BH-NAPHTHO[2',1':4,5]INDENO[1,2-D][1,3]DIOXOL-6B-YL)-2-OXOETHYL ACETATE #	681	693	93789-66-3	57890754.82	0.71	WileyRegistry8e
70.17	19162.000000	66.22	(22S)-21-Acetoxy-6à,11á-dihydroxy-16à,17à-propylmethylenedioxypregna-1,4-diene-3,20-dione	680	693	93789-66-3	57890754.82	0.71	mainlib
70.17	19162.000000	19.12	(22S)-21-Acetoxy-6á,11á-dihydroxy-16à,17à-propylmethylenedioxypregna-1,4-diene-3,20-dione	653	666	93789-69-6	57890754.82	0.71	mainlib
70.17	19162.000000	19.12	2-(5,12-DIHYDROXY-4A,6A-DIMETHYL-2-OXO-8-PROPYL-2,4A,4B,5,6,6A,9A,10,10A,10B,11,12-DODECAHYDRO-6BH-NAPHTHO[2',1':4,5]INDENO[1,2-D][1,3]DIOXOL-6B-YL)-2-OXOETHYL ACETATE #	653	664	93789-69-6	57890754.82	0.71	WileyRegistry8e
70.17	19162.000000	6.40	2,2-DIMETHYL-3-OXA-5à-CHOLESTANE	629	837	83626-01-1	57890754.82	0.71	WileyRegistry8e

Hit Spectrum | Compound Structure

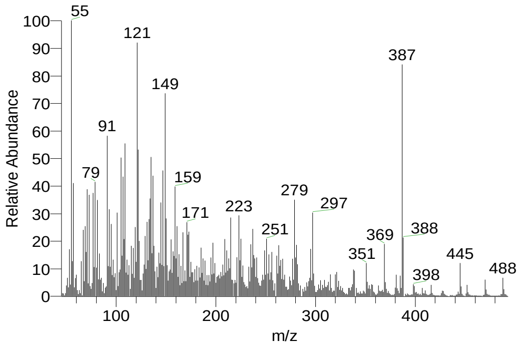

Formula C27H36O8, MW 488, CAS# 93789-66-3, Entry# 283468

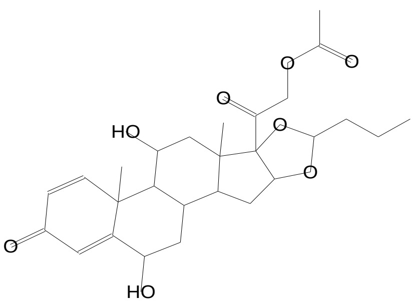

There is no signature data to report.

Library Search Report

Hit Spectrum | Compound Structure

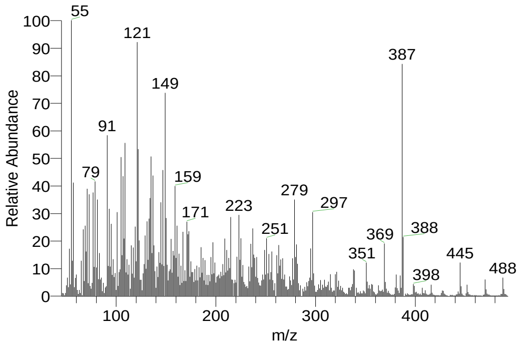

(22S)-21-Acetoxy-6à,11á-dihydroxy-16à,17à-propylmethylenedioxypregna-1,4-diene-3,20-dione
Formula C27H36O8, MW 488, CAS# 93789-66-3, Entry# 23153

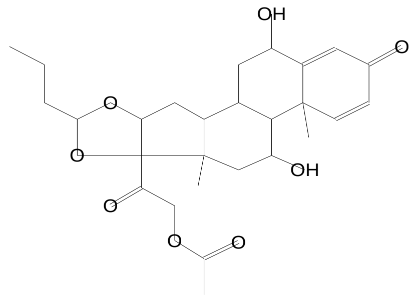

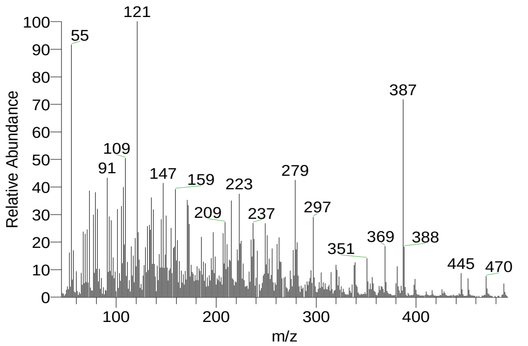

(22S)-21-Acetoxy-6á,11á-dihydroxy-16à,17à-propylmethylenedioxypregna-1,4-diene-3,20-dione
Formula C27H36O8, MW 488, CAS# 93789-69-6, Entry# 111800

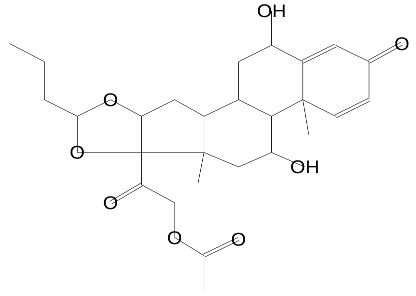

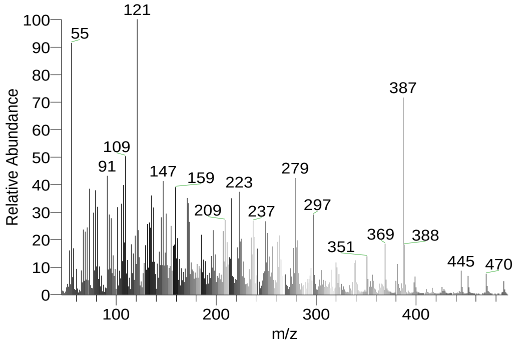

Formula C27H36O8, MW 488, CAS# 93789-69-6, Entry# 283467

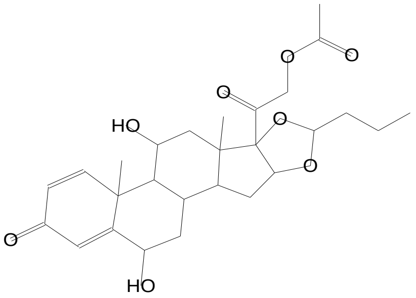

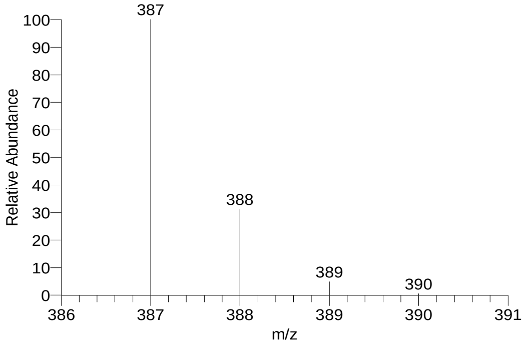

2,2-DIMETHYL-3-OXA-5à-CHOLESTANE
Formula C28H50O, MW 402, CAS# 83626-01-1, Entry# 257320

There is no signature data to report.

Library Search Report

shrefa100 #19289 RT: 70.60 AV: 1 NL: 7.00E6
T: + c EI Full ms [50.00-1000.00]

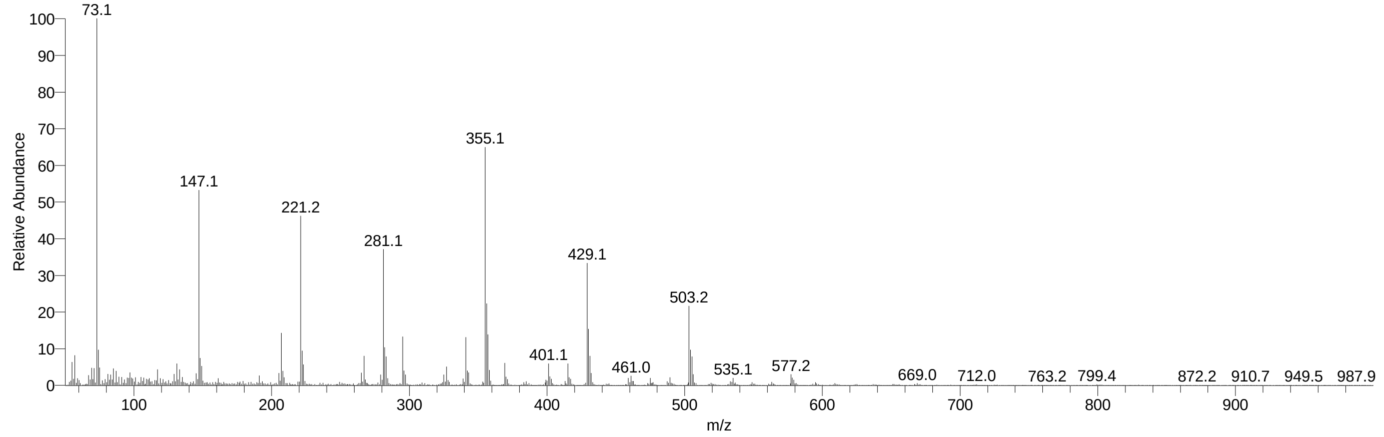

RT	Scan #	Probability	Compound Name	SI	RSI	Cas #	Area	Area %	Library
70.60	19289.000000	26.53	SILICONE OIL	797	852	NA	211924162.03	2.59	WileyRegistry8e
70.60	19289.000000	26.53	SILIKONFETT SE30 (GREVELS)	797	852	NA	211924162.03	2.59	WileyRegistry8e
70.60	19289.000000	22.41	1H-PURIN-6-AMINE, [(2-FLUOROPHENYL)METHYL]-	793	853	74421-44-6	211924162.03	2.59	WileyRegistry8e
70.60	19289.000000	20.68	Cyclodecasiloxane, eicosamethyl-	791	835	18772-36-6	211924162.03	2.59	mainlib
70.60	19289.000000	20.68	2,2,4,4,6,6,8,8,10,10,12,12,14,14,16,16,18,18,20,20-ICOSAMETHYLCYCLODECASILOXANE #	791	835	18772-36-6	211924162.03	2.59	WileyRegistry8e

Hit Spectrum	Compound Structure

SILICONE OIL
Formula , MW 0, CAS# NA, Entry# 305490
SILIKONFETT SE30 (GREVELS)

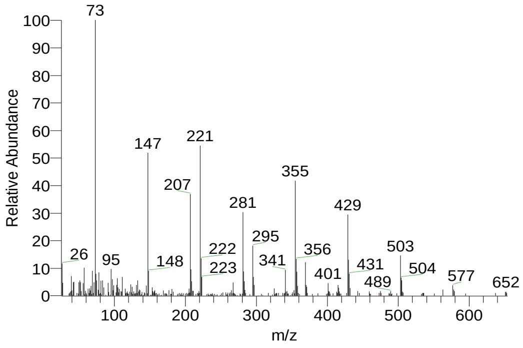

SILIKONFETT SE30 (GREVELS)
Formula , MW 0, CAS# NA, Entry# 392776

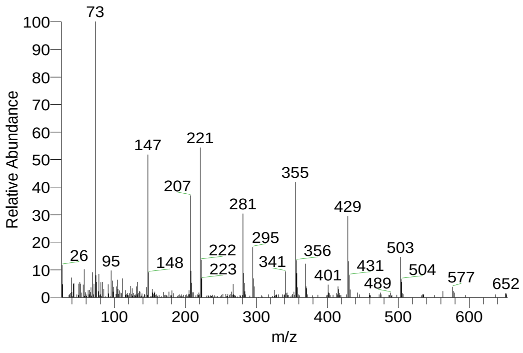

There is no signature data to report.

Library Search Report

Hit Spectrum	Compound Structure

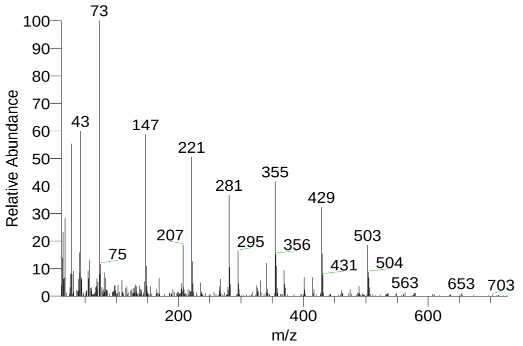

1H-PURIN-6-AMINE, [(2-FLUOROPHENYL)METHYL]-
Formula C12H10FN5, MW 243, CAS# 74421-44-6, Entry# 132518

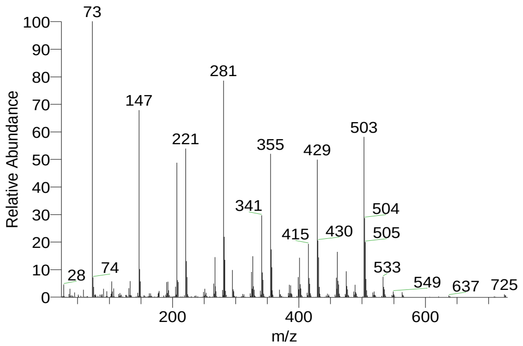

Cyclodecasiloxane, eicosamethyl-
Formula C20H60O10Si10, MW 740, CAS# 18772-36-6, Entry# 47864
2,2,4,4,6,6,8,8,10,10,12,12,14,14,16,16,18,18,20,20-Icosamethylcyclodecasiloxane #

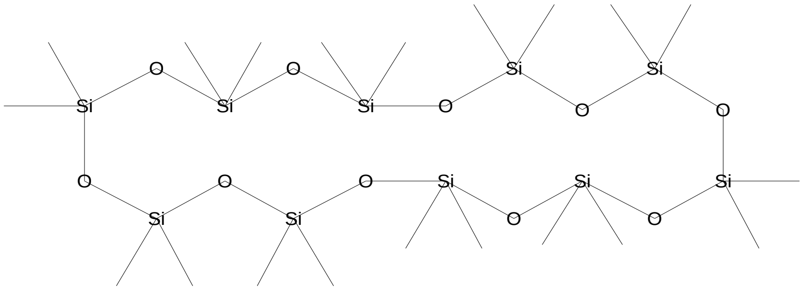

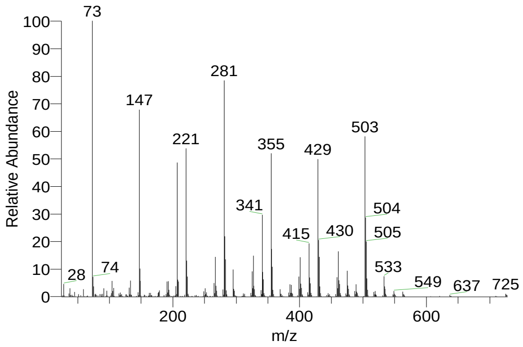

2,2,4,4,6,6,8,8,10,10,12,12,14,14,16,16,18,18,20,20-ICOSAMETHYLCYCLODECASILOXANE #
Formula C20H60O10Si10, MW 740, CAS# 18772-36-6, Entry# 380233
2,2,4,4,6,6,8,8,10,10,12,12,14,14,16,16,18,18,20,20-ICOSAMETHYLCYCLODECASILOXANE

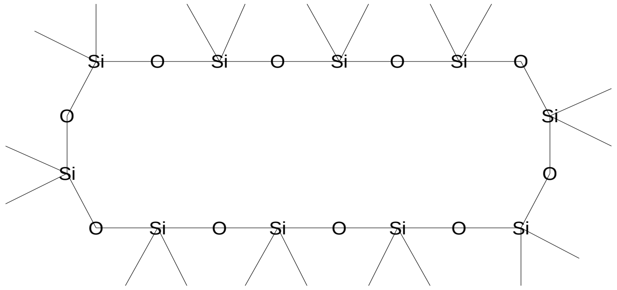

There is no signature data to report.

Library Search Report

shrefa100 #19304 RT: 70.66 AV: 1 NL: 2.37E6
T: + c EI Full ms [50.00-1000.00]

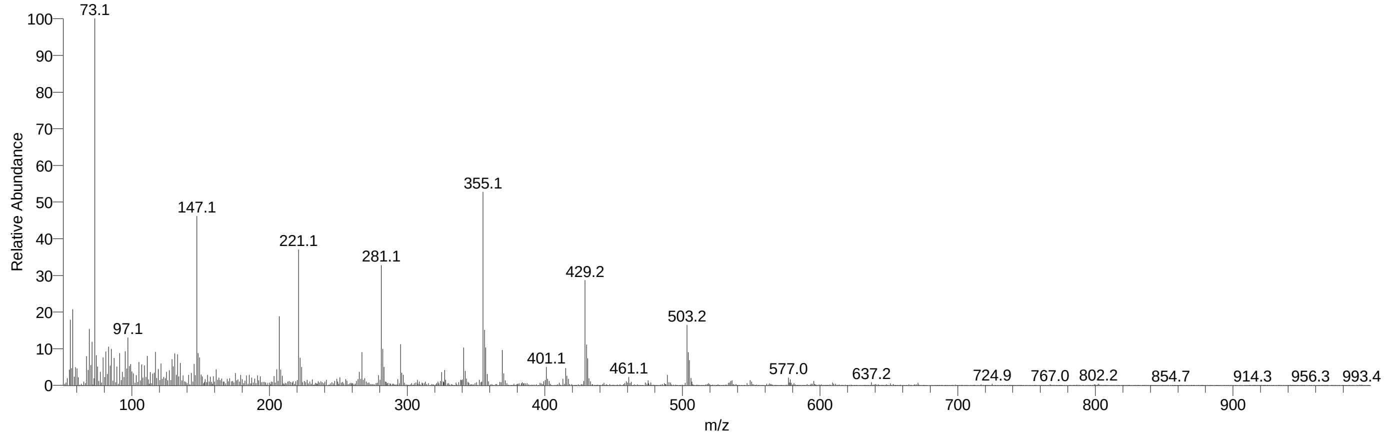

RT	Scan #	Probability	Compound Name	SI	RSI	Cas #	Area	Area %	Library
70.66	19304.000000	33.69	SILIKONFETT SE30 (GREVELS)	760	845	NA	69068346.31	0.85	WileyRegistry8e
70.66	19304.000000	33.69	SILICONE OIL	760	844	NA	69068346.31	0.85	WileyRegistry8e
70.66	19304.000000	23.78	1H-PURIN-6-AMINE, [(2-FLUOROPHENYL)METHYL]-	750	844	74421-44-6	69068346.31	0.85	WileyRegistry8e
70.66	19304.000000	4.93	Cyclodecasiloxane, eicosamethyl-	702	778	18772-36-6	69068346.31	0.85	mainlib
70.66	19304.000000	4.93	2,2,4,4,6,6,8,8,10,10,12,12,14,14,16,16,18,18,20,20-ICOSAMETHYLCYCLODECASILOXANE #	702	778	18772-36-6	69068346.31	0.85	WileyRegistry8e

Hit Spectrum | Compound Structure

SILIKONFETT SE30 (GREVELS)
Formula , MW 0, CAS# NA, Entry# 392776

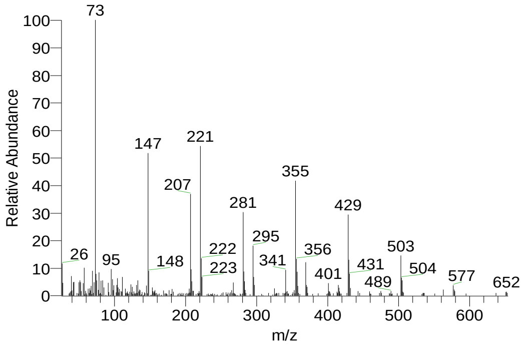

SILICONE OIL
Formula , MW 0, CAS# NA, Entry# 305490
SILIKONFETT SE30 (GREVELS)

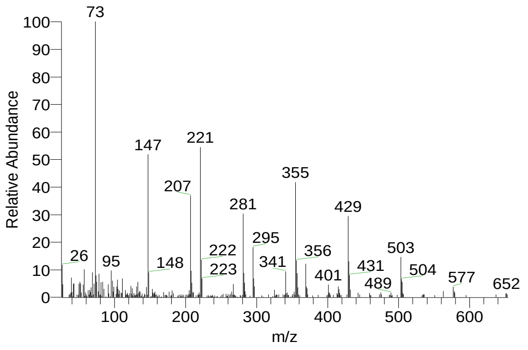

There is no signature data to report.

Library Search Report

Hit Spectrum | Compound Structure

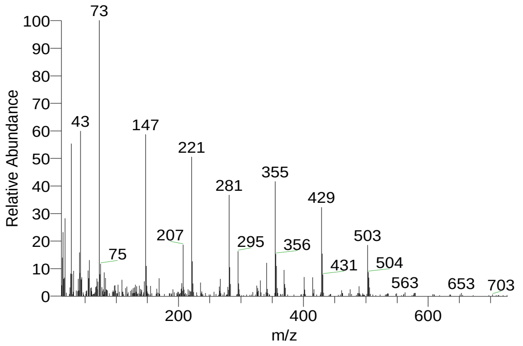

1H-PURIN-6-AMINE, [(2-FLUOROPHENYL)METHYL]-
Formula C12H10FN5, MW 243, CAS# 74421-44-6, Entry# 132518

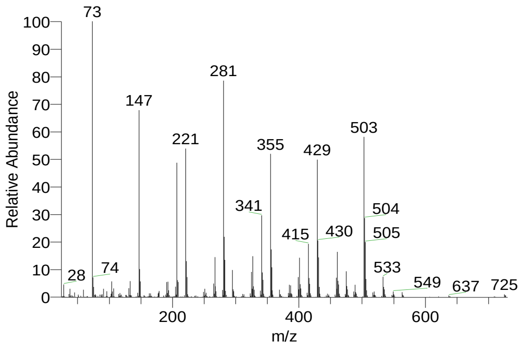

Cyclodecasiloxane, eicosamethyl-
Formula C20H60O10Si10, MW 740, CAS# 18772-36-6, Entry# 47864
2,2,4,4,6,6,8,8,10,10,12,12,14,14,16,16,18,18,20,20-Icosamethylcyclodecasiloxane #

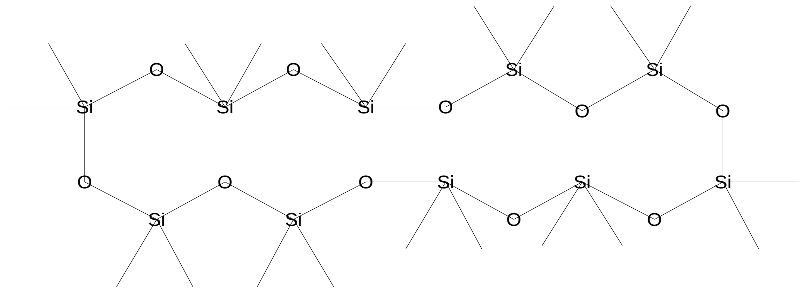

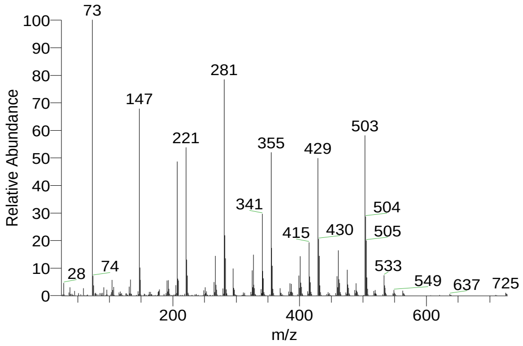

2,2,4,4,6,6,8,8,10,10,12,12,14,14,16,16,18,18,20,20-ICOSAMETHYLCYCLODECASILOXANE #
Formula C20H60O10Si10, MW 740, CAS# 18772-36-6, Entry# 380233
2,2,4,4,6,6,8,8,10,10,12,12,14,14,16,16,18,18,20,20-ICOSAMETHYLCYCLODECASILOXANE

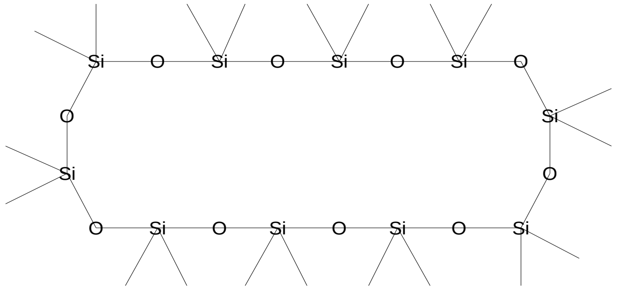

There is no signature data to report.

Library Search Report

shrefa100 #19454 RT: 71.17 AV: 1 NL: 7.71E5
T: + c EI Full ms [50.00-1000.00]

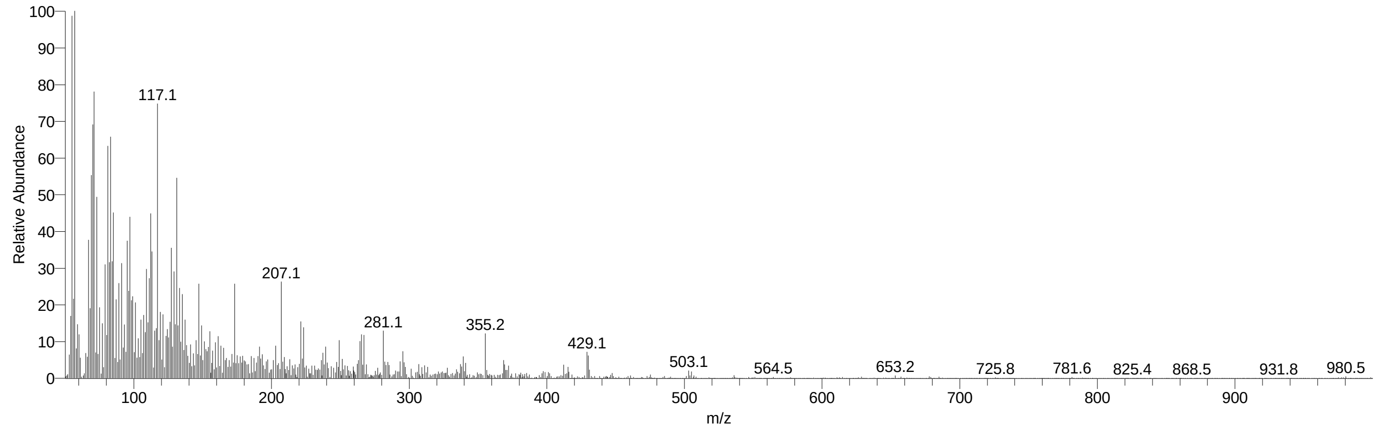

RT	Scan #	Probability	Compound Name	SI	RSI	Cas #	Area	Area %	Library
71.17	19454.000000	32.53	4H-1-BENZOPYRAN-4-ONE, 2-(3,4-DIHYDROXYPHENYL)-6,8-DI-á-D-GLUCOPYRANOSYL-5,7-DIHYDROXY-	731	747	29428-58-8	39115710.39	0.48	WileyRegistry8e
71.17	19454.000000	11.88	9,12,15-OCTADECATRIENOIC ACID, 2,3-BIS[(TRIMETHYLSILYL)OXY]PROPYL ESTER, (Z,Z,Z)-	708	786	55521-22-7	39115710.39	0.48	WileyRegistry8e
71.17	19454.000000	11.88	HAHNFETT	708	734	NA	39115710.39	0.48	WileyRegistry8e
71.17	19454.000000	11.88	HAHNFETT	708	734	NA	39115710.39	0.48	WileyRegistry8e
71.17	19454.000000	6.84	9-OCTADECENOIC ACID (Z)-, 2-[(TRIMETHYLSILYL)OXY]-1-[[(TRIMETHYLSILYL)OXY]METHYL]ETHYL ESTER	692	752	54284-48-9	39115710.39	0.48	WileyRegistry8e

Hit Spectrum

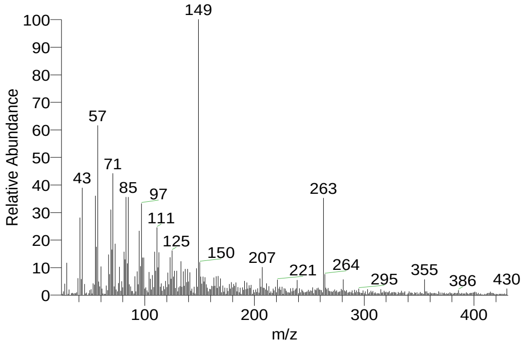

Compound Structure

Formula C27H30O16, MW 610, CAS# 29428-58-8, Entry# 297453
6,8-DI-C-á-GLUCOSYLLUTEOLIN

There is no signature data to report.

Library Search Report

Hit Spectrum | Compound Structure

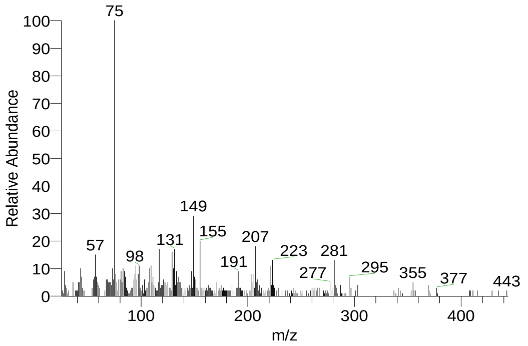

9,12,15-OCTADECATRIENOIC ACID, 2,3-BIS[(TRIMETHYLSILYL)OXY]PROPYL ESTER, (Z,Z,Z)-
Formula C27H52O4Si2, MW 496, CAS# 55521-22-7, Entry# 284834
2,3-BIS[(TRIMETHYLSILYL)OXY]PROPYL (9E,12E,15E)-9,12,15-OCTADECATRIENOATE #

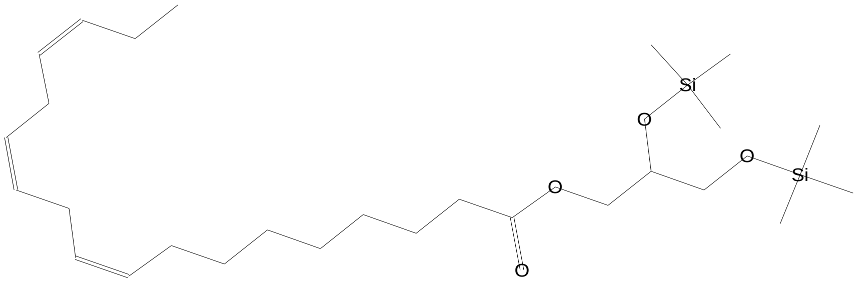

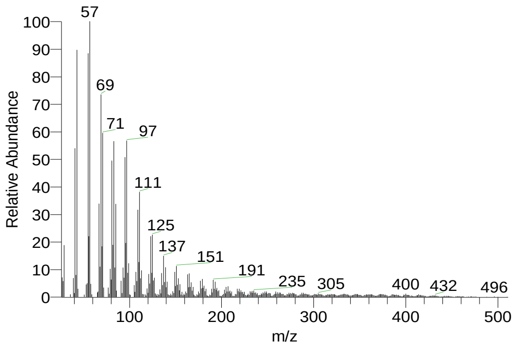

HAHNFETT
Formula , MW 0, CAS# NA, Entry# 305496

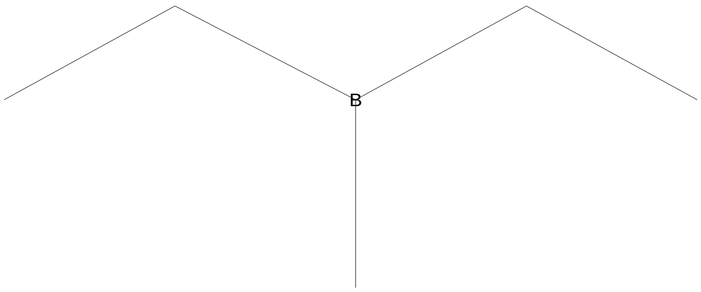

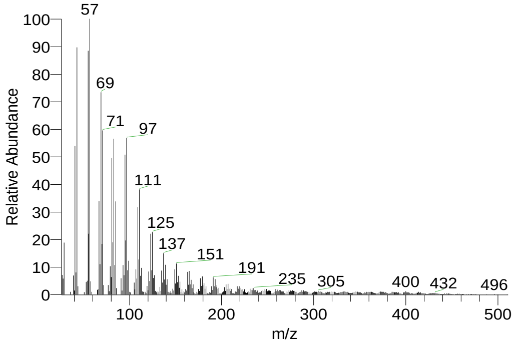

HAHNFETT
Formula , MW 0, CAS# NA, Entry# 391160

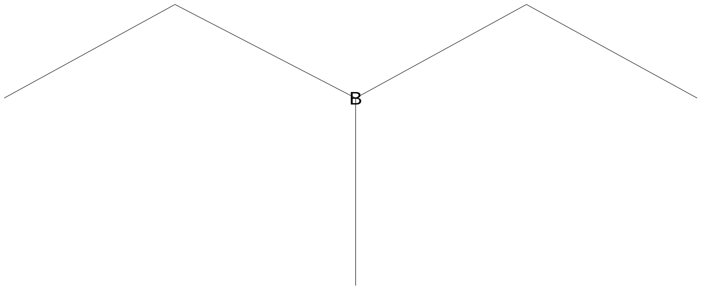

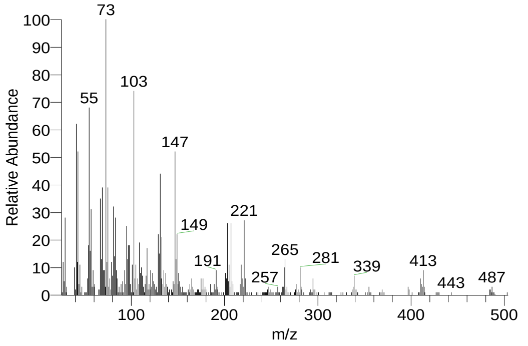

Formula C27H56O4Si2, MW 500, CAS# 54284-48-9, Entry# 285456
2-MONOOLEOYLGLYCEROL TRIMETHYLSILYL ETHER

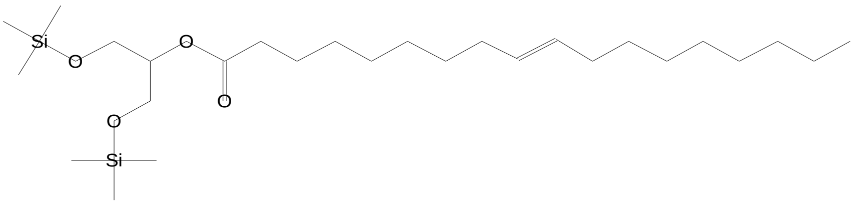

There is no signature data to report.

Library Search Report

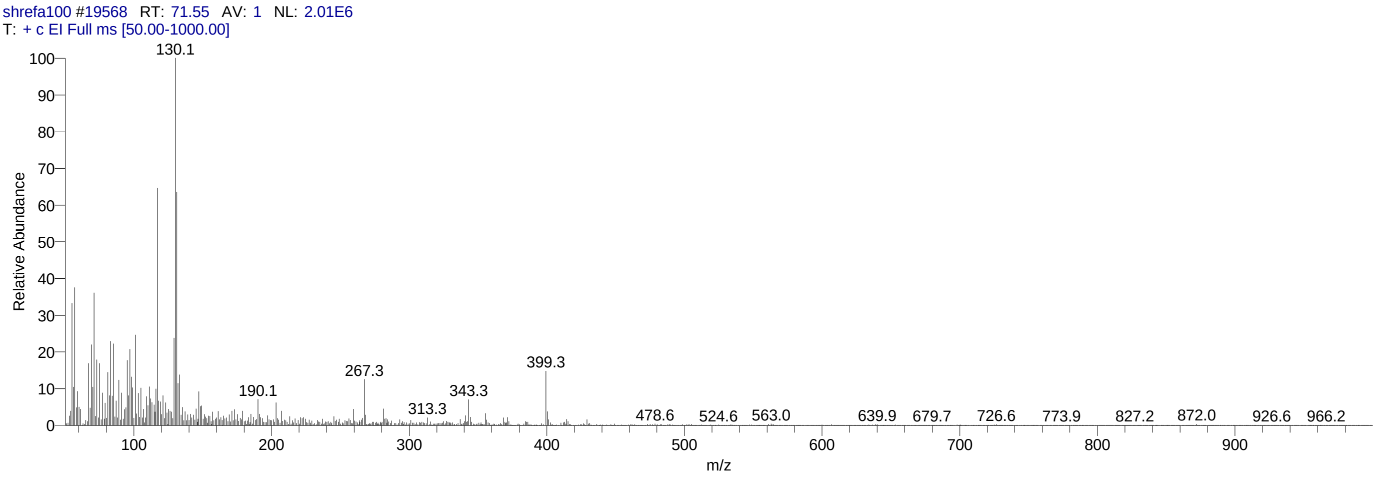

RT	Scan #	Probability	Compound Name	SI	RSI	Cas #	Area	Area %	Library
71.55	19568.000000	43.94	1,25-Dihydroxyvitamin D3, TMS derivative	638	734	55759-94-9	51531136.33	0.63	mainlib
71.55	19568.000000	43.94	9,10-SECOCHOLESTA-5,7,10(19)-TRIENE-1,3-DIOL, 25-[(TRIMETHYLSILYL)OXY]-, (3á,5Z,7E)-	638	734	55759-94-9	51531136.33	0.63	WileyRegistry8e
71.55	19568.000000	9.27	17-(1,5-Dimethylhexyl)-10,13-dimethyl-4-vinylhexadecahydrocyclopenta[a]phenanthren-3-ol	592	623	NA	51531136.33	0.63	mainlib
71.55	19568.000000	9.27	17-(1,5-DIMETHYL-HEXYL)-10,13-DIMETHYL-4-VINYL-HEXADECAHYDRO-CYCLOPENTA[A]PHENANTHREN-3-OL	592	623	NA	51531136.33	0.63	WileyRegistry8e
71.55	19568.000000	5.06	2,3-BIS[(TRIMETHYLSILYL)OXY]PROPYL STEARATE #	575	620	68000-49-7	51531136.33	0.63	WileyRegistry8e

Hit Spectrum	Compound Structure

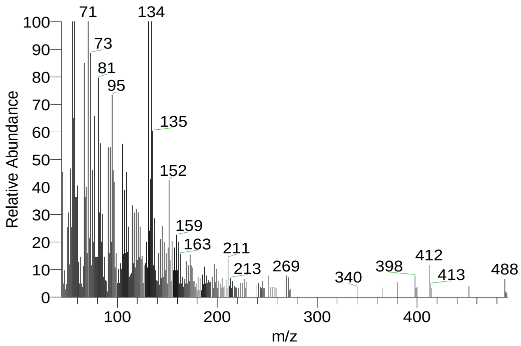

1,25-Dihydroxyvitamin D3, TMS derivative
Formula C30H52O3Si, MW 488, CAS# 55759-94-9, Entry# 21585
9,10-Secocholesta-5,7,10(19)-triene-1,3-diol, 25-[(trimethylsilyl)oxy]-, (3á,5Z,7E)-

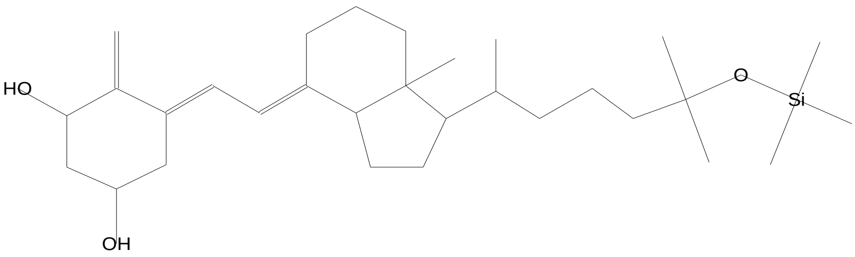

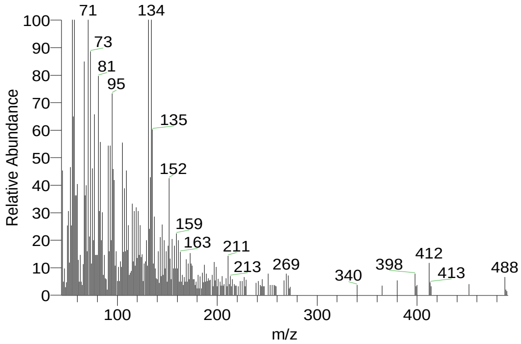

9,10-SECOCHOLESTA-5,7,10(19)-TRIENE-1,3-DIOL, 25-[(TRIMETHYLSILYL)OXY]-, (3á,5Z,7E)-
Formula C30H52O3Si, MW 488, CAS# 55759-94-9, Entry# 283552
(5E,7E)-25-[(TRIMETHYLSILYL)OXY]-9,10-SECOCHOLESTA-5,7,10-TRIENE-1,3-DIOL #

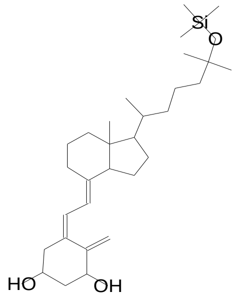

There is no signature data to report.

Library Search Report

Hit Spectrum | Compound Structure

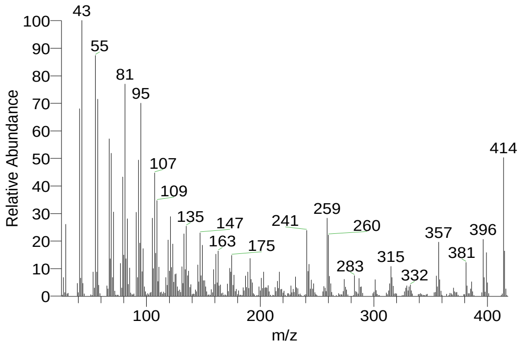

17-(1,5-Dimethylhexyl)-10,13-dimethyl-4-vinylhexadecahydrocyclopenta[a]phenanthren-3-ol
Formula C29H50O, MW 414, CAS# NA, Entry# 7869
4-Vinylcholestan-3-ol #

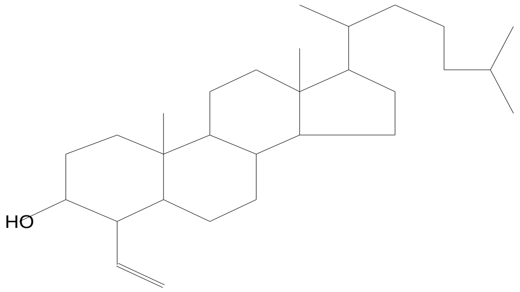

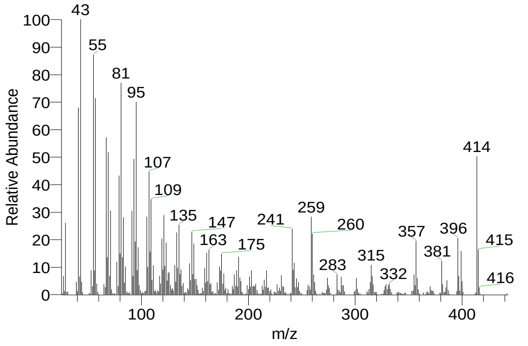

Formula C29H50O, MW 414, CAS# NA, Entry# 361448
4-VINYLCHOLESTAN-3-OL

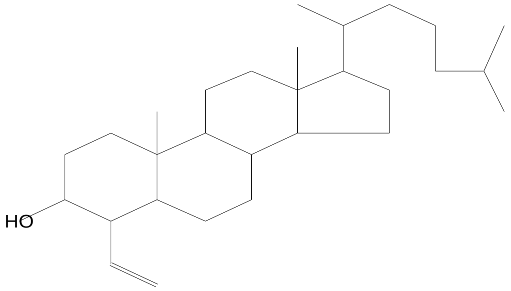

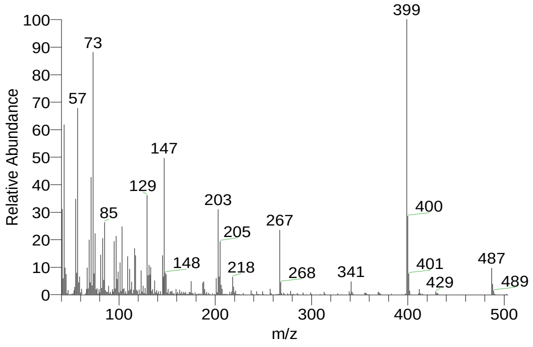

2,3-BIS[(TRIMETHYLSILYL)OXY]PROPYL STEARATE #
Formula C27H58O4Si2, MW 502, CAS# 68000-49-7, Entry# 285796
2,3-BIS[(TRIMETHYLSILYL)OXY]PROPYL STEARATE

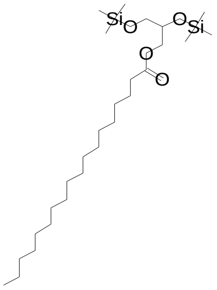

There is no signature data to report.

Library Search Report

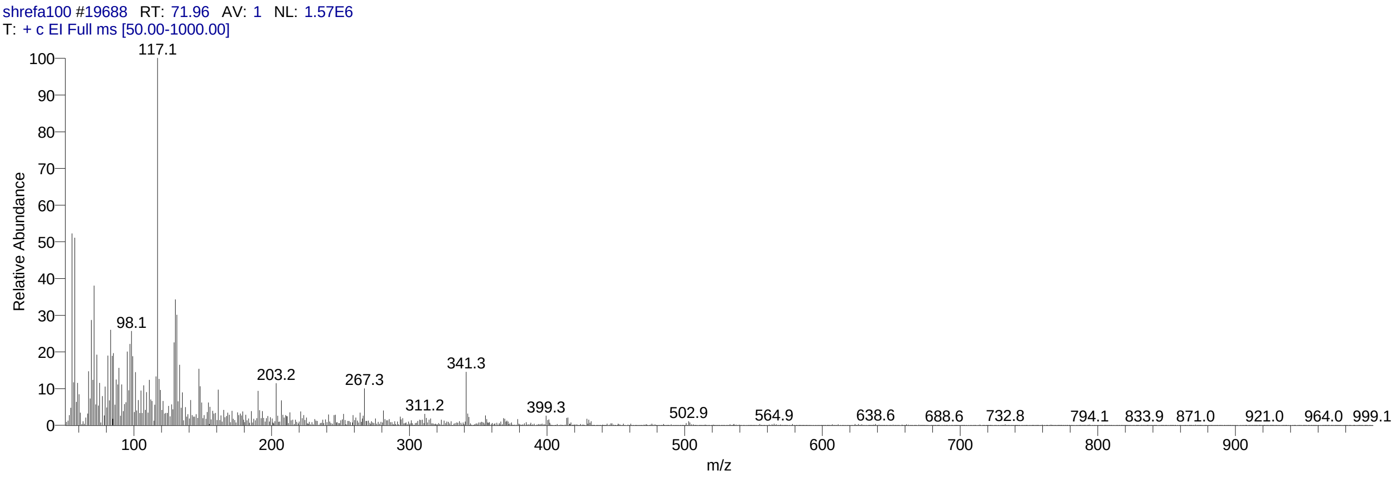

RT	Scan #	Probability	Compound Name	SI	RSI	Cas #	Area	Area %	Library
71.96	19688.000000	34.59	1,25-Dihydroxyvitamin D3, TMS derivative	627	746	55759-94-9	50665088.62	0.62	mainlib
71.96	19688.000000	34.59	9,10-SECOCHOLESTA-5,7,10(19)-TRIENE-1,3-DIOL, 25-[(TRIMETHYLSILYL)OXY]-, (3á,5Z,7E)-	627	746	55759-94-9	50665088.62	0.62	WileyRegistry8e
71.96	19688.000000	13.67	1H-Cyclopenta[a]phenanthrene-7-carboxylic acid, 2,3,6,7,8,9,10,11,12,13,14,15,16,17-tetradecahydro-10,13-dimethyl-3-oxo-17,2'-spiro(5-oxotetrahydrofuran)-, ethyl ester	605	625	NA	50665088.62	0.62	mainlib
71.96	19688.000000	13.67	1H-CYCLOPENTA[A]PHENANTHRENE-7-CARBOXYLIC ACID, 2,3,6,7,8,9,10,11,12,13,14,15,16,17-TETRADECAHYDRO-10,13-DIMETHYL-3-OXO-17,2'-SPIRO(5-OXOTETRAHYDROFURANE)-, ETHYL ESTER	605	625	NA	50665088.62	0.62	WileyRegistry8e
71.96	19688.000000	7.46	Glycine, N-[(3à,5á)-24-oxo-3-[(trimethylsilyl)oxy]cholan-24-yl]-, methyl ester	588	675	57326-15-5	50665088.62	0.62	mainlib

Hit Spectrum

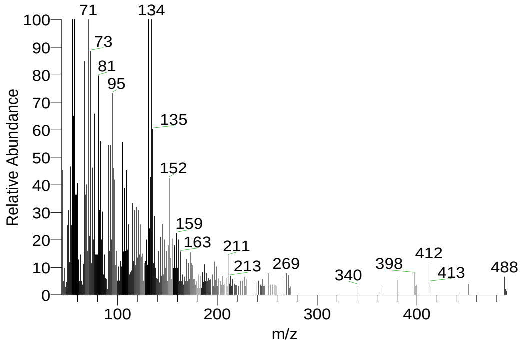

Compound Structure

1,25-Dihydroxyvitamin D3, TMS derivative
Formula C30H52O3Si, MW 488, CAS# 55759-94-9, Entry# 21585
9,10-Secocholesta-5,7,10(19)-triene-1,3-diol, 25-[(trimethylsilyl)oxy]-, (3á,5Z,7E)-

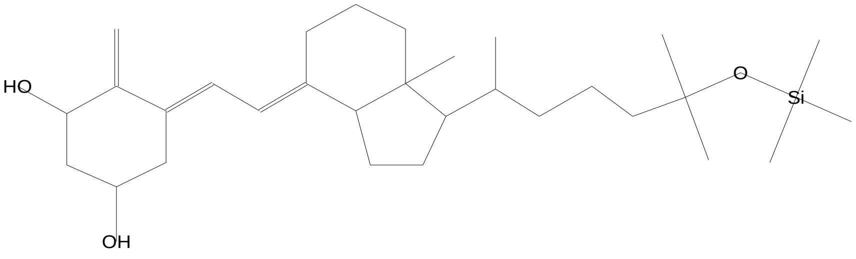

There is no signature data to report.

Library Search Report

Hit Spectrum	Compound Structure

9,10-SECOCHOLESTA-5,7,10(19)-TRIENE-1,3-DIOL, 25-[(TRIMETHYLSILYL)OXY]-, (3á,5Z,7E)-
Formula C30H52O3Si, MW 488, CAS# 55759-94-9, Entry# 283552
(5E,7E)-25-[(TRIMETHYLSILYL)OXY]-9,10-SECOCHOLESTA-5,7,10-TRIENE-1,3-DIOL #

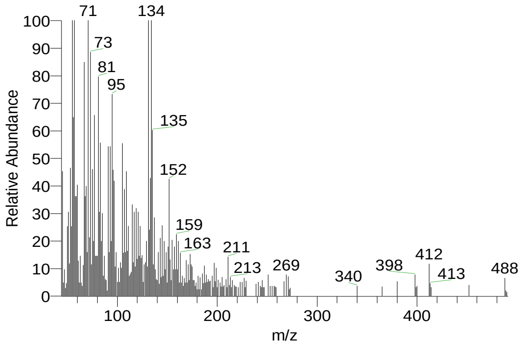

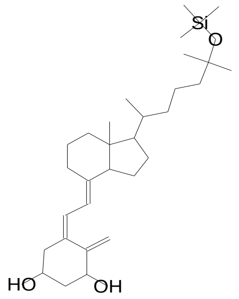

Formula C25H34O5, MW 414, CAS# NA, Entry# 263655
$:28VQTNMTIQLQDEAC-UHFFFAOYSA-N

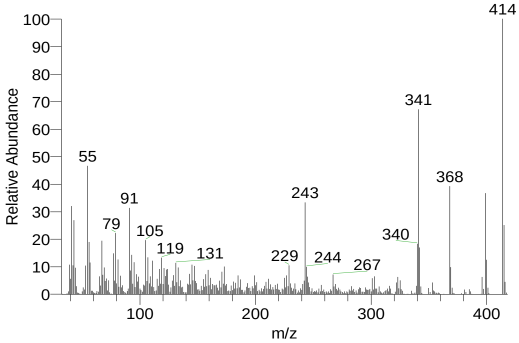

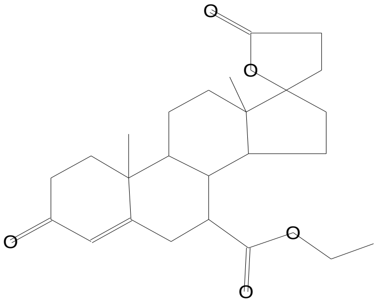

Formula C25H34O5, MW 414, CAS# NA, Entry# 305567

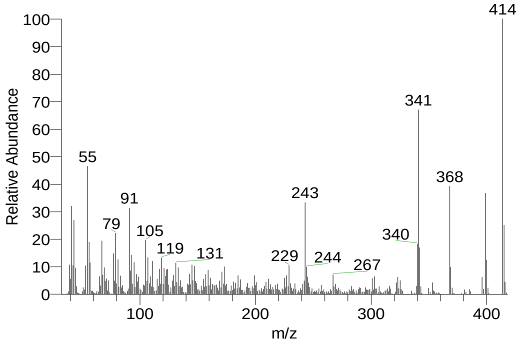

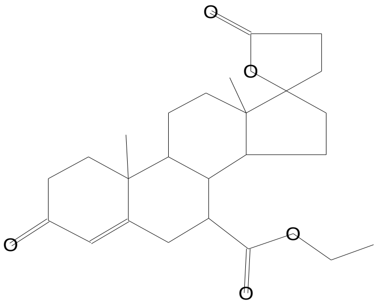

Glycine, N-[(3à,5á)-24-oxo-3-[(trimethylsilyl)oxy]cholan-24-yl]-, methyl ester
Formula C30H53NO4Si, MW 519, CAS# 57326-15-5, Entry# 126328
Methyl ((24-oxo-3-[(trimethylsilyl)oxy]cholan-24-yl)amino)acetate #

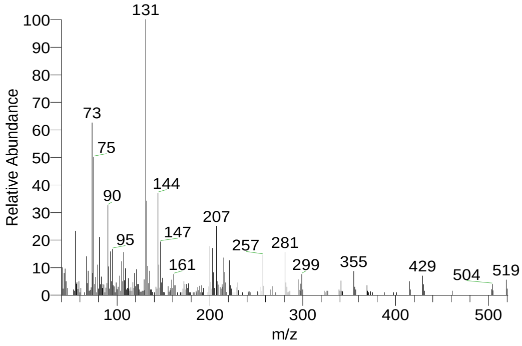

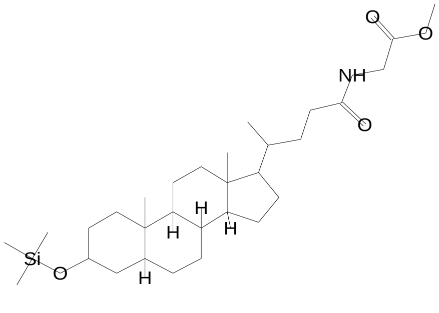

There is no signature data to report.

Library Search Report

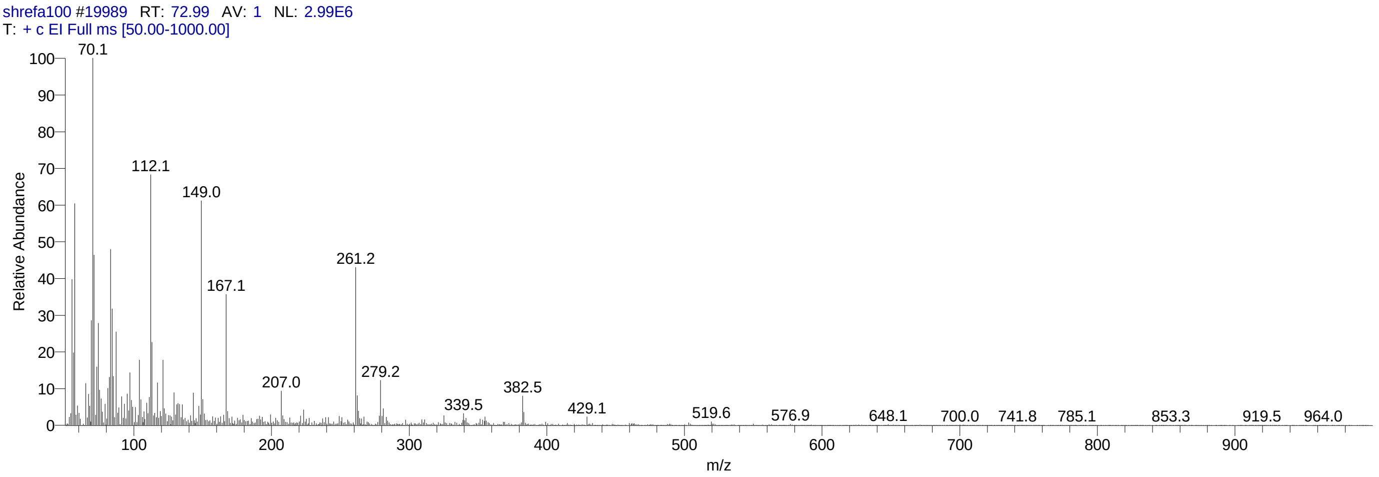

RT	Scan #	Probability	Compound Name	SI	RSI	Cas #	Area	Area %	Library
72.99	19989.000000	75.75	4H-1-BENZOPYRAN-4-ONE, 2-(3,4-DIHYDROXYPHENYL)-6,8-DI-á-D-GLUCOPYRANOSYL-5,7-DIHYDROXY-	683	695	29428-58-8	98541482.44	1.21	WileyRegistry8e
72.99	19989.000000	6.54	1,3-Benzenedicarboxylic acid, bis(2-ethylhexyl) ester	594	749	137-89-3	98541482.44	1.21	mainlib
72.99	19989.000000	6.54	1,3-Benzenedicarboxylic acid, bis(2-ethylhexyl) ester	590	769	137-89-3	98541482.44	1.21	replib
72.99	19989.000000	4.88	1,4-Benzenedicarboxylic acid, bis(2-ethylhexyl) ester	586	724	6422-86-2	98541482.44	1.21	replib
72.99	19989.000000	3.54	9-(2',2'-Dimethylpropanoilhydrazono)-3,6-dichloro-2,7-bis-[2-(diethylamino)-ethoxy]fluorene	577	713	NA	98541482.44	1.21	mainlib

Hit Spectrum	Compound Structure

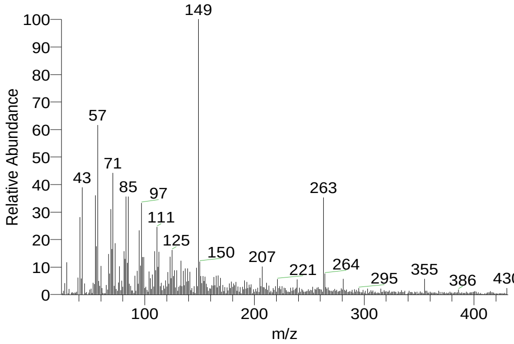

Formula C27H30O16, MW 610, CAS# 29428-58-8, Entry# 297453
6,8-DI-C-á-GLUCOSYLLUTEOLIN

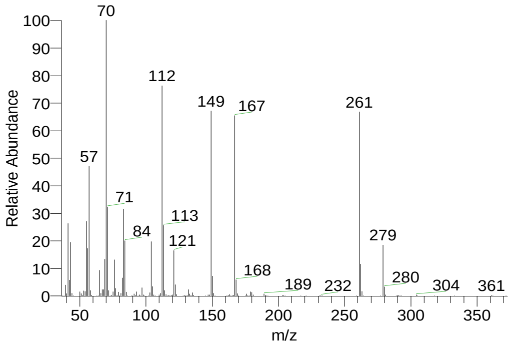

1,3-Benzenedicarboxylic acid, bis(2-ethylhexyl) ester
Formula C24H38O4, MW 390, CAS# 137-89-3, Entry# 40786
Bis(2-ethylhexyl) isophthalate

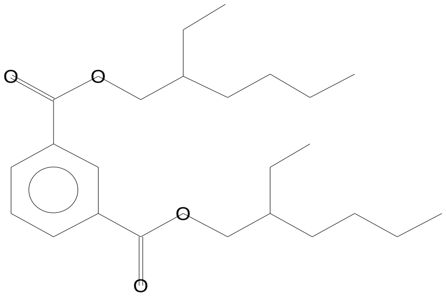

There is no signature data to report.

Library Search Report

Hit Spectrum	Compound Structure

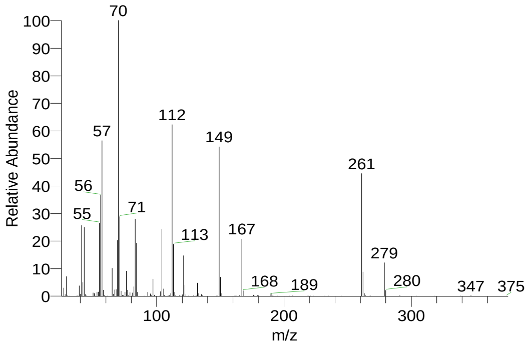

1,3-Benzenedicarboxylic acid, bis(2-ethylhexyl) ester
Formula C24H38O4, MW 390, CAS# 137-89-3, Entry# 10410
Bis(2-ethylhexyl) isophthalate

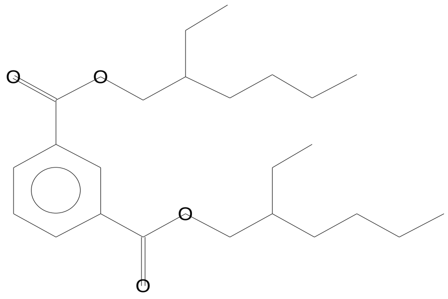

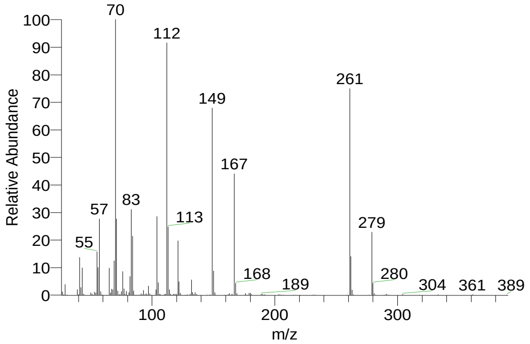

1,4-Benzenedicarboxylic acid, bis(2-ethylhexyl) ester
Formula C24H38O4, MW 390, CAS# 6422-86-2, Entry# 10413
Terephthalic acid, bis(2-ethylhexyl)ester

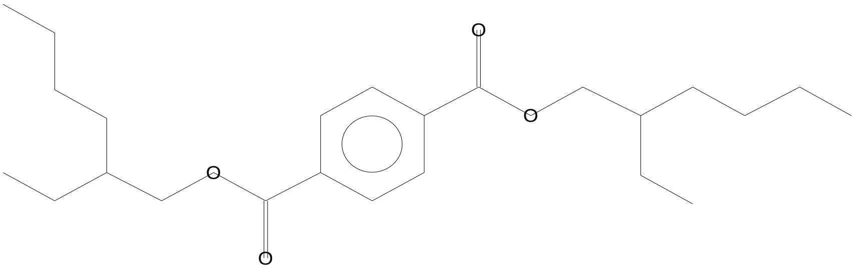

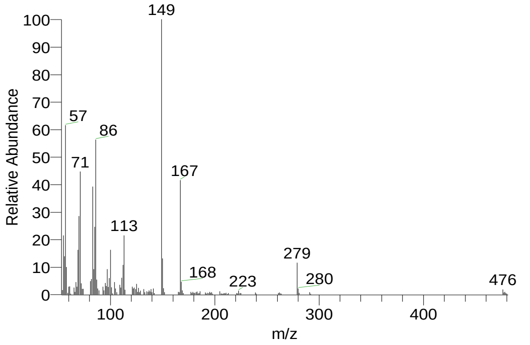

9-(2',2'-Dimethylpropanoilhydrazono)-3,6-dichloro-2,7-bis-[2-(diethylamino)-ethoxy]fluorene
Formula C30H42Cl2N4O3, MW 576, CAS# NA, Entry# 150355

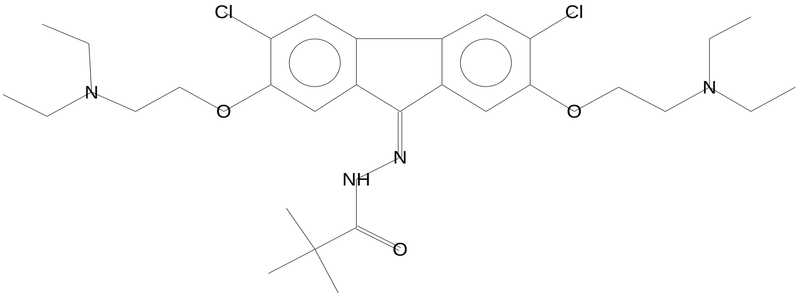

There is no signature data to report.

Library Search Report

shrefa100 #20070 RT: 73.26 AV: 1 NL: 8.88E5
T: + c EI Full ms [50.00-1000.00]

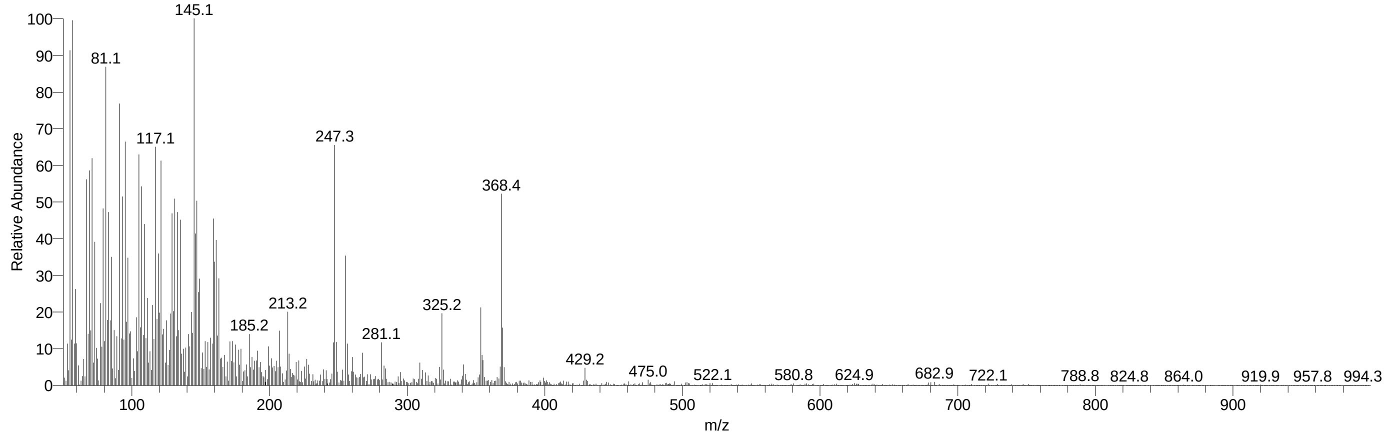

RT	Scan #	Probability	Compound Name	SI	RSI	Cas #	Area	Area %	Library
73.26	20070.000000	14.75	CHOLEST-5-EN-3-YL STEARATE	775	861	NA	69498383.76	0.85	WileyRegistry8e
73.26	20070.000000	8.49	Cholesterol margarate	759	792	24365-37-5	69498383.76	0.85	mainlib
73.26	20070.000000	7.50	CHOLEST-5-EN-3-YL PALMITATE	756	820	NA	69498383.76	0.85	WileyRegistry8e
73.26	20070.000000	7.50	CHOLEST-5-EN-3-YL BENZOATE	756	820	604-32-0	69498383.76	0.85	WileyRegistry8e
73.26	20070.000000	6.92	CHOLEST-5-EN-3-OL (3á)-, ACETATE	754	779	604-35-3	69498383.76	0.85	WileyRegistry8e

Hit Spectrum | Compound Structure

CHOLEST-5-EN-3-YL STEARATE
Formula C45H80O2, MW 652, CAS# NA, Entry# 359228
CHOLESTERINSTEARAT

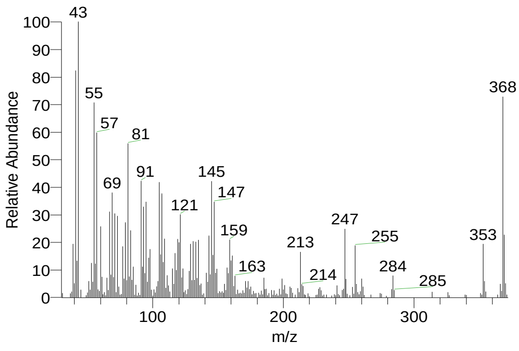

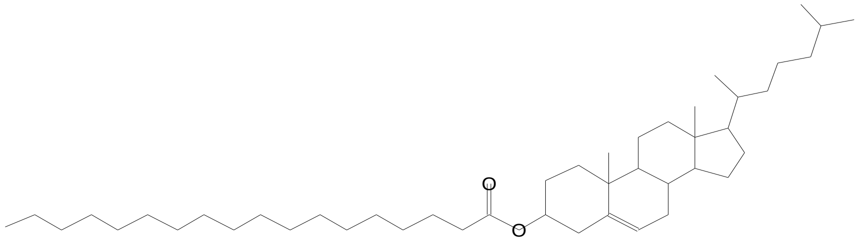

Cholesterol margarate
Formula C44H78O2, MW 638, CAS# 24365-37-5, Entry# 16039

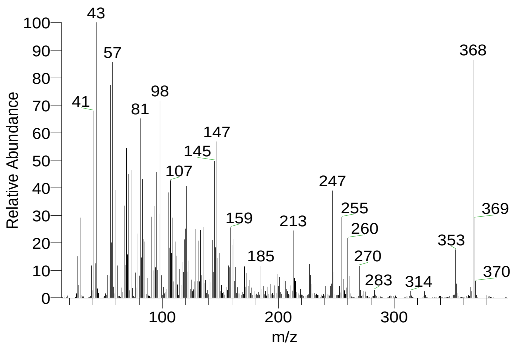

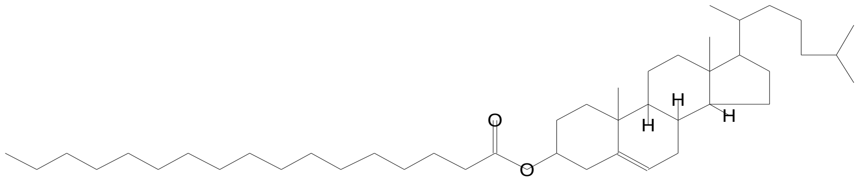

There is no signature data to report.

Library Search Report

Hit Spectrum | Compound Structure

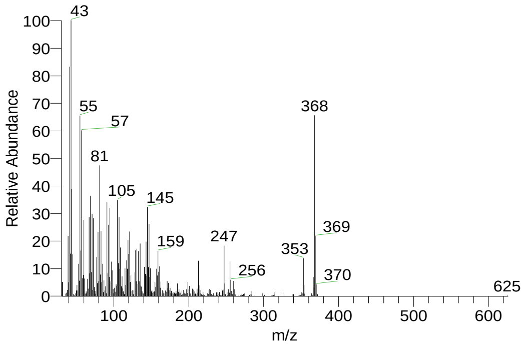

CHOLEST-5-EN-3-YL PALMITATE
Formula C43H76O2, MW 624, CAS# NA, Entry# 359227
CHOLESTERINPALMITAT

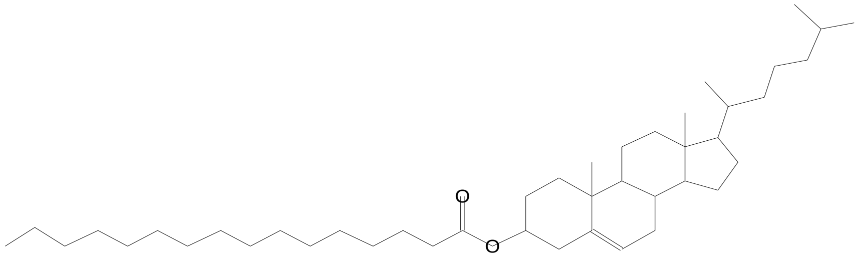

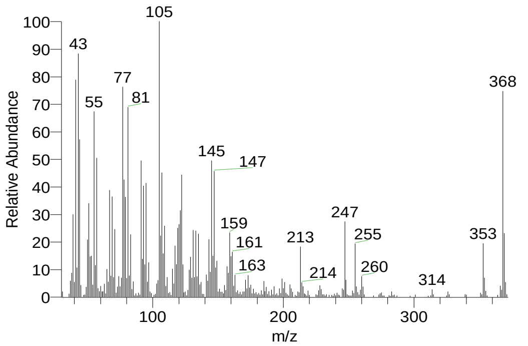

CHOLEST-5-EN-3-YL BENZOATE
Formula C34H50O2, MW 490, CAS# 604-32-0, Entry# 359229
CHOLESTERINBENZOAT

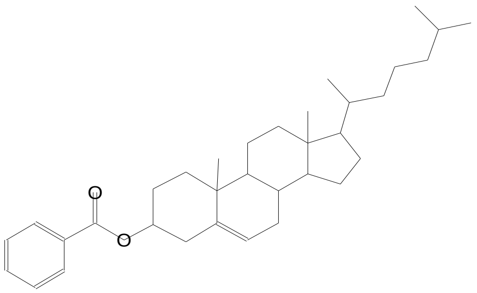

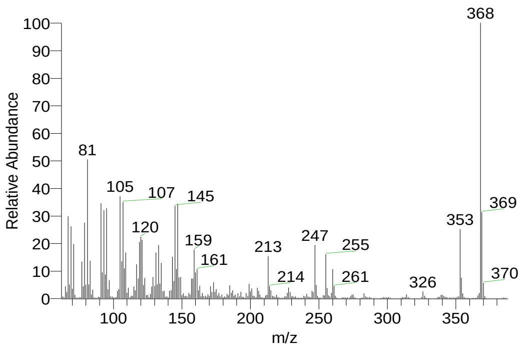

CHOLEST-5-EN-3-OL (3á)-, ACETATE
Formula C29H48O2, MW 428, CAS# 604-35-3, Entry# 267497
CHOLEST-5-EN-3-YL ACETATE #

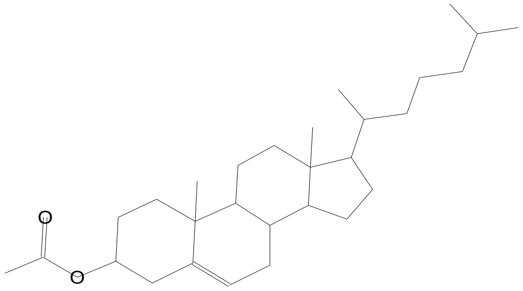

There is no signature data to report.

Library Search Report

shrefa100 #20100 RT: 73.36 AV: 1 NL: 2.28E6
T: + c EI Full ms [50.00-1000.00]

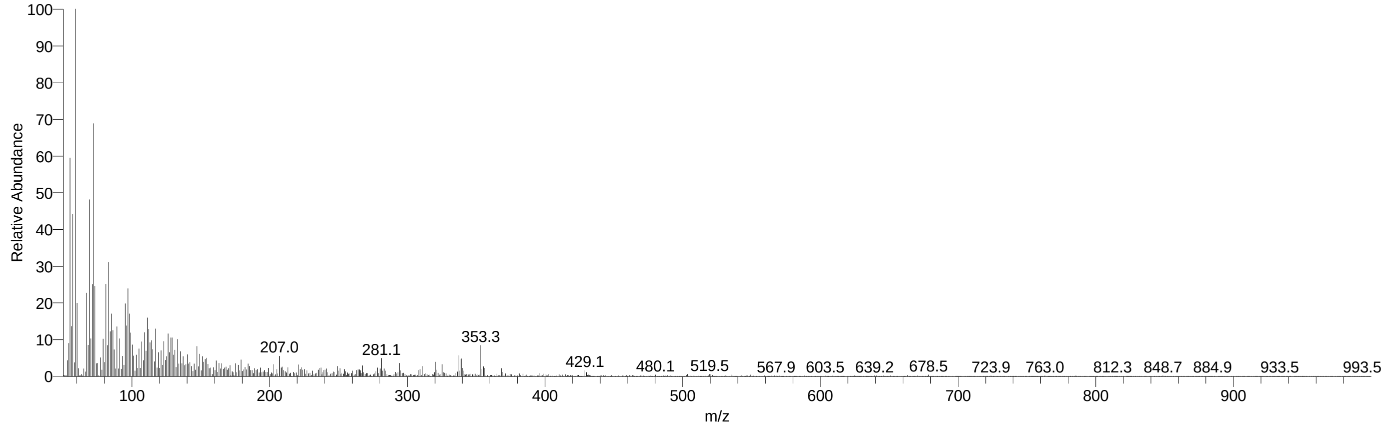

RT	Scan #	Probability	Compound Name	SI	RSI	Cas #	Area	Area %	Library
73.36	20100.000000	14.17	HAHNFETT	706	729	NA	120305346.51	1.47	WileyRegistry8e
73.36	20100.000000	14.17	HAHNFETT	706	729	NA	120305346.51	1.47	WileyRegistry8e
73.36	20100.000000	6.45	2-HYDROXY-3-[(9E)-9-OCTADECENOYLOXY]PROPYL (9E)-9-OCTADECENOATE #	686	736	2465-32-9	120305346.51	1.47	WileyRegistry8e
73.36	20100.000000	5.45	Docosanoic acid, 1,2,3-propanetriyl ester	682	700	18641-57-1	120305346.51	1.47	mainlib
73.36	20100.000000	5.45	DOCOSANOIC ACID, 1,2,3-PROPANETRIYL ESTER	682	700	18641-57-1	120305346.51	1.47	WileyRegistry8e

Hit Spectrum | Compound Structure

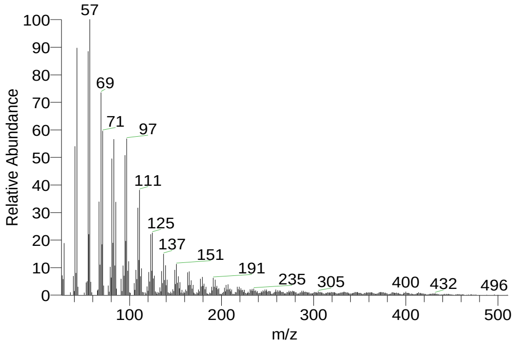

HAHNFETT
Formula , MW 0, CAS# NA, Entry# 305496

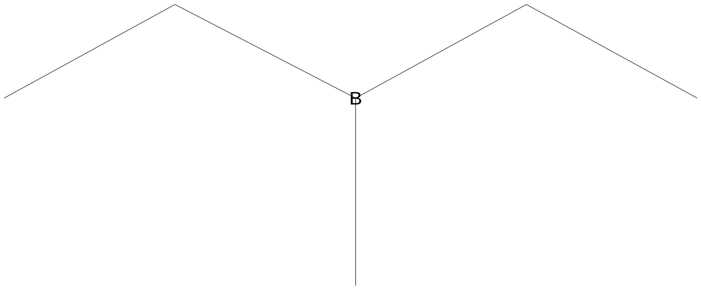

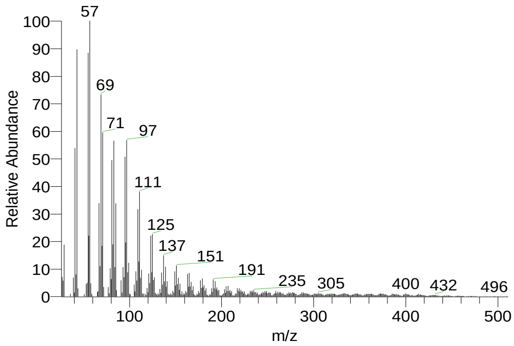

HAHNFETT
Formula , MW 0, CAS# NA, Entry# 391160

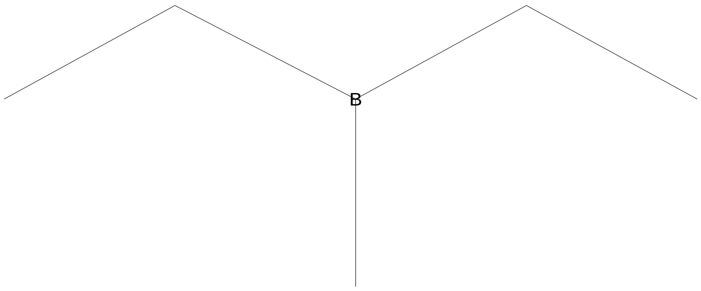

There is no signature data to report.

Library Search Report

Hit Spectrum | Compound Structure

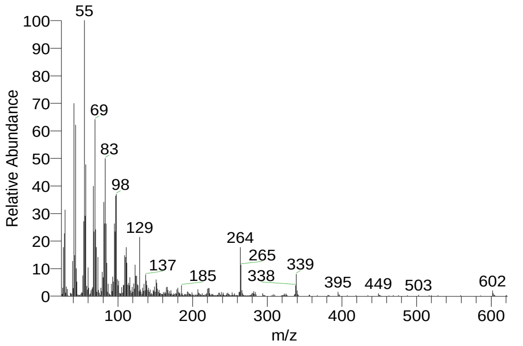

2-HYDROXY-3-[(9E)-9-OCTADECENOYLOXY]PROPYL (9E)-9-OCTADECENOATE #
Formula C39H72O5, MW 620, CAS# 2465-32-9, Entry# 298152
(Z,Z)-1,3-DIOCTADECENOYL GLYCEROL

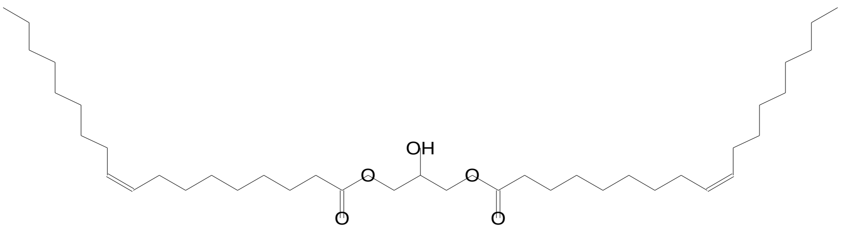

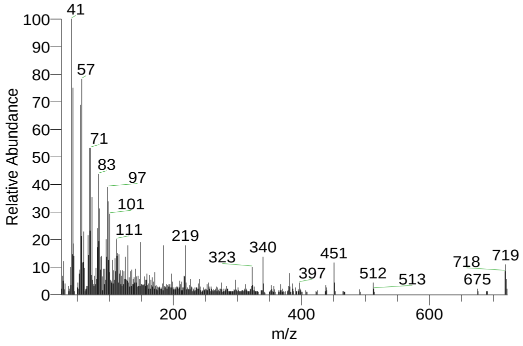

Docosanoic acid, 1,2,3-propanetriyl ester
Formula C69H134O6, MW 1058, CAS# 18641-57-1, Entry# 3322
Docosanoin, tri-

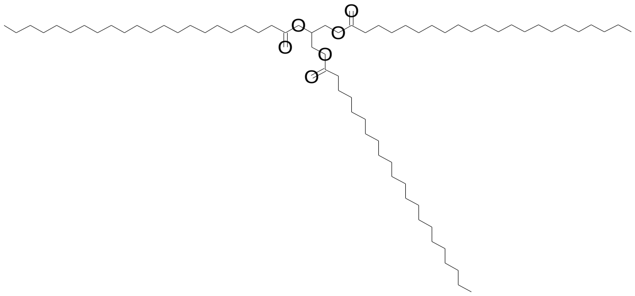

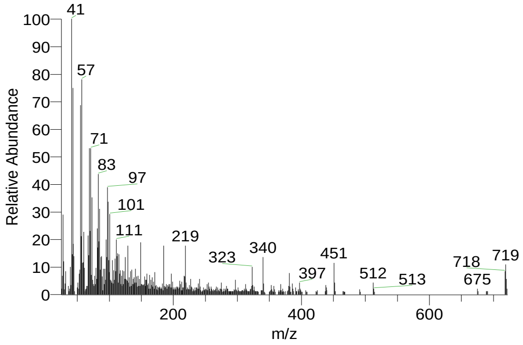

DOCOSANOIC ACID, 1,2,3-PROPANETRIYL ESTER
Formula C69H134O6, MW 1058, CAS# 18641-57-1, Entry# 305117
2,3-BIS(DOCOSANOYLOXY)PROPYL DOCOSANOATE #

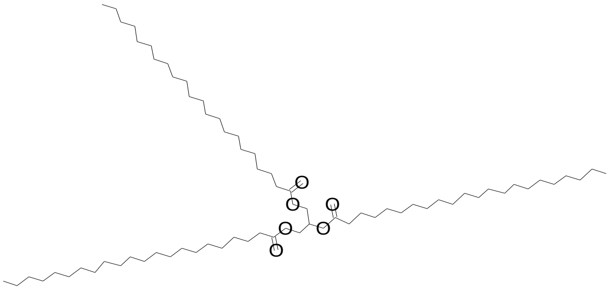

There is no signature data to report.

Library Search Report

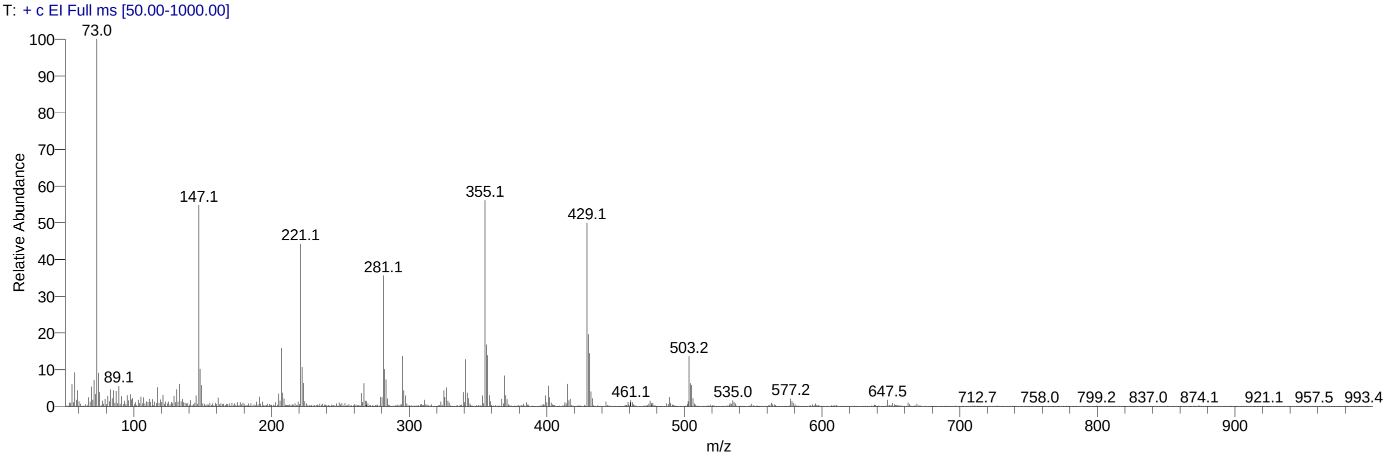

RT	Scan #	Probability	Compound Name	SI	RSI	Cas #	Area	Area %	Library
74.27	20367.000000	29.83	SILICONE OIL	787	851	NA	228475823.38	2.80	WileyRegistry8e
74.27	20367.000000	29.83	SILIKONFETT SE30 (GREVELS)	787	851	NA	228475823.38	2.80	WileyRegistry8e
74.27	20367.000000	20.46	1H-PURIN-6-AMINE, [(2-FLUOROPHENYL)METHYL]-	776	848	74421-44-6	228475823.38	2.80	WileyRegistry8e
74.27	20367.000000	14.45	Cyclodecasiloxane, eicosamethyl-	766	812	18772-36-6	228475823.38	2.80	mainlib
74.27	20367.000000	14.45	2,2,4,4,6,6,8,8,10,10,12,12,14,14,16,16,18,18,20,20-ICOSAMETHYLCYCLODECASILOXANE #	766	812	18772-36-6	228475823.38	2.80	WileyRegistry8e

Hit Spectrum	Compound Structure

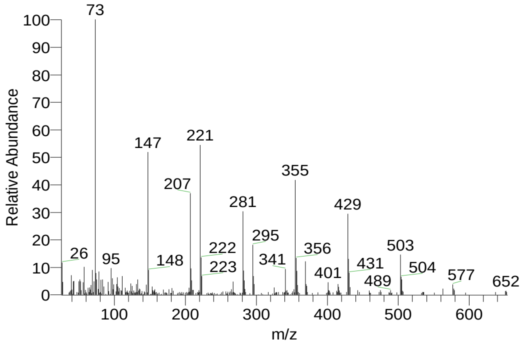

SILICONE OIL
Formula , MW 0, CAS# NA, Entry# 305490
SILIKONFETT SE30 (GREVELS)

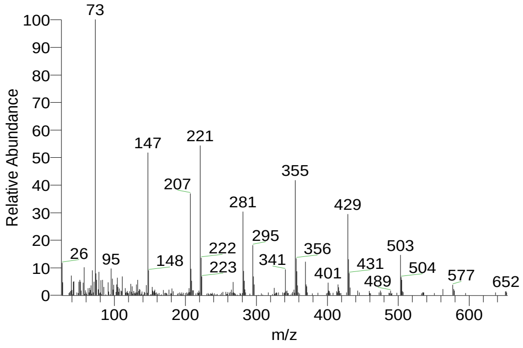

SILIKONFETT SE30 (GREVELS)
Formula , MW 0, CAS# NA, Entry# 392776

There is no signature data to report.

Library Search Report

Hit Spectrum | Compound Structure

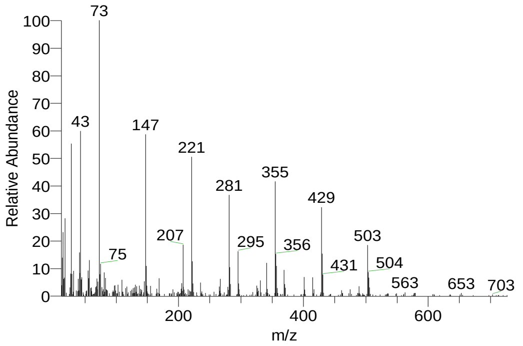

1H-PURIN-6-AMINE, [(2-FLUOROPHENYL)METHYL]-
Formula C12H10FN5, MW 243, CAS# 74421-44-6, Entry# 132518

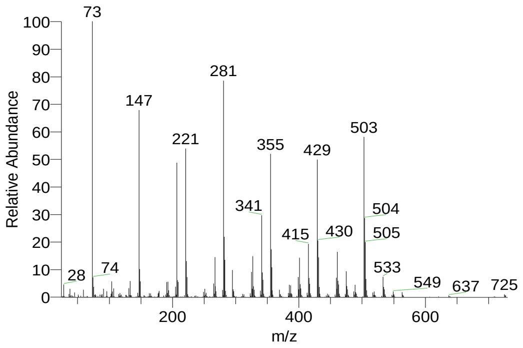

Cyclodecasiloxane, eicosamethyl-
Formula C20H60O10Si10, MW 740, CAS# 18772-36-6, Entry# 47864
2,2,4,4,6,6,8,8,10,10,12,12,14,14,16,16,18,18,20,20-Icosamethylcyclodecasiloxane #

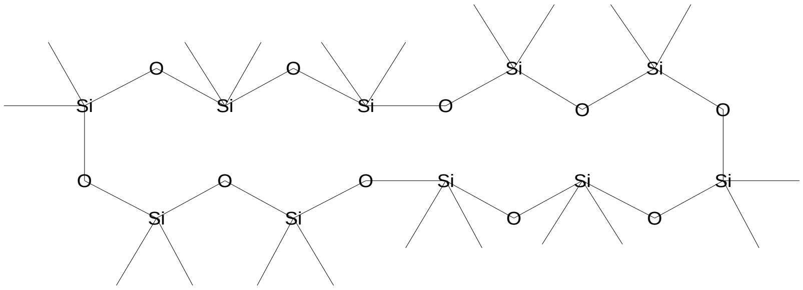

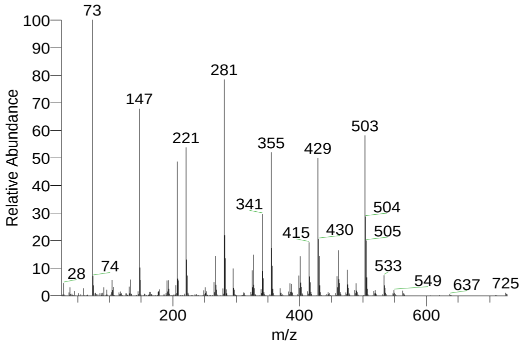

2,2,4,4,6,6,8,8,10,10,12,12,14,14,16,16,18,18,20,20-ICOSAMETHYLCYCLODECASILOXANE #
Formula C20H60O10Si10, MW 740, CAS# 18772-36-6, Entry# 380233
2,2,4,4,6,6,8,8,10,10,12,12,14,14,16,16,18,18,20,20-ICOSAMETHYLCYCLODECASILOXANE

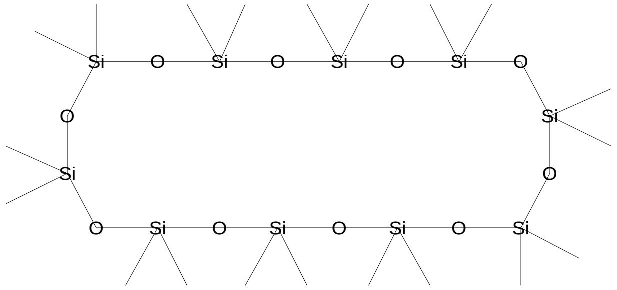

There is no signature data to report.

Library Search Report

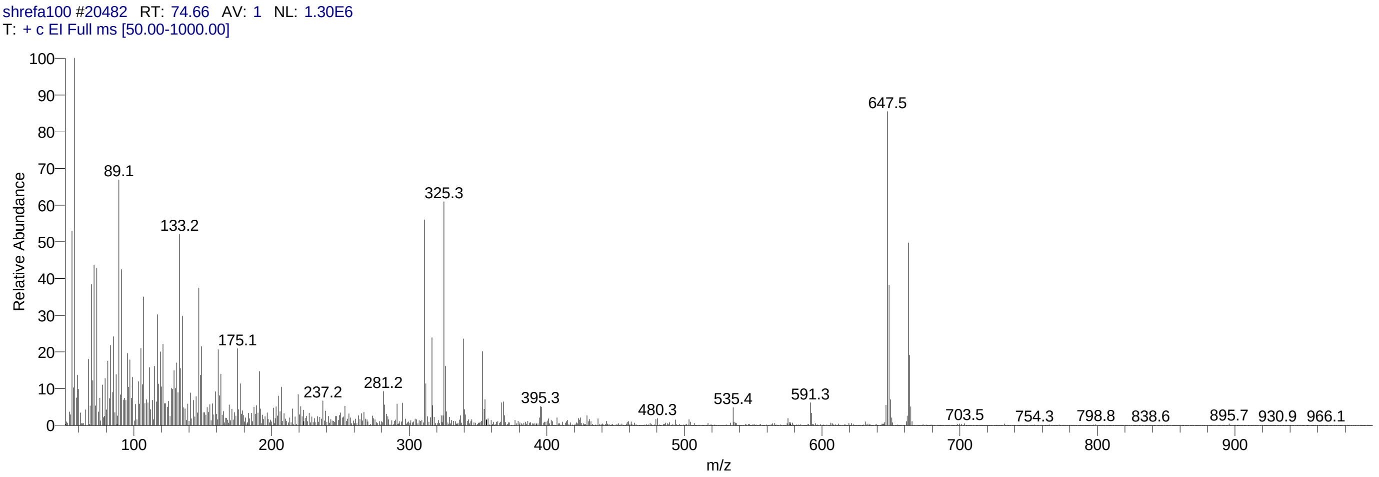

RT	Scan #	Probability	Compound Name	SI	RSI	Cas #	Area	Area %	Library
74.66	20482.000000	14.65	2,4(1H)-CYCLO-3,4-SECOAKUAMMILAN-16-CARBOXYLIC ACID, 17-HYDROXY-10-METHOXY-, METHYL ESTER, (16R)-	525	651	56259-11-1	69030451.98	0.85	WileyRegistry8e
74.66	20482.000000	13.51	3-OXO-20-METHYL-11-à-HYDROXY-N-DEMETHYLCONANINE-1,4,20-TRIENE	523	725	NA	69030451.98	0.85	WileyRegistry8e
74.66	20482.000000	7.78	3,6,9,12-Tetraoxatetradecan-1-ol, 14-(nonylphenoxy)-	507	653	26264-02-8	69030451.98	0.85	mainlib
74.66	20482.000000	5.65	Pregnane-3,20-dione, 16-methylene-11,17,21-tris[(trifluoroacetyl)oxy]-, (5à,11à)-	498	693	56438-16-5	69030451.98	0.85	mainlib
74.66	20482.000000	5.65	PREGNANE-3,20-DIONE, 16-METHYLENE-11,17,21-TRIS[(TRIFLUOROACETYL)OXY]-, (5à,11à)-	498	693	56438-16-5	69030451.98	0.85	WileyRegistry8e

Hit Spectrum	Compound Structure

Formula C22H28N2O4, MW 384, CAS# 56259-11-1, Entry# 248233

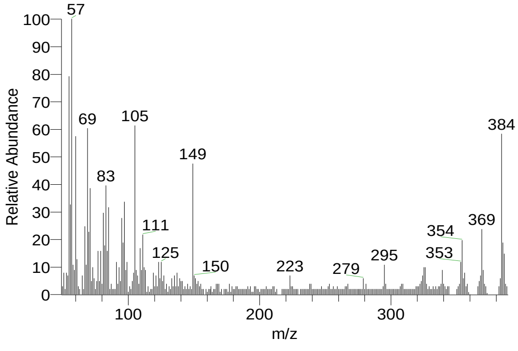

3-OXO-20-METHYL-11-à-HYDROXY-N-DEMETHYLCONANINE-1,4,20-TRIENE
Formula C21H27NO2, MW 325, CAS# NA, Entry# 210800

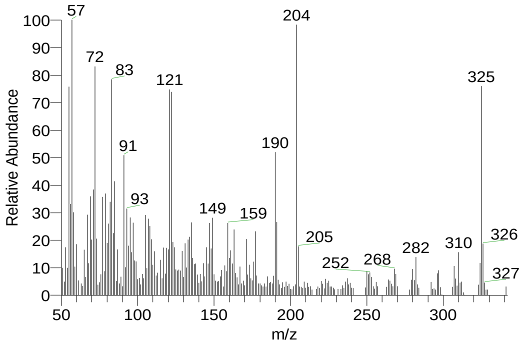

There is no signature data to report.

Library Search Report

Hit Spectrum	Compound Structure

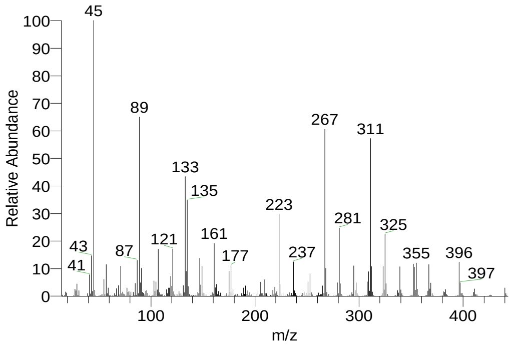

3,6,9,12-Tetraoxatetradecan-1-ol, 14-(nonylphenoxy)-
Formula C25H44O6, MW 440, CAS# 26264-02-8, Entry# 18934
$:28PITRRWWILGYENJ-UHFFFAOYSA-N

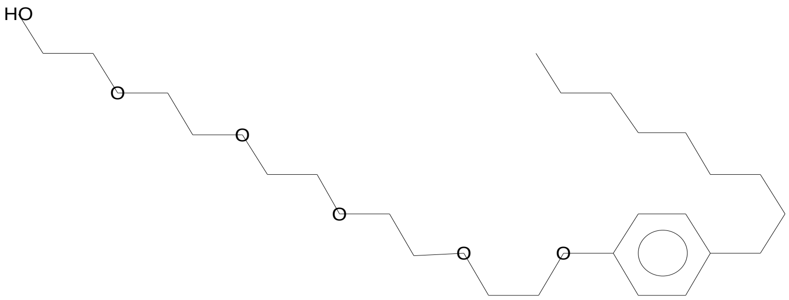

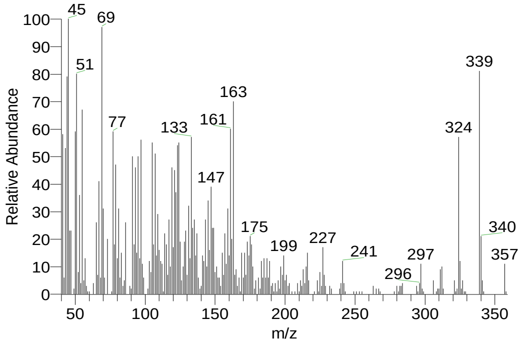

Pregnane-3,20-dione, 16-methylene-11,17,21-tris[(trifluoroacetyl)oxy]-, (5à,11à)-
Formula C28H29F9O8, MW 664, CAS# 56438-16-5, Entry# 18676
16-Methylene-3,20-dioxo-11,17-bis[(trifluoroacetyl)oxy]pregnan-21-yl trifluoroacetate #

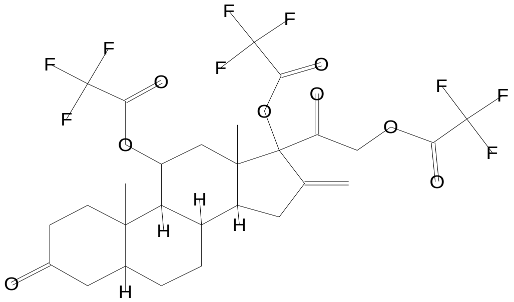

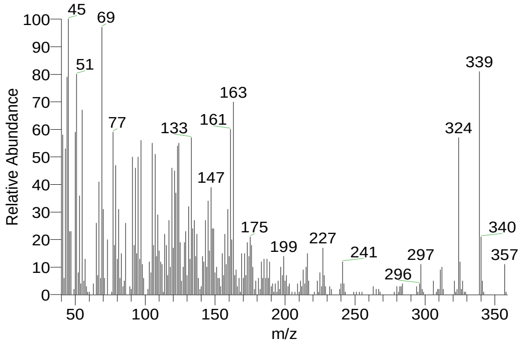

PREGNANE-3,20-DIONE, 16-METHYLENE-11,17,21-TRIS[(TRIFLUOROACETYL)OXY]-, (5à,11à)-
Formula C28H29F9O8, MW 664, CAS# 56438-16-5, Entry# 300273

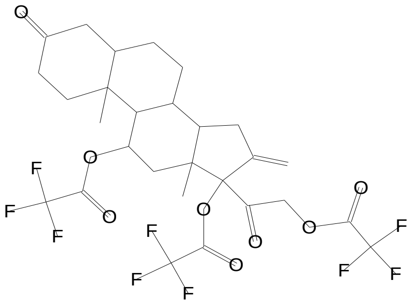

There is no signature data to report.

Library Search Report

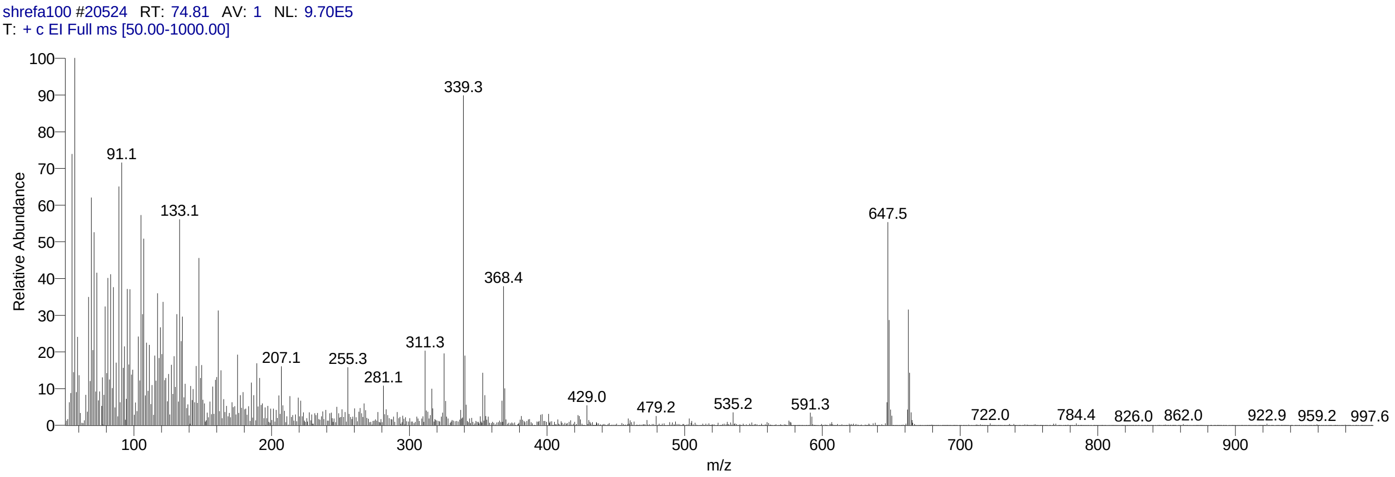

RT	Scan #	Probability	Compound Name	SI	RSI	Cas #	Area	Area %	Library
74.81	20524.000000	32.12	Prostaglandin A1-biotin	609	877	NA	53393652.53	0.65	nist_msms
74.81	20524.000000	32.12	Prostaglandin A1-biotin	609	874	NA	53393652.53	0.65	nist_msms
74.81	20524.000000	16.56	5,11,17 23-TETRAKIS(1,1-DIMETHYLETHYL)-28-METHOXYPENTACYCLO[19.3.1.1(3,7).1(9,13).1(15,19)]OCTACOSA-1(25),3,5,7(28),9,11,13(27),15,17,19(26),21,23-DODECENE-25,26,27-TRIOL	591	605	NA	53393652.53	0.65	WileyRegistry8e
74.81	20524.000000	6.54	Prostaglandin F2à-biotinamide	569	889	NA	53393652.53	0.65	nist_msms
74.81	20524.000000	1.54	TERT-BUTYL(DIMETHYL)SILYL (11E)-12-METHYL-11-ICOSENYL ETHER	530	635	NA	53393652.53	0.65	WileyRegistry8e

Hit Spectrum

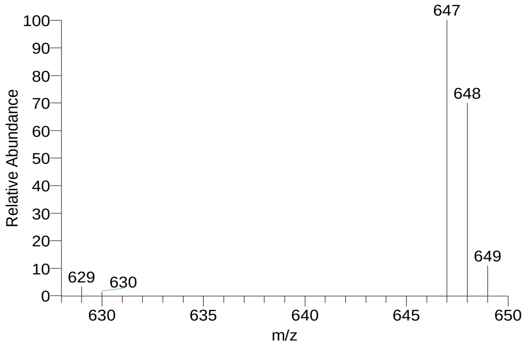

Compound Structure

Prostaglandin A1-biotin
Formula C35H58N4O5S, MW 646, CAS# NA, Entry# 158118
$:17Consensus spectrum; Acetonitrile/Water/Formic acid; Vial_ID=4701; mz_diff=0.0014; Nreps=2/2

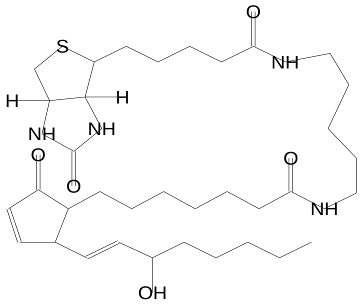

There is no signature data to report.

Library Search Report

Hit Spectrum | Compound Structure

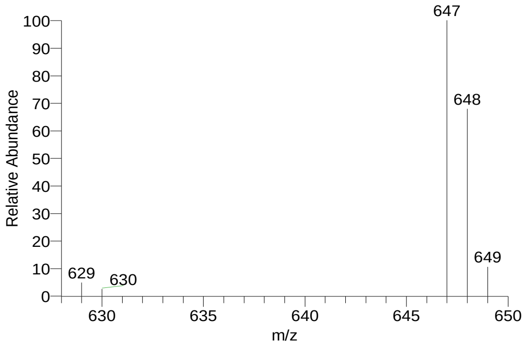

Prostaglandin A1-biotin
Formula C35H58N4O5S, MW 646, CAS# NA, Entry# 158119
$:17Consensus spectrum; Acetonitrile/Water/Formic acid; Vial_ID=4701; mz_diff=0.0014; Nreps=2/2

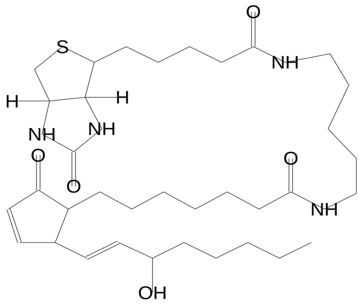

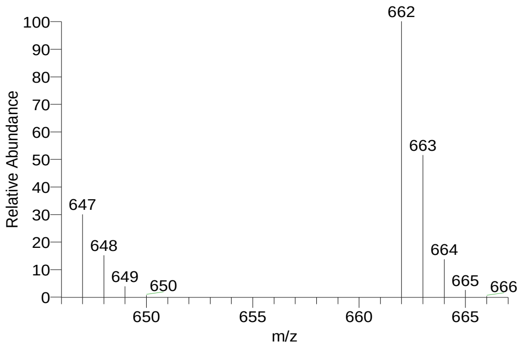

Formula C45H58O4, MW 662, CAS# NA, Entry# 300252

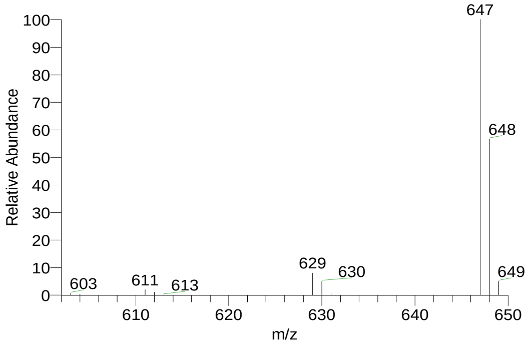

Prostaglandin F2à-biotinamide
Formula C35H60N4O6S, MW 664, CAS# NA, Entry# 95521
9à,11à,15S-Trihydroxyprosta-5Z,13E-dien-1-oyl-N'-biotinoyl-1,5-diaminopentane

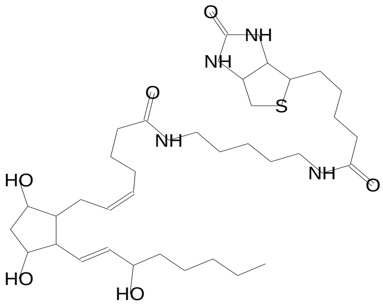

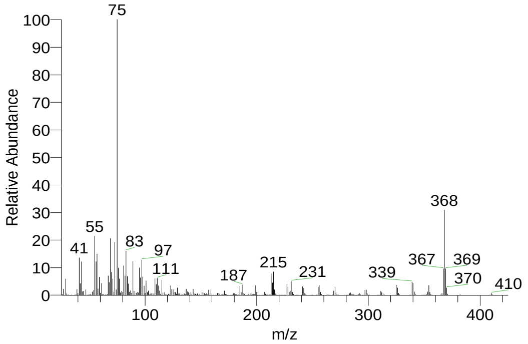

TERT-BUTYL(DIMETHYL)SILYL (11E)-12-METHYL-11-ICOSENYL ETHER
Formula C27H55DOSi, MW 425, CAS# NA, Entry# 366593
TERT-BUTYL(DIMETHYL)SILYL 12-METHYL-11-ICOSENYL ETHER

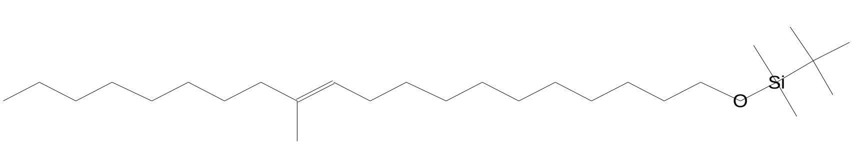

There is no signature data to report.

Library Search Report

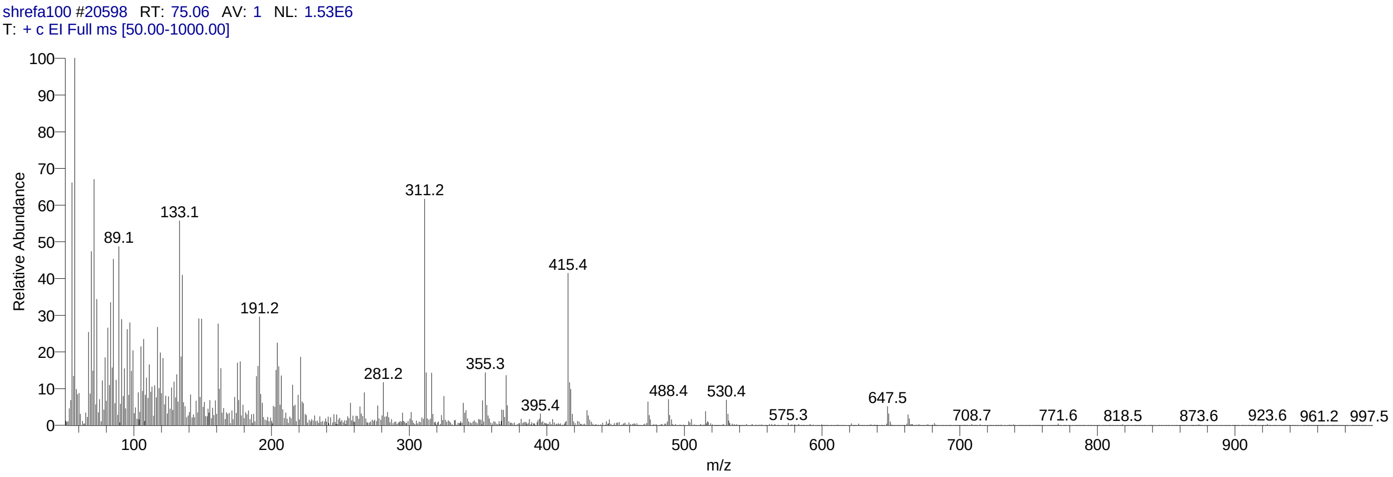

RT	Scan #	Probability	Compound Name	SI	RSI	Cas #	Area	Area %	Library
75.06	20598.000000	19.35	Propanoic acid, 2-(3-acetoxy-4,4,14-trimethylandrost-8-en-17-yl)-	650	693	NA	74716121.36	0.91	mainlib
75.06	20598.000000	19.35	2-(3-ACETOXY-4,4,10,13,14-PENTAMETHYL-2,3,4,5,6,7,10,11,12,13,14,15,16,17-TETRADECAHYDRO-1H-CYCLOPENTA[A]PHENANTHREN-17-YL)-PROPIONI	650	693	NA	74716121.36	0.91	WileyRegistry8e
75.06	20598.000000	12.89	3-Cholestanol, 2-fromyl-3-benzyl-	638	677	NA	74716121.36	0.91	mainlib
75.06	20598.000000	12.89	3-BENZYL-17-(1,5-DIMETHYL-HEXYL)-3-HYDROXY-10,13-DIMETHYL-HEXADECAHYDRO-CYCLOPENTA[A]PHENANTHRENE-2-CARBALDEHYDE	638	677	NA	74716121.36	0.91	WileyRegistry8e
75.06	20598.000000	5.87	3-Desoxo-3,16-dihydroxy-12-desoxyphorbol 3,13,16,20-tetraacetate	618	658	NA	74716121.36	0.91	mainlib

Hit Spectrum

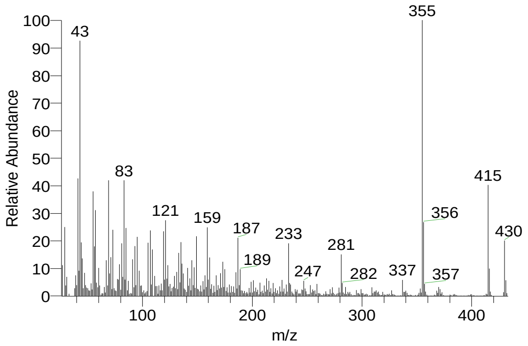

Compound Structure

Propanoic acid, 2-(3-acetoxy-4,4,14-trimethylandrost-8-en-17-yl)-
Formula C27H42O4, MW 430, CAS# NA, Entry# 258204

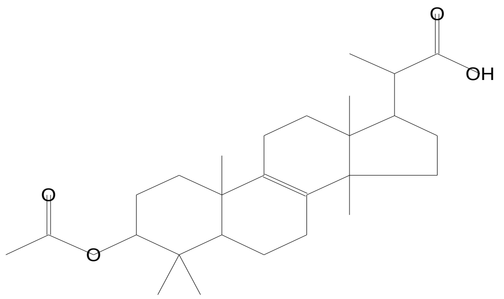

There is no signature data to report.

Library Search Report

Hit Spectrum | Compound Structure

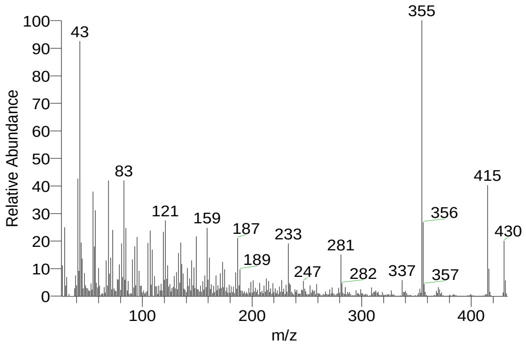

Formula C27H42O4, MW 430, CAS# NA, Entry# 367790

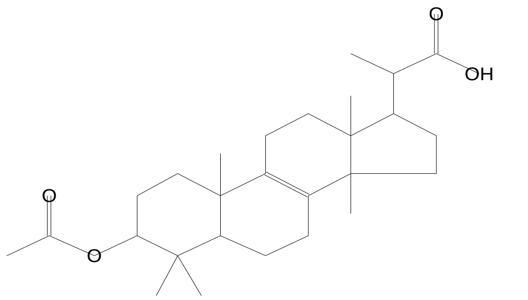

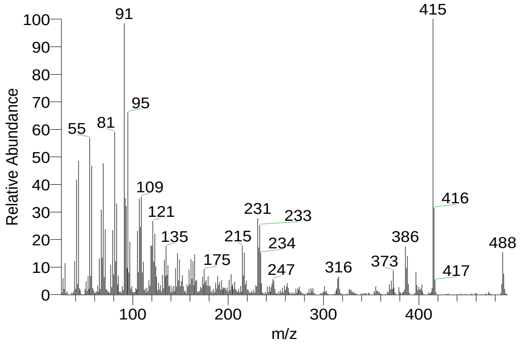

3-Cholestanol, 2-fromyl-3-benzyl-
Formula C35H54O2, MW 506, CAS# NA, Entry# 263694
3-Benzyl-3-hydroxycholestane-2-carbaldehyde #

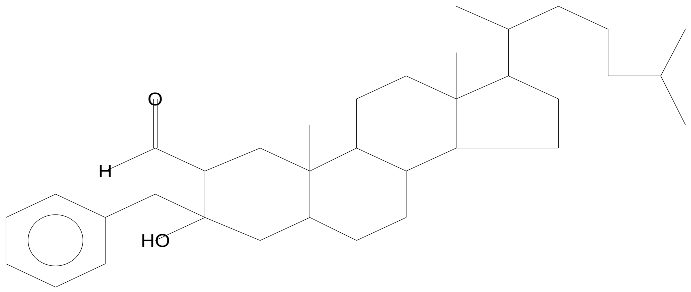

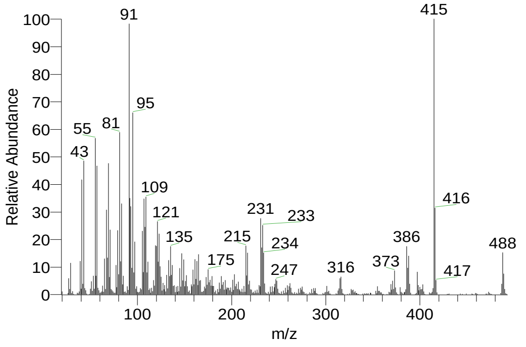

Formula C35H54O2, MW 506, CAS# NA, Entry# 361570
3-BENZYL-3-HYDROXYCHOLESTANE-2-CARBALDEHYDE

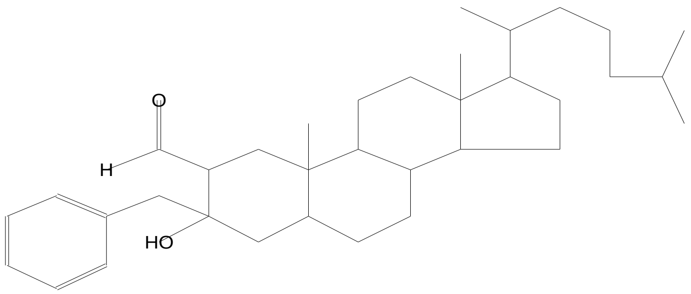

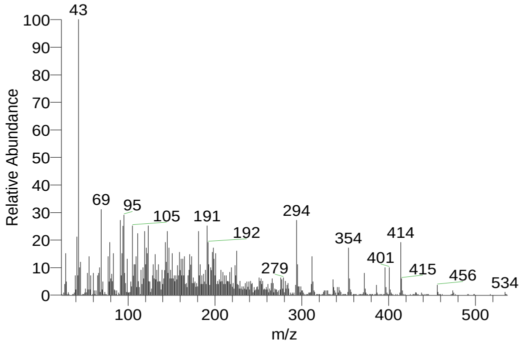

3-Desoxo-3,16-dihydroxy-12-desoxyphorbol 3,13,16,20-tetraacetate
Formula C28H38O10, MW 534, CAS# NA, Entry# 9152

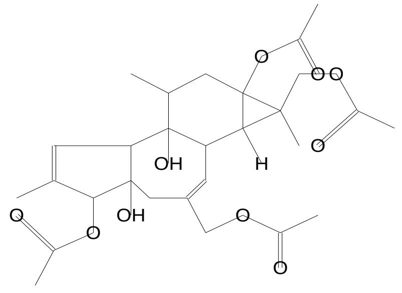

There is no signature data to report.

Library Search Report

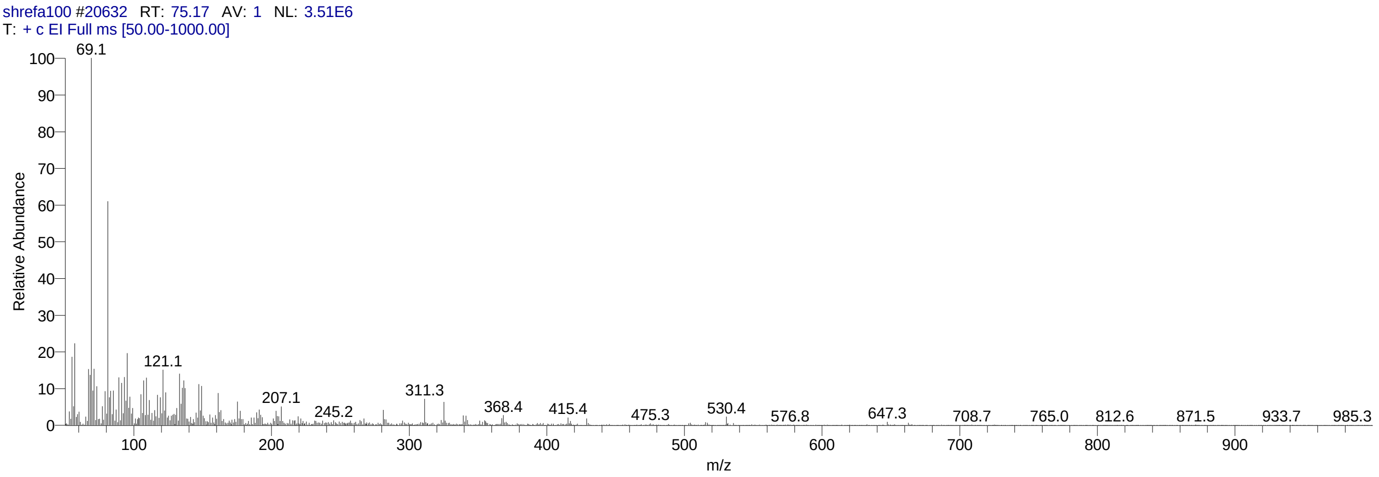

RT	Scan #	Probability	Compound Name	SI	RSI	Cas #	Area	Area %	Library
75.17	20632.000000	58.77	03027205002 FLAVONE 4'-OH,5-OH,7-DI-O-GLUCOSIDE	729	747	NA	50372356.40	0.62	WileyRegistry8e
75.17	20632.000000	16.97	1-Heptatriacotanol	701	825	105794-58-9	50372356.40	0.62	mainlib
75.17	20632.000000	8.75	Rhodopin	683	694	105-92-0	50372356.40	0.62	mainlib
75.17	20632.000000	8.75	.PSI.,.PSI.-CAROTENE, 1,2-DIHYDRO-1-HYDROXY-	681	692	105-92-0	50372356.40	0.62	WileyRegistry8e
75.17	20632.000000	2.29	Cholest-4-ene, 3á-(methoxymethoxy)-	650	681	4707-85-1	50372356.40	0.62	mainlib

Hit Spectrum | Compound Structure

03027205002 FLAVONE 4'-OH,5-OH,7-DI-O-GLUCOSIDE
Formula C27H30O15, MW 594, CAS# NA, Entry# 296184

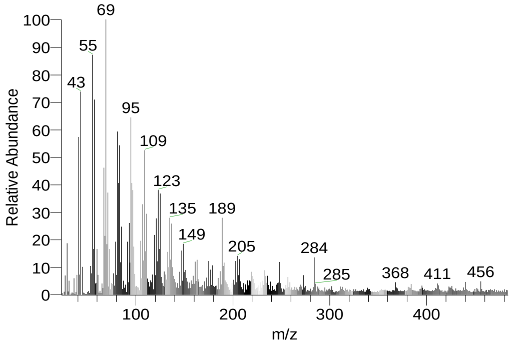

1-Heptatriacotanol
Formula C37H76O, MW 536, CAS# 105794-58-9, Entry# 7819
1-Heptatriacontanol #

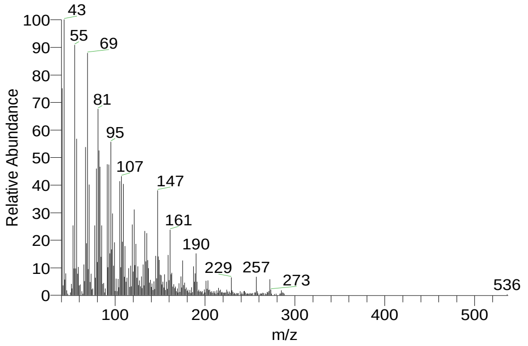

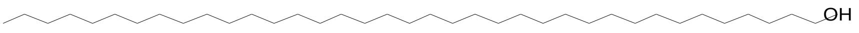

There is no signature data to report.

Library Search Report

Hit Spectrum

Compound Structure

SI 683, RSI 694, mainlib, Entry# 37045, CAS# 105-92-0, Rhodopin

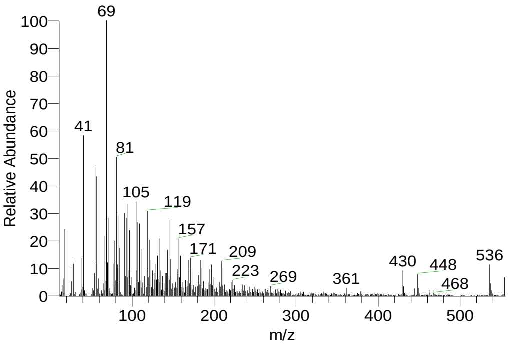

Rhodopin
Formula C40H58O, MW 554, CAS# 105-92-0, Entry# 37045
.psi.,.psi.-Carotene, 1,2-dihydro-1-hydroxy-

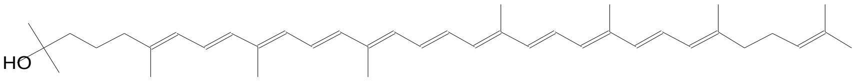

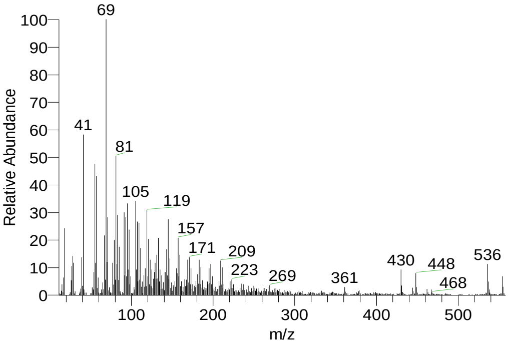

.PSI.,.PSI.-CAROTENE, 1,2-DIHYDRO-1-HYDROXY-
Formula C40H58O, MW 554, CAS# 105-92-0, Entry# 292854
1,2-DIHYDRO-PSI,PSI-CAROTENE #

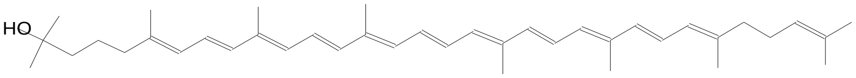

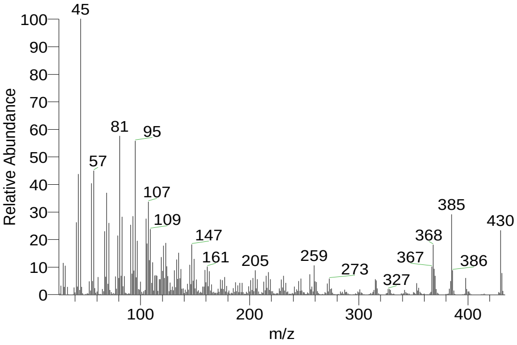

Cholest-4-ene, 3á-(methoxymethoxy)-
Formula C29H50O2, MW 430, CAS# 4707-85-1, Entry# 18816
3-(Methoxymethoxy)cholest-4-ene #

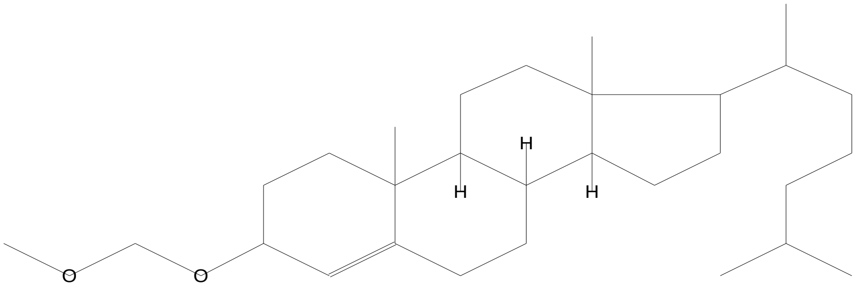

There is no signature data to report.

Library Search Report

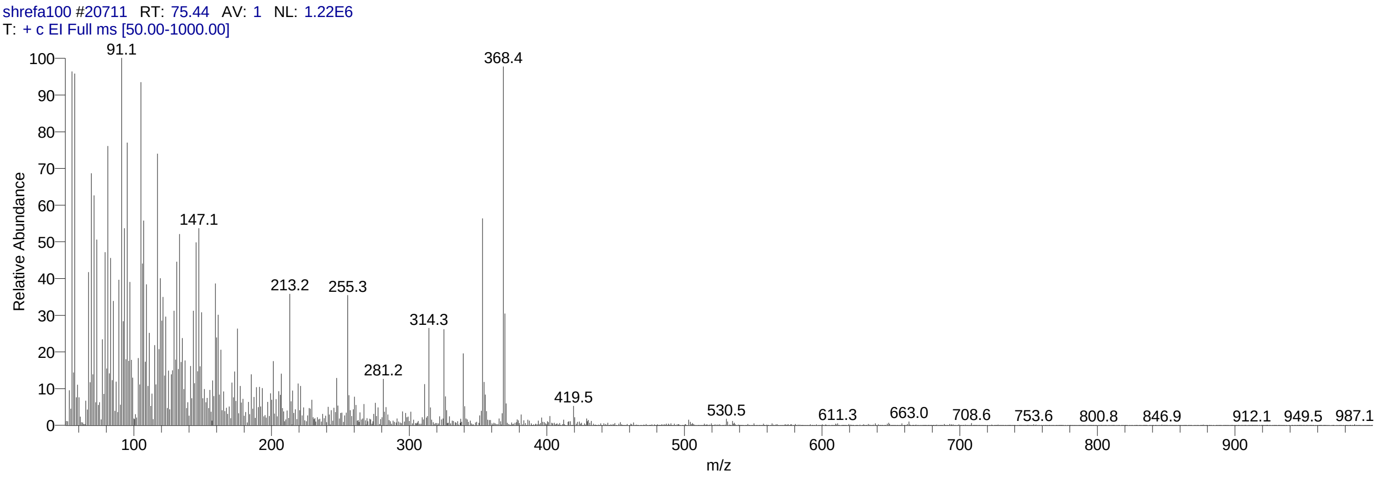

RT	Scan #	Probability	Compound Name	SI	RSI	Cas #	Area	Area %	Library
75.44	20711.000000	9.85	CHOLEST-5-EN-3-YL STEARATE	780	865	NA	112843218.33	1.38	WileyRegistry8e
75.44	20711.000000	7.54	CHOLEST-5-EN-3-YL PALMITATE	773	834	NA	112843218.33	1.38	WileyRegistry8e
75.44	20711.000000	7.25	Cholesterol margarate	772	802	24365-37-5	112843218.33	1.38	mainlib
75.44	20711.000000	6.97	CHOLEST-5-EN-3-OL (3á)-, ACETATE	771	797	604-35-3	112843218.33	1.38	WileyRegistry8e
75.44	20711.000000	6.70	CHOLEST-5-EN-3-YL BENZOATE	770	827	604-32-0	112843218.33	1.38	WileyRegistry8e

Hit Spectrum | Compound Structure

CHOLEST-5-EN-3-YL STEARATE
Formula C45H80O2, MW 652, CAS# NA, Entry# 359228
CHOLESTERINSTEARAT

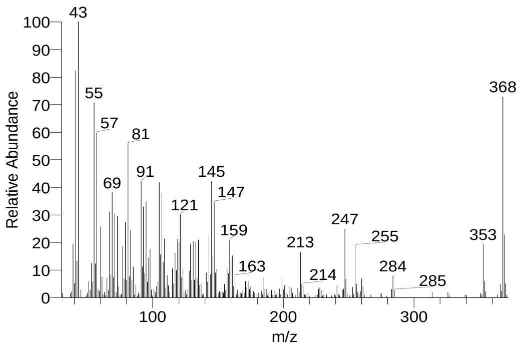

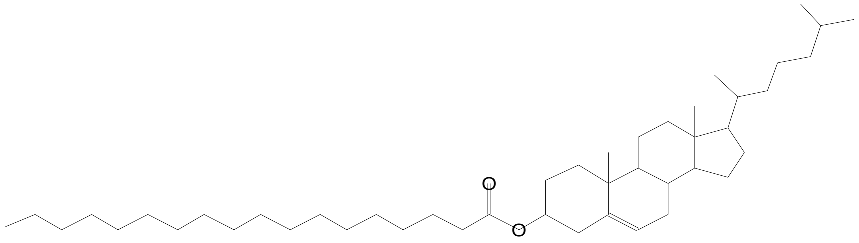

CHOLEST-5-EN-3-YL PALMITATE
Formula C43H76O2, MW 624, CAS# NA, Entry# 359227
CHOLESTERINPALMITAT

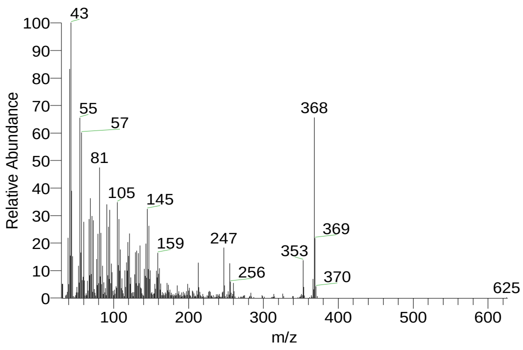

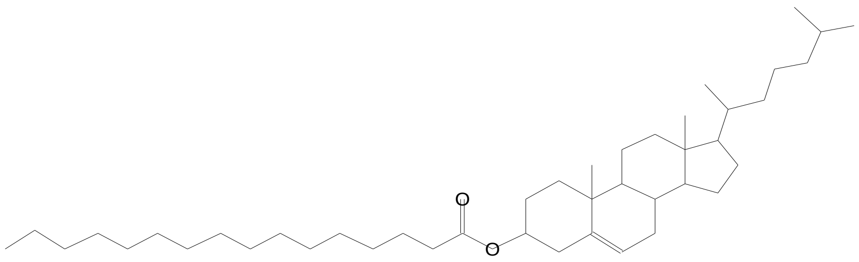

There is no signature data to report.

Library Search Report

Hit Spectrum | Compound Structure

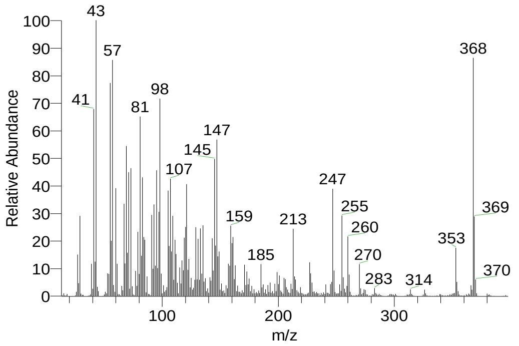

Cholesterol margarate
Formula C44H78O2, MW 638, CAS# 24365-37-5, Entry# 16039

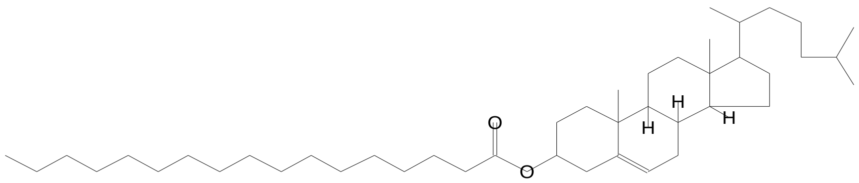

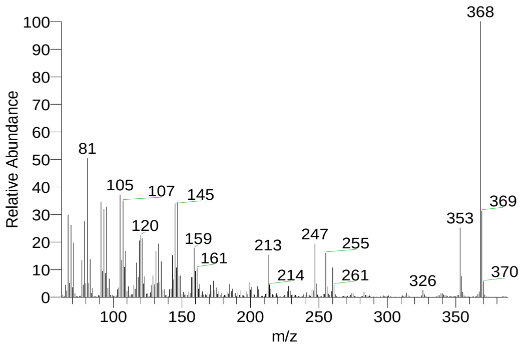

CHOLEST-5-EN-3-OL (3á)-, ACETATE
Formula C29H48O2, MW 428, CAS# 604-35-3, Entry# 267497
CHOLEST-5-EN-3-YL ACETATE #

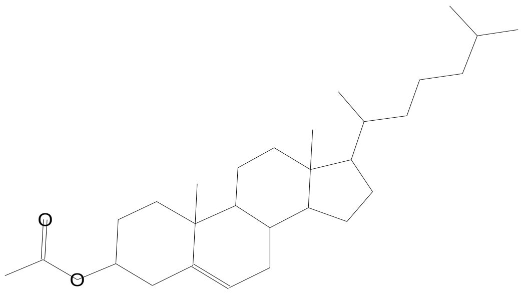

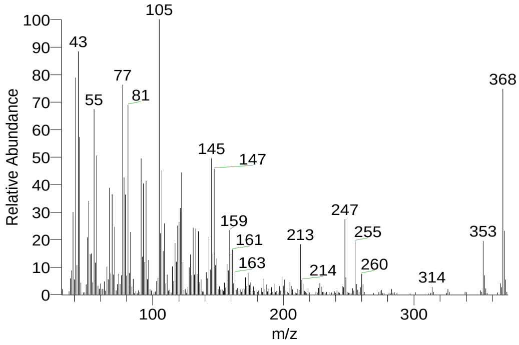

CHOLEST-5-EN-3-YL BENZOATE
Formula C34H50O2, MW 490, CAS# 604-32-0, Entry# 359229
CHOLESTERINBENZOAT

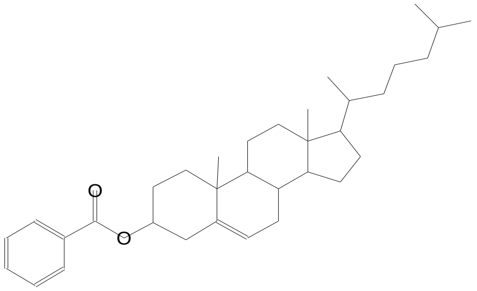

There is no signature data to report.

Library Search Report

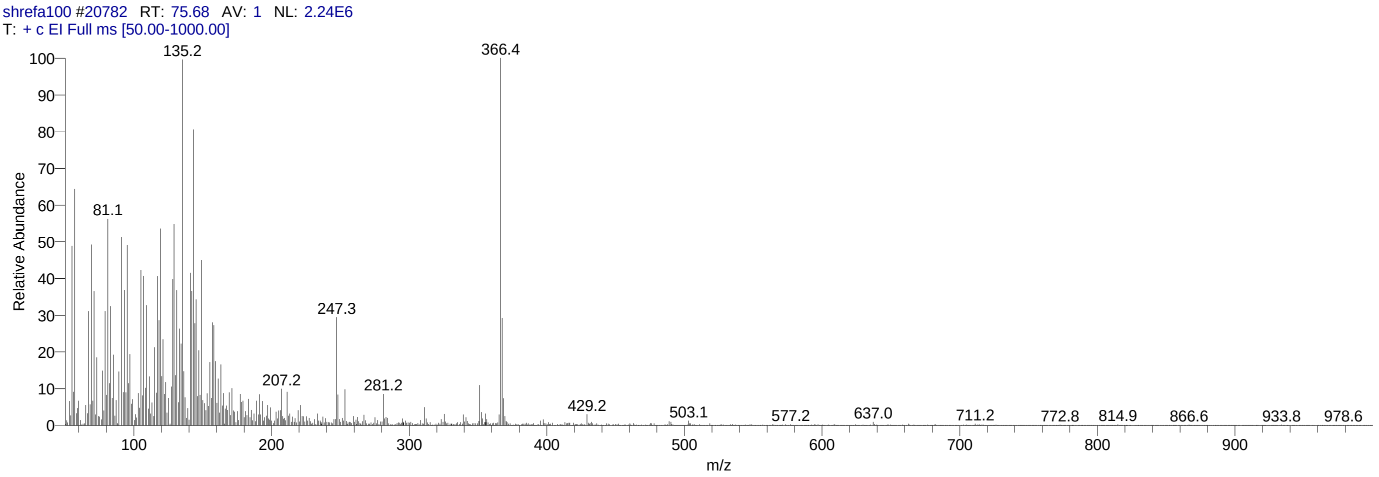

RT	Scan #	Probability	Compound Name	SI	RSI	Cas #	Area	Area %	Library
75.68	20782.000000	43.21	CHOLESTA-4,6-DIEN-3-OL, (3á)-	798	888	14214-69-8	193408339.58	2.37	WileyRegistry8e
75.68	20782.000000	43.21	Cholesta-4,6-dien-3-ol, (3á)-	793	884	14214-69-8	193408339.58	2.37	mainlib
75.68	20782.000000	31.37	CHOLESTA-4,6-DIEN-3-OL, BENZOATE, (3á)-	789	887	25485-34-1	193408339.58	2.37	WileyRegistry8e
75.68	20782.000000	4.85	DIMETHYL 3-(3',4',5'-TRIMETHYLPHENYL)THIOPHENE-2,5-DICARBOXYLATE	722	889	NA	193408339.58	2.37	WileyRegistry8e
75.68	20782.000000	3.81	(Z)-5-TERT-BUTYL-8-(2-PHENYLETHENYL)[2.2]METACYCLOPHANE	716	888	NA	193408339.58	2.37	WileyRegistry8e

Hit Spectrum | Compound Structure

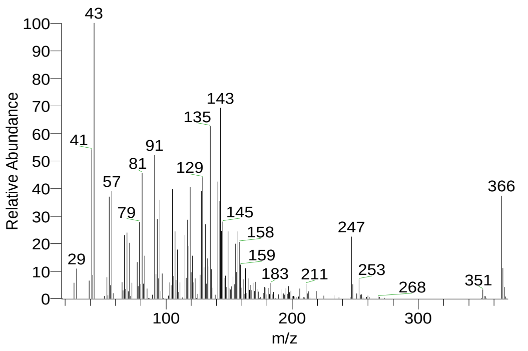

CHOLESTA-4,6-DIEN-3-OL, (3á)-
Formula C27H44O, MW 384, CAS# 14214-69-8, Entry# 248519
CHOLESTA-4,6-DIEN-3-OL #

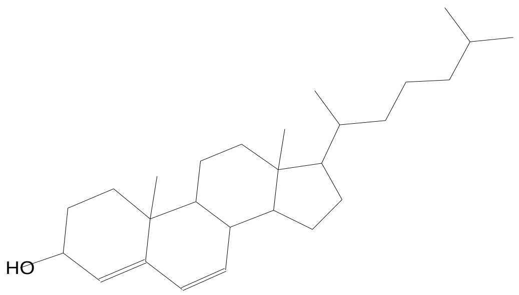

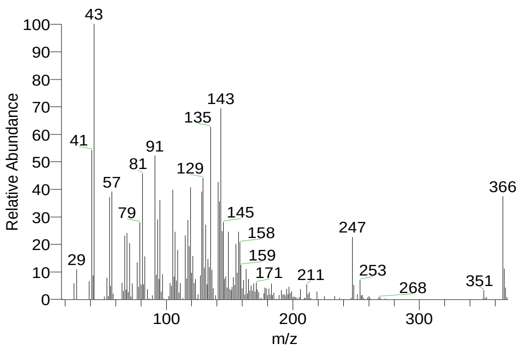

Cholesta-4,6-dien-3-ol, (3á)-
Formula C27H44O, MW 384, CAS# 14214-69-8, Entry# 13311
4,6-Cholestadien-3á-ol

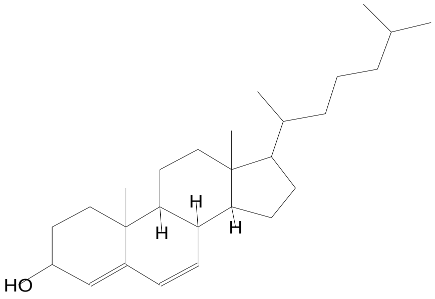

There is no signature data to report.

Library Search Report

Hit Spectrum | Compound Structure

CHOLESTA-4,6-DIEN-3-OL, BENZOATE, (3á)-
Formula C34H48O2, MW 488, CAS# 25485-34-1, Entry# 283595
4,6-CHOLESTADIEN-3á-OL, BENZOATE

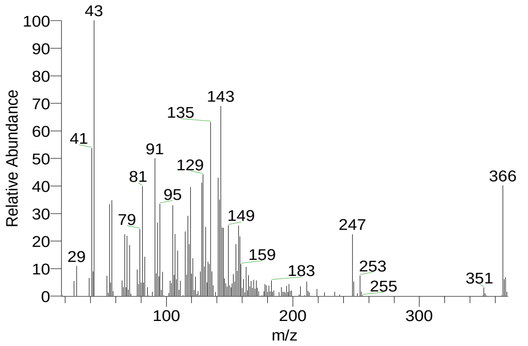

DIMETHYL 3-(3',4',5'-TRIMETHYLPHENYL)THIOPHENE-2,5-DICARBOXYLATE
Formula C17H18O7S, MW 366, CAS# NA, Entry# 238344

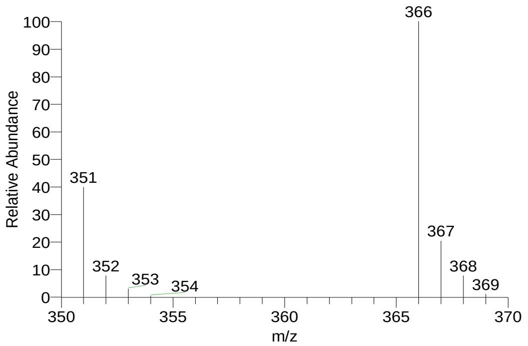

(Z)-5-TERT-BUTYL-8-(2-PHENYLETHENYL)[2.2]METACYCLOPHANE
Formula C28H30, MW 366, CAS# NA, Entry# 238960

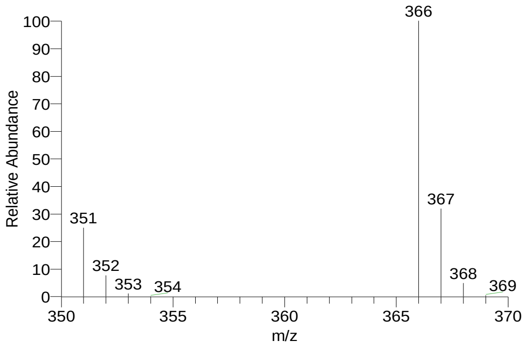

There is no signature data to report.

Library Search Report

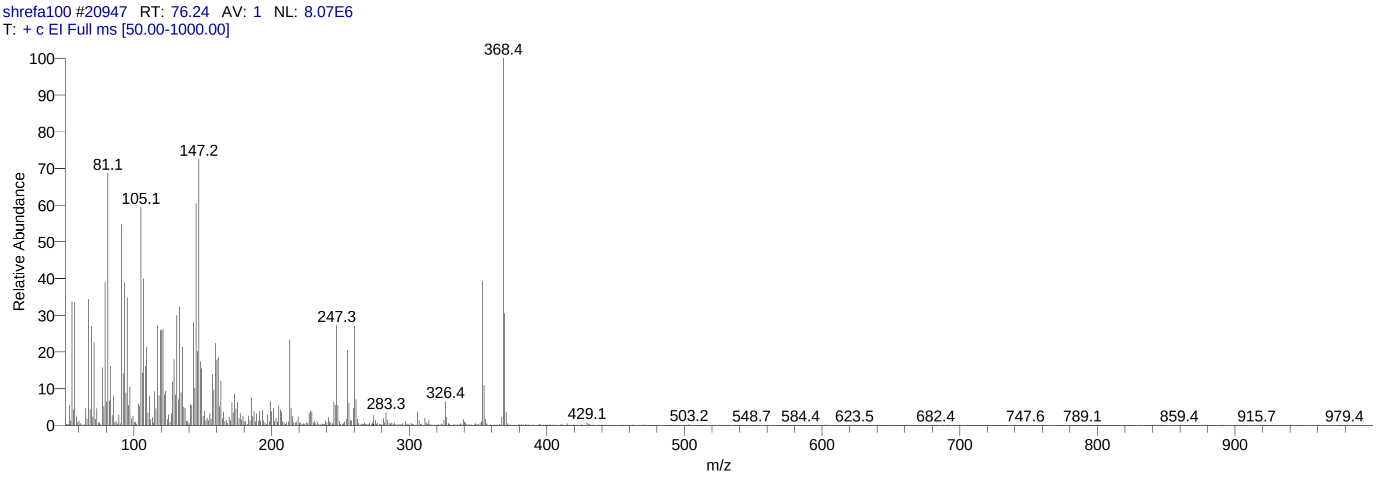

RT	Scan #	Probability	Compound Name	SI	RSI	Cas #	Area	Area %	Library
76.24	20947.000000	15.95	Cholest-3,5-diene	914	947	NA	747186027.90	9.15	mainlib
76.24	20947.000000	11.26	Cholesta-3,5-diene	904	923	747-90-0	747186027.90	9.15	mainlib
76.24	20947.000000	11.26	CHOLESTA-3,5-DIENE	904	923	747-90-0	747186027.90	9.15	WileyRegistry8e
76.24	20947.000000	11.26	CHOLESTA-3,5-DIENE	898	944	747-90-0	747186027.90	9.15	WileyRegistry8e
76.24	20947.000000	8.63	CHOLEST-5-EN-3-YL ACETATE	897	911	NA	747186027.90	9.15	WileyRegistry8e

Hit Spectrum

Compound Structure

SI 914, RSI 947, mainlib, Entry# 259791, CAS# NA, Cholest-3,5-diene

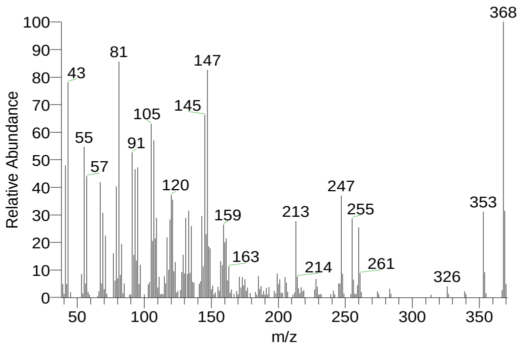

Cholest-3,5-diene
Formula C27H44, MW 368, CAS# NA, Entry# 259791
$:28RLHIRZFWJBOHHD-UHFFFAOYSA-N

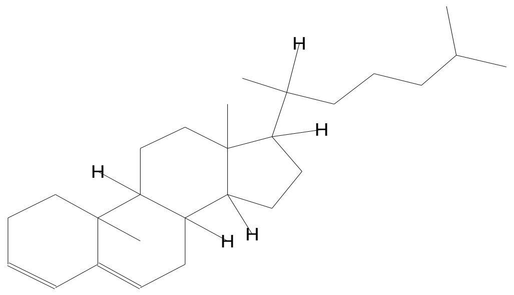

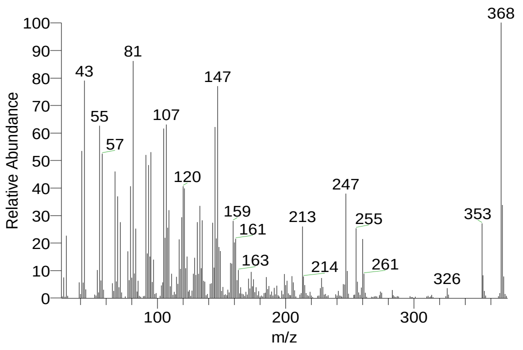

Cholesta-3,5-diene
Formula C27H44, MW 368, CAS# 747-90-0, Entry# 259789
ë(3,5)-Cholestadiene

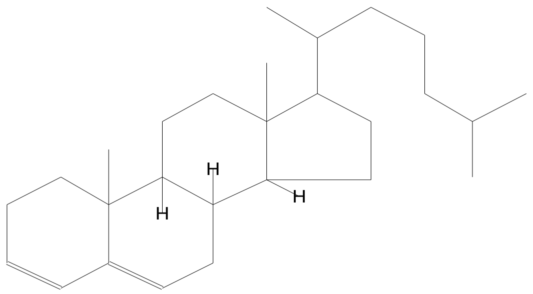

There is no signature data to report.

Library Search Report

Hit Spectrum	Compound Structure

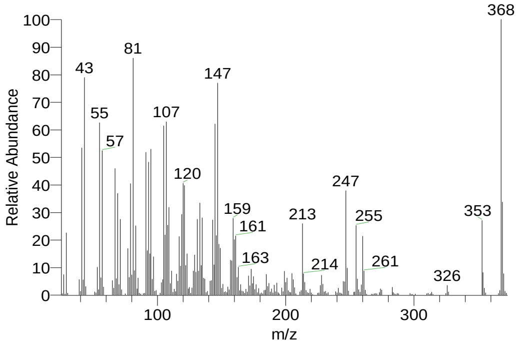

CHOLESTA-3,5-DIENE
Formula C27H44, MW 368, CAS# 747-90-0, Entry# 374566
.DELTA.(SUP3,5)-CHOLESTADIENE

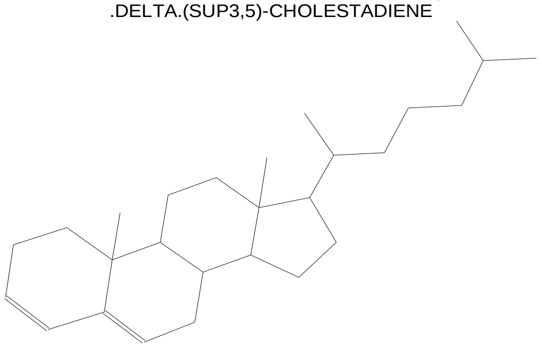

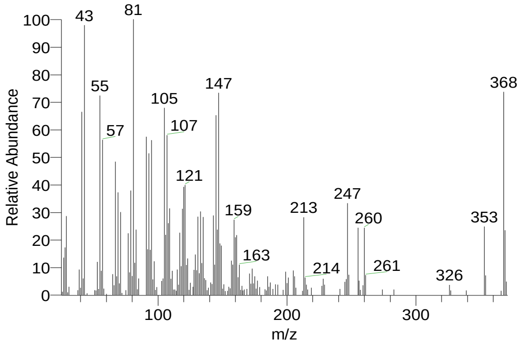

CHOLESTA-3,5-DIENE
Formula C27H44, MW 368, CAS# 747-90-0, Entry# 384698
.DELTA.(SUP3,5)-CHOLESTADIENE

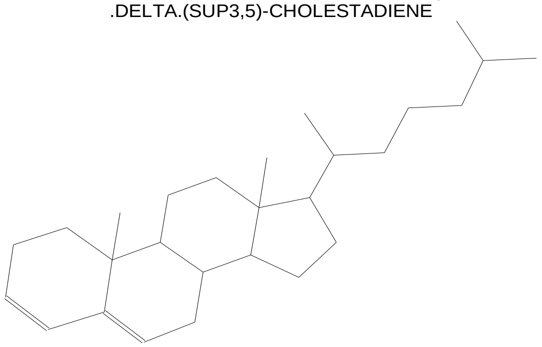

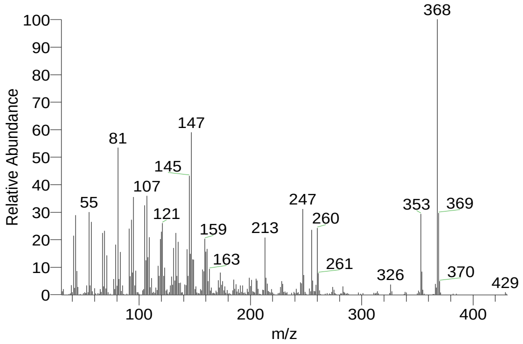

CHOLEST-5-EN-3-YL ACETATE
Formula C29H48O2, MW 428, CAS# NA, Entry# 359269
CHOLESTERYLACETAT

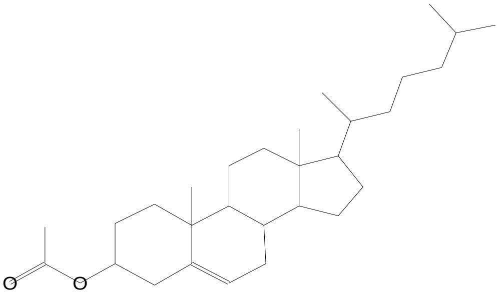

There is no signature data to report.

Library Search Report

shrefa100 #21266 RT: 77.33 AV: 1 NL: 2.25E6
T: + c EI Full ms [50.00-1000.00]

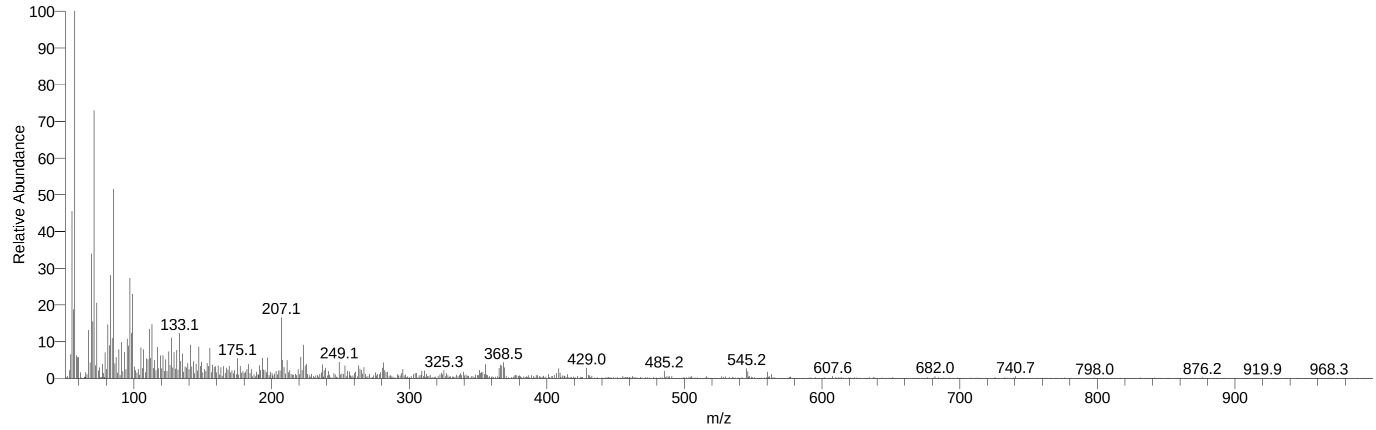

RT	Scan #	Probability	Compound Name	SI	RSI	Cas #	Area	Area %	Library
77.33	21266.000000	28.70	4H-1-BENZOPYRAN-4-ONE, 2-(3,4-DIHYDROXYPHENYL)-6,8-DI-á-D-GLUCOPYRANOSYL-5,7-DIHYDROXY-	737	775	29428-58-8	43533412.39	0.53	WileyRegistry8e
77.33	21266.000000	18.54	HAHNFETT	724	765	NA	43533412.39	0.53	WileyRegistry8e
77.33	21266.000000	18.54	HAHNFETT	724	765	NA	43533412.39	0.53	WileyRegistry8e
77.33	21266.000000	5.25	ISOCHIAPIN B	695	794	NA	43533412.39	0.53	WileyRegistry8e
77.33	21266.000000	5.25	ISOCHIAPIN B %2<	695	794	NA	43533412.39	0.53	WileyRegistry8e

Hit Spectrum	Compound Structure

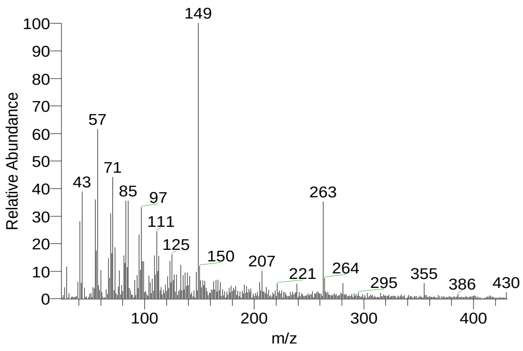

Formula C27H30O16, MW 610, CAS# 29428-58-8, Entry# 297453
6,8-DI-C-á-GLUCOSYLLUTEOLIN

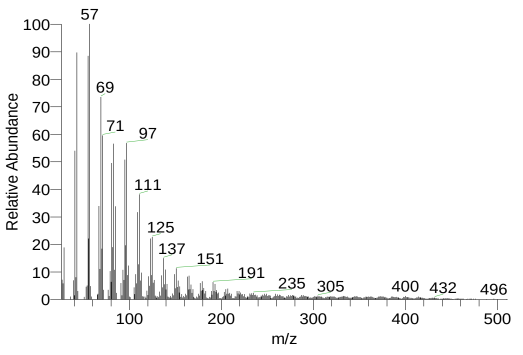

HAHNFETT
Formula , MW 0, CAS# NA, Entry# 305496

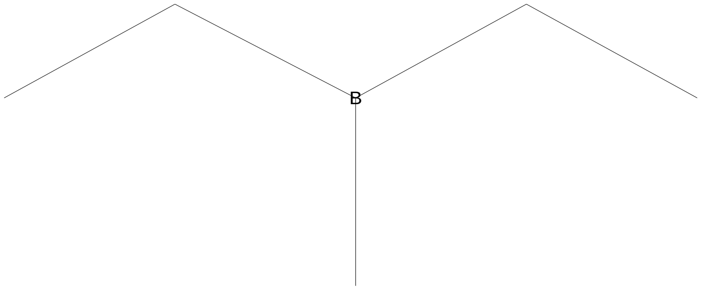

There is no signature data to report.

Library Search Report

Hit Spectrum | Compound Structure

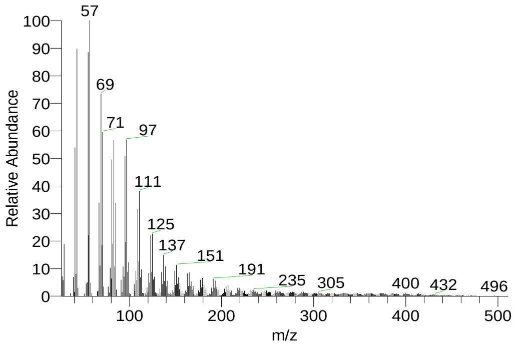

HAHNFETT
Formula , MW 0, CAS# NA, Entry# 391160

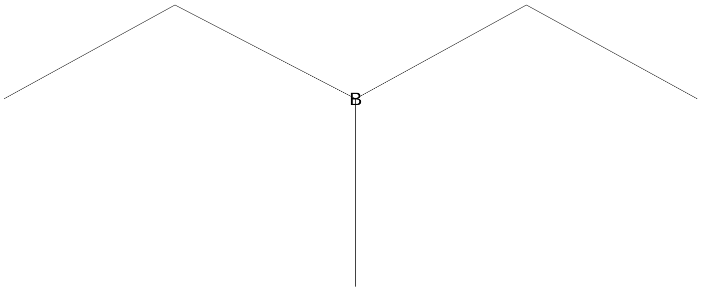

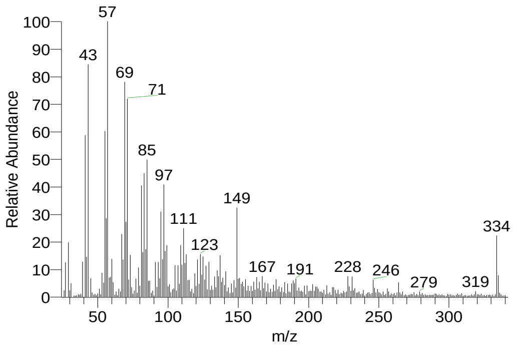

ISOCHIAPIN B
Formula C19H22O6, MW 346, CAS# NA, Entry# 225807

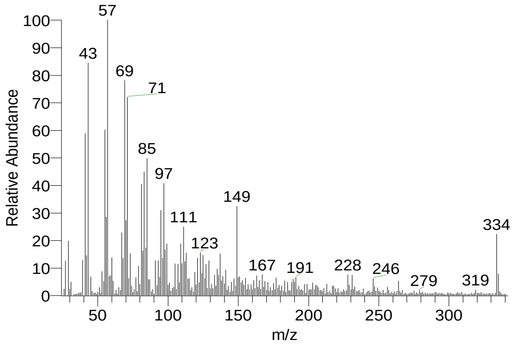

ISOCHIAPIN B %2<
Formula C19H26O6, MW 350, CAS# NA, Entry# 228500

There is no signature data to report.

Library Search Report

shrefa100 #21349 RT: 77.61 AV: 1 NL: 7.35E6
T: + c EI Full ms [50.00-1000.00]

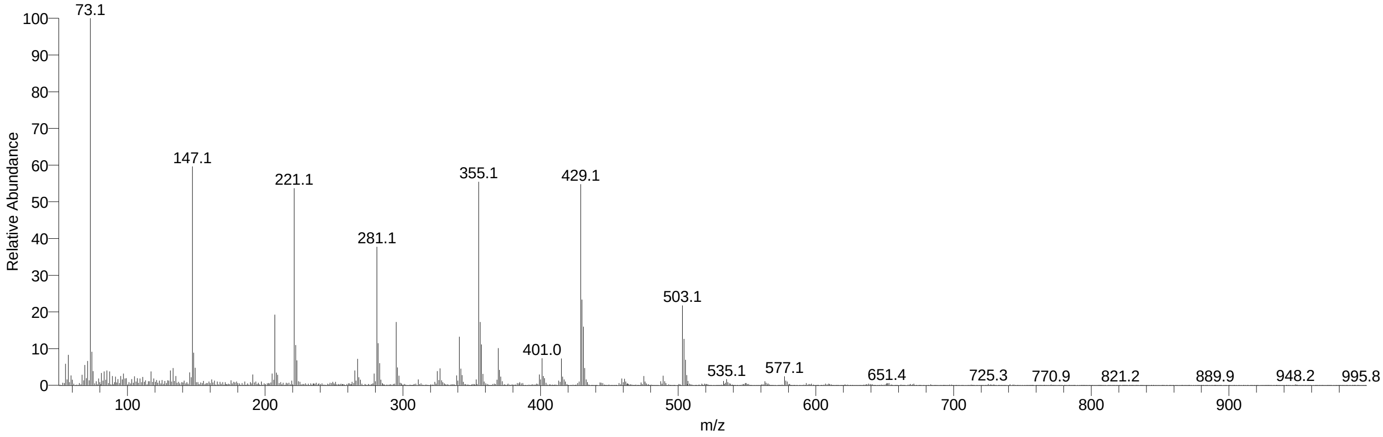

RT	Scan #	Probability	Compound Name	SI	RSI	Cas #	Area	Area %	Library
77.61	21349.000000	29.14	SILICONE OIL	805	860	NA	228208810.35	2.79	WileyRegistry8e
77.61	21349.000000	29.14	SILIKONFETT SE30 (GREVELS)	805	860	NA	228208810.35	2.79	WileyRegistry8e
77.61	21349.000000	18.82	Cyclodecasiloxane, eicosamethyl-	792	833	18772-36-6	228208810.35	2.79	mainlib
77.61	21349.000000	18.82	2,2,4,4,6,6,8,8,10,10,12,12,14,14,16,16,18,18,20,20-ICOSAMETHYLCYCLODECASILOXANE #	792	833	18772-36-6	228208810.35	2.79	WileyRegistry8e
77.61	21349.000000	17.36	1H-PURIN-6-AMINE, [(2-FLUOROPHENYL)METHYL]-	790	855	74421-44-6	228208810.35	2.79	WileyRegistry8e

Hit Spectrum | Compound Structure

SILICONE OIL
Formula , MW 0, CAS# NA, Entry# 305490
SILIKONFETT SE30 (GREVELS)

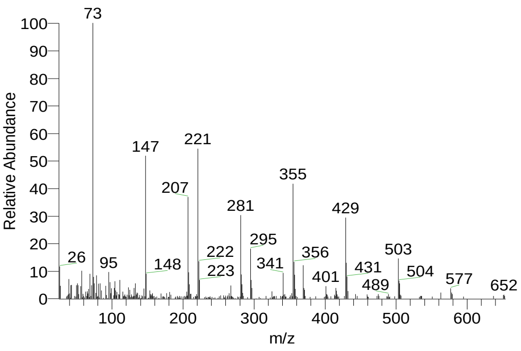

SILIKONFETT SE30 (GREVELS)
Formula , MW 0, CAS# NA, Entry# 392776

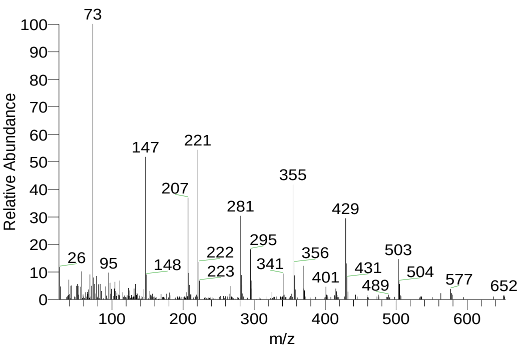

There is no signature data to report.

Library Search Report

Hit Spectrum | Compound Structure

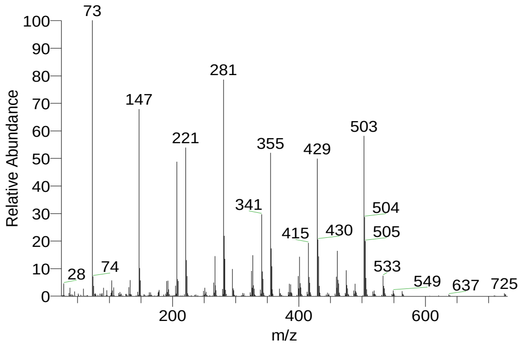

Cyclodecasiloxane, eicosamethyl-
Formula C20H60O10Si10, MW 740, CAS# 18772-36-6, Entry# 47864
2,2,4,4,6,6,8,8,10,10,12,12,14,14,16,16,18,18,20,20-Icosamethylcyclodecasiloxane #

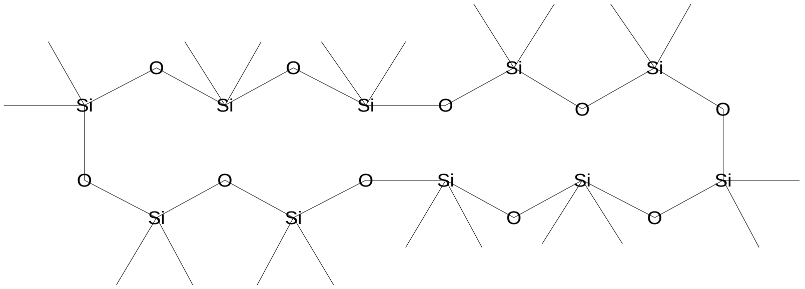

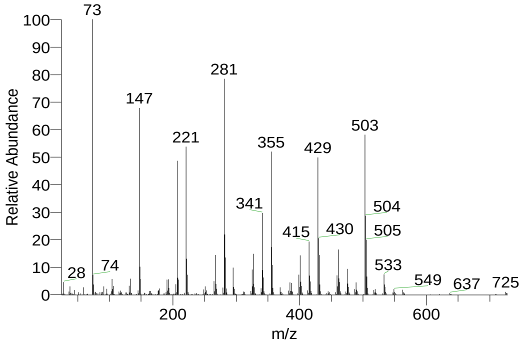

2,2,4,4,6,6,8,8,10,10,12,12,14,14,16,16,18,18,20,20-ICOSAMETHYLCYCLODECASILOXANE #
Formula C20H60O10Si10, MW 740, CAS# 18772-36-6, Entry# 380233
2,2,4,4,6,6,8,8,10,10,12,12,14,14,16,16,18,18,20,20-ICOSAMETHYLCYCLODECASILOXANE

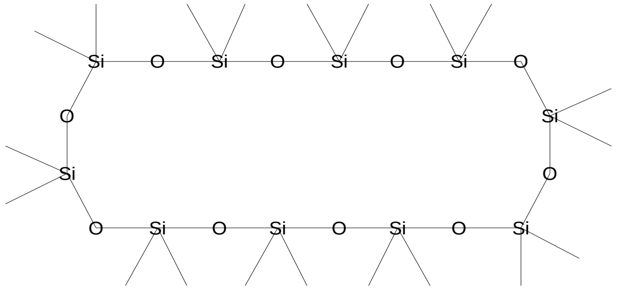

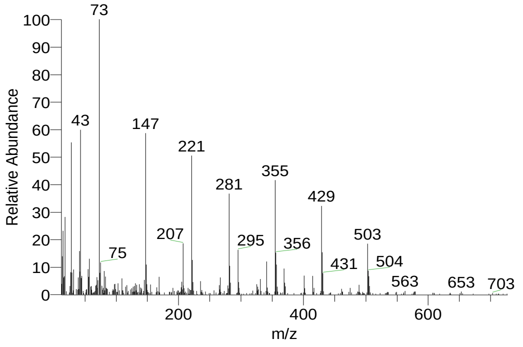

1H-PURIN-6-AMINE, [(2-FLUOROPHENYL)METHYL]-
Formula C12H10FN5, MW 243, CAS# 74421-44-6, Entry# 132518

There is no signature data to report.

Library Search Report

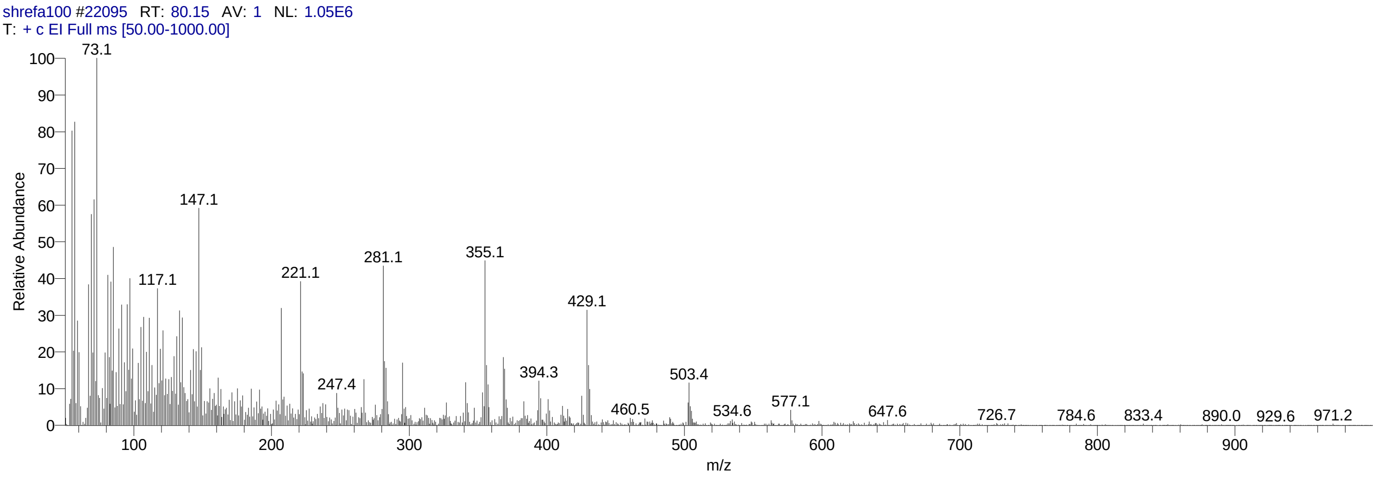

RT	Scan #	Probability	Compound Name	SI	RSI	Cas #	Area	Area %	Library
80.15	22095.000000	14.52	Propanoic acid, 2-(3-acetoxy-4,4,14-trimethylandrost-8-en-17-yl)-	663	717	NA	53377704.24	0.65	mainlib
80.15	22095.000000	14.52	2-(3-ACETOXY-4,4,10,13,14-PENTAMETHYL-2,3,4,5,6,7,10,11,12,13,14,15,16,17-TETRADECAHYDRO-1H-CYCLOPENTA[A]PHENANTHREN-17-YL)-PROPIONI	663	717	NA	53377704.24	0.65	WileyRegistry8e
80.15	22095.000000	8.36	Methyl glycocholate, 3TMS derivative	647	734	57326-16-6	53377704.24	0.65	mainlib
80.15	22095.000000	8.36	GLYCINE, N-[(3à,5á,7à,12à)-24-OXO-3,7,12-TRIS[(TRIMETHYLSILYL)OXY]CHOLAN-24-YL]-, METHYL ESTER	647	734	57326-16-6	53377704.24	0.65	WileyRegistry8e
80.15	22095.000000	6.07	4H-1-BENZOPYRAN-4-ONE, 2-(3,4-DIMETHOXYPHENYL)-3,5-DIHYDROXY-7-METHOXY-	638	793	6068-80-0	53377704.24	0.65	WileyRegistry8e

Hit Spectrum	Compound Structure

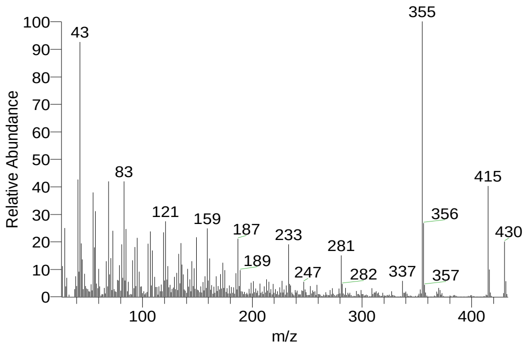

Propanoic acid, 2-(3-acetoxy-4,4,14-trimethylandrost-8-en-17-yl)-
Formula C27H42O4, MW 430, CAS# NA, Entry# 258204

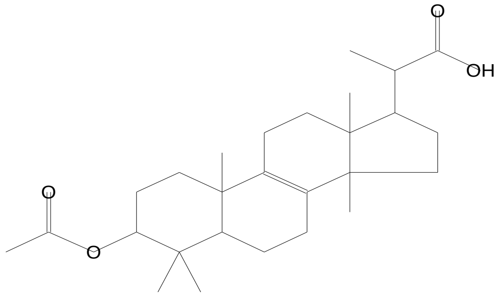

There is no signature data to report.

Library Search Report

Hit Spectrum | Compound Structure

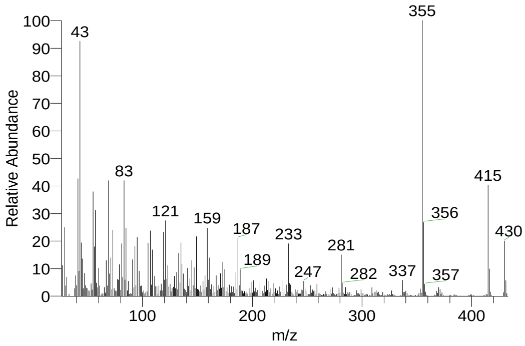

Formula C27H42O4, MW 430, CAS# NA, Entry# 367790

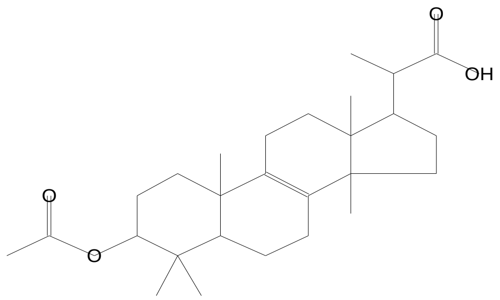

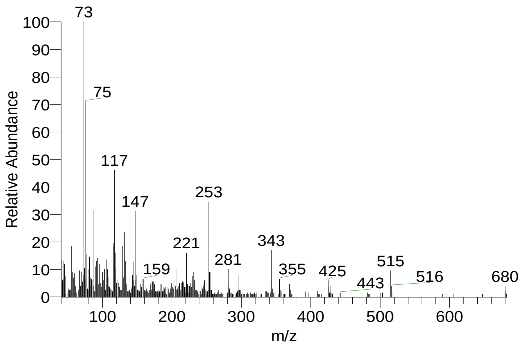

Methyl glycocholate, 3TMS derivative
Formula C36H69NO6Si3, MW 695, CAS# 57326-16-6, Entry# 45192
Glycine, N-[(3à,5á,7à,12à)-24-oxo-3,7,12-tris[(trimethylsilyl)oxy]cholan-24-yl]-, methyl ester

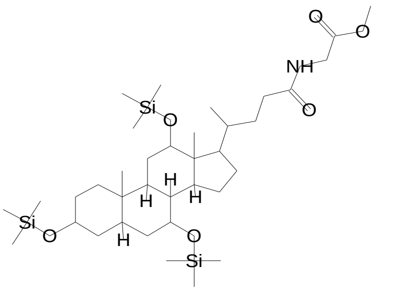

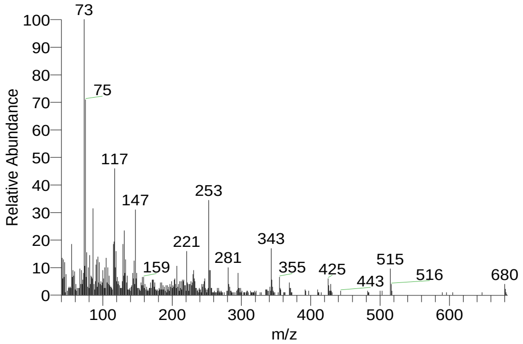

Formula C36H69NO6Si3, MW 695, CAS# 57326-16-6, Entry# 301490
METHYL ((24-OXO-3,7,12-TRIS[(TRIMETHYLSILYL)OXY]CHOLAN-24-YL)AMINO)ACETATE #

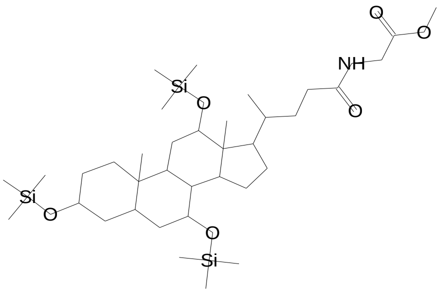

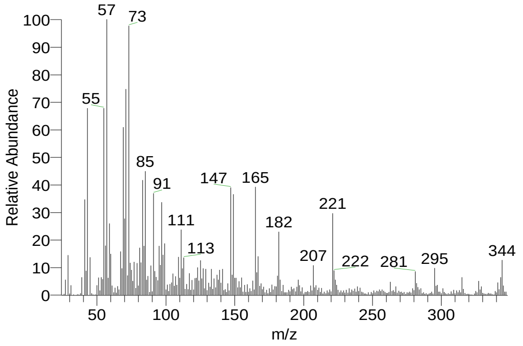

4H-1-BENZOPYRAN-4-ONE, 2-(3,4-DIMETHOXYPHENYL)-3,5-DIHYDROXY-7-METHOXY-
Formula C18H16O7, MW 344, CAS# 6068-80-0, Entry# 224392
3',4',7-TRIMETHYLQUERCETIN

There is no signature data to report.

Library Search Report

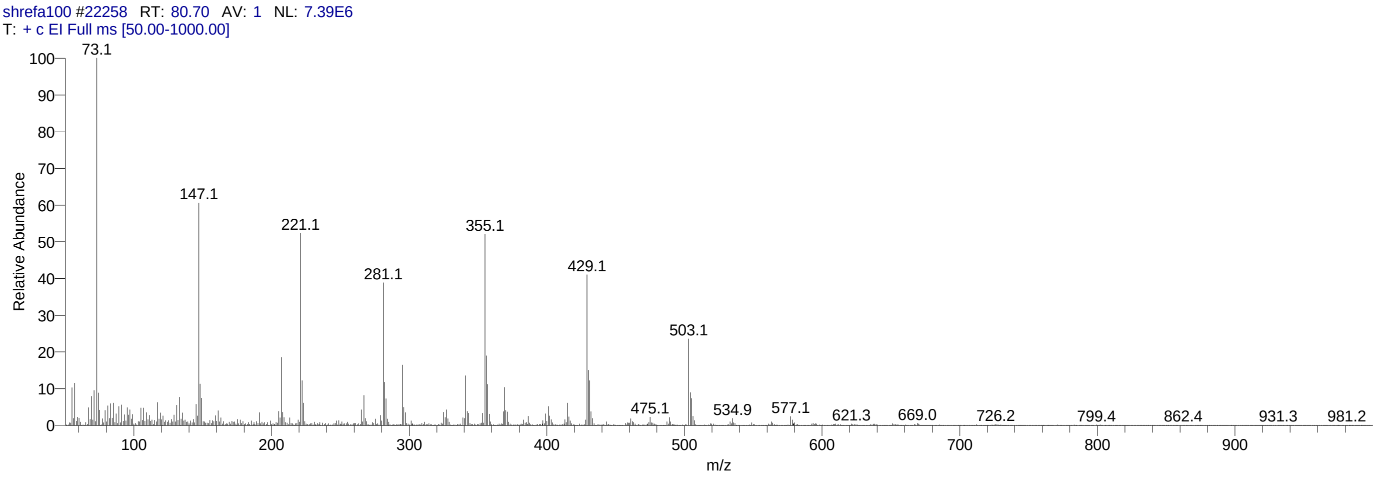

RT	Scan #	Probability	Compound Name	SI	RSI	Cas #	Area	Area %	Library
80.70	22258.000000	32.50	SILICONE OIL	791	861	NA	231727089.49	2.84	WileyRegistry8e
80.70	22258.000000	32.50	SILIKONFETT SE30 (GREVELS)	791	861	NA	231727089.49	2.84	WileyRegistry8e
80.70	22258.000000	19.70	1H-PURIN-6-AMINE, [(2-FLUOROPHENYL)METHYL]-	776	858	74421-44-6	231727089.49	2.84	WileyRegistry8e
80.70	22258.000000	12.33	Cyclodecasiloxane, eicosamethyl-	762	812	18772-36-6	231727089.49	2.84	mainlib
80.70	22258.000000	12.33	2,2,4,4,6,6,8,8,10,10,12,12,14,14,16,16,18,18,20,20-ICOSAMETHYLCYCLODECASILOXANE #	762	812	18772-36-6	231727089.49	2.84	WileyRegistry8e

Hit Spectrum | Compound Structure

SILICONE OIL
Formula , MW 0, CAS# NA, Entry# 305490
SILIKONFETT SE30 (GREVELS)

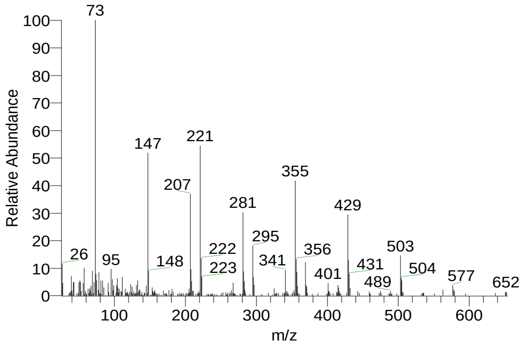

SILIKONFETT SE30 (GREVELS)
Formula , MW 0, CAS# NA, Entry# 392776

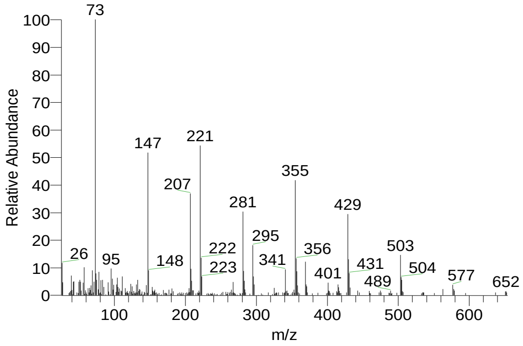

There is no signature data to report.

Library Search Report

Hit Spectrum | Compound Structure

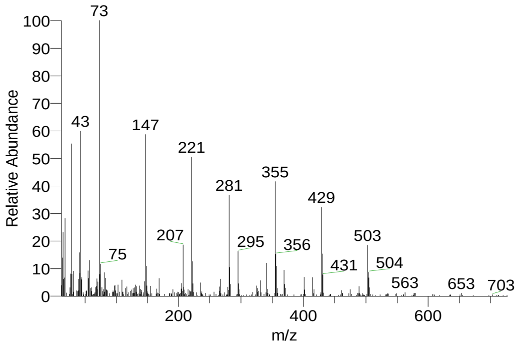

1H-PURIN-6-AMINE, [(2-FLUOROPHENYL)METHYL]-
Formula C12H10FN5, MW 243, CAS# 74421-44-6, Entry# 132518

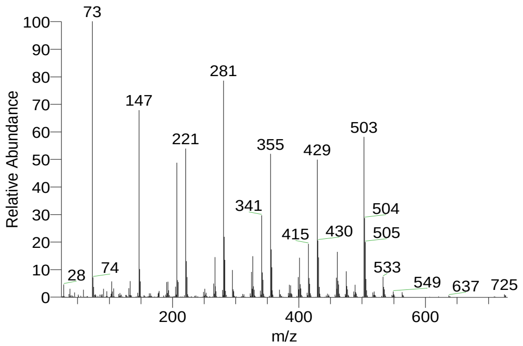

Cyclodecasiloxane, eicosamethyl-
Formula C20H60O10Si10, MW 740, CAS# 18772-36-6, Entry# 47864
2,2,4,4,6,6,8,8,10,10,12,12,14,14,16,16,18,18,20,20-Icosamethylcyclodecasiloxane #

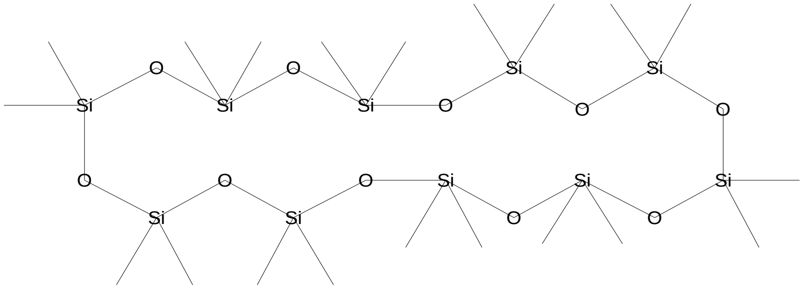

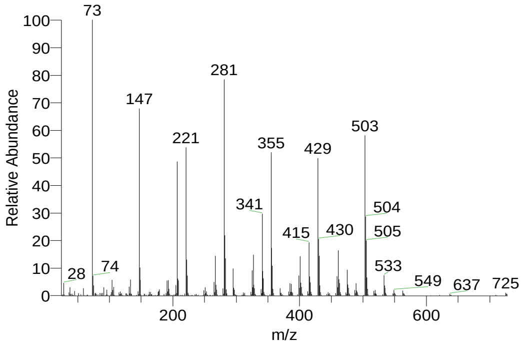

2,2,4,4,6,6,8,8,10,10,12,12,14,14,16,16,18,18,20,20-ICOSAMETHYLCYCLODECASILOXANE #
Formula C20H60O10Si10, MW 740, CAS# 18772-36-6, Entry# 380233
2,2,4,4,6,6,8,8,10,10,12,12,14,14,16,16,18,18,20,20-ICOSAMETHYLCYCLODECASILOXANE

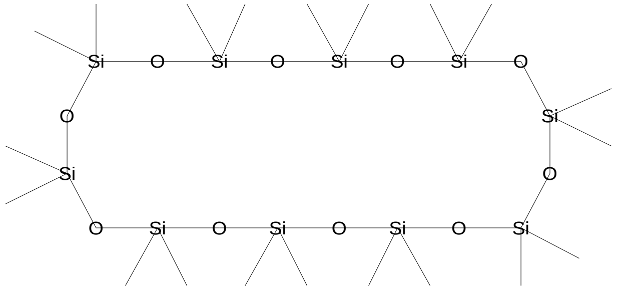

There is no signature data to report.

Library Search Report

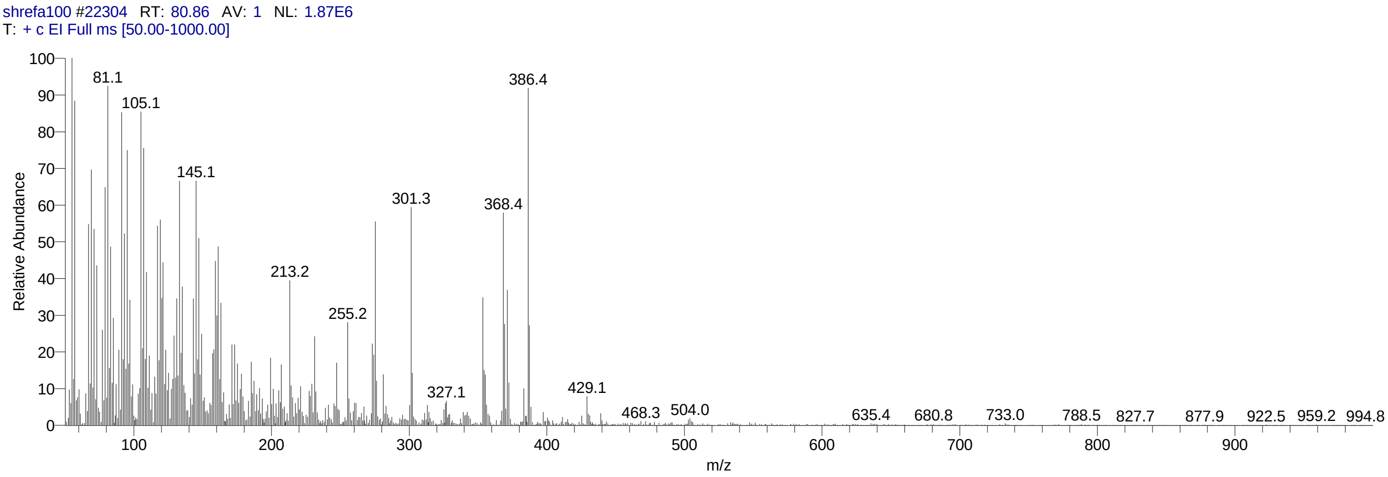

RT	Scan #	Probability	Compound Name	SI	RSI	Cas #	Area	Area %	Library
80.86	22304.000000	35.39	17-(1,5-DIMETHYL-HEXYL)-10,13-DIMETHYL-2,3,4,7,8,9,10,11,12,13,14,15,16,17-TETRADECAHYDRO-1H-CYCLOPENTA[A]PHENANTHREN-3-OL	843	901	NA	243142424.46	2.98	WileyRegistry8e
80.86	22304.000000	23.57	CHOLEST-5-EN-3-OL (3á)-	831	863	57-88-5	243142424.46	2.98	WileyRegistry8e
80.86	22304.000000	23.57	CHOLEST-5-EN-3-OL (3á)-	823	915	57-88-5	243142424.46	2.98	WileyRegistry8e
80.86	22304.000000	23.57	Cholesterol	822	872	57-88-5	243142424.46	2.98	replib
80.86	22304.000000	23.57	CHOLEST-5-EN-3-OL (3á)-	822	862	57-88-5	243142424.46	2.98	WileyRegistry8e

Hit Spectrum | Compound Structure

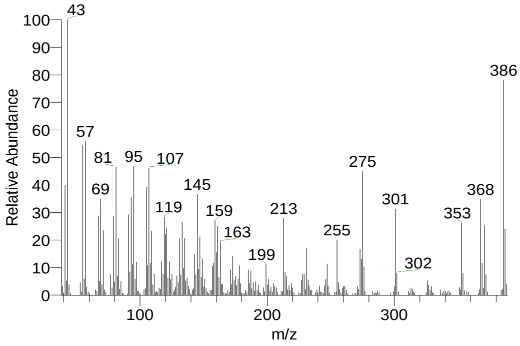

Formula C27H46O, MW 386, CAS# NA, Entry# 361933
CHOLEST-5-EN-3-OL

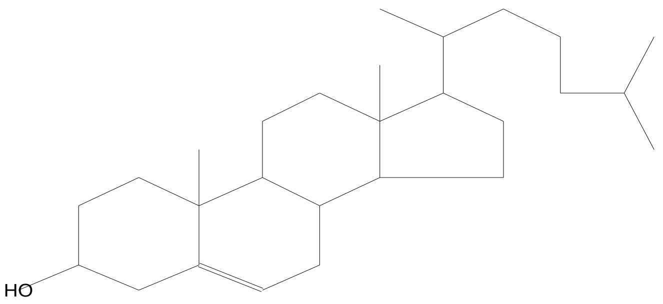

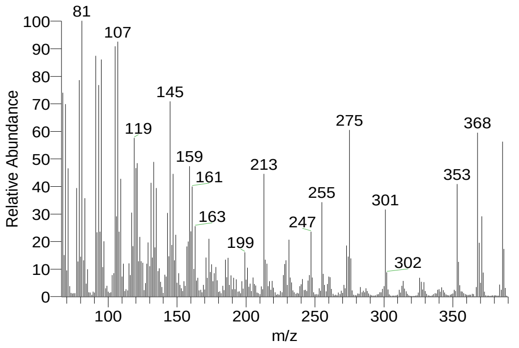

CHOLEST-5-EN-3-OL (3á)-
Formula C27H46O, MW 386, CAS# 57-88-5, Entry# 249655

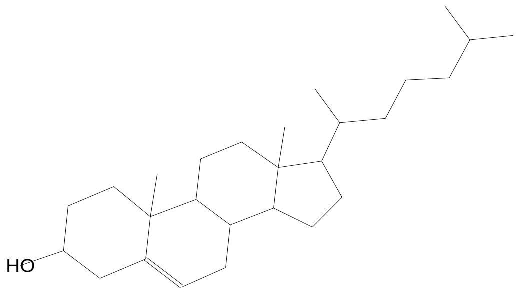

There is no signature data to report.

Library Search Report

Hit Spectrum | Compound Structure

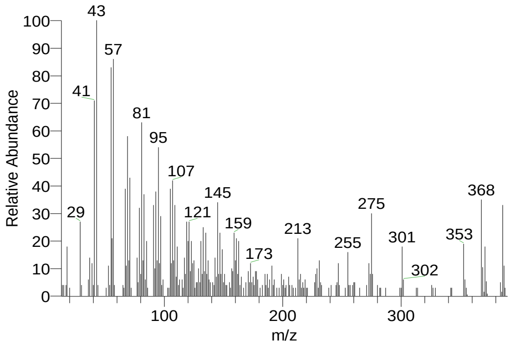

CHOLEST-5-EN-3-OL (3á)-
Formula C27H46O, MW 386, CAS# 57-88-5, Entry# 249608

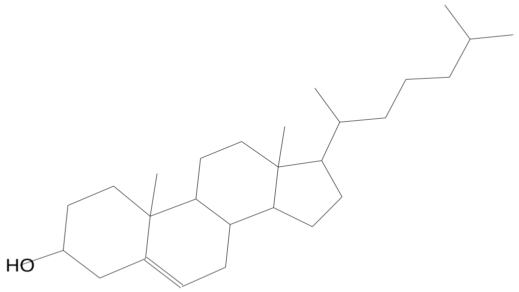

SI 822, RSI 872, replib, Entry# 38936, CAS# 57-88-5, Cholesterol

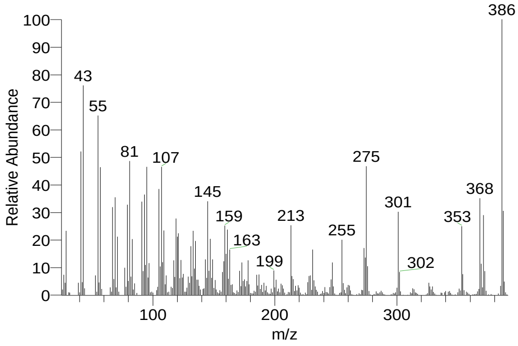

Cholesterol
Formula C27H46O, MW 386, CAS# 57-88-5, Entry# 38936
Cholest-5-en-3-ol (3á)-

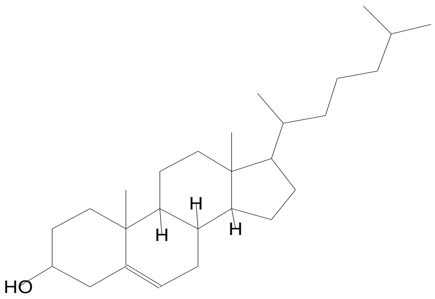

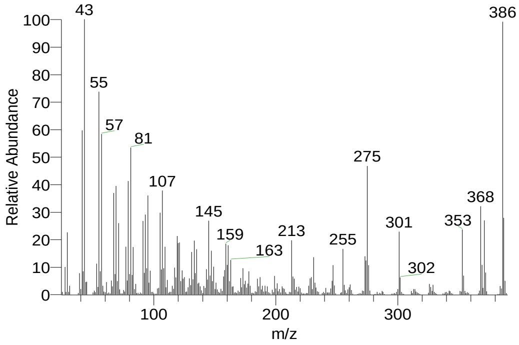

CHOLEST-5-EN-3-OL (3á)-
Formula C27H46O, MW 386, CAS# 57-88-5, Entry# 373188

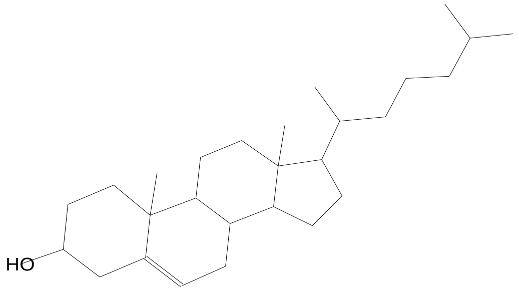

There is no signature data to report.

Library Search Report

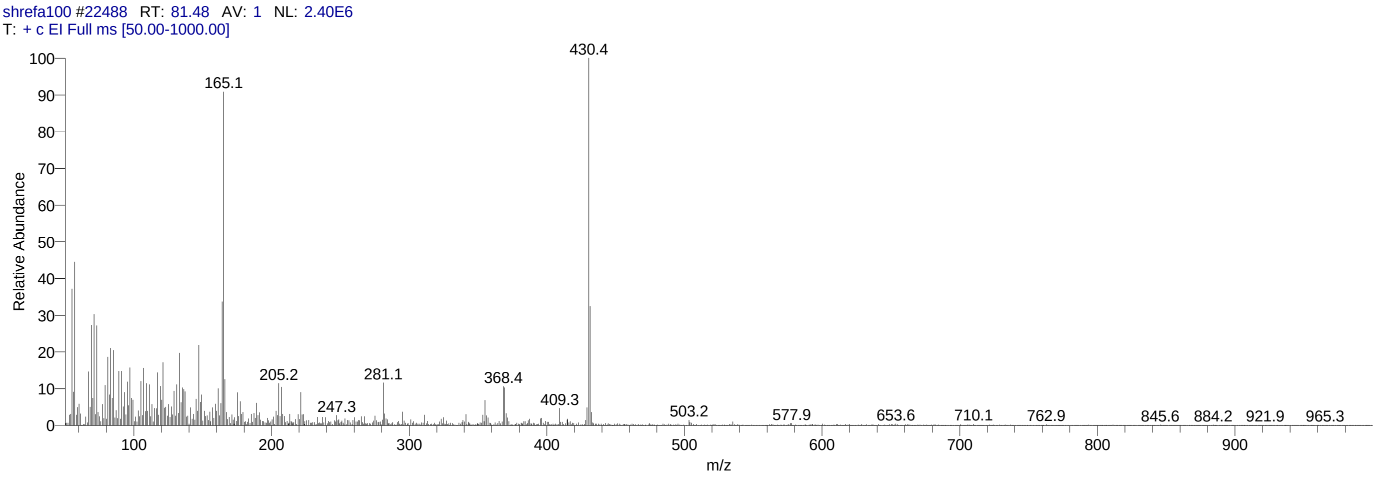

RT	Scan #	Probability	Compound Name	SI	RSI	Cas #	Area	Area %	Library
81.48	22488.000000	33.33	Vitamin E	676	767	59-02-9	44584091.57	0.55	replib
81.48	22488.000000	33.33	2H-1-BENZOPYRAN-6-OL, 3,4-DIHYDRO-2,5,7,8-TETRAMETHYL-2-(4,8,12-TRIMETHYLTRIDECYL)-, [2R-[2R*(4R*,8R*)]]-	676	767	59-02-9	44584091.57	0.55	WileyRegistry8e
81.48	22488.000000	33.33	2,5,7,8-TETRAMETHYL-2-(4,8,12-TRIMETHYLTRIDECYL)-6-CHROMANOL	676	767	NA	44584091.57	0.55	WileyRegistry8e
81.48	22488.000000	8.24	Cholest-4-ene, 3á-(methoxymethoxy)-	640	669	4707-85-1	44584091.57	0.55	mainlib
81.48	22488.000000	8.24	3-(METHOXYMETHOXY)CHOLEST-4-ENE #	640	669	4707-85-1	44584091.57	0.55	WileyRegistry8e

Hit Spectrum

Compound Structure

SI 676, RSI 767, replib, Entry# 29464, CAS# 59-02-9, Vitamin E

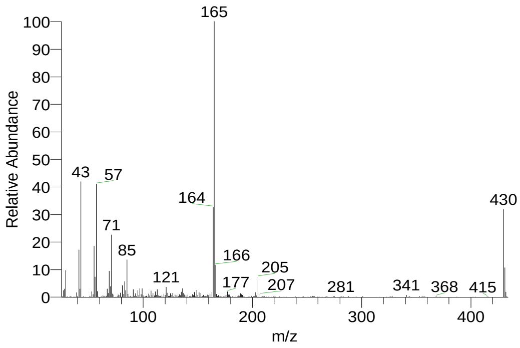

Vitamin E
Formula C29H50O2, MW 430, CAS# 59-02-9, Entry# 29464

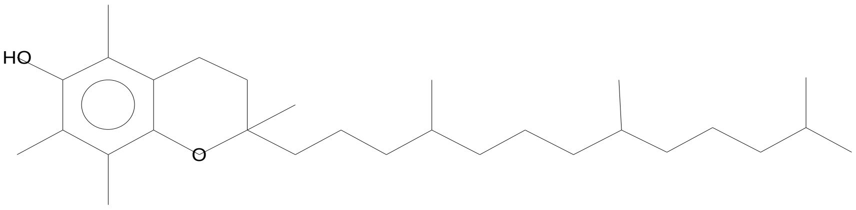

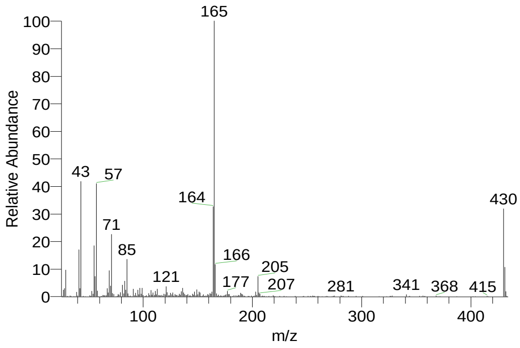

Formula C29H50O2, MW 430, CAS# 59-02-9, Entry# 268385
2,5,7,8-TETRAMETHYL-2-(4,8,12-TRIMETHYLTRIDECYL)-6-CHROMANOL #

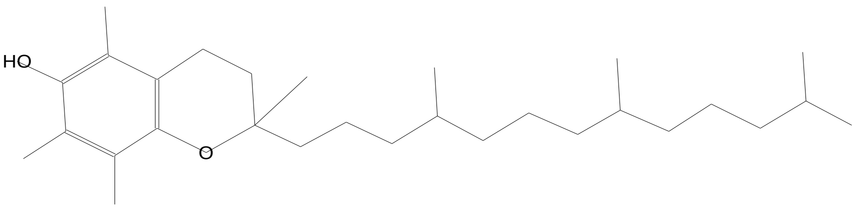

There is no signature data to report.

Library Search Report

Hit Spectrum	Compound Structure

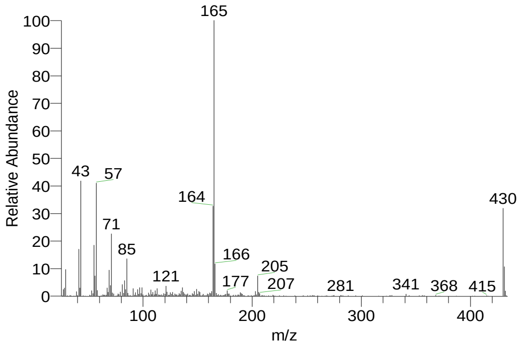

2,5,7,8-TETRAMETHYL-2-(4,8,12-TRIMETHYLTRIDECYL)-6-CHROMANOL
Formula C29H50O2, MW 430, CAS# NA, Entry# 387607
ALPHA-TOCOPHEROL (VITAMIN E)

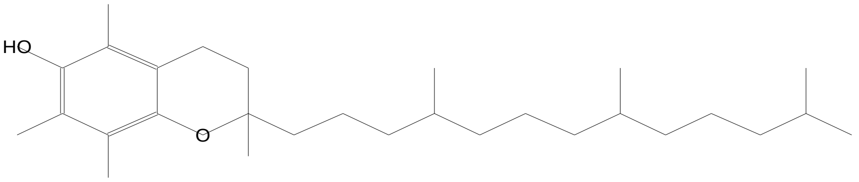

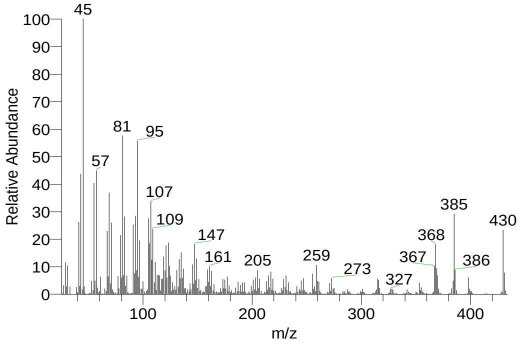

Cholest-4-ene, 3á-(methoxymethoxy)-
Formula C29H50O2, MW 430, CAS# 4707-85-1, Entry# 18816
3-(Methoxymethoxy)cholest-4-ene #

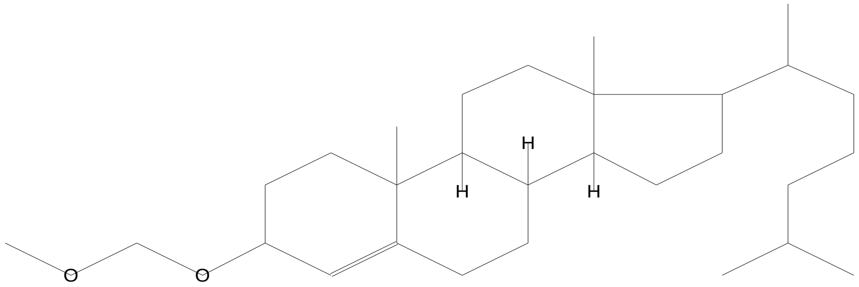

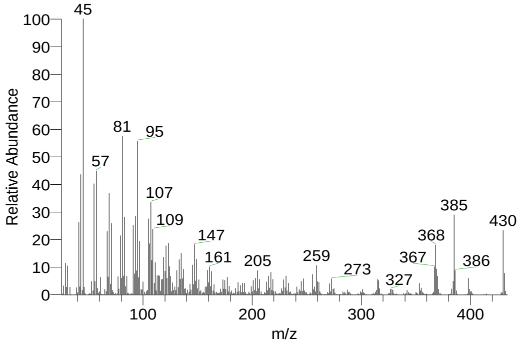

3-(METHOXYMETHOXY)CHOLEST-4-ENE #
Formula C29H50O2, MW 430, CAS# 4707-85-1, Entry# 373114
3-(METHOXYMETHOXY)CHOLEST-4-ENE

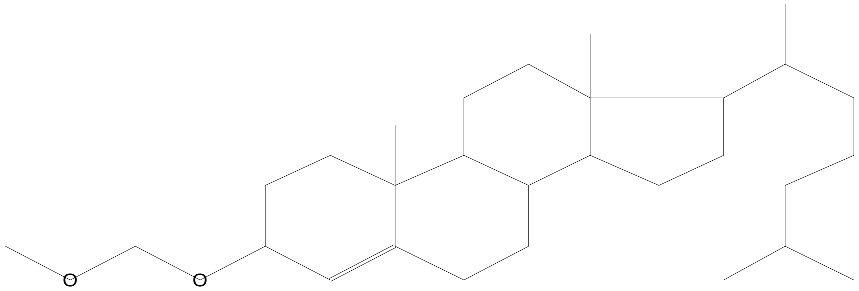

There is no signature data to report.

Library Search Report

shrefa100 #23035 RT: 83.35 AV: 1 NL: 1.60E6
T: + c EI Full ms [50.00-1000.00]

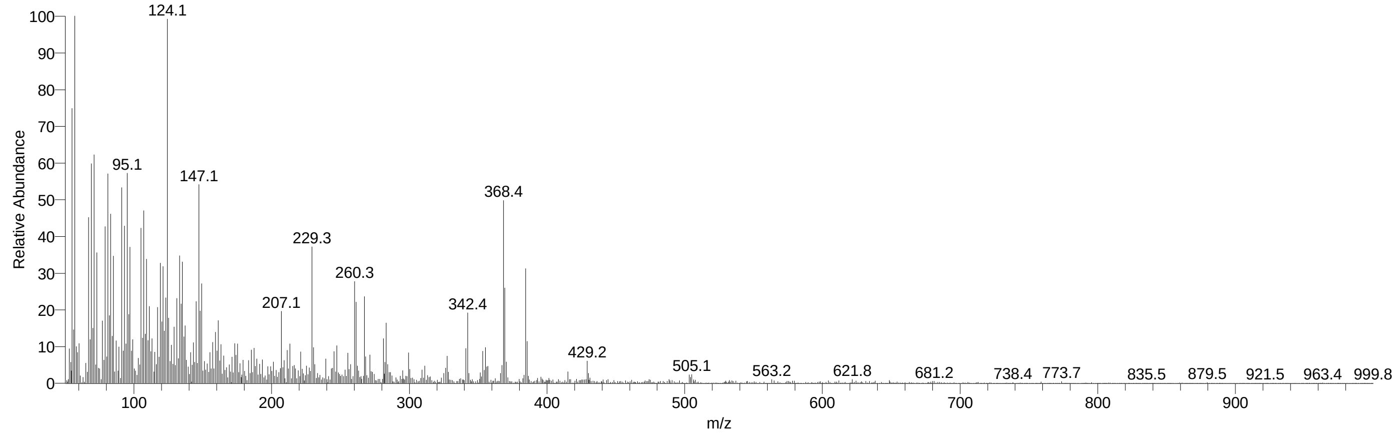

RT	Scan #	Probability	Compound Name	SI	RSI	Cas #	Area	Area %	Library
83.35	23035.000000	34.40	CHOLEST-5-EN-3-ONE	752	804	601-54-7	97696049.64	1.20	WileyRegistry8e
83.35	23035.000000	34.40	Cholest-5-en-3-one	752	803	601-54-7	97696049.64	1.20	replib
83.35	23035.000000	9.19	1-Hydroxy-2-(2,3,4,6-tetra-O-acetyl-beta-d-glucopyranosyl)-9H-xanthene-3,6,7-triyl triacetate	720	759	NA	97696049.64	1.20	mainlib
83.35	23035.000000	7.22	Cholest-4-en-3-one	714	783	601-57-0	97696049.64	1.20	mainlib
83.35	23035.000000	7.22	Cholest-4-en-3-one	709	765	601-57-0	97696049.64	1.20	replib

Hit Spectrum | Compound Structure

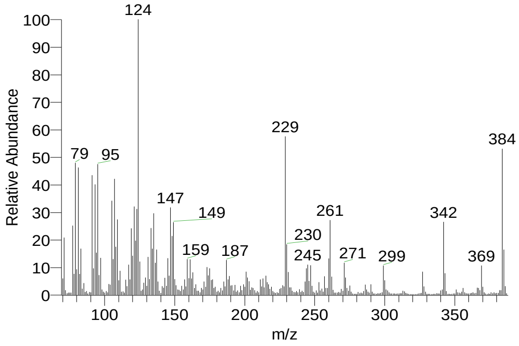

CHOLEST-5-EN-3-ONE
Formula $C_{27}H_{44}O$, MW 384, CAS# 601-54-7, Entry# 248497
.DELTA.(SUP5)-CHOLESTENONE

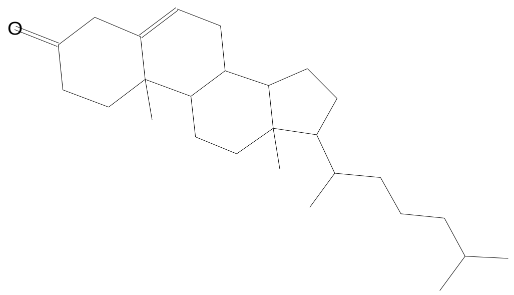

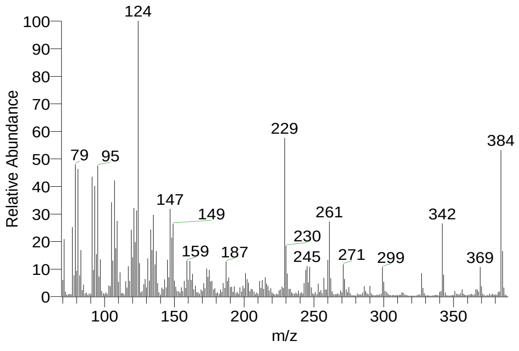

Cholest-5-en-3-one
Formula $C_{27}H_{44}O$, MW 384, CAS# 601-54-7, Entry# 23181
Cholesterone

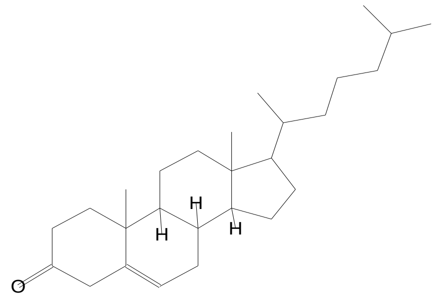

There is no signature data to report.

Library Search Report

Hit Spectrum | Compound Structure

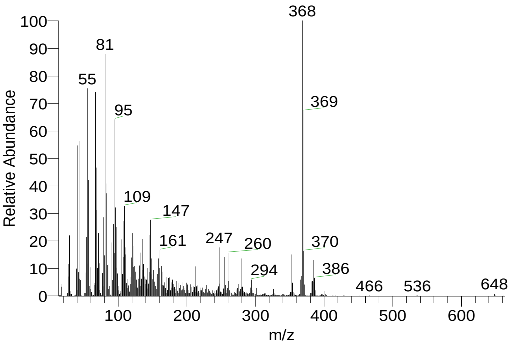

1-Hydroxy-2-(2,3,4,6-tetra-O-acetyl-beta-d-glucopyranosyl)-9H-xanthene-3,6,7-triyl triacetate
Formula C33H34O18, MW 718, CAS# NA, Entry# 259790
2,6-Bis(acetyloxy)-8-hydroxy-7-[(2,3,4,6-tetra-O-acetylhexopyranosyl)oxy]-9H-xanthen-3-yl acetate #

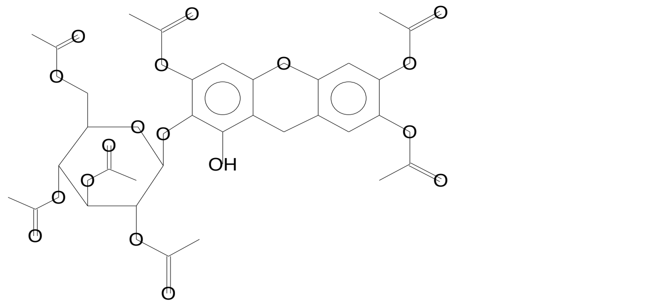

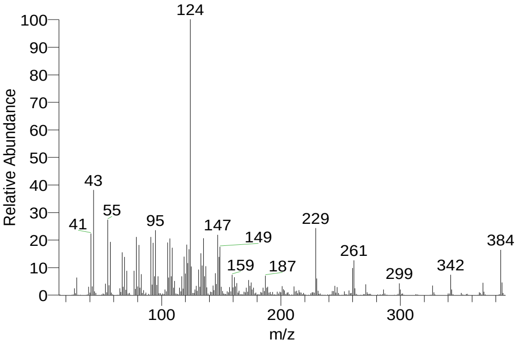

Cholest-4-en-3-one
Formula C27H44O, MW 384, CAS# 601-57-0, Entry# 117783
3-Oxocholest-4-ene

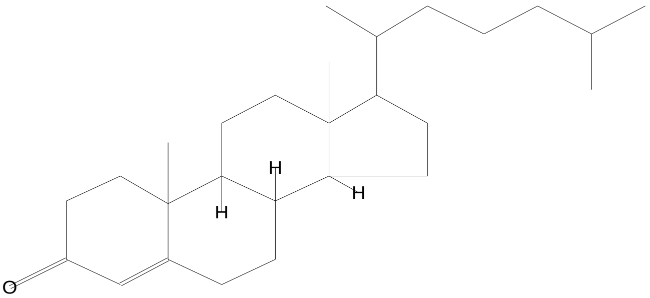

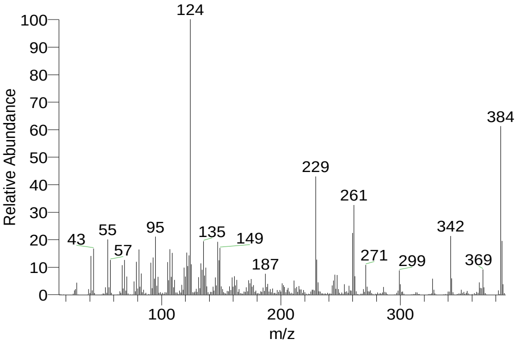

Cholest-4-en-3-one
Formula C27H44O, MW 384, CAS# 601-57-0, Entry# 23195
3-Oxocholest-4-ene

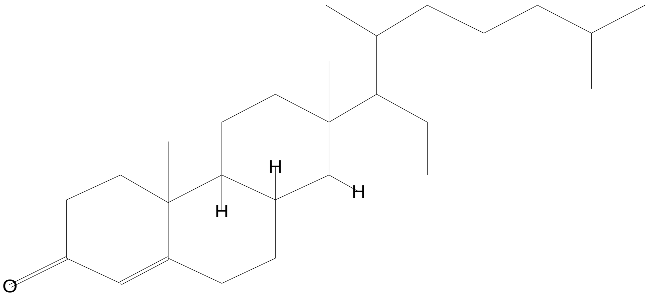

There is no signature data to report.

Library Search Report

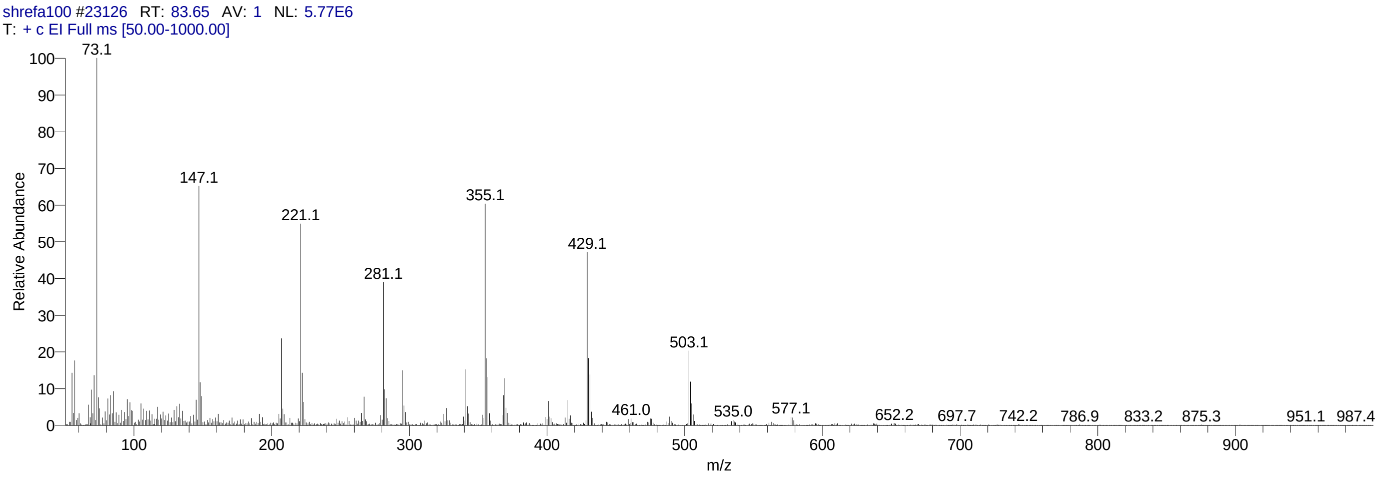

RT	Scan #	Probability	Compound Name	SI	RSI	Cas #	Area	Area %	Library
83.65	23126.000000	32.07	1H-PURIN-6-AMINE, [(2-FLUOROPHENYL)METHYL]-	791	877	74421-44-6	188182191.98	2.30	WileyRegistry8e
83.65	23126.000000	29.58	SILICONE OIL	789	865	NA	188182191.98	2.30	WileyRegistry8e
83.65	23126.000000	29.58	SILIKONFETT SE30 (GREVELS)	789	865	NA	188182191.98	2.30	WileyRegistry8e
83.65	23126.000000	6.75	Cyclodecasiloxane, eicosamethyl-	748	808	18772-36-6	188182191.98	2.30	mainlib
83.65	23126.000000	6.75	2,2,4,4,6,6,8,8,10,10,12,12,14,14,16,16,18,18,20,20-ICOSAMETHYLCYCLODECASILOXANE #	748	808	18772-36-6	188182191.98	2.30	WileyRegistry8e

Hit Spectrum	Compound Structure

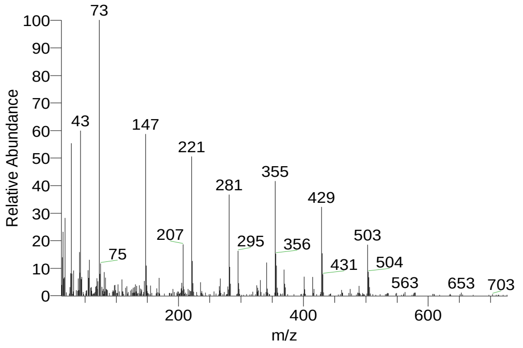

1H-PURIN-6-AMINE, [(2-FLUOROPHENYL)METHYL]-
Formula C12H10FN5, MW 243, CAS# 74421-44-6, Entry# 132518

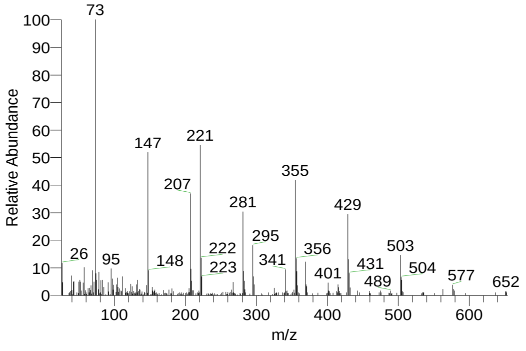

SILICONE OIL
Formula , MW 0, CAS# NA, Entry# 305490
SILIKONFETT SE30 (GREVELS)

There is no signature data to report.

Library Search Report

Hit Spectrum | Compound Structure

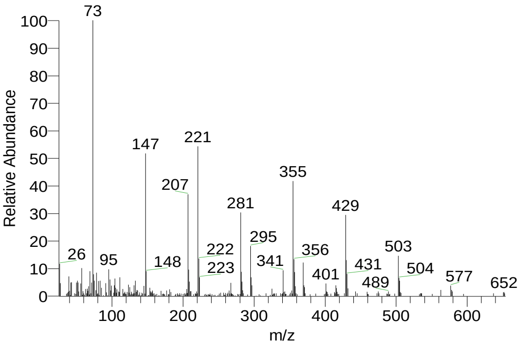

SILIKONFETT SE30 (GREVELS)
Formula , MW 0, CAS# NA, Entry# 392776

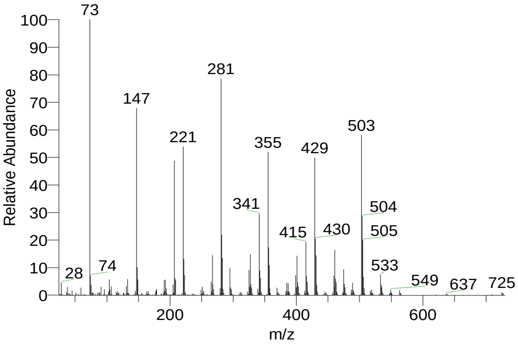

Cyclodecasiloxane, eicosamethyl-
Formula C20H60O10Si10, MW 740, CAS# 18772-36-6, Entry# 47864
2,2,4,4,6,6,8,8,10,10,12,12,14,14,16,16,18,18,20,20-Icosamethylcyclodecasiloxane #

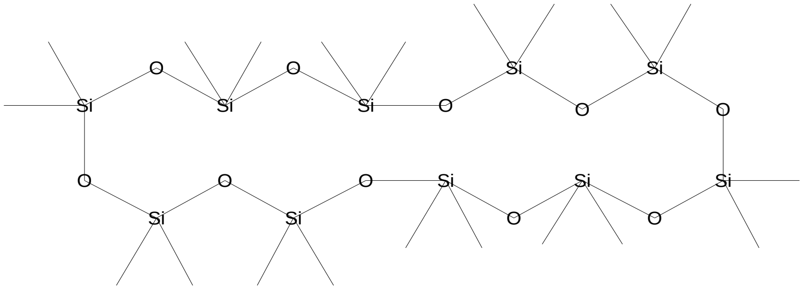

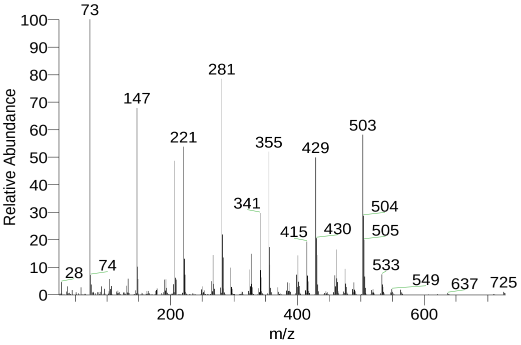

2,2,4,4,6,6,8,8,10,10,12,12,14,14,16,16,18,18,20,20-ICOSAMETHYLCYCLODECASILOXANE #
Formula C20H60O10Si10, MW 740, CAS# 18772-36-6, Entry# 380233
2,2,4,4,6,6,8,8,10,10,12,12,14,14,16,16,18,18,20,20-ICOSAMETHYLCYCLODECASILOXANE

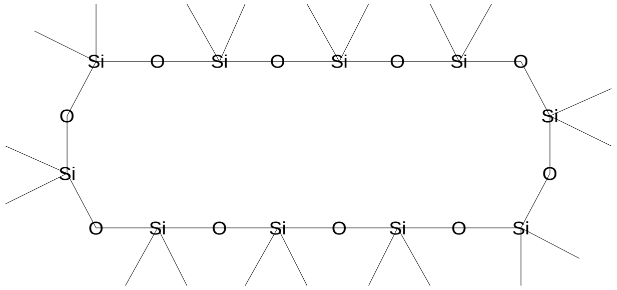

There is no signature data to report.

Library Search Report

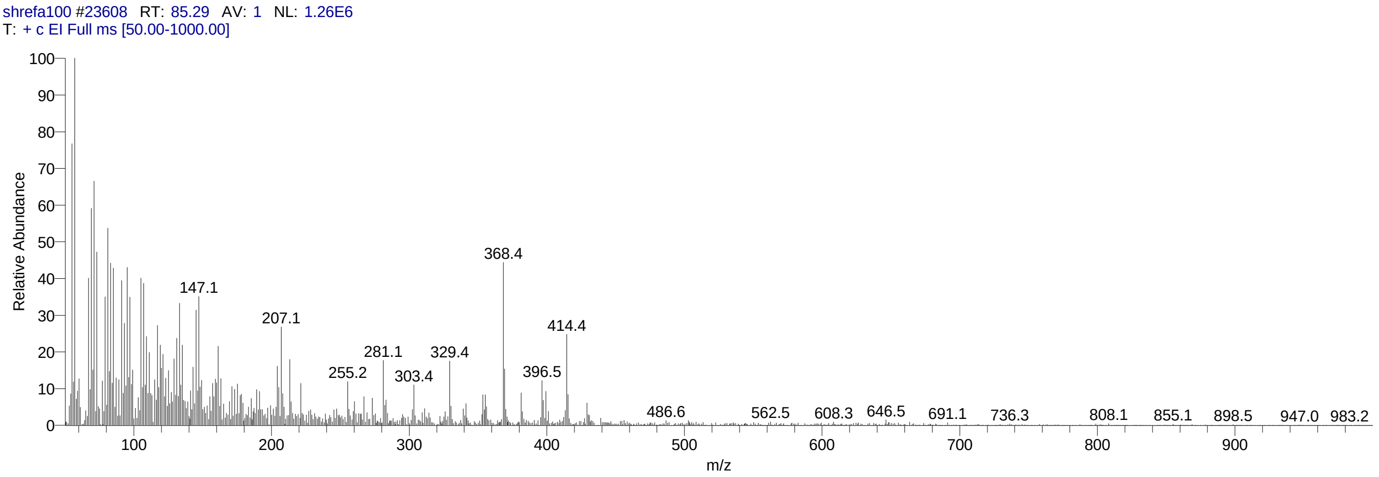

RT	Scan #	Probability	Compound Name	SI	RSI	Cas #	Area	Area %	Library
85.29	23608.000000	14.79	03027205002 FLAVONE 4'-OH,5-OH,7-DI-O-GLUCOSIDE	707	740	NA	42929260.10	0.53	WileyRegistry8e
85.29	23608.000000	8.96	á-Sitosterol	692	813	83-46-5	42929260.10	0.53	mainlib
85.29	23608.000000	6.86	ç-Sitosterol	685	768	83-47-6	42929260.10	0.53	mainlib
85.29	23608.000000	6.86	STIGMAST-5-EN-3-OL, (3á,24S)-	685	768	83-47-6	42929260.10	0.53	WileyRegistry8e
85.29	23608.000000	6.60	Ethyl iso-allocholate	684	773	NA	42929260.10	0.53	mainlib

Hit Spectrum | Compound Structure

03027205002 FLAVONE 4'-OH,5-OH,7-DI-O-GLUCOSIDE
Formula C27H30O15, MW 594, CAS# NA, Entry# 296184

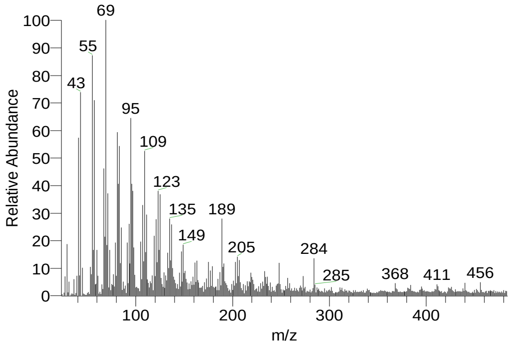

SI 692, RSI 813, mainlib, Entry# 7621, CAS# 83-46-5, á-Sitosterol

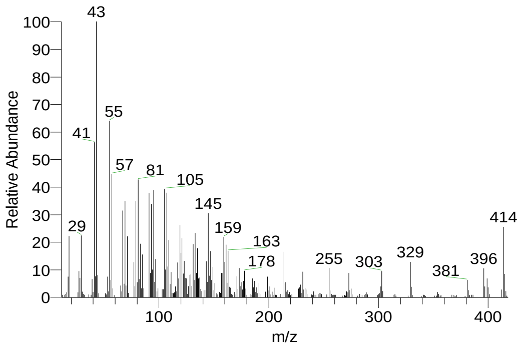

á-Sitosterol
Formula C29H50O, MW 414, CAS# 83-46-5, Entry# 7621
Stigmast-5-en-3-ol, (3á)-

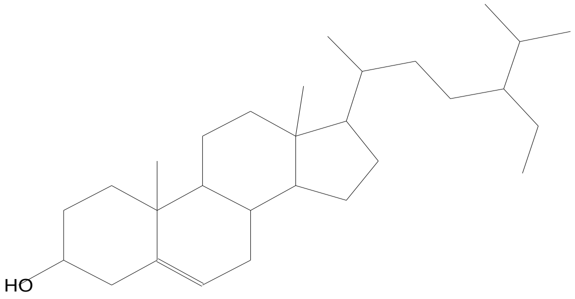

There is no signature data to report.

Library Search Report

Hit Spectrum	Compound Structure

SI 685, RSI 768, mainlib, Entry# 7753, CAS# 83-47-6, ç-Sitosterol

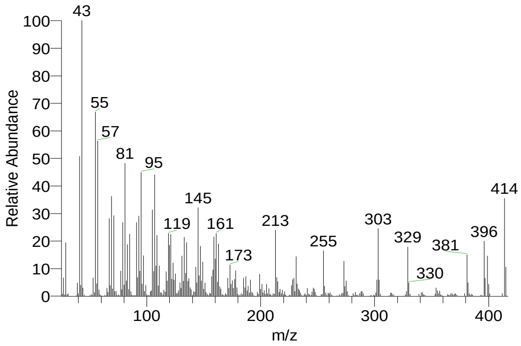

ç-Sitosterol
Formula C29H50O, MW 414, CAS# 83-47-6, Entry# 7753
Stigmast-5-en-3-ol, (3á,24S)-

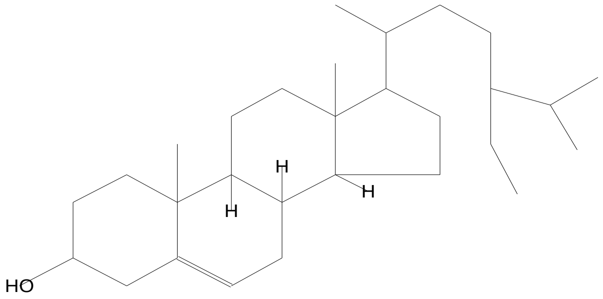

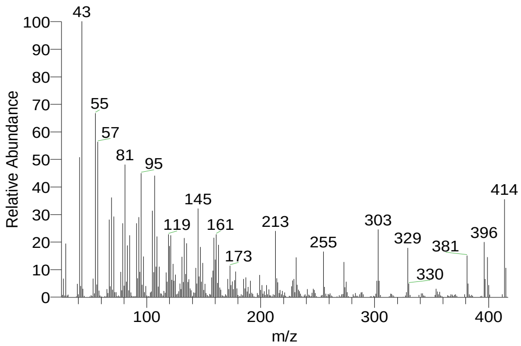

STIGMAST-5-EN-3-OL, (3á,24S)-
Formula C29H50O, MW 414, CAS# 83-47-6, Entry# 387446
STIGMAST-5-EN-3-OL #

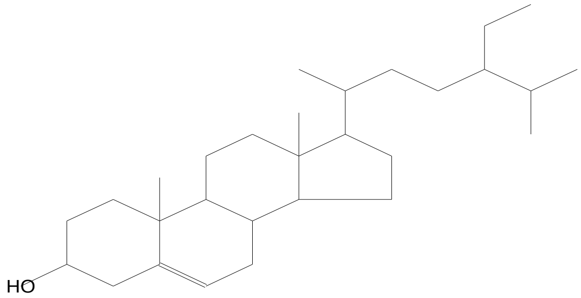

SI 684, RSI 773, mainlib, Entry# 7555, CAS# NA, Ethyl iso-allocholate

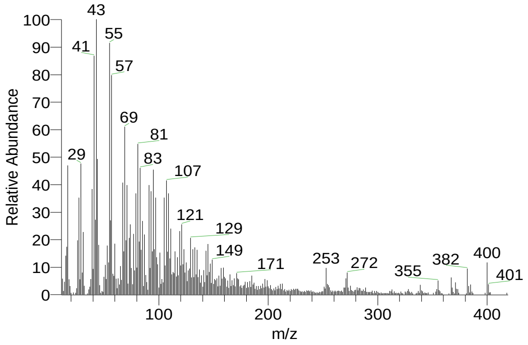

Ethyl iso-allocholate
Formula C26H44O5, MW 436, CAS# NA, Entry# 7555
Ethyl 3,7,12-trihydroxycholan-24-oate #

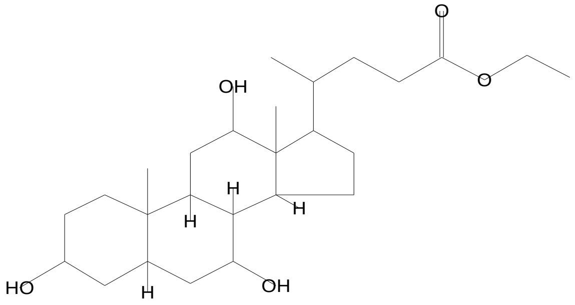

There is no signature data to report.

Library Search Report

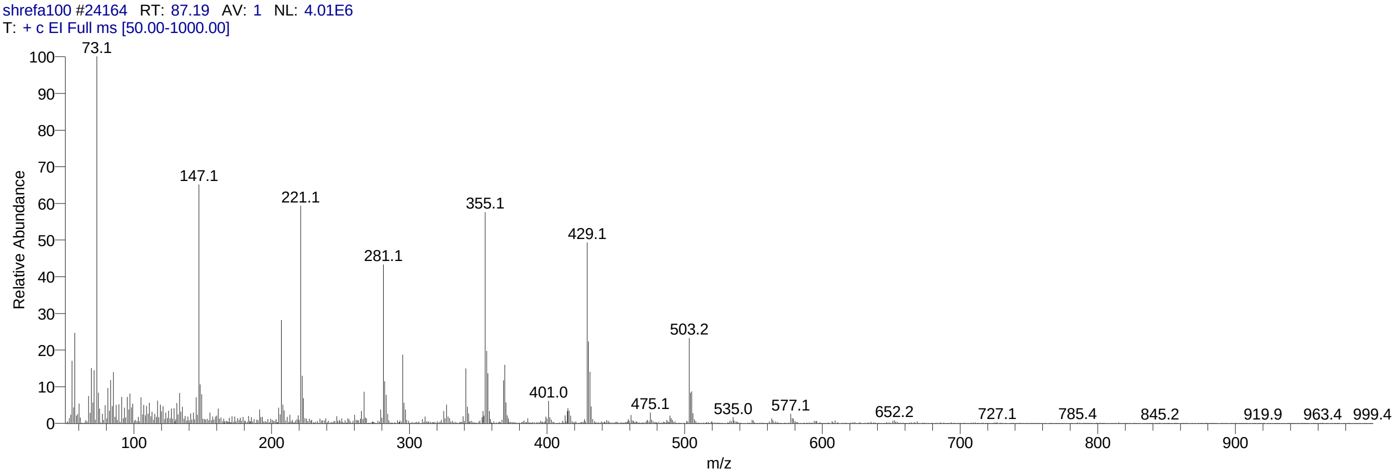

RT	Scan #	Probability	Compound Name	SI	RSI	Cas #	Area	Area %	Library
87.19	24164.000000	33.41	SILICONE OIL	781	863	NA	171135713.58	2.10	WileyRegistry8e
87.19	24164.000000	33.41	SILIKONFETT SE30 (GREVELS)	781	863	NA	171135713.58	2.10	WileyRegistry8e
87.19	24164.000000	25.59	1H-PURIN-6-AMINE, [(2-FLUOROPHENYL)METHYL]-	774	868	74421-44-6	171135713.58	2.10	WileyRegistry8e
87.19	24164.000000	5.01	Cyclodecasiloxane, eicosamethyl-	720	789	18772-36-6	171135713.58	2.10	mainlib
87.19	24164.000000	5.01	2,2,4,4,6,6,8,8,10,10,12,12,14,14,16,16,18,18,20,20-ICOSAMETHYLCYCLODECASILOXANE #	720	789	18772-36-6	171135713.58	2.10	WileyRegistry8e

Hit Spectrum | Compound Structure

SILICONE OIL
Formula , MW 0, CAS# NA, Entry# 305490
SILIKONFETT SE30 (GREVELS)

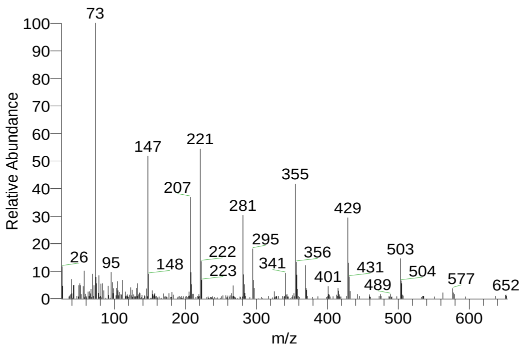

SILIKONFETT SE30 (GREVELS)
Formula , MW 0, CAS# NA, Entry# 392776

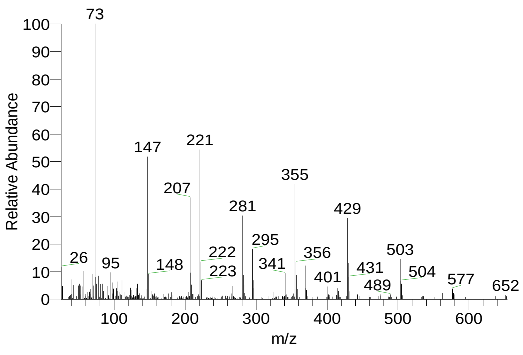

There is no signature data to report.

Library Search Report

Hit Spectrum	Compound Structure

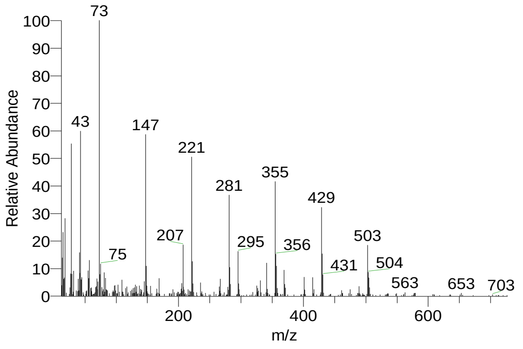

1H-PURIN-6-AMINE, [(2-FLUOROPHENYL)METHYL]-
Formula C12H10FN5, MW 243, CAS# 74421-44-6, Entry# 132518

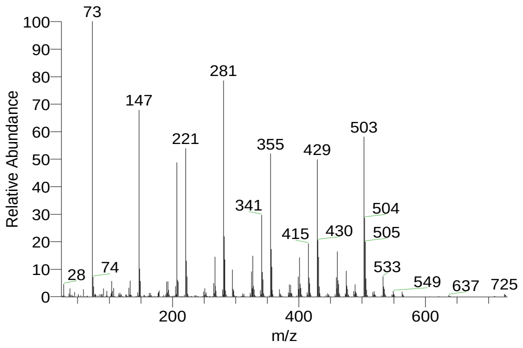

Cyclodecasiloxane, eicosamethyl-
Formula C20H60O10Si10, MW 740, CAS# 18772-36-6, Entry# 47864
2,2,4,4,6,6,8,8,10,10,12,12,14,14,16,16,18,18,20,20-Icosamethylcyclodecasiloxane #

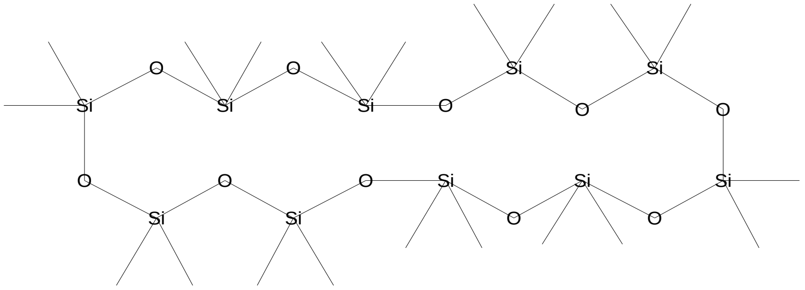

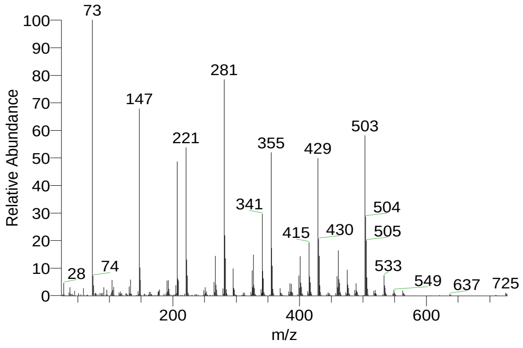

2,2,4,4,6,6,8,8,10,10,12,12,14,14,16,16,18,18,20,20-ICOSAMETHYLCYCLODECASILOXANE #
Formula C20H60O10Si10, MW 740, CAS# 18772-36-6, Entry# 380233
2,2,4,4,6,6,8,8,10,10,12,12,14,14,16,16,18,18,20,20-ICOSAMETHYLCYCLODECASILOXANE

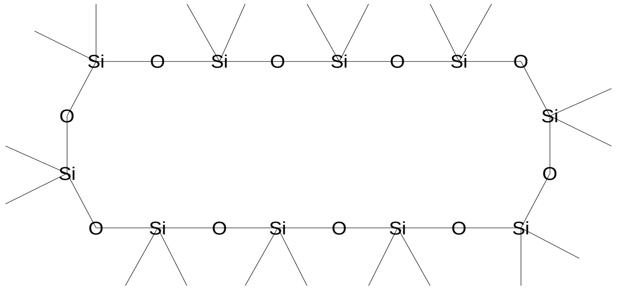

There is no signature data to report.

Library Search Report

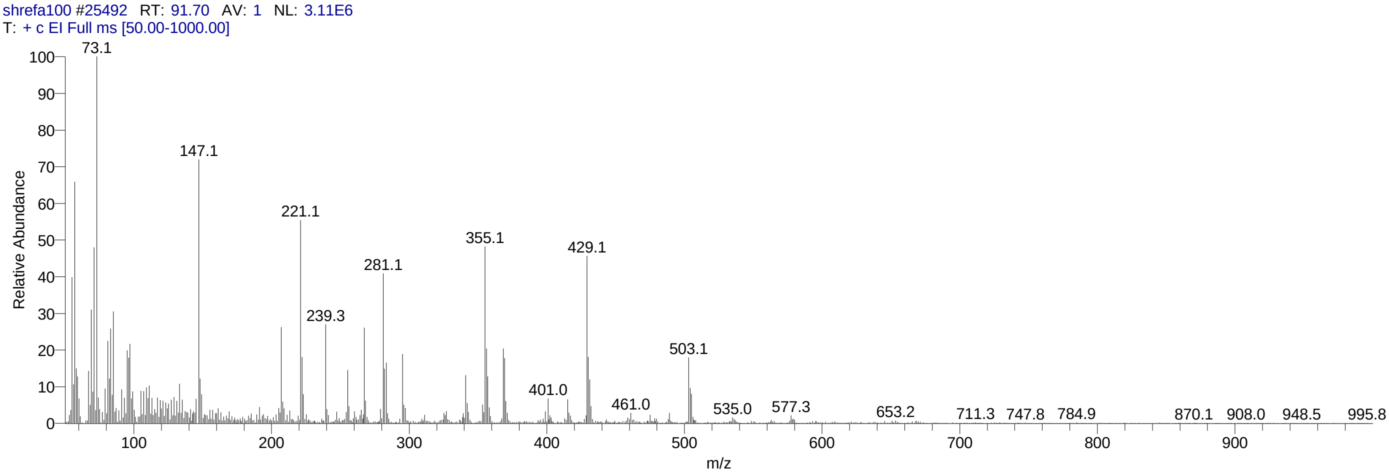

RT	Scan #	Probability	Compound Name	SI	RSI	Cas #	Area	Area %	Library
91.70	25492.000000	33.35	SILICONE OIL	726	829	NA	214758762.41	2.63	WileyRegistry8e
91.70	25492.000000	33.35	SILIKONFETT SE30 (GREVELS)	726	829	NA	214758762.41	2.63	WileyRegistry8e
91.70	25492.000000	22.87	1H-PURIN-6-AMINE, [(2-FLUOROPHENYL)METHYL]-	715	823	74421-44-6	214758762.41	2.63	WileyRegistry8e
91.70	25492.000000	4.28	Cyclodecasiloxane, eicosamethyl-	658	745	18772-36-6	214758762.41	2.63	mainlib
91.70	25492.000000	4.28	2,2,4,4,6,6,8,8,10,10,12,12,14,14,16,16,18,18,20,20-ICOSAMETHYLCYCLODECASILOXANE #	658	745	18772-36-6	214758762.41	2.63	WileyRegistry8e

Hit Spectrum	Compound Structure

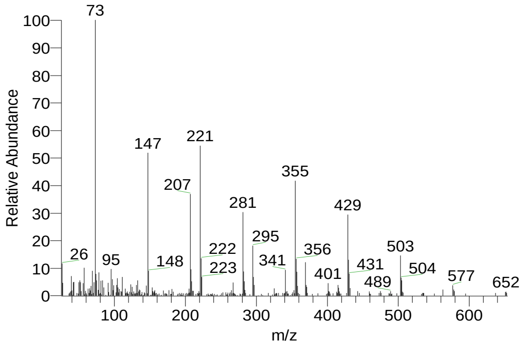

SILICONE OIL
Formula , MW 0, CAS# NA, Entry# 305490
SILIKONFETT SE30 (GREVELS)

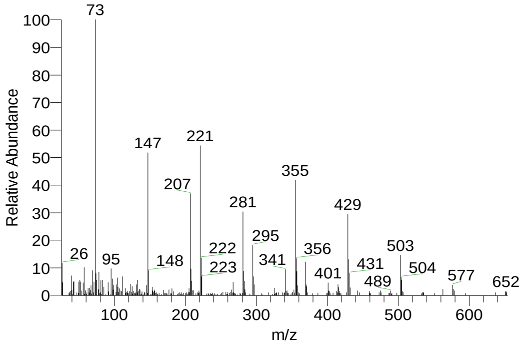

SILIKONFETT SE30 (GREVELS)
Formula , MW 0, CAS# NA, Entry# 392776

There is no signature data to report.

Library Search Report

Hit Spectrum | Compound Structure

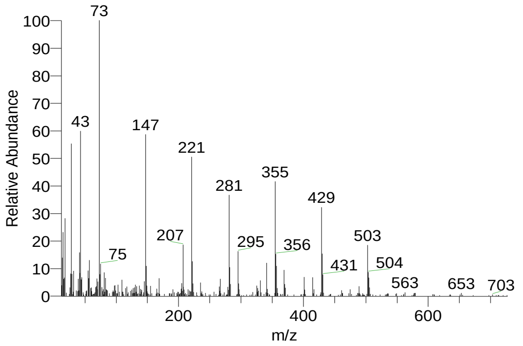

1H-PURIN-6-AMINE, [(2-FLUOROPHENYL)METHYL]-
Formula C12H10FN5, MW 243, CAS# 74421-44-6, Entry# 132518

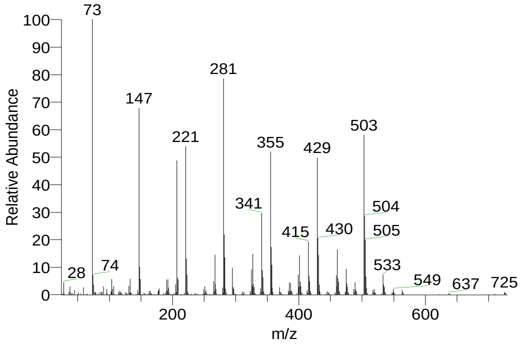

Cyclodecasiloxane, eicosamethyl-
Formula C20H60O10Si10, MW 740, CAS# 18772-36-6, Entry# 47864
2,2,4,4,6,6,8,8,10,10,12,12,14,14,16,16,18,18,20,20-Icosamethylcyclodecasiloxane #

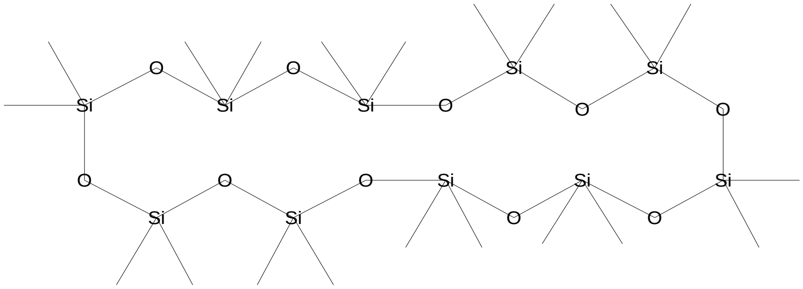

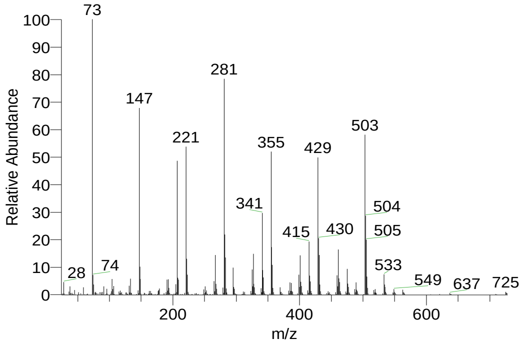

2,2,4,4,6,6,8,8,10,10,12,12,14,14,16,16,18,18,20,20-ICOSAMETHYLCYCLODECASILOXANE #
Formula C20H60O10Si10, MW 740, CAS# 18772-36-6, Entry# 380233
2,2,4,4,6,6,8,8,10,10,12,12,14,14,16,16,18,18,20,20-ICOSAMETHYLCYCLODECASILOXANE

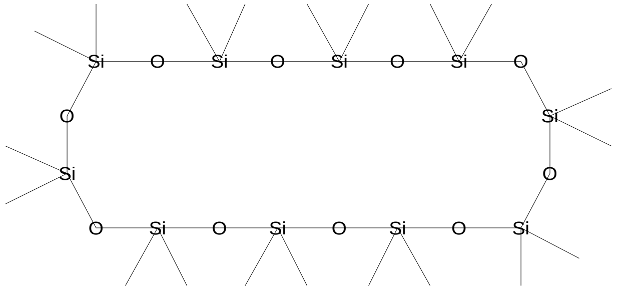

There is no signature data to report.
